# Vasectomy and Photoperiodic Regimen Modify the Protein Profile, Hormonal Content and Antioxidant Enzymes Activity of Ram Seminal Plasma

**DOI:** 10.3390/ijms21218063

**Published:** 2020-10-29

**Authors:** Melissa Carvajal-Serna, Meriem Fatnassi, Felipe Torres-Ruda, Jaime Antonio Cardozo, Henry Grajales-Lombana, Mohamed Hammadi, Jose Alfonso Abecia, Teresa Muiño-Blanco, Rosaura Pérez-Pe, Jose Álvaro Cebrián-Pérez, Adriana Casao

**Affiliations:** 1 Grupo BIOFITER (Biología, Fisiología y Tecnologías de la Reproducción), Departamento de Bioquímica y Biología Molecular y Celular, Instituto Universitario de Investigación en Ciencias Ambientales de Aragón (IUCA), Facultad de Veterinaria, Universidad de Zaragoza, 50013 Zaragoza, Spain; melissac@unizar.es (M.C.-S.); alf@unizar.es (J.A.A.); muino@unizar.es (T.M.-B.); rosperez@unizar.es (R.P.-P.); pcebrian@unizar.es (J.Á.C.-P.); 2 Departamento de Producción Animal, Facultad de Medicina Veterinaria y de Zootecnia, Universidad Nacional de Colombia, Bogotá 111321, Colombia; daftorresr@unal.edu.co (F.T.-R.); hagrajalesl@unal.edu.co (H.G.-L.); 3 Livestock and Wildlife Laboratory, Arid Lands Institute, University of Gabès, Médenine 6029, Tunisia; fatnassi_meriem@yahoo.com (M.F.); mohamed.hammadi@ira.rnrt.tn (M.H.); 4 Researcher Center Tibaitatá, AGROSAVIA (Corporación Colombiana de Investigación Agropecuaria), Bogotá 250047, Colombia; jcardozo@agrosavia.co

**Keywords:** melatonin, testosterone, antioxidant enzymes, vasectomy, DIGE, seminal plasma, ram

## Abstract

This work aimed to determine the contribution of the testis and epididymis and the effect of the photoperiodic regimen on ram seminal plasma (SP). Semen was collected from 15 mature rams located in an equatorial (Colombian Creole and Romney Marsh, eight intact and two vasectomized) or a temperate climate (Rasa Aragonesa, three intact and two vasectomized). SP proteins were analyzed by Bradford, SDS-PAGE and difference gel electrophoresis (DIGE). Melatonin and testosterone concentrations were quantified by ELISA, and activity of glutathione peroxidase (GPx), glutathione reductase (GRD), and catalase by enzymatic assays. Vasectomy increased protein concentration and the intensity of high molecular weight bands (*p* < 0.001), with no differences between breeds. DIGE revealed the absence of six proteins in vasectomized rams: angiotensin-converting enzyme, lactotransferrin, phosphoglycerate kinase, sorbitol dehydrogenase, epididymal secretory glutathione peroxidase and epididymal secretory protein E1. Vasectomy also decreased melatonin concentrations in seasonal rams, and testosterone in all of them (*p* < 0.001), but did not affect antioxidant enzyme activity. Equatorial rams showed lower melatonin and testosterone concentration (*p* < 0.01) and catalase, but higher GPx activity (*p* < 0.05). In conclusion, vasectomy modifies the protein profile and hormonal content of ram seminal plasma, whereas the exposure to a constant photoperiod affects hormonal concentration and antioxidant enzymes activity.

## 1. Introduction

Seminal plasma (SP) is a complex fluid secreted mainly by the testis, epididymis and accessory glands, although some contribution from the spermatozoa [1] has also been identified. SP composition varies among species, mainly due to differences in size and secretory capacity of the accessory glands [2]. SP is composed of proteins, amino acids, enzymes, ions, lipids, sugars, hormones and cytokines, each of these components having essential functions for the spermatozoa (reviewed in [3]). Moreover, several elements of seminal plasma, specially proteins [4], but also others, such as fructose [5] and noncoding microRNAs [6], are related with fertility. Seminal plasma proteome analyses has been used to identify fertility markers in several species, such as human [7], boar [8], bull [9,10] or dromedary camel [11].

Additionally, SP analysis after vasectomy is a useful tool for identifying which constituents of this fluid originate from the testis and epididymis. A previous study of human vasectomized patients identified several proteins unique either to control or post-vasectomized patients [12]. Vasectomy also decreased alkaline phosphatase in alpacas [13], and several biochemical components, including protein composition, in dogs [14]. In the ram, vasectomy increases SP protein concentration [15], while it decreases the high molecular weight proteins [16], and eliminates the membrane vesicles, which suggests a testicular or epididymal origin. However, there are no reports on the effect of vasectomy on the proteomic composition of ram seminal plasma.

Besides proteins, other SP components necessary for the sperm function are antioxidant enzymes and hormones. SP antioxidant enzymes, namely glutathione peroxidase (GPx), glutathione reductase (GRD), superoxide dismutase (SOD) and catalase, protect the spermatozoa from oxidative damage, and have been related with male fertility [17,18]. The origin of these SP antioxidant enzymes is unclear. Several isoforms of GPx are secreted by the epididymis [19], although some contribution of the accessory gland has also been identified [20,21]. GRD, SOD and catalase are likely secreted by the accessory sex glands [21,22]. To the best of our knowledge, no report on the source of ram SP antioxidant enzymes has been published.

As regards hormones, testosterone and melatonin can be found in ram seminal plasma [23]. Testosterone is secreted by the Leydig cells of the testes; thus, a testicular origin of the seminal plasma testosterone is expected. Nonetheless, testosterone also enters the general circulation after its secretion [24], and a systemic source cannot be ruled out either. In contrast, melatonin is secreted mainly by the pineal gland [25]; thus, a pineal origin of seminal plasma melatonin is likely. However, we recently demonstrated the presence of melatonin-synthesizing enzymes in the testis [26], thus this SP melatonin could also be of testicular origin. The levels of testosterone [27] and melatonin [28] in seminal plasma have been connected to male fertility. The beneficial effects of SP melatonin could been related to its antioxidant properties [29,30], whereas the levels of SP testosterone has been proposed as a spermatogenesis marker [31] in human.

Ovine is a seasonal species, and its reproductive activity is regulated by the photoperiod through melatonin secretion [32,33]. In rams located in temperate regions (>30 °C and <45 °C north or south latitudes, 18L:6D light regime), differences in seminal plasma protein and hormonal content, and in antioxidant enzyme activity, vary between reproductive and non-reproductive seasons [23,34]. However, in rams located in equatorial climates (between 10° N and 10° S, 12L:12D), variations in the composition of SP are most likely related to food intake or climatic factors [35]. Nonetheless, the differences in SP between rams located in temperate and equatorial climates has never been studied before.

Thus, the objectives of this work were: 1) to determine the contribution of the testis, epididymis and accessory sex glands secretions to protein and hormonal concentrations, and to antioxidant enzyme activity in ram seminal plasma and 2) to elucidate whether the photoperiodic regimen can affect the seminal plasma composition of intact and vasectomized rams.

## 2. Results

No differences were found in protein, hormonal concentration or antioxidant enzyme activity between the two analyzed non-seasonal breeds (Creole and Romney Marsh), either intact or vasectomized (Appendix A, Table A1). Thus, data from intact or vasectomized rams of both non-seasonal breeds were pooled for further analysis.

### 2.1. Vasectomy Modifies the Protein Profile of Ram Seminal Plasma

**Figure 1:**
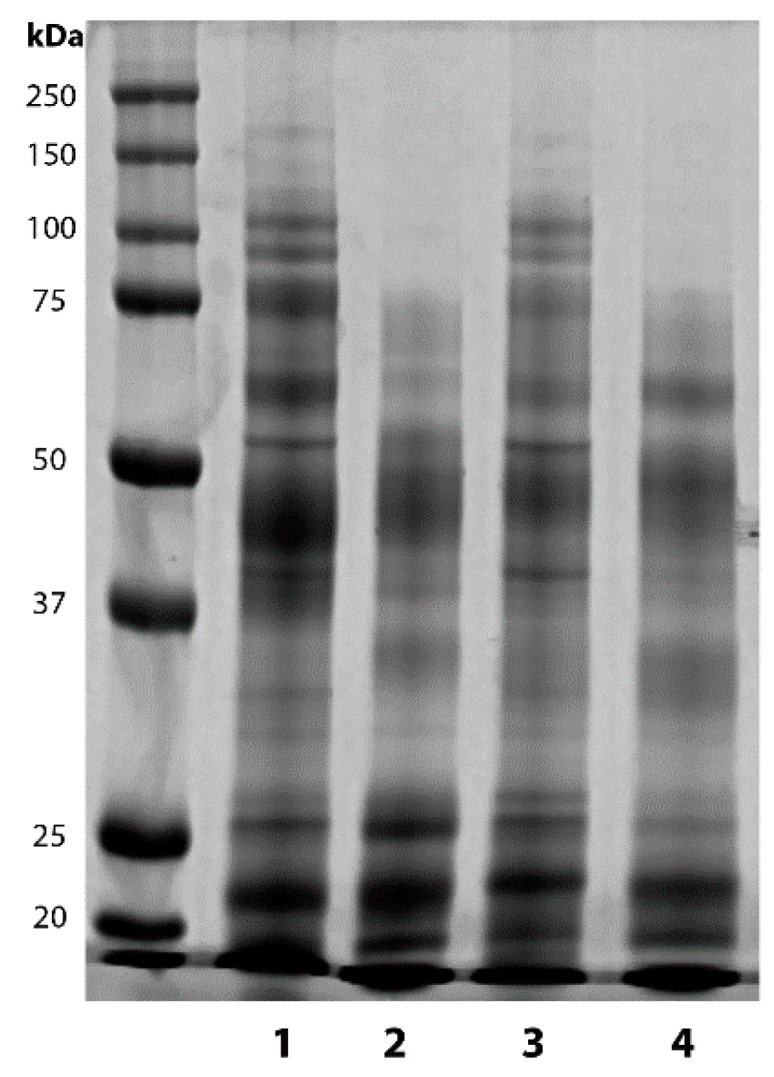
Representative image of SDS-PAGE of seminal plasma proteins from intact and vasectomized rams subjected to temperate (seasonal rams) or equatorial (non-seasonal rams) photoperiod. 1: intact seasonal, 2: vasectomized seasonal, 3: intact non-seasonal, 4: vasectomized non-seasonal.

Protein concentration was higher (*p* < 0.001) in SP from vasectomized than from the intact rams, (Table 1). SDS-PAGE, followed by band quantification, showed that vasectomy decreased high molecular weight bands (*p* < 0.001), with no differences in medium or low molecular bands in either the seasonal or equatorial rams (Table 1 and Figure 1). The photoperiodic regime did not affect protein concentration or band quantification.

**Figure 2:**
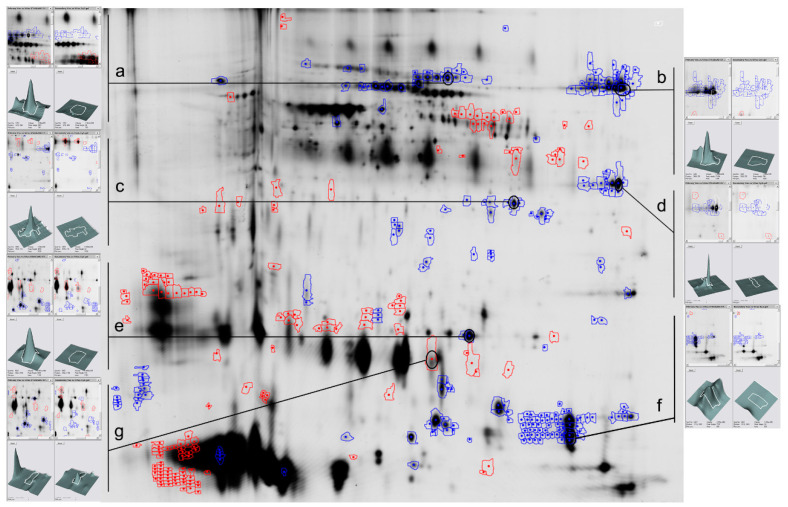
Representative image of difference gel electrophoresis (DIGE) analysis of seminal plasma proteins from intact and vasectomized rams. Angiotensin-converting enzyme (**a**), lactotransferrin (**b**), sorbitol dehydrogenase (**c**), phosphoglycerate kinase (**d**), epididymal secretory glutathione peroxidase (**e**), epididymal secretory protein E1 (**f**) and inactive ribonuclease-like protein 9 (**g**) spots are identified.

Due to the lack of significant differences between seasonal and equatorial rams, we only analyzed differences in the seminal plasma protein composition by difference gel electrophoresis (DIGE) in the seasonal males. DIGE revealed that the abundance of 40 spots increased (0.6%) and of 144 spots decreased (2.0%) in vasectomized animals (*p* < 0.01). DIGE also detected six proteins that were only present in intact rams (−6.0 Log volume ratio, Supplementary Figure S1). These proteins were identified (Supplementary File S2) as angiotensin-converting enzyme (ACE, Figure 2a), lactotransferrin (Figure 2b), sorbitol dehydrogenase (Figure 2c), phosphoglycerate kinase (Figure 2d), epididymal secretory glutathione peroxidase (Figure 2e) and epididymal secretory protein E1 (Figure 2f). Among the spots that increased in the vasectomized rams, we identified the inactive ribonuclease-like protein 9 (RNase9, Figure 2g) as the most abundant.

### 2.2. Vasectomy Decreases Melatonin and Testosterone Concentrations, but Not Antioxidant Enzyme Activity in Ram Seminal Plasma

**Figure 3:**
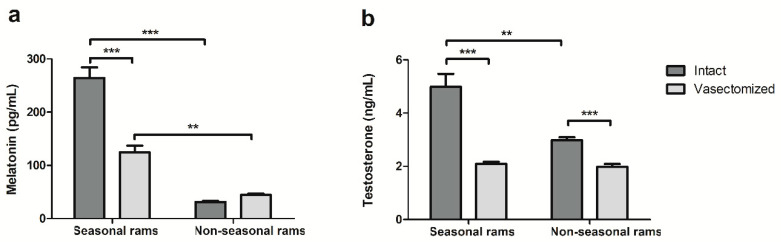
(**a**) Melatonin and (**b**) testosterone concentration in seminal plasma of intact and vasectomized rams subjected to temperate (seasonal rams) or equatorial (non-seasonal rams) photoperiod. Results are shown as mean ± S.E.M of *n* = 12 seminal plasma samples for seasonal rams, and *n* = 24 seminal plasma samples for non-seasonal. ** *p* < 0.01, *** *p* < 0.001.

Melatonin analyses revealed that the vasectomized rams in temperate climate had lower SP melatonin concentrations than the intact rams (264.3 ± 19.9 vs. 124.3 ± 12.3 pg/mL for intact and vasectomized rams, respectively, *p* < 0.001). This vasectomy effect was not found in males located in the equatorial photoperiod. Moreover, the melatonin concentration was significantly higher in seasonal than in non-seasonal rams, regardless of the state of their reproductive tract (Figure 3a).

On the other hand, vasectomy reduced the testosterone concentration in the seminal plasma of both seasonal and non-seasonal vasectomized rams when compared with their intact counterparts (Figure 3b, *p* < 0.001) This hormone was higher in the intact animals located in a temperate climate than in those in an equatorial photoperiod; however, this difference was not found in the vasectomized rams (Figure 3b)

**Figure 4:**
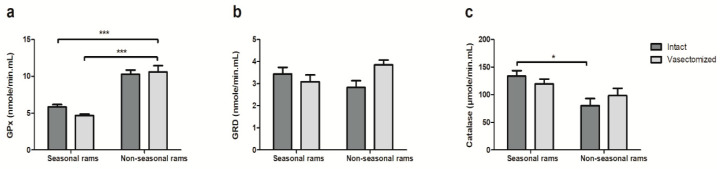
(**a**) Glutathione peroxidase (GPx), (**b**) glutathione reductase (GRD) and (**c**) catalase enzymatic activity in seminal plasma of intact and vasectomized rams subjected to temperate (seasonal) or equatorial (non-seasonal rams) photoperiod. Results are shown as mean ± S.E.M of *n* = 12 seminal plasma samples for seasonal rams, and *n* = 24 seminal plasma samples for non-seasonal. * *p* < 0.05, *** *p* < 0.001.

The photoperiod but not vasectomy affected the antioxidant enzyme activity, but vasectomy did not. Both the intact and vasectomized rams located in an equatorial photoperiod showed a higher GPx activity than the seasonal ones (Figure 4a, *p* < 0.001). In contrast, the intact rams located in a temperate climate had more catalase enzymatic activity than their equatorial counterparts (Figure 4c, *p* < 0.05). No differences were found in GRD activity.

## 3. Discussion

Previous studies revealed no differences in seminal plasma protein concentration between Colombian Creole and Romney Marsh rams [36]. In this work, we have also found that the photoperiodic regime (temperate vs. equatorial) does not affect protein concentration or the SDS-PAGE band profile.

However, vasectomy increased the protein concentration in seminal plasma, irrespective of the location of the rams. Nonetheless, this variation was not uniform, and a decrease in high molecular weight proteins was detected. These results were in concordance with those of Ghaoui et al. [15,16] and further support the hypothesis that most of the protein constituents of seminal plasma are derived from the accessory sex glands, whereas the high molecular weight proteins would have a testicular and epididymal origin in the form of membrane vesicles [15]. 

DIGE analyses allowed us to delve into these differences, and revealed an increase in the abundance of 40 spots and a decrease of 144 in seminal plasma from vasectomized rams, along with the absence of six proteins, subsequently identified as angiotensin-converting enzyme (ACE), lactotransferrin, phosphoglycerate kinase, sorbitol dehydrogenase, epididymal secretory glutathione peroxidase and epididymal secretory protein E1. Lactotransferrin [37], epididymal secretory glutathione peroxidase and epididymal secretory protein E1 [38] are secreted in the epididymis, and phosphoglycerate kinase in the testis [39]. In contrast, ACE and sorbitol dehydrogenase had been postulated to be of sperm origin [40] and released in the caput epididymis [1], although in human seminal plasma ACE could also be secreted by the prostate [41].

These proteins have multiple effects on spermatozoa: epididymal secretory protein E1 binds to the sperm surface, especially in the acrosome and midpiece [42], and it is possibly related to sperm motility; phosphoglycerate kinase is also associated with sperm motility in several species [43,44]; sorbitol dehydrogenase, which has been linked with cryopreservation resistance [45] and also with sperm motility during the epididymal transit [46]; ACE, which binds to the spermatozoa and is crucial for sperm-egg binding [47]. Finally, epididymal secretory glutathione peroxidase has antioxidant activity, and lactotransferrin is an iron carrier that prevents sperm lipid peroxidation [48]. These proteins could be useful as markers of fertility: in human semen, an increase in lactotransferrin levels has been related to a decrease in leukocyte levels [49], whereas epididymal secretory protein E1 was increased in asthenozoospermic samples [50].

Surprisingly, the protein with the biggest increase in the seminal plasma of vasectomized rams was inactive ribonuclease-like protein 9 (RNase9). RNase9, which has antibacterial activity [51] and was previously identified in the epithelium of the epididymis in human [52], mouse [53] and rat [54], and also in the postacrosomal region of human spermatozoa [52]. However, its abundance in the seminal plasma of the vasectomized rams suggests that in this species it would not be secreted by the epididymis, but in one of the accessory glands, possibly the seminal vesicles [55].

On the other hand, vasectomy reduced, but did not suppress, the melatonin and testosterone concentrations in the seminal plasma, which suggests that a portion of the seminal plasma hormonal content has a testicular origin.

The decrease in the melatonin concentration caused by vasectomy was only detected in seasonal rams. In rams located in temperate climates, the melatonin concentration in seminal plasma undergoes seasonal variation [23]. Additionally, the melatonin-synthesizing enzymes aralkylamine N-acetyltransferase (AANAT) and N-Acetylserotonin O-methyltransferase (ASMT) are present in ram testes [26]. In this work, we have demonstrated that around half of the melatonin found in the seminal plasma of seasonal rams during the reproductive season has a testicular origin, and the pineal gland might be the source of the other half. However, the lack of differences in the melatonin concentration between intact and vasectomized rams located in a constant photoperiod suggests that, in these males, either the testicular melatonin synthesis is eliminated, or the testicular melatonin is not secreted to the seminal plasma. This could be due to the different photoperiodic environment the rams are exposed to. In seasonal rams, melatonin receptors MT1 and MT2 are also present in the testes [56], thus variations in pineal melatonin could regulate testicular melatonin secretion, as in other extrapineal melatonin-synthesizing organs or cells [57]. In contrast, equatorial rams are subjected to a constant 12L:12D light regimen and the lack of seasonal variations in pineal melatonin could be reflected in testicular melatonin secretion. However, more studies on the presence and functionality of melatonin-synthesizing enzymes and melatonin receptors in the testes of equatorial rams are needed to test this hypothesis. Apart from the lack of differences between intact and vasectomized rams, non-seasonal males had a significantly lower concentration of seminal plasma melatonin than their seasonal counterparts. We had previously detected these low melatonin levels in a previous study [35]. This difference in melatonin concentration could be due to either the shorter night length (12 h 10′ vs. 16 h 12′ of darkness for equatorial and Mediterranean rams at the end of the experiment) or the static photoperiodic signal caused by a 12L:12D light regimen [58].

Vasectomy also decreased the testosterone concentration in the seminal plasma of seasonal and non-seasonal rams, although this change was more marked in the Mediterranean males. This seminal plasma testosterone decrease has been previously identified in human [59] and boar [60]. Our results also suggest a dual origin of the testosterone present in the ram seminal plasma, with testicular (from rete testes fluid [61]) and non-testicular (likely from circulating blood or, to a lesser extent, from adrenal glands [62]) contribution. We also detected differences in testosterone concentration between intact rams, but not between the vasectomized ones from different light regimens, which suggests a possible photoperiodic regulation of the local testosterone secretion by the testis. In the seasonal ram, testosterone secretion is regulated by the photoperiod [63,64], thus, it is possible that, as previously discussed for melatonin concentration, the differences in the dark/light regimen between the rams would explain the differences in the testis contribution to seminal plasma testosterone found in this work.

Finally, vasectomy did not affect antioxidant enzymatic activity. Previous studies in humans revealed that vasectomy did not affect catalase and superoxide dismutase activities in human seminal plasma [65], and that most antioxidant enzymes present in this fluid had a prostatic origin [22]. Thus, an accessory gland source would explain the presence of GPx in vasectomized rams although DIGE analyses revealed the absence of epididymal secretory glutathione peroxidase in these animals.

GPx was significantly higher in equatorial than in Mediterranean rams, whereas catalase activity was higher in seasonal males. We had previously detected this increase in GPx activity during the rainy season [35] in tropical climates, which would likely protect the spermatozoa from oxidative damage in high humidity or temperature conditions. We had also previously detected higher levels of catalase activity, although not significant, during the autumn months in seasonal rams, and a positive correlation with seminal plasma melatonin [23], which could explain the results found in this work. 

## 4. Materials and Methods

### 4.1. Animals and Seminal Plasma Extraction

#### 4.1.1. Rams in the Mediterranean Climate

Seminal plasma was obtained weekly for three months during the reproductive season (September to November) from first ejaculates of five Rasa Aragonesa rams (three intact and two vasectomized) in compliance with the requirements of the Directive 2010/63/EU of the European Parliament on the protection of animals used for scientific purposes. All experimental procedures were performed under Project License PI19/17 approved by the Ethics Committee for Animal Experiments of the University of Zaragoza (approval date: 24 May 2017). The males were maintained under uniform nutritional conditions and natural photoperiod at the Experimental Farm of the University of Zaragoza, Spain (41°38′05.8″ N 0°51′35.2″ W). Local amplitude of the photophase during the period of study (September to November) varies from 12 h 35′ (11 h 25′ of darkness) at the beginning of the experiment to 7 h 48′ (16 h 12′ of darkness) at the end, i.e., 4 h 47′ of difference between the longest and the shortest day of this period. 

Ejaculates from intact and vasectomized animals were collected once a week using an artificial vagina. In order to eliminate individual differences and obtain enough volume of sample for all the analyses, daily ejaculates from the rams of each experimental group were pooled and processed together [66]. Seminal plasma was extracted by centrifugation at 7500× *g* for 10 min in a microfuge at 4 °C (Eppendorf Centrifuge 5430R, Hamburg, Germany). The supernatant was centrifuged again; the seminal plasma was then recovered and filtered through a 0.22 µm Millipore membrane (Merck KGaA, Darmstadt, Germany). After adding 10% protease and phosphatase inhibitor (Sigma Chemical Co., St. Louis, MO, USA), SP was aliquoted and kept at −20 °C until analysis.

#### 4.1.2. Rams under Equatorial Photoperiod

All procedures used in this study were in strict accordance with Colombian Animal Protection Regulations (Law 84/1989, modified by Law 1774/2016) and were approved by the Bioethics Committee of the Faculty of Veterinary Medicine and Zootechnics, Bogotá Headquarters, National University of Colombia (Project license: CB-074-2014, approval date: 5 November 2014). The rams were located at the Center for Ovine Research, Technological Development and Extension (CIDTEO), of the National University of Colombia, Mosquera (4°″ 40′57″ N 74°″ 12′50″ W). Local amplitude of the photophase during the period of study (September to November, rainy season) varies from 12 h 09′ (11 h 51′ of darkness) to 11 h 50′ (12 h 10′ of darkness), i.e., 19′ of difference between the longest and the shortest day.

Semen was obtained using an artificial vagina from 10 mature rams of two breeds (Creole and Romney Marsh, eight intact and two vasectomized). Ejaculates were collected once a week, and daily samples from each breed and experimental group were pooled together for seminal plasma extraction.

Seminal plasma was obtained following the same protocol as in the rams in the Mediterranean Climate, and sent to Spain, where the SP was analyzed, in dry ice.

### 4.2. Seminal Plasma Protein Analyses

The seminal plasma protein concentration was calculated by Bradford’s method [67] using a commercial kit (Quick Start Bradford protein assay, Bio-Rad, Hercules, CA, USA), whereas the protein composition was analyzed by SDS-PAGE and difference gel electrophoresis (DIGE).

For SDS-PAGE, 20 μg of SP proteins were mixed with a loading buffer (Tris/HCl 0.045 M, EDTA 0.8 mM, SDS 3% (*wt*/*v*), glycerol 10% (*v*/*v*), β-mercaptoethanol 5% (*v*/*v*) and bromophenol blue 0.004% (*wt*/*v*)) and loaded in a 10% polyacrylamide gel. The electrophoresis was performed at 130 V for 90 min at 4 °C. A mixture of prestained molecular weights ranging from 10 to 250 kDa (Bio-Rad, Hercules, CA, USA) was used as a standard. After electrophoresis, the gels were stained with Coomassie Brilliant Blue (0.1% *wt*/*v*) in 45% (*v*/*v*) methanol and 10% (*v*/*v*) acetic acid, and de-stained in 30% (*v*/*v*) methanol, 10% (*v*/*v*) acetic acid and distilled water until no background was detectable. Gel images were captured and analyzed with the Odyssey Clx Infrared Imaging System (Li-Cor Biosciences, Lincoln, NE, USA).

Finally, the proteomic analysis by difference gel electrophoresis (DIGE) was performed in the Proteomics Unit of the Complutense University of Madrid, Spain, and analyzed using the DeCyder 2-D Differential Analysis software (version 5.0, GE Healthcare, Chicago, IL, USA). Differentially expressed spots were excised, digested and identified by MALDI-TOF MS fingerprinting and the MASCOT algorithm v2.1 (Matrix Science, Boston, MA, USA).

### 4.3. Seminal Plasma Hormonal Analyses

#### 4.3.1. Melatonin Evaluation

Seminal plasma melatonin concentration was measured using a competitive commercial immunoassay (Direct saliva melatonin ELISA kit, Bühlmann Laboratories AG, Schönenbuch, Switzerland). Assay specifications were: Sensitivity: 0.5 pg/mL, Intra-assay variability: 5.2%, Inter-assay variability: 11.2%. Following the manufacturer’s instructions, 100 μL of seminal plasma sample, control, or calibrator were loaded in duplicate in a microtiter plate coated with an anti-melatonin antibody. After 16 to 20 h incubation at 2 to 8 °C, 50 μL of biotinylated melatonin were added to each well. Following another 3 h of incubation at 2 to 8 °C and three washes, 100 μL of streptavidin-conjugated horseradish peroxidase (HRP) was added into the wells. The plate was then incubated for 60 min at 600 rpm at room temperature in a plate rotator. After the other three were washed, 100 μL of tetramethylbenzidine substrate (TMB) was added and the plate was incubated for 30 min at the same conditions but protected from direct light. Finally, 100 μL of 0.25 M SO_4_H_2_ solution was added, and absorbance was measured at 450 nm on a plate reader (TECAN Spectrafluor plus, Tecan Group Ltd., Männedorf, Switzerland).

#### 4.3.2. Testosterone Evaluation

Seminal plasma total testosterone concentration in the ram seminal plasma was evaluated by means of a commercial ELISA kit assay (Testo-Easia, BioSource Europe, SA, Belgium; Sensitivity: 0.05 ng/mL; Intra-assay variability: 4.8%, Inter-assay variability: 7.1%). Following the manufacturer’s instructions, 50 μL of seminal plasma, control or calibrators was loaded in duplicate in an anti-testosterone coated microtiter plate. After the addition of 100 μL of HRP-labelled testosterone, the plate was incubated for one hour at room temperature. At the end of the incubation, the wells were washed three times, 100 μL of TMB were added to each well and the plate was incubated for 30 min at room temperature, protected from direct light. Finally, 100 μL of 0.2 M HCl solution was added, and absorbance was measured on a plate reader (TECAN Spectrafluor plus, Tecan Group Ltd., Männedorf, Switzerland) at 450 nm.

### 4.4. Seminal Plasma Antioxidant Enzymes Activity

In these assays, all samples were loaded in duplicate and analyzed the same day.

#### 4.4.1. Glutathione Peroxidase (GPx)

Glutathione Peroxidase enzymatic activity was evaluated in 6 µL of seminal plasma, measuring the oxidation of glutathione (GSH, 2 mM) to oxidized glutathione (GSSG) catalyzed by GPx. Ter-Butylhydroperoxide (t-BuO2H, 1.2 mM) was used as an electron acceptor, and GSSG was recycled back to GSH using GRD (54 mUI) and NADPH (85 µM), in a 300 mM sodium phosphate buffer (pH 7.2) that also contained EDTA 0.5 mM. Final volume was 200 µL. The enzymatic activity was monitored for 3 min at 340 nm in a microtiter plate reader (TECAN Spectrafluor plus, Tecan Group Ltd., Männedorf, Switzerland).

#### 4.4.2. Glutathione Reductase (GRD)

Glutathione Reductase enzymatic activity was evaluated measuring the decrease in absorbance produced by NADPH oxidation because of the oxidized glutathione (GSSG) reduction. The reaction mixture contained 300 mM sodium phosphate buffer at pH 7.2; 0.5 mM EDTA; 85 µM NADPH; 0.8 mM GSSG. Five µL of seminal plasma were added to complete a final volume of 200 µL. The enzymatic activity was evaluated for 3 min at 340 nm with a microtiter plate reader (TECAN Spectrafluor plus, Tecan Group Ltd., Männedorf, Switzerland).

#### 4.4.3. Catalase

Catalase enzymatic activity was evaluated by the decrease in absorbance due to the H_2_O_2_ reduction produced by this enzyme. The reaction mixture contained 50 mM sodium phosphate buffer (pH 7), 30 mM H_2_O_2_ and 4 µL seminal plasma (final volume of 200 µL). The change in absorbance was measured for 2 min at 240 nm in a plate reader (TECAN Spectrafluor plus, Tecan Group Ltd., Männedorf, Switzerland).

### 4.5. Statistical Analyses

The number of evaluated samples was 12 from each breed (Rasa aragonesa, Creole or Romney Marsh) and experimental group (intact or vasectomized). The distribution of the data and homoscedasticity were evaluated by the Kolmogorov-Smirnov and Bartlett’s tests, respectively. When data showed a normal distribution and equal variances (i.e., total protein, band intensity and GRD), differences between experimental groups were compared by means of ANOVA, followed by Tukey’s Multiple Comparison Test. When the studied data failed the normality or homoscedasticity test (i.e., hormonal data, GPx and catalase), differences between groups were analyzed by means of the Kruskal-Wallis test, followed by Dunn’s post-test. All statistical analysis was performed with GraphPad Prism version 5.01 for Windows (GraphPad Software, La Jolla, CA, USA).

## 5. Conclusions

In conclusion, vasectomy modifies the protein profile and hormonal content of ram seminal plasma, but not antioxidant enzyme activity. The exposure to a constant photoperiod resulted in decreased melatonin and testosterone concentrations in this fluid, and increased GPx activity, with no effect on the protein profile.

## Figures and Tables

**Table 1:** Protein concentration (mg/mL, *n* = 12 seminal plasma samples for seasonal rams, and *n* = 24 seminal plasma samples for non-seasonal) and densitometric quantification (×10^3^ arbitrary units, *n* = 4 seminal plasma samples) of high, medium and low molecular weight (MW) bands of seminal plasma from intact and vasectomized rams subjected to a temperate (seasonal rams) or equatorial (non-seasonal rams) photoperiod. Results are shown as mean ± SEM. ^a^, ^b^ indicate *p* < 0.001.

		Band Intensity × 10^3^ (Arbitrary Units)		
	Total Protein (mg/mL)	High MW Bands (250–75 kDa)	Medium MW Bands (75–37 kDa)	Low MW Bands (37–10 kDa)
Intact seasonal rams	34.20 ± 5.42 ^a^	3.90 ± 1.43 ^a^	11.25 ± 3.54	9.95 ± 2.80
Vasectomized seasonal rams	64.63 ± 6.36 ^b^	0.44 ± 0.08 ^b^	7.57 ± 1.0	11.80 ± 1.46
Intact non-seasonal rams	39.60 ± 1.66 ^a^	3.54 ± 0.53 ^a^	9.45 ± 1.21	12.20 ± 1.47
Vasectomized non-seasonal rams	53.40 ± 2.10 ^b^	0.60 ± 0.13 ^b^	10.56 ± 5.29	10.85 ± 3.44

## Supplementary Materials

Supplementary materials can be found at https://www.mdpi.com/1422-0067/21/21/8063/s1.

## Abbreviations

SP	Seminal plasma
GPx	Glutathione peroxidase
GRD	Glutathione reductase
SOD	Superoxide dismutase
DIGE	Difference gel electrophoresis

## Appendix A

**Table A1:** Protein, melatonin and testosterone concentration, and antioxidant enzymes activity in seminal plasma of intact and vasectomized rams of two breeds (Creole and Rommey Marsh) located in equatorial photoperiod. Results are shown as mean ± SEM of *n* = 12 seminal plasma samples.

	Intact		Vasectomized	
	Creole	Romney Marsh	Creole	Romney Marsh
Total protein (mg/mL)	36.51 ± 2.30	42.69 ± 2.18	52.05 ± 2.98	55.01 ± 10.12
Melatonin (pg/mL)	32.78 ± 3.38	30.15 ± 1.50	46.63 ± 3.05	42.51 ± 4.52
Testosterone (ng/mL)	3.06 ± 0.14	2.89 ± 0.18	1.92 ± 0.13	2.05 ± 0.14
Glutathione peroxidase (nmole/min mL)	10.44 ± 0.82	10.15 ± 0.79	12.68 ± 1.43	9.58 ± 0.71
Glutathione reductase (nmole/min mL)	2.41 ± 0.46	2.73 ± 0.42	4.50 ± 0.43	3.60 ± 0.29
Catalase (µmole/min mL)	68.84 ± 16.00	91.21 ± 19.50	104.04 ± 20.06	93.75 ± 17.71
