# Supplementary material for: Vasectomy and Photoperiodic Regimen Modify the Protein Profile, Hormonal Content and Antioxidant Enzymes Activity of Ram Seminal Plasma
Source: Int J Mol Sci. 2020 Oct 29;21(21):8063. doi: 10.3390/ijms21218063 (PMC7663742; doi:10.3390/ijms21218063)
Supplement: Supplementary file 1 [file ijms-21-08063-s001.zip › Supplementary figure 1.pdf]

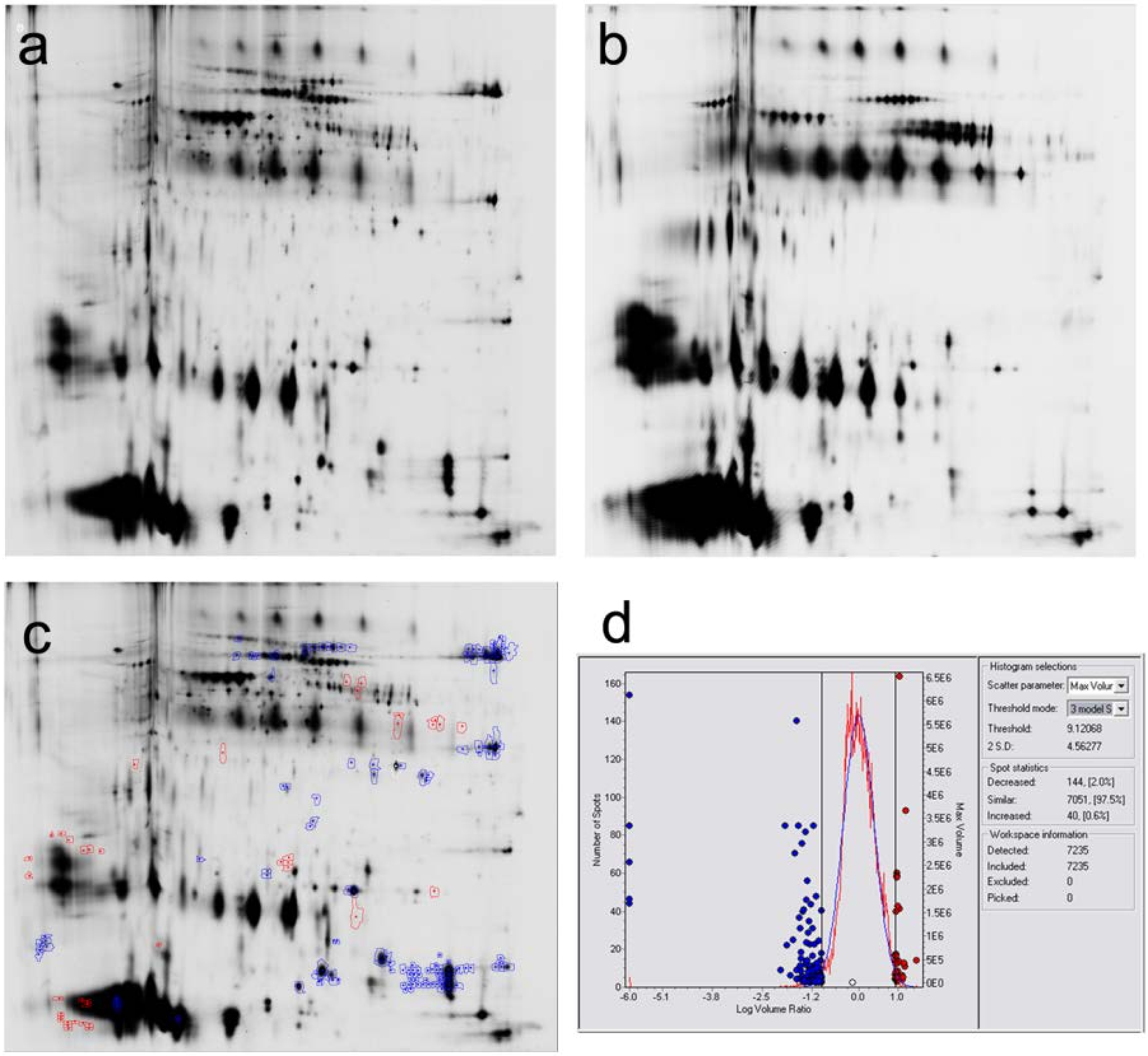

Supplemental figure 1. Representative image of difference gel electrophoresis (DIGE) analysis of seminal plasma proteins from intact (panel **a**) and vasectomized (panel **b**)rams. Blue spots in panel **c** show the significantly increased (P<0.01) proteins from intact rams, whereas red spots indicate significant increased proteins from vasectomized ones, identified on intact rams gel. DeCyder software spot analysis and differential protein expression is also shown (panel **d**)
