# Supplementary material for: Vasectomy and Photoperiodic Regimen Modify the Protein Profile, Hormonal Content and Antioxidant Enzymes Activity of Ram Seminal Plasma
Source: Int J Mol Sci. 2020 Oct 29;21(21):8063. doi: 10.3390/ijms21218063 (PMC7663742; doi:10.3390/ijms21218063)
Supplement: Supplementary file 1 [file ijms-21-08063-s001.zip › Supplementary file 2_Mascot protein identification/ACE identification.pdf]

Mascot Search Results

Protein View

Match to: **ACE_MOUSE** Score: **92** Expect: **4e-005**
Angiotensin-converting enzyme OS=Mus musculus GN=Ace PE=1 SV=3

Nominal mass (M_r): **151678**; Calculated pI value: **6.10**
NCBI BLAST search of ACE_MOUSE against nr
Unformatted sequence string for pasting into other applications

Taxonomy: Mus musculus

Fixed modifications: Carbamidomethyl (C)
Variable modifications: Oxidation (M)
Cleavage by Trypsin: cuts C-term side of KR unless next residue is P
Sequence Coverage: **5%**

Matched peptides shown in **Bold Red**

1 MGAASGQRGR WPLSPPLLML SLLVLLLQPS PAPALDPGLQ PGNFSPDEAG
51 AQLFAESYNS SAEVVMFQST VASWAHDTNI TEENARRQEE AALVSQEFAE
101 VWGKKAKELY ESIWQNFTDS KLRRIIGSIR TLGPANLPLA QRQQYNSLLS
151 NMSRIYSTGK VCFPNKTATC WSLDPELTNI LASSRSYAKL LFAWEGWHDA
201 VGIPLKPLYQ DFTAISNEAY RQDDFSDTGA FWRSWYESPS FEESLEHIYH
251 QLEPLYLNLH AYVRRALHRR YGDKYVNLRG PIPAHLLGDM WAQSWENIYD
301 MVVPFPDKPN LDVTSTMVQK GWNATHMFRV SEEFFTSLGL SPMPPEFWAE
351 SMLEKPTDGR EVVCHASAWD FYNRKDFRIK QCTRVTMEQL ATVHHEMGHV
401 QYYLQYK**DLH** **VSLRR**GANPG FHEAIGDVLA LSVSTPAHLH KIGLLDHVTN
451 DIESDINYLL KMALEKIAFL PFGYLVDQWR WGVFSGRTPP SRYNFDWWYL
501 RTK**YQGICPP** **VAR**NETHFDA GAKFHIPNVT PYIRYFVSFV LQFQFHQALC
551 KEAGHQGPLH QCDIYQSAQA GAKLKQVLQA GCSRPWQEVL K**DLVGSDALD**
601 **AK**ALLEYFQP VSQWLEEQNQ RNGEVLGWPE NQWRPPLPDN YPEGIDLETD
651 EAKADRFVEE YDRTAQVLLN EYAEANWQYN TNITIEGSKI LLEKSTEVSN
701 HTLKYGTRAK **TFDVSNFQNS** **SIKR**IIKKLQ NLDRAVLPPK ELEEYNQILL
751 DMETTYSLSN ICYTNGTCMP LEPDLTNMMA TSRKYEELLW AWKSWRDKVG
801 RAILPFFPKY VEFSNKIAKL NGYTDAGDSW RSLYESDNLE QDLEK**LYQEL**
851 **QPLYLNLHAY** **VRR**SLHRHYG SEYINLDGPI PAHLLGNMWA QTWSNIYDLV
901 APFPSAPNID ATEAMIKQGW TPRRIFKEAD NFFTSLGLLP VPPEFWNKSM
951 LEKPTDGREV VCHPSAWDFY NGKDFRIKQC TSVNMEDLVI AHHEMGHIQY
1001 FMQYKDLPVT FREGANPGFH EAIGDIMALS VSTPKHLYSL NLLSTEGSGY
1051 EYDINFLMKM ALDKIAFIPF SYLIDQWRWR VFDGSITK**EN** **YNQEWWSLR**L
1101 KYQGLCPPVP RSQGDFDPGS KFHVPANVPY VRYFVSFIIQ FQFHEALCRA
1151 AGHTGPLHKC DIYQSKEAGK LLADAMKLGY SKPWPEAMKL ITGQPNMSAS
1201 AMMNYFKPLT EWLVTENRRH GETLGWPEYN WAPNTARAEG STAESNRVNF
1251 LGLYLEPQQA RVGQWVLLFL GVALLVATVG LAHRLYNIRN HHSLRRPHRG
1301 PQFGSEVELR HS

Show predicted peptides also

Sort Peptides By ◉ Residue Number ○ Increasing Mass ○ Decreasing Mass

Start - End	Observed	Mr(expt)	Mr(calc)	ppm	Miss	Sequence
408 - 415	995.4992	994.4919	994.5672	-76	1	K.DLHVSLRR.G (No match)
504 - 513	1160.5824	1159.5751	1159.5808	-5	0	K.YQGICPPVAR.N (No match)
592 - 602	1103.5219	1102.5146	1102.5506	-33	0	K.DLVGSDALDAK.A (No match)
711 - 724	1642.7389	1641.7316	1641.8111	-48	1	K.TFDVSNFQNSSIKR.I (No match)
846 - 863	2289.1655	2288.1582	2288.2429	-37	1	K.LYQELQPLYLNLHAYVRR.S (No match)
1089 - 1099	1524.6910	1523.6837	1523.6793	3	0	K.ENYNQEWWSLR.L (No match)
1089 - 1099	1524.6910	1523.6837	1523.6793	3	0	K.ENYNQEWWSLR.L (Ions score 82)

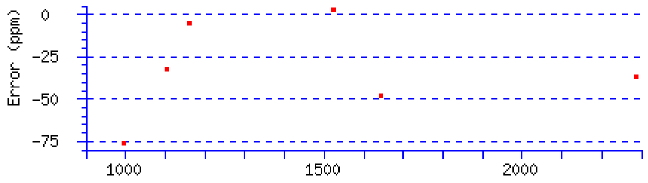

RMS error 38 ppm

Mascot Search Results

User	:
Email	:
Search title	: SampleSetID: 611, AnalysisID: 4512, MaldiWellID: 55963, SpectrumID: 109840, Path=\160212\MSMS\16-13 Jose Alvaro
Database	: SwissProt sprot_160208 (550116 sequences; 196219159 residues)
Taxonomy	: Mammalia (mammals) (66429 sequences)
Timestamp	: 12 Feb 2016 at 11:36:06 GMT
Warning	: A Peptide summary report will usually give a much clearer picture of MS/MS search results.
Top Score	: 92 for ACE_MOUSE, Angiotensin-converting enzyme OS=Mus musculus GN=Ace PE=1 SV=3

Mascot Score Histogram

Protein score is -10*Log(P), where P is the probability that the observed match is a random event.
Protein scores greater than 61 are significant (p<0.05).
Protein scores are derived from ions scores as a non-probabilistic basis for ranking protein hits.

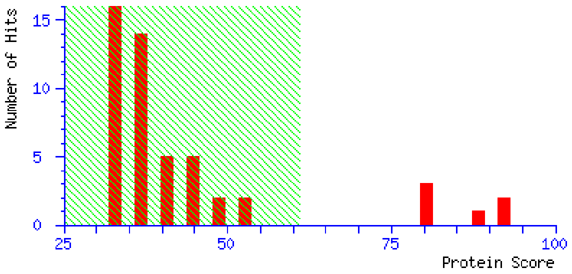

Protein Summary Report

Format As | Protein Summary (deprecated) | Help

Significance threshold p< 0.05 Max. number of hits 20

Re-Search All | Search Unmatched

Index

	Accession	Mass	Score	Description
1.	ACE_MOUSE	151678	92	Angiotensin-converting enzyme OS=Mus musculus GN=Ace PE=1 SV=3
2.	ACE_MESAU	152297	91	Angiotensin-converting enzyme OS=Mesocricetus auratus GN=Ace PE=2 SV=1
3.	ACE_RAT	151668	89	Angiotensin-converting enzyme OS=Rattus norvegicus GN=Ace PE=1 SV=1
4.	ACE_HUMAN	150418	82	Angiotensin-converting enzyme OS=Homo sapiens GN=ACE PE=1 SV=1
5.	ACE_PANTR	150074	82	Angiotensin-converting enzyme OS=Pan troglodytes GN=ACE PE=3 SV=1
6.	ACE_RABIT	151165	82	Angiotensin-converting enzyme OS=Oryctolagus cuniculus GN=ACE PE=1 SV=3
7.	MYOF_HUMAN	236100	54	Myoferlin OS=Homo sapiens GN=MYOF PE=1 SV=1
8.	CJ120_BOVIN	39450	51	Uncharacterized protein C10orf120 homolog OS=Bos taurus PE=2 SV=1
9.	MIA3_HUMAN	214255	50	Melanoma inhibitory activity protein 3 OS=Homo sapiens GN=MIA3 PE=1 SV=1
10.	K2C8_HUMAN	53671	47	Keratin, type II cytoskeletal 8 OS=Homo sapiens GN=KRT8 PE=1 SV=7
11.	GOGB1_HUMAN	377215	47	Golgin subfamily B member 1 OS=Homo sapiens GN=GOLGB1 PE=1 SV=2
12.	ILKAP_HUMAN	43450	45	Integrin-linked kinase-associated serine/threonine phosphatase 2C OS=Homo sapiens GN=ILKAP PE=1 SV=1
13.	K2C8_RAT	53985	44	Keratin, type II cytoskeletal 8 OS=Rattus norvegicus GN=Krt8 PE=1 SV=3
14.	UACA_CANLF	163865	44	Uveal autoantigen with coiled-coil domains and ankyrin repeats OS=Canis lupus familiaris GN=UACA PE=2 SV=2
15.	SYDC_HUMAN	57499	43	Aspartate--tRNA ligase, cytoplasmic OS=Homo sapiens GN=DARS PE=1 SV=2
16.	IFT81_MOUSE	79692	42	Intraflagellar transport protein 81 homolog OS=Mus musculus GN=Ift81 PE=1 SV=4
17.	WDR87_HUMAN	335256	40	WD repeat-containing protein 87 OS=Homo sapiens GN=WDR87 PE=1 SV=3
18.	TSG10_RAT	83644	40	Testis-specific gene 10 protein OS=Rattus norvegicus GN=Tsga10 PE=1 SV=2
19.	SERC_MOUSE	40732	40	Phosphoserine aminotransferase OS=Mus musculus GN=Psat1 PE=1 SV=1
20.	SPTN1_MOUSE	285221	40	Spectrin alpha chain, non-erythrocytic 1 OS=Mus musculus GN=Sptan1 PE=1 SV=4

Results List

1. ACE_MOUSE **Mass:** 151678 **Score:** 92 **Expect:** 4e-005 **Matches:** 7
Angiotensin-converting enzyme OS=Mus musculus GN=Ace PE=1 SV=3

Observed	Mr(expt)	Mr(calc)	ppm	Start	End	Miss	Ions	Peptide
995.4992	994.4919	994.5672	-75.69	408 -	415	1	---	K.DLHVSLRR.G
1103.5219	1102.5146	1102.5506	-32.64	592 -	602	0	---	K.DLVGSDALDAK.A
1160.5824	1159.5751	1159.5808	-4.90	504 -	513	0	---	K.YQGICPPVAR.N
1524.6910	1523.6837	1523.6793	2.90	1089 -	1099	0	---	K.ENYNQEWWSLR.L
1524.6910	1523.6837	1523.6793	2.90	1089 -	1099	0	82	K.ENYNQEWWSLR.L
1642.7389	1641.7316	1641.8111	-48.40	711 -	724	1	---	K.TFDVSNFQNSSIKR.I
2289.1655	2288.1582	2288.2429	-37.01	846 -	863	1	---	K.LYQELQPLYLNLHAYVRR.S

No match to: 855.0244, 857.4423, 861.0447, 877.0201, 885.9838, 892.9934, 906.4807, 914.5364, 964.5336, 1018.4599, 1020.4916, 1036.5293, 1050.0758, 1050.5299, 1066.0537, 1082.0249, 1082.5898, 1111.5421, 1113.5225, 1118.5022, 1136.5441, 1174.5968, 1201.6826, 1234.6770, 1241.6234, 1241.6234, 1255.6364, 1277.7050, 1285.7051, 1285.7051, 1294.6182, 1300.5901, 1303.5570, 1308.6539, 1320.5820, 1350.5736, 1367.5901, 1368.5878, 1383.6404, 1399.7301, 1401.7665, 1415.7883, 1416.7784, 1418.6888, 1431.7214, 1434.7179, 1449.7212, 1465.6866, 1475.7599, 1506.6792, 1523.7628, 1537.7434, 1638.8650, 1707.7817, 1738.9299, 1993.9830, 2185.0576, 2188.0432, 2199.0037, 2224.9702, 2335.0881

2. ACE_MESAU **Mass:** 152297 **Score:** 91 **Expect:** 5e-005 **Matches:** 8
Angiotensin-converting enzyme OS=Mesocricetus auratus GN=Ace PE=2 SV=1

Observed	Mr(expt)	Mr(calc)	ppm	Start	End	Miss	Ions	Peptide

906.4807	905.4734	905.4389	38.1	729 - 735	1	---	K.KVQNMDR.A + Oxidation (M)
1285.7051	1284.6978	1284.6098	68.5	658 - 667	1	---	R.FVEEYDRTAR.V
1285.7051	1284.6978	1284.6098	68.5	658 - 667	1	---	R.FVEEYDRTAR.V
1383.6404	1382.6331	1382.7340	-72.96	730 - 741	1	---	K.VQNMDRAVLPPK.E + Oxidation (M)
1415.7883	1414.7810	1414.7932	-8.63	699 - 711	1	---	K.VANHTLKYGTLAK.K
1475.7599	1474.7526	1474.7092	29.4	713 - 724	0	---	K.FDVSNFQNYTIK.R
1524.6910	1523.6837	1523.6793	2.90	1090 - 1100	0	---	K.ENYNQEWWSLR.L
1524.6910	1523.6837	1523.6793	2.90	1090 - 1100	0	82	K.ENYNQEWWSLR.L

No match to: 855.0244, 857.4423, 861.0447, 877.0201, 885.9838, 892.9934, 914.5364, 964.5336, 995.4992, 1018.4599, 1020.4916, 1036.5293, 1050.0758, 1050.5299, 1066.0537, 1082.0249, 1082.5898, 1103.5219, 1111.5421, 1113.5225, 1118.5022, 1136.5441, 1160.5824, 1174.5968, 1201.6826, 1234.6770, 1241.6234, 1241.6234, 1255.6364, 1277.7050, 1294.6182, 1300.5901, 1303.5570, 1308.6539, 1320.5820, 1350.5736, 1367.5901, 1368.5878, 1399.7301, 1401.7665, 1416.7784, 1418.6888, 1431.7214, 1434.7179, 1449.7212, 1465.6866, 1506.6792, 1523.7628, 1537.7434, 1638.8650, 1642.7389, 1707.7817, 1738.9299, 1993.9830, 2185.0576, 2188.0432, 2199.0037, 2224.9702, 2289.1655, 2335.0881

3. ACE_RAT **Mass:** 151668 **Score:** 89 **Expect:** 8e-005 **Matches:** 5
Angiotensin-converting enzyme OS=Rattus norvegicus GN=Ace PE=1 SV=1

Observed	Mr(expt)	Mr(calc)	ppm	Start - End	Miss	Ions	Peptide
995.4992	994.4919	994.5672	-75.69	409 - 416	1	---	K.DLHVSLRR.G
1160.5824	1159.5751	1159.5808	-4.90	505 - 514	0	---	K.YQGICPPVAR.N
1524.6910	1523.6837	1523.6793	2.90	1090 - 1100	0	---	K.ENYNQEWWSLR.L
1524.6910	1523.6837	1523.6793	2.90	1090 - 1100	0	82	K.ENYNQEWWSLR.L
2289.1655	2288.1582	2288.2429	-37.01	847 - 864	1	---	K.LYQELQPLYLNLHAYVRR.S

No match to: 855.0244, 857.4423, 861.0447, 877.0201, 885.9838, 892.9934, 906.4807, 914.5364, 964.5336, 1018.4599, 1020.4916, 1036.5293, 1050.0758, 1050.5299, 1066.0537, 1082.0249, 1082.5898, 1103.5219, 1111.5421, 1113.5225, 1118.5022, 1136.5441, 1174.5968, 1201.6826, 1234.6770, 1241.6234, 1241.6234, 1255.6364, 1277.7050, 1285.7051, 1285.7051, 1294.6182, 1300.5901, 1303.5570, 1308.6539, 1320.5820, 1350.5736, 1367.5901, 1368.5878, 1383.6404, 1399.7301, 1401.7665, 1415.7883, 1416.7784, 1418.6888, 1431.7214, 1434.7179, 1449.7212, 1465.6866, 1475.7599, 1506.6792, 1523.7628, 1537.7434, 1638.8650, 1642.7389, 1707.7817, 1738.9299, 1993.9830, 2185.0576, 2188.0432, 2199.0037, 2224.9702, 2335.0881

4. ACE_HUMAN **Mass:** 150418 **Score:** 82 **Expect:** 0.00039 **Matches:** 3
Angiotensin-converting enzyme OS=Homo sapiens GN=ACE PE=1 SV=1

Observed	Mr(expt)	Mr(calc)	ppm	Start - End	Miss	Ions	Peptide
1475.7599	1474.7526	1474.7303	15.1	730 - 742	0	---	R.AALPAQELEEYNK.I
1524.6910	1523.6837	1523.6793	2.90	1084 - 1094	0	---	K.ENYNQEWWSLR.L
1524.6910	1523.6837	1523.6793	2.90	1084 - 1094	0	82	K.ENYNQEWWSLR.L

No match to: 855.0244, 857.4423, 861.0447, 877.0201, 885.9838, 892.9934, 906.4807, 914.5364, 964.5336, 995.4992, 1018.4599, 1020.4916, 1036.5293, 1050.0758, 1050.5299, 1066.0537, 1082.0249, 1082.5898, 1103.5219, 1111.5421, 1113.5225, 1118.5022, 1136.5441, 1160.5824, 1174.5968, 1201.6826, 1234.6770, 1241.6234, 1241.6234, 1255.6364, 1277.7050, 1285.7051, 1285.7051, 1294.6182, 1300.5901, 1303.5570, 1308.6539, 1320.5820, 1350.5736, 1367.5901, 1368.5878, 1383.6404, 1399.7301, 1401.7665, 1415.7883, 1416.7784, 1418.6888, 1431.7214, 1434.7179, 1449.7212, 1465.6866, 1506.6792, 1523.7628, 1537.7434, 1638.8650, 1642.7389, 1707.7817, 1738.9299, 1993.9830, 2185.0576, 2188.0432, 2199.0037, 2224.9702, 2289.1655, 2335.0881

5. ACE_PANTR **Mass:** 150074 **Score:** 82 **Expect:** 0.00039 **Matches:** 3
Angiotensin-converting enzyme OS=Pan troglodytes GN=ACE PE=3 SV=1

Observed	Mr(expt)	Mr(calc)	ppm	Start - End	Miss	Ions	Peptide
1475.7599	1474.7526	1474.7303	15.1	728 - 740	0	---	R.AALPAQELEEYNK.I
1524.6910	1523.6837	1523.6793	2.90	1082 - 1092	0	---	K.ENYNQEWWSLR.L
1524.6910	1523.6837	1523.6793	2.90	1082 - 1092	0	82	K.ENYNQEWWSLR.L

No match to: 855.0244, 857.4423, 861.0447, 877.0201, 885.9838, 892.9934, 906.4807, 914.5364, 964.5336, 995.4992, 1018.4599, 1020.4916, 1036.5293, 1050.0758, 1050.5299, 1066.0537, 1082.0249, 1082.5898, 1103.5219, 1111.5421, 1113.5225, 1118.5022, 1136.5441, 1160.5824, 1174.5968, 1201.6826, 1234.6770, 1241.6234, 1241.6234, 1255.6364, 1277.7050, 1285.7051, 1285.7051, 1294.6182, 1300.5901, 1303.5570, 1308.6539, 1320.5820, 1350.5736, 1367.5901, 1368.5878, 1383.6404, 1399.7301, 1401.7665, 1415.7883, 1416.7784, 1418.6888, 1431.7214, 1434.7179, 1449.7212, 1465.6866, 1506.6792, 1523.7628, 1537.7434, 1638.8650, 1642.7389, 1707.7817, 1738.9299, 1993.9830, 2185.0576, 2188.0432, 2199.0037, 2224.9702, 2289.1655, 2335.0881

6. ACE_RABIT **Mass:** 151165 **Score:** 82 **Expect:** 0.00039 **Matches:** 3
Angiotensin-converting enzyme OS=Oryctolagus cuniculus GN=ACE PE=1 SV=3

Observed	Mr(expt)	Mr(calc)	ppm	Start - End	Miss	Ions	Peptide
1399.7301	1398.7228	1398.7442	-15.26	1099 - 1110	1	---	R.LKYQGLCPPAPR.S
1524.6910	1523.6837	1523.6793	2.90	1088 - 1098	0	---	K.ENYNQEWWSLR.L
1524.6910	1523.6837	1523.6793	2.90	1088 - 1098	0	82	K.ENYNQEWWSLR.L

No match to: 855.0244, 857.4423, 861.0447, 877.0201, 885.9838, 892.9934, 906.4807, 914.5364, 964.5336, 995.4992, 1018.4599, 1020.4916, 1036.5293, 1050.0758, 1050.5299, 1066.0537, 1082.0249, 1082.5898, 1103.5219, 1111.5421, 1113.5225, 1118.5022, 1136.5441, 1160.5824, 1174.5968, 1201.6826, 1234.6770, 1241.6234, 1241.6234, 1255.6364, 1277.7050, 1285.7051, 1285.7051, 1294.6182, 1300.5901, 1303.5570, 1308.6539, 1320.5820, 1350.5736, 1367.5901, 1368.5878, 1383.6404, 1401.7665, 1415.7883, 1416.7784, 1418.6888, 1431.7214, 1434.7179, 1449.7212, 1465.6866, 1475.7599, 1506.6792, 1523.7628, 1537.7434, 1638.8650, 1642.7389, 1707.7817, 1738.9299, 1993.9830, 2185.0576, 2188.0432, 2199.0037, 2224.9702, 2289.1655, 2335.0881

7. MYOF_HUMAN **Mass:** 236100 **Score:** 54 **Expect:** 0.24 **Matches:** 21
Myoferlin OS=Homo sapiens GN=MYOF PE=1 SV=1

Observed	Mr(expt)	Mr(calc)	ppm	Start - End	Miss	Ions	Peptide
906.4807	905.4734	905.5375	-70.73	357 - 363	0	---	R.WVTFLLK.I
964.5336	963.5263	963.4886	39.1	528 - 536	1	---	K.GEGVAYRGR.I
1018.4599	1017.4526	1017.5244	-70.49	883 - 891	1	---	R.HKFSDVTGK.I
1036.5293	1035.5220	1035.5019	19.5	740 - 748	1	---	R.MRSEATDVK.S
1113.5225	1112.5152	1112.5073	7.11	1400 - 1408	1	---	R.FRCDPYAGK.E
1174.5968	1173.5895	1173.5778	9.99	820 - 829	1	---	K.YPQEKNNGPK.V
1285.7051	1284.6978	1284.6384	46.3	1937 - 1947	1	---	K.TASLFEQKSMK.G + Oxidation (M)
1285.7051	1284.6978	1284.6384	46.3	1937 - 1947	1	---	K.TASLFEQKSMK.G + Oxidation (M)
1294.6182	1293.6109	1293.5738	28.7	1837 - 1847	0	---	R.SLDGEGNFNWR.F
1320.5820	1319.5747	1319.6034	-21.70	1447 - 1456	0	---	K.EEEIVDWWSK.F
1350.5736	1349.5663	1349.6735	-79.41	222 - 232	1	---	K.VHVCGQTHRTR.I
1383.6404	1382.6331	1382.6904	-41.45	1349 - 1360	0	---	K.TPNFPSSVLFMK.V + Oxidation (M)
1399.7301	1398.7228	1398.7368	-9.97	1715 - 1726	0	---	K.ILHQHLGAPEER.L

1416.7784	1415.7711	1415.7442	19.0	654	666	0	---	R.LDAVNTLLAMAER.L
1449.7212	1448.7139	1448.7776	-43.95	1770	1783	0	---	K.SLGPPGPPFNITPR.K
1523.7628	1522.7555	1522.7337	14.3	1211	1223	0	---	K.VIMELFDNDQVGK.D + Oxidation (M)
1642.7389	1641.7316	1641.8263	-57.69	1591	1604	0	---	R.DHYIPNTLNPVFGR.M
1993.9830	1992.9757	1993.0666	-45.61	1409	1426	1	---	K.EDIVPQLKASLLSAPPCR.D
2224.9702	2223.9629	2224.0834	-54.18	1211	1229	1	---	K.VIMELFDNDQVGKDEFLGR.S
2289.1655	2288.1582	2288.2185	-26.33	1427	1446	1	---	R.DIVIEMEDTKPLLASKLTEK.E + Oxidation (M)
2335.0881	2334.0808	2334.1467	-28.22	1996	2014	0	---	K.LDLPNRPETSFLWFTNPCK.T

No match to: 855.0244, 857.4423, 861.0447, 877.0201, 885.9838, 892.9934, 914.5364, 995.4992, 1020.4916, 1050.0758, 1050.5299, 1066.0537, 1082.0249, 1082.5898, 1103.5219, 1111.5421, 1118.5022, 1136.5441, 1160.5824, 1201.6826, 1234.6770, 1241.6234, 1241.6234, 1255.6364, 1277.7050, 1300.5901, 1303.5570, 1308.6539, 1367.5901, 1368.5878, 1401.7665, 1415.7883, 1418.6888, 1431.7214, 1434.7179, 1465.6866, 1475.7599, 1506.6792, 1524.6910, 1524.6910, 1537.7434, 1638.8650, 1707.7817, 1738.9299, 2185.0576, 2188.0432, 2199.0037

8. CJ120_BOVIN **Mass:** 39450 **Score:** 51 **Expect:** 0.57 **Matches:** 11

Uncharacterized protein C10orf120 homolog OS=Bos taurus PE=2 SV=1

Observed	Mr(expt)	Mr(calc)	ppm	Start	End	Miss	Ions	Peptide
964.5336	963.5263	963.4873	40.5	237	245	1	---	R.GEVESKTSK.R
1018.4599	1017.4526	1017.4767	-23.65	325	332	0	---	R.LEEENAWK.E
1399.7301	1398.7228	1398.7289	-4.36	199	213	0	---	R.GSSLLPSGGLGPMAR.A
1415.7883	1414.7810	1414.7238	40.4	199	213	0	---	R.GSSLLPSGGLGPMAR.A + Oxidation (M)
1434.7179	1433.7106	1433.6220	61.8	333	343	1	---	K.EYMCKVAPHHY.-
1449.7212	1448.7139	1448.7333	-13.39	251	262	1	---	K.MNVIFKSEEPQK.C
1465.6866	1464.6793	1464.7282	-33.40	251	262	1	---	K.MNVIFKSEEPQK.C + Oxidation (M)
1475.7599	1474.7526	1474.7602	-5.15	155	166	1	---	K.VIVSPEEFRMPR.R + Oxidation (M)
1642.7389	1641.7316	1641.8621	-79.44	199	215	1	---	R.GSSLLPSGGLGPMARAR.A + Oxidation (M)
2185.0576	2184.0503	2183.9064	65.9	216	236	1	---	R.AGEGQDDGNTDDGNDVHQKGR.G
2199.0037	2197.9964	2197.9221	33.8	214	234	1	---	R.ARAGEGQDDGNTDDGNDVHQK.G

No match to: 855.0244, 857.4423, 861.0447, 877.0201, 885.9838, 892.9934, 906.4807, 914.5364, 995.4992, 1020.4916, 1036.5293, 1050.0758, 1050.5299, 1066.0537, 1082.0249, 1082.5898, 1103.5219, 1111.5421, 1113.5225, 1118.5022, 1136.5441, 1160.5824, 1174.5968, 1201.6826, 1234.6770, 1241.6234, 1241.6234, 1255.6364, 1277.7050, 1285.7051, 1285.7051, 1294.6182, 1300.5901, 1303.5570, 1308.6539, 1320.5820, 1350.5736, 1367.5901, 1368.5878, 1383.6404, 1401.7665, 1416.7784, 1418.6888, 1431.7214, 1506.6792, 1523.7628, 1524.6910, 1524.6910, 1537.7434, 1638.8650, 1707.7817, 1738.9299, 1993.9830, 2188.0432, 2224.9702, 2289.1655, 2335.0881

9. MIA3_HUMAN **Mass:** 214255 **Score:** 50 **Expect:** 0.63 **Matches:** 10

Melanoma inhibitory activity protein 3 OS=Homo sapiens GN=MIA3 PE=1 SV=1

Observed	Mr(expt)	Mr(calc)	ppm	Start	End	Miss	Ions	Peptide
1103.5219	1102.5146	1102.5335	-17.13	69	77	0	---	K.GDPVYVVYK.L
1277.7050	1276.6977	1276.6333	50.5	1254	1264	0	---	K.QNMILSDEAIK.Y + Oxidation (M)
1300.5901	1299.5828	1299.6360	-40.94	81	92	0	---	R.GWPEVWAGSVGR.T
1449.7212	1448.7139	1448.7545	-27.98	1222	1233	1	---	K.TIMKENTELVQK.L + Oxidation (M)
1523.7628	1522.7555	1522.8355	-52.52	364	377	1	---	K.VQLTVPPGIKNDDK.N
1524.6910	1523.6837	1523.6926	-5.80	961	973	0	---	K.SAQQESLPYNMEK.V
1524.6910	1523.6837	1523.6926	-5.80	961	973	0	36	K.SAQQESLPYNMEK.V
1638.8650	1637.8577	1637.9691	-67.99	1	15	0	---	-.MAAAPGLLVWLLVLR.L + Oxidation (M)
1993.9830	1992.9757	1992.9476	14.1	1698	1715	1	---	R.DMPRSEFGSVDGPLPHPR.W
2185.0576	2184.0503	2184.1255	-34.44	93	110	1	---	R.TFGYFPKDLIQVVHEYTK.E

No match to: 855.0244, 857.4423, 861.0447, 877.0201, 885.9838, 892.9934, 906.4807, 914.5364, 964.5336, 995.4992, 1018.4599, 1020.4916, 1036.5293, 1050.0758, 1050.5299, 1066.0537, 1082.0249, 1082.5898, 1111.5421, 1113.5225, 1118.5022, 1136.5441, 1160.5824, 1174.5968, 1201.6826, 1234.6770, 1241.6234, 1241.6234, 1255.6364, 1285.7051, 1285.7051, 1294.6182, 1303.5570, 1308.6539, 1320.5820, 1350.5736, 1367.5901, 1368.5878, 1383.6404, 1399.7301, 1401.7665, 1415.7883, 1416.7784, 1418.6888, 1431.7214, 1434.7179, 1465.6866, 1475.7599, 1506.6792, 1537.7434, 1642.7389, 1707.7817, 1738.9299, 2188.0432, 2199.0037, 2224.9702, 2289.1655, 2335.0881

10. K2C8_HUMAN **Mass:** 53671 **Score:** 47 **Expect:** 1.3 **Matches:** 12

Keratin, type II cytoskeletal 8 OS=Homo sapiens GN=KRT8 PE=1 SV=7

Observed	Mr(expt)	Mr(calc)	ppm	Start	End	Miss	Ions	Peptide
906.4807	905.4734	905.4607	14.1	111	117	0	---	R.FLEQQNK.M
995.4992	994.4919	994.4389	53.3	305	312	0	---	K.TEISEMNR.N + Oxidation (M)
1082.5898	1081.5825	1081.5920	-8.79	102	110	1	---	K.FASFIDKVR.F
1201.6826	1200.6753	1200.6462	24.2	149	158	1	---	R.RQLETLGQEK.L
1277.7050	1276.6977	1276.7027	-3.87	382	392	0	---	K.LALDIEIATYR.K
1320.5820	1319.5747	1319.6642	-67.84	253	264	0	---	R.SLDMDSIIAEVK.A
1368.5878	1367.5805	1367.6642	-61.22	187	197	0	---	R.TEMENEFVLIK.K + Oxidation (M)
1449.7212	1448.7139	1448.7041	6.76	305	316	1	---	K.TEISEMNRNISR.L
1465.6866	1464.6793	1464.6991	-13.47	305	316	1	---	K.TEISEMNRNISR.L + Oxidation (M)
1475.7599	1474.7526	1474.6908	41.9	402	414	0	---	R.LESGMQNMSIHTK.T
1524.6910	1523.6837	1523.7653	-53.57	186	197	1	---	K.RTEMENEFVLIK.K + Oxidation (M)
1524.6910	1523.6837	1523.7653	-53.57	111	122	1	---	R.FLEQQNKMLETK.W + Oxidation (M)

No match to: 855.0244, 857.4423, 861.0447, 877.0201, 885.9838, 892.9934, 914.5364, 964.5336, 1018.4599, 1020.4916, 1036.5293, 1050.0758, 1050.5299, 1066.0537, 1082.0249, 1103.5219, 1111.5421, 1113.5225, 1118.5022, 1136.5441, 1160.5824, 1174.5968, 1234.6770, 1241.6234, 1241.6234, 1255.6364, 1285.7051, 1285.7051, 1294.6182, 1300.5901, 1303.5570, 1308.6539, 1350.5736, 1367.5901, 1383.6404, 1399.7301, 1401.7665, 1415.7883, 1416.7784, 1418.6888, 1431.7214, 1434.7179, 1506.6792, 1523.7628, 1537.7434, 1638.8650, 1642.7389, 1707.7817, 1738.9299, 1993.9830, 2185.0576, 2188.0432, 2199.0037, 2224.9702, 2289.1655, 2335.0881

11. GOGB1_HUMAN **Mass:** 377215 **Score:** 47 **Expect:** 1.5 **Matches:** 28

Golgin subfamily B member 1 OS=Homo sapiens GN=GOLGB1 PE=1 SV=2

Observed	Mr(expt)	Mr(calc)	ppm	Start	End	Miss	Ions	Peptide
964.5336	963.5263	963.5025	24.7	2073	2081	0	---	K.AQAELASFK.V
1020.4916	1019.4843	1019.5499	-64.27	1589	1597	1	---	K.EIESLKSSK.I
1050.5299	1049.5226	1049.5982	-71.98	1632	1640	0	---	R.IQHVVEAVR.Q
1103.5219	1102.5146	1102.5771	-56.64	363	371	1	---	R.YSALEQKHK.A
1111.5421	1110.5348	1110.5128	19.9	2027	2034	1	---	K.DCIRYQEK.I

1113.5225	1112.5152	1112.5536	-34.47	1741	1749	1	---	K.KFQSLMSEK.D + Oxidation (M)
1118.5022	1117.4949	1117.5251	-27.00	844	853	0	---	K.ESEVLEGAER.V
1136.5441	1135.5368	1135.5543	-15.40	1549	1557	1	---	R.DKLITEMDR.S + Oxidation (M)
1160.5824	1159.5751	1159.5469	24.3	2344	2353	0	---	R.QQEADIQNSK.F
1174.5968	1173.5895	1173.6679	-66.75	558	567	0	---	K.ELSVLLLEMK.E
1201.6826	1200.6753	1200.6350	33.6	1025	1034	1	---	K.KEIPLSETER.G
1234.6770	1233.6697	1233.7333	-51.50	922	932	1	---	K.FSLGVEIKTLK.E
1277.7050	1276.6977	1276.6155	64.4	1456	1465	0	---	K.QLQVELCEMK.Q
1285.7051	1284.6978	1284.6496	37.5	1438	1448	0	---	K.ALHTQLEMQAK.E + Oxidation (M)
1285.7051	1284.6978	1284.6496	37.5	1438	1448	0	---	K.ALHTQLEMQAK.E + Oxidation (M)
1294.6182	1293.6109	1293.6789	-52.55	2061	2071	1	---	K.ENLAQAVEHRK.K
1303.5570	1302.5497	1302.6237	-56.83	768	778	0	---	K.QLEMNLAEAER.Q
1308.6539	1307.6466	1307.5888	44.2	2882	2892	1	---	K.AMSSLQNDRDR.L + Oxidation (M)
1383.6404	1382.6331	1382.6314	1.27	585	596	0	---	R.AEEADHEVLDQK.E
1416.7784	1415.7711	1415.7507	14.4	523	535	0	---	R.EVSEISIVDIANK.R
1431.7214	1430.7141	1430.7261	-8.38	600	612	1	---	K.QMEGEGIAPIKMK.V
1475.7599	1474.7526	1474.7263	17.8	1519	1532	0	---	K.SLADVESQVSAQNK.E
1506.6792	1505.6719	1505.7759	-69.07	2434	2446	1	---	K.AVDKTNQLMETLK.T + Oxidation (M)
1642.7389	1641.7316	1641.7556	-14.60	900	913	0	---	K.DQQVTEISFSMTEK.M
1707.7817	1706.7744	1706.7305	25.8	135	148	1	---	K.HDKSSTEEEMEIEK.I + Oxidation (M)
2185.0576	2184.0503	2184.1889	-63.43	1419	1437	1	---	K.EAALTKIQTEIIEQEDLIK.A
2199.0037	2197.9964	2198.0087	-5.59	536	555	1	---	K.RSSSAEESGQDVLENTFSQK.H
2289.1655	2288.1582	2288.2488	-39.58	1374	1393	1	---	K.LESSQLQIAGLEHLRELQPK.L

No match to: 855.0244, 857.4423, 861.0447, 877.0201, 885.9838, 892.9934, 906.4807, 914.5364, 995.4992, 1018.4599, 1036.5293, 1050.0758, 1066.0537, 1082.0249, 1082.5898, 1241.6234, 1241.6234, 1255.6364, 1300.5901, 1320.5820, 1350.5736, 1367.5901, 1368.5878, 1399.7301, 1401.7665, 1415.7883, 1418.6888, 1434.7179, 1449.7212, 1465.6866, 1523.7628, 1524.6910, 1524.6910, 1537.7434, 1638.8650, 1738.9299, 1993.9830, 2188.0432, 2224.9702, 2335.0881

12. ILKAP_HUMAN **Mass:** 43450 **Score:** 45 **Expect:** 2.2 **Matches:** 10

Integrin-linked kinase-associated serine/threonine phosphatase 2C OS=Homo sapiens GN=ILKAP PE=1 SV=1

Observed	Mr(expt)	Mr(calc)	ppm	Start	End	Miss	Ions	Peptide
1018.4599	1017.4526	1017.4767	-23.68	197	204	0	---	K.HTDEEFLK.Q
1103.5219	1102.5146	1102.5441	-26.75	301	310	0	---	R.CGVTSVPDIR.R
1174.5968	1173.5895	1173.6605	-60.48	178	188	1	---	K.GDVISVEKTVK.R
1308.6539	1307.6466	1307.7271	-61.57	97	108	1	---	K.VCKASSVIFGLK.G
1418.6888	1417.6815	1417.6548	18.9	1	12	0	---	-.MDLFGDLPEPER.S
1434.7179	1433.7106	1433.6497	42.5	1	12	0	---	-.MDLFGDLPEPER.S + Oxidation (M)
1524.6910	1523.6837	1523.7521	-44.90	145	158	0	---	R.VSYFAVFDGHGGIR.A
1524.6910	1523.6837	1523.7521	-44.90	145	158	0	---	R.VSYFAVFDGHGGIR.A
1707.7817	1706.7744	1706.7846	-5.97	81	95	1	---	K.TSEEEKNGSEELVEK.K
2199.0037	2197.9964	2198.0790	-37.56	1	20	1	---	-.MDLFGDLPEPERSPRPAAGK.E + Oxidation (M)

No match to: 855.0244, 857.4423, 861.0447, 877.0201, 885.9838, 892.9934, 906.4807, 914.5364, 964.5336, 995.4992, 1020.4916, 1036.5293, 1050.0758, 1050.5299, 1066.0537, 1082.0249, 1082.5898, 1111.5421, 1113.5225, 1118.5022, 1136.5441, 1160.5824, 1201.6826, 1234.6770, 1241.6234, 1241.6234, 1255.6364, 1277.7050, 1285.7051, 1285.7051, 1294.6182, 1300.5901, 1303.5570, 1320.5820, 1350.5736, 1367.5901, 1368.5878, 1383.6404, 1399.7301, 1401.7665, 1415.7883, 1416.7784, 1431.7214, 1449.7212, 1465.6866, 1475.7599, 1506.6792, 1523.7628, 1537.7434, 1638.8650, 1642.7389, 1738.9299, 1993.9830, 2185.0576, 2188.0432, 2224.9702, 2289.1655, 2335.0881

13. K2C8_RAT **Mass:** 53985 **Score:** 44 **Expect:** 2.6 **Matches:** 12

Keratin, type II cytoskeletal 8 OS=Rattus norvegicus GN=Krt8 PE=1 SV=3

Observed	Mr(expt)	Mr(calc)	ppm	Start	End	Miss	Ions	Peptide
906.4807	905.4734	905.4607	14.1	111	117	0	---	R.FLEQQNK.M
964.5336	963.5263	963.5172	9.52	1	8	1	---	-.MSVRVTQK.S + Oxidation (M)
995.4992	994.4919	994.4389	53.3	305	312	0	---	K.TEISEMNR.N + Oxidation (M)
1082.5898	1081.5825	1081.5920	-8.79	102	110	1	---	K.FASFIDKVR.F
1113.5225	1112.5152	1112.5284	-11.87	9	18	1	---	K.SYKMSTSGPR.A
1277.7050	1276.6977	1276.7027	-3.87	382	392	0	---	K.LALDIEIATYR.K
1368.5878	1367.5805	1367.6642	-61.22	187	197	0	---	R.TEMENEFVLIK.K + Oxidation (M)
1449.7212	1448.7139	1448.7041	6.76	305	316	1	---	K.TEISEMNRNISR.L
1465.6866	1464.6793	1464.6991	-13.47	305	316	1	---	K.TEISEMNRNISR.L + Oxidation (M)
1475.7599	1474.7526	1474.6908	41.9	402	414	0	---	R.LESGMQNMSIHTK.T
1524.6910	1523.6837	1523.7653	-53.57	186	197	1	---	K.RTEMENEFVLIK.K + Oxidation (M)
1524.6910	1523.6837	1523.7653	-53.57	111	122	1	---	R.FLEQQNKMLETK.W + Oxidation (M)

No match to: 855.0244, 857.4423, 861.0447, 877.0201, 885.9838, 892.9934, 914.5364, 1018.4599, 1020.4916, 1036.5293, 1050.0758, 1050.5299, 1066.0537, 1082.0249, 1103.5219, 1111.5421, 1118.5022, 1136.5441, 1160.5824, 1174.5968, 1201.6826, 1234.6770, 1241.6234, 1241.6234, 1255.6364, 1285.7051, 1285.7051, 1294.6182, 1300.5901, 1303.5570, 1308.6539, 1320.5820, 1350.5736, 1367.5901, 1383.6404, 1399.7301, 1401.7665, 1415.7883, 1416.7784, 1418.6888, 1431.7214, 1434.7179, 1506.6792, 1523.7628, 1537.7434, 1638.8650, 1642.7389, 1707.7817, 1738.9299, 1993.9830, 2185.0576, 2188.0432, 2199.0037, 2224.9702, 2289.1655, 2335.0881

14. UACA_CANLF **Mass:** 163865 **Score:** 44 **Expect:** 2.8 **Matches:** 20

Uveal autoantigen with coiled-coil domains and ankyrin repeats OS=Canis lupus familiaris GN=UACA PE=2 SV=2

Observed	Mr(expt)	Mr(calc)	ppm	Start	End	Miss	Ions	Peptide
857.4423	856.4350	856.5018	-77.95	825	831	0	---	R.QLLELNK.K
1036.5293	1035.5220	1035.4841	36.6	931	939	0	---	K.MSAINQNMK.S
1111.5421	1110.5348	1110.5319	2.67	27	35	1	---	K.NRHAADWNK.Y
1136.5441	1135.5368	1135.5179	16.7	1006	1014	0	---	K.EQLSEQMQK.Y + Oxidation (M)
1160.5824	1159.5751	1159.5721	2.65	579	588	1	---	R.DEGKLVEENK.R
1174.5968	1173.5895	1173.5989	-8.02	909	918	0	---	R.NLENTQSQIK.A
1201.6826	1200.6753	1200.6350	33.6	1320	1329	0	---	K.ITELLNDVER.L
1234.6770	1233.6697	1233.6969	-22.00	1035	1044	1	---	K.EIFTLQKDLK.D
1241.6234	1240.6161	1240.5506	52.8	450	459	1	---	R.TFCESAKQDR.L
1241.6234	1240.6161	1240.5506	52.8	450	459	1	---	R.TFCESAKQDR.L
1383.6404	1382.6331	1382.6500	-12.20	1140	1150	1	---	K.CYEKEQQTVAK.L

1416.7784	1415.7711	1415.7984	-19.23	840 -	852	1	---	K.INALVSENTSLKK.T
1418.6888	1417.6815	1417.7235	-29.62	766 -	777	1	---	K.QLLDVTQKCADK.Q
1431.7214	1430.7141	1430.7630	-34.15	568 -	578	1	---	K.QNELLVEQFRR.D
1434.7179	1433.7106	1433.7011	6.64	524 -	536	0	---	K.EHLTSEAAIGNHR.L
1506.6792	1505.6719	1505.7395	-44.89	1003 -	1014	1	---	K.ELKEQLSEQMQK.Y + Oxidation (M)
1523.7628	1522.7555	1522.7119	28.6	590 -	601	1	---	R.LQKELSMCETER.D
1537.7434	1536.7361	1536.8260	-58.48	1351 -	1363	0	---	R.QSQLIDTLQHQVK.S
1707.7817	1706.7744	1706.8587	-49.39	940 -	954	1	---	K.SVQDNSAEILANYRK.G
2289.1655	2288.1582	2287.9959	71.0	11 -	28	1	---	R.NQILSMMNCWFSCAPKNR.H + 2 Oxidation (M)

No match to: 855.0244, 861.0447, 877.0201, 885.9838, 892.9934, 906.4807, 914.5364, 964.5336, 995.4992, 1018.4599, 1020.4916, 1050.0758, 1050.5299, 1066.0537, 1082.0249, 1082.5898, 1103.5219, 1113.5225, 1118.5022, 1255.6364, 1277.7050, 1285.7051, 1285.7051, 1294.6182, 1300.5901, 1303.5570, 1308.6539, 1320.5820, 1350.5736, 1367.5901, 1368.5878, 1399.7301, 1401.7665, 1415.7883, 1449.7212, 1465.6866, 1475.7599, 1524.6910, 1524.6910, 1638.8650, 1642.7389, 1738.9299, 1993.9830, 2185.0576, 2188.0432, 2199.0037, 2224.9702, 2335.0881

15. SYDC_HUMAN **Mass:** 57499 **Score:** 43 **Expect:** 3.1 **Matches:** 10

Aspartate--tRNA ligase, cytoplasmic OS=Homo sapiens GN=DARS PE=1 SV=2

Observed	Mr(expt)	Mr(calc)	ppm	Start	End	Miss	Ions	Peptide
1160.5824	1159.5751	1159.6601	-73.26	142 -	151	0	---	K.IYVISLAEPR.L
1201.6826	1200.6753	1200.6350	33.6	111 -	121	0	---	K.ESIVDVEGVVR.K
1255.6364	1254.6291	1254.5438	68.0	16 -	26	0	---	R.EIMDAAEDYAK.E
1294.6182	1293.6109	1293.5118	76.6	412 -	421	0	---	K.QSNSYDMFMR.G + Oxidation (M)
1368.5878	1367.5805	1367.6577	-56.43	346 -	356	0	---	R.LEYCEALAMLR.E
1399.7301	1398.7228	1398.7806	-41.29	476 -	487	0	---	R.VTMLFLGLHNVR.Q
1415.7883	1414.7810	1414.7755	3.91	476 -	487	0	---	R.VTMLFLGLHNVR.Q + Oxidation (M)
1738.9299	1737.9226	1737.8356	50.1	29 -	43	0	---	R.YGISSMIQSQEKPDR.V
2224.9702	2223.9629	2224.1384	-78.89	339 -	356	1	---	K.FLEPTLRLEYCEALAMLR.E
2335.0881	2334.0808	2334.0369	18.8	412 -	431	1	---	K.QSNSYDMFMRGEEILSGAQR.I + Oxidation (M)

No match to: 855.0244, 857.4423, 861.0447, 877.0201, 885.9838, 892.9934, 906.4807, 914.5364, 964.5336, 995.4992, 1018.4599, 1020.4916, 1036.5293, 1050.0758, 1050.5299, 1066.0537, 1082.0249, 1082.5898, 1103.5219, 1111.5421, 1113.5225, 1118.5022, 1136.5441, 1174.5968, 1234.6770, 1241.6234, 1241.6234, 1277.7050, 1285.7051, 1285.7051, 1300.5901, 1303.5570, 1308.6539, 1320.5820, 1350.5736, 1367.5901, 1383.6404, 1401.7665, 1416.7784, 1418.6888, 1431.7214, 1434.7179, 1449.7212, 1465.6866, 1475.7599, 1506.6792, 1523.7628, 1524.6910, 1524.6910, 1537.7434, 1638.8650, 1642.7389, 1707.7817, 1993.9830, 2185.0576, 2188.0432, 2199.0037, 2289.1655

16. IFT81_MOUSE **Mass:** 79692 **Score:** 42 **Expect:** 4.2 **Matches:** 17

Intraflagellar transport protein 81 homolog OS=Mus musculus GN=Ift81 PE=1 SV=4

Observed	Mr(expt)	Mr(calc)	ppm	Start	End	Miss	Ions	Peptide
914.5364	913.5291	913.5021	29.5	291 -	297	0	---	K.ELHFLQK.V
1050.5299	1049.5226	1049.4811	39.5	473 -	481	1	---	K.SEVDEMKGR.T
1103.5219	1102.5146	1102.5917	-69.90	243 -	251	1	---	R.VQNQLKSMR.H
1118.5022	1117.4949	1117.5114	-14.73	272 -	280	0	---	K.FNSYMVTEK.F
1241.6234	1240.6161	1240.5982	14.4	624 -	634	1	---	K.AVRESHGPNMK.Q + Oxidation (M)
1241.6234	1240.6161	1240.5982	14.4	624 -	634	1	---	K.AVRESHGPNMK.Q + Oxidation (M)
1277.7050	1276.6977	1276.6411	44.3	217 -	226	1	---	K.EREEFLAQQK.Q
1285.7051	1284.6978	1284.6496	37.5	153 -	162	1	---	K.TLHKECEQLK.T
1285.7051	1284.6978	1284.6496	37.5	153 -	162	1	---	K.TLHKECEQLK.T
1303.5570	1302.5497	1302.5802	-23.37	143 -	152	0	---	K.QYEELMEAFK.T + Oxidation (M)
1368.5878	1367.5805	1367.6867	-77.63	252 -	264	0	---	R.HAAADAKPESLMK.R
1418.6888	1417.6815	1417.7565	-52.87	334 -	345	1	---	R.NEPIEGKLSLYR.Q
1506.6792	1505.6719	1505.7224	-33.55	272 -	283	1	---	K.FNSYMVTEKFPK.E + Oxidation (M)
1524.6910	1523.6837	1523.7878	-68.30	252 -	265	1	---	R.HAAADAKPESLMKR.L
1524.6910	1523.6837	1523.7878	-68.30	252 -	265	1	---	R.HAAADAKPESLMKR.L
1638.8650	1637.8577	1637.7364	74.1	638 -	649	1	---	K.MWRDLEQLMECK.K
1642.7389	1641.7316	1641.8573	-76.56	316 -	329	0	---	K.VNEVNTEINQLIEK.K

No match to: 855.0244, 857.4423, 861.0447, 877.0201, 885.9838, 892.9934, 906.4807, 964.5336, 995.4992, 1018.4599, 1020.4916, 1036.5293, 1050.0758, 1066.0537, 1082.0249, 1082.5898, 1111.5421, 1113.5225, 1136.5441, 1160.5824, 1174.5968, 1201.6826, 1234.6770, 1255.6364, 1294.6182, 1300.5901, 1308.6539, 1320.5820, 1350.5736, 1367.5901, 1383.6404, 1399.7301, 1401.7665, 1415.7883, 1416.7784, 1431.7214, 1434.7179, 1449.7212, 1465.6866, 1475.7599, 1523.7628, 1537.7434, 1707.7817, 1738.9299, 1993.9830, 2185.0576, 2188.0432, 2199.0037, 2224.9702, 2289.1655, 2335.0881

17. WDR87_HUMAN **Mass:** 335256 **Score:** 40 **Expect:** 5.9 **Matches:** 22

WD repeat-containing protein 87 OS=Homo sapiens GN=WDR87 PE=1 SV=3

Observed	Mr(expt)	Mr(calc)	ppm	Start	End	Miss	Ions	Peptide
857.4423	856.4350	856.3756	69.4	2693 -	2698	0	---	K.DFWDFK.D
995.4992	994.4919	994.5560	-64.38	2651 -	2659	0	---	R.AQQISIAHK.E
1018.4599	1017.4526	1017.5283	-74.41	1516 -	1522	1	---	K.LKWEEWK.Q
1020.4916	1019.4843	1019.4706	13.5	2535 -	2543	0	---	R.MEAGEQLSR.D
1036.5293	1035.5220	1035.4655	54.6	2535 -	2543	0	---	R.MEAGEQLSR.D + Oxidation (M)
1082.5898	1081.5825	1081.4961	79.9	50 -	58	1	---	K.SKTEDMVEK.R + Oxidation (M)
1118.5022	1117.4949	1117.5615	-59.55	1648 -	1657	1	---	R.GEKLSQEAEK.L
1136.5441	1135.5368	1135.5655	-25.28	2588 -	2597	1	---	R.ISSRQSMSPK.Y + Oxidation (M)
1160.5824	1159.5751	1159.5833	-7.04	1641 -	1650	1	---	K.EETLAQRGEK.L
1277.7050	1276.6977	1276.6775	15.8	829 -	839	1	---	R.VRAISNTEYPK.N
1300.5901	1299.5828	1299.6459	-48.54	2672 -	2683	0	---	R.DIFPSAHASVEK.Q
1320.5820	1319.5747	1319.6503	-57.30	530 -	541	0	---	K.SVGAITETNCLR.L
1350.5736	1349.5663	1349.5558	7.83	842 -	851	0	---	K.EEDEHFLEMR.L + Oxidation (M)
1368.5878	1367.5805	1367.5975	-12.45	331 -	340	1	---	R.RVCCGNNWFR.I
1399.7301	1398.7228	1398.8082	-61.03	12 -	23	1	---	K.DLKLLLNDTINK.S
1418.6888	1417.6815	1417.7486	-47.32	2023 -	2035	1	---	R.ILAMEESEIAKGK.L
1431.7214	1430.7141	1430.7630	-34.15	270 -	281	1	---	K.EWNLTSGSLLRR.L
1434.7179	1433.7106	1433.6998	7.56	1982 -	1994	1	---	K.ETLSKGETPETSR.Q
1523.7628	1522.7555	1522.8290	-48.24	181 -	194	1	---	R.VLMHQGKGQLGEVK.R

1537.7434	1536.7361	1536.7606	-15.93	1998 - 2009	1	---	K.MTQVEQELFERK.L
1738.9299	1737.9226	1737.8897	18.9	852 - 867	1	---	R.LSKDVTYSVLTDGANR.S
2199.0037	2197.9964	2197.9970	-0.25	813 - 828	1	---	R.ELEWDRSQEFFFWHSR.V

No match to: 855.0244, 861.0447, 877.0201, 885.9838, 892.9934, 906.4807, 914.5364, 964.5336, 1050.0758, 1050.5299, 1066.0537, 1082.0249, 1103.5219, 1111.5421, 1113.5225, 1174.5968, 1201.6826, 1234.6770, 1241.6234, 1241.6234, 1255.6364, 1285.7051, 1285.7051, 1294.6182, 1303.5570, 1308.6539, 1367.5901, 1383.6404, 1401.7665, 1415.7883, 1416.7784, 1449.7212, 1465.6866, 1475.7599, 1506.6792, 1524.6910, 1524.6910, 1638.8650, 1642.7389, 1707.7817, 1993.9830, 2185.0576, 2188.0432, 2224.9702, 2289.1655, 2335.0881

18. TSG10_RAT **Mass:** 83644 **Score:** 40 **Expect:** 6.6 **Matches:** 15

Testis-specific gene 10 protein OS=Rattus norvegicus GN=Tsga10 PE=1 SV=2

Observed	Mr(expt)	Mr(calc)	ppm	Start - End	Miss	Ions	Peptide
906.4807	905.4734	905.4164	63.0	505 - 511	0	---	K.MEEELQK.V
1050.5299	1049.5226	1049.5288	-5.85	115 - 123	1	---	R.MTTARDSLR.E
1113.5225	1112.5152	1112.5284	-11.85	612 - 620	0	---	K.EHLCLAENK.M
1118.5022	1117.4949	1117.5437	-43.69	18 - 27	0	---	R.AANCDVDLLK.S
1174.5968	1173.5895	1173.6353	-39.02	533 - 542	1	---	K.LDSSKELLNR.Q
1241.6234	1240.6161	1240.5250	73.5	81 - 90	1	---	R.EMMKSCQSPK.S + Oxidation (M)
1241.6234	1240.6161	1240.5250	73.5	81 - 90	1	---	R.EMMKSCQSPK.S + Oxidation (M)
1277.7050	1276.6977	1276.6081	70.2	301 - 311	0	---	K.NIIAEMEQASR.Q + Oxidation (M)
1294.6182	1293.6109	1293.6057	4.04	269 - 278	1	---	K.ECLQTCLDKK.S
1415.7883	1414.7810	1414.7416	27.9	385 - 396	1	---	K.QKVQDTNLEVNK.L
1449.7212	1448.7139	1448.7180	-2.85	279 - 292	0	---	K.SENIASLGESLAMK.E
1465.6866	1464.6793	1464.7130	-22.97	279 - 292	0	---	K.SENIASLGESLAMK.E + Oxidation (M)
1475.7599	1474.7526	1474.7641	-7.75	240 - 251	1	---	K.IDNFTRQNIAQR.E
1537.7434	1536.7361	1536.7494	-8.61	505 - 516	1	---	K.MEEELQKVQFEK.V
2289.1655	2288.1582	2288.0772	35.4	157 - 175	1	---	R.MEQMSNMTLMKETITIVEK.E + 2 Oxidation (M)

No match to: 855.0244, 857.4423, 861.0447, 877.0201, 885.9838, 892.9934, 914.5364, 964.5336, 995.4992, 1018.4599, 1020.4916, 1036.5293, 1050.0758, 1066.0537, 1082.0249, 1082.5898, 1103.5219, 1111.5421, 1136.5441, 1160.5824, 1201.6826, 1234.6770, 1255.6364, 1285.7051, 1285.7051, 1300.5901, 1303.5570, 1308.6539, 1320.5820, 1350.5736, 1367.5901, 1368.5878, 1383.6404, 1399.7301, 1401.7665, 1416.7784, 1418.6888, 1431.7214, 1434.7179, 1506.6792, 1523.7628, 1524.6910, 1524.6910, 1638.8650, 1642.7389, 1707.7817, 1738.9299, 1993.9830, 2185.0576, 2188.0432, 2199.0037, 2224.9702, 2335.0881

19. SERC_MOUSE **Mass:** 40732 **Score:** 40 **Expect:** 7 **Matches:** 8

Phosphoserine aminotransferase OS=Mus musculus GN=Psat1 PE=1 SV=1

Observed	Mr(expt)	Mr(calc)	ppm	Start - End	Miss	Ions	Peptide
1020.4916	1019.4843	1019.5334	-48.18	299 - 306	1	---	R.SRMNIPFR.I
1036.5293	1035.5220	1035.5284	-6.12	299 - 306	1	---	R.SRMNIPFR.I + Oxidation (M)
1111.5421	1110.5348	1110.6186	-75.42	118 - 127	0	---	K.FGTVNIVHPK.L
1113.5225	1112.5152	1112.5979	-74.27	6 - 16	0	---	K.QVVNFGPGPAK.L
1160.5824	1159.5751	1159.6237	-41.88	62 - 71	0	---	R.ELLAVPNNYK.V
1506.6792	1505.6719	1505.7507	-52.35	260 - 274	1	---	K.NNGGAAAMEKLSSIK.S + Oxidation (M)
1638.8650	1637.8577	1637.8162	25.4	95 - 110	1	---	K.AGRSADYVVTGAWSAK.A
2289.1655	2288.1582	2288.3005	-62.19	191 - 213	1	---	K.FGVIFAGAQKNVGSAGVTVVIVR.D

No match to: 855.0244, 857.4423, 861.0447, 877.0201, 885.9838, 892.9934, 906.4807, 914.5364, 964.5336, 995.4992, 1018.4599, 1050.0758, 1050.5299, 1066.0537, 1082.0249, 1082.5898, 1103.5219, 1118.5022, 1136.5441, 1174.5968, 1201.6826, 1234.6770, 1241.6234, 1241.6234, 1255.6364, 1277.7050, 1285.7051, 1285.7051, 1294.6182, 1300.5901, 1303.5570, 1308.6539, 1320.5820, 1350.5736, 1367.5901, 1368.5878, 1383.6404, 1399.7301, 1401.7665, 1415.7883, 1416.7784, 1418.6888, 1431.7214, 1434.7179, 1449.7212, 1465.6866, 1475.7599, 1523.7628, 1524.6910, 1524.6910, 1537.7434, 1642.7389, 1707.7817, 1738.9299, 1993.9830, 2185.0576, 2188.0432, 2199.0037, 2224.9702, 2335.0881

20. SPTN1_MOUSE **Mass:** 285221 **Score:** 40 **Expect:** 7.1 **Matches:** 22

Spectrin alpha chain, non-erythrocytic 1 OS=Mus musculus GN=Sptan1 PE=1 SV=4

Observed	Mr(expt)	Mr(calc)	ppm	Start - End	Miss	Ions	Peptide
857.4423	856.4350	856.4629	-32.57	1093 - 1098	1	---	K.KFMLFR.E + Oxidation (M)
914.5364	913.5291	913.5345	-5.87	758 - 765	1	---	K.KQEALVAR.Y
1118.5022	1117.4949	1117.5152	-18.17	1238 - 1246	1	---	R.FHRDADETK.E
1160.5824	1159.5751	1159.6561	-69.80	1055 - 1065	1	---	R.ITKEAGSVSLR.M
1234.6770	1233.6697	1233.6149	44.5	556 - 565	1	---	R.NALHERAMHR.R
1241.6234	1240.6161	1240.6775	-49.50	74 - 84	1	---	K.DPTNLQGKLQK.H
1241.6234	1240.6161	1240.6775	-49.50	74 - 84	1	---	K.DPTNLQGKLQK.H
1308.6539	1307.6466	1307.5854	46.8	1940 - 1950	0	---	K.NNHHEENISSK.M
1368.5878	1367.5805	1367.5663	10.4	475 - 485	0	---	R.DTEQVDNWMSK.Q + Oxidation (M)
1399.7301	1398.7228	1398.7983	-53.95	450 - 460	1	---	R.TALLELWELRR.Q
1401.7665	1400.7592	1400.7372	15.7	1046 - 1057	1	---	R.QGQIDNQTRITK.E
1415.7883	1414.7810	1414.7317	34.9	1215 - 1225	1	---	R.WRSLQQLAEER.S
1416.7784	1415.7711	1415.7633	5.51	2064 - 2075	1	---	K.RWTQLLANSATR.K
1431.7214	1430.7141	1430.7088	3.69	2343 - 2353	1	---	R.LNHQEFKSCLR.S
1449.7212	1448.7139	1448.6783	24.6	581 - 592	1	---	R.DSDELKSWVNEK.M
1475.7599	1474.7526	1474.7086	29.9	1926 - 1938	0	---	R.VNDVCTNGQDLIK.K
1524.6910	1523.6837	1523.6450	25.4	1772 - 1783	1	---	R.DMDDEESWIKEK.K
1524.6910	1523.6837	1523.6450	25.4	1772 - 1783	1	---	R.DMDDEESWIKEK.K
1707.7817	1706.7744	1706.8529	-45.97	567 - 580	0	---	R.AQLADSFHLQQFFR.D
2199.0037	2197.9964	2198.0830	-39.38	1094 - 1110	1	---	K.FMLFREANELQQWITEK.E + Oxidation (M)
2224.9702	2223.9629	2224.1297	-74.98	1890 - 1910	0	---	K.MTLVASEDYGDTLAAIQGLLK.K + Oxidation (M)
2335.0881	2334.0808	2334.1638	-35.54	275 - 295	1	---	K.EQLMASDDFGRDLASVQALLR.K

No match to: 855.0244, 861.0447, 877.0201, 885.9838, 892.9934, 906.4807, 964.5336, 995.4992, 1018.4599, 1020.4916, 1036.5293, 1050.0758, 1050.5299, 1066.0537, 1082.0249, 1082.5898, 1103.5219, 1111.5421, 1113.5225, 1136.5441, 1174.5968, 1201.6826, 1255.6364, 1277.7050, 1285.7051, 1285.7051, 1294.6182, 1300.5901, 1303.5570, 1320.5820, 1350.5736, 1367.5901, 1383.6404, 1418.6888, 1434.7179, 1465.6866, 1506.6792, 1523.7628, 1537.7434, 1638.8650, 1642.7389, 1738.9299, 1993.9830, 2185.0576, 2188.0432, 2289.1655

Search Parameters

```
Type of search         : Sequence Query
Enzyme                 : Trypsin
Fixed modifications    : Carbamidomethyl (C)
Variable modifications : Oxidation (M)
Mass values            : Monoisotopic
Protein Mass           : Unrestricted
Peptide Mass Tolerance : ± 80 ppm
Fragment Mass Tolerance: ± 0.3 Da
Max Missed Cleavages   : 1
Instrument type        : MALDI-TOF-TOF
Query1 (855.0244,1+)   : <no title>
Query2 (857.4423,1+)   : <no title>
Query3 (861.0447,1+)   : <no title>
Query4 (877.0201,1+)   : <no title>
Query5 (885.9838,1+)   : <no title>
Query6 (892.9934,1+)   : <no title>
Query7 (906.4807,1+)   : <no title>
Query8 (914.5364,1+)   : <no title>
Query9 (964.5336,1+)   : <no title>
Query10 (995.4992,1+)  : <no title>
Query11 (1018.4599,1+) : <no title>
Query12 (1020.4916,1+) : <no title>
Query13 (1036.5293,1+) : <no title>
Query14 (1050.0758,1+) : <no title>
Query15 (1050.5299,1+) : <no title>
Query16 (1066.0537,1+) : <no title>
Query17 (1082.0249,1+) : <no title>
Query18 (1082.5898,1+) : <no title>
Query19 (1103.5219,1+) : <no title>
Query20 (1111.5421,1+) : <no title>
Query21 (1113.5225,1+) : <no title>
Query22 (1118.5022,1+) : <no title>
Query23 (1136.5441,1+) : <no title>
Query24 (1160.5824,1+) : <no title>
Query25 (1174.5968,1+) : <no title>
Query26 (1201.6826,1+) : <no title>
Query27 (1234.6770,1+) : <no title>
Query28 (1241.6234,1+) : <no title>
Query29 (1241.6234,1+) : MaldiWellID: 55963, SpectrumID: 109842,
Query30 (1255.6364,1+) : <no title>
Query31 (1277.7050,1+) : <no title>
Query32 (1285.7051,1+) : <no title>
Query33 (1285.7051,1+) : MaldiWellID: 55963, SpectrumID: 109843,
Query34 (1294.6182,1+) : <no title>
Query35 (1300.5901,1+) : <no title>
Query36 (1303.5570,1+) : <no title>
Query37 (1308.6539,1+) : <no title>
Query38 (1320.5820,1+) : <no title>
Query39 (1350.5736,1+) : <no title>
Query40 (1367.5901,1+) : <no title>
Query41 (1368.5878,1+) : <no title>
Query42 (1383.6404,1+) : <no title>
Query43 (1399.7301,1+) : <no title>
Query44 (1401.7665,1+) : <no title>
Query45 (1415.7883,1+) : <no title>
Query46 (1416.7784,1+) : <no title>
Query47 (1418.6888,1+) : <no title>
Query48 (1431.7214,1+) : <no title>
Query49 (1434.7179,1+) : <no title>
Query50 (1449.7212,1+) : <no title>
Query51 (1465.6866,1+) : <no title>
Query52 (1475.7599,1+) : <no title>
Query53 (1506.6792,1+) : <no title>
Query54 (1523.7628,1+) : <no title>
Query55 (1524.6910,1+) : <no title>
Query56 (1524.6910,1+) : MaldiWellID: 55963, SpectrumID: 109841,
Query57 (1537.7434,1+) : <no title>
Query58 (1638.8650,1+) : <no title>
Query59 (1642.7389,1+) : <no title>
Query60 (1707.7817,1+) : <no title>
Query61 (1738.9299,1+) : <no title>
Query62 (1993.9830,1+) : <no title>
Query63 (2185.0576,1+) : <no title>
Query64 (2188.0432,1+) : <no title>
Query65 (2199.0037,1+) : <no title>
Query66 (2224.9702,1+) : <no title>
Query67 (2289.1655,1+) : <no title>
Query68 (2335.0881,1+) : <no title>
```

Mascot: http://www.matrixscience.com/
