# Supplementary material for: Vasectomy and Photoperiodic Regimen Modify the Protein Profile, Hormonal Content and Antioxidant Enzymes Activity of Ram Seminal Plasma
Source: Int J Mol Sci. 2020 Oct 29;21(21):8063. doi: 10.3390/ijms21218063 (PMC7663742; doi:10.3390/ijms21218063)
Supplement: Supplementary file 1 [file ijms-21-08063-s001.zip › Supplementary file 2_Mascot protein identification/Epididymal secretory glutathione peroxidase identification.pdf]

Mascot Search Results

Protein View

Match to: **GPX5_PIG** Score: **84** Expect: **0.00024**
Epididymal secretory glutathione peroxidase OS=Sus scrofa GN=GPX5 PE=1 SV=1

Nominal mass (M_r): **25148**; Calculated pI value: **5.70**
NCBI BLAST search of GPX5_PIG against nr
Unformatted sequence string for pasting into other applications

Taxonomy: Sus scrofa

Fixed modifications: Carbamidomethyl (C)
Variable modifications: Oxidation (M)
Cleavage by Trypsin: cuts C-term side of KR unless next residue is P
Sequence Coverage: **17%**

Matched peptides shown in **Bold Red**

1 MTVQLGAFYL FPLFMAGFVQ TNSNLEKMDC YKDVTGTIYD YDAFTLNGNE
51 HIQFKQYAGK HVLFVNVATY CGLTAQYPEL NTLQEELKPF GLVVLGFPCN
101 QFGKQEPGEN SEILLGLK**YV RPGGGYVPNF QLFEK**GDVNG EKEQKVFTFL
151 KHSCPHPSEL IGSIGYISWE PIR**VHDIRWN FEKFLVGPDG VPVMR**WVHET
201 PISTVKSDIL AYLKQFKTE

Show predicted peptides also

Sort Peptides By ◉ Residue Number ○ Increasing Mass ○ Decreasing Mass

Start - End	Observed	Mr(expt)	Mr(calc)	ppm	Miss	Sequence
119 - 135	1971.0214	1970.0141	1970.0050	5	0	K.YVRPGGGYVPNFQLFEK.G (No match)
119 - 135	1971.0214	1970.0141	1970.0050	5	0	K.YVRPGGGYVPNFQLFEK.G (Ions score 60)
174 - 183	1343.7094	1342.7021	1342.6782	18	1	R.VHDIRWNFEK.F (No match)
179 - 195	1991.0232	1990.0159	1990.0135	1	1	R.WNFEKFLVGPDGVPVMR.W (No match)
184 - 195	1286.7054	1285.6981	1285.6853	10	0	K.FLVGPDGVPVMR.W (No match)
184 - 195	1302.6960	1301.6887	1301.6802	7	0	K.FLVGPDGVPVMR.W Oxidation (M) (No match)

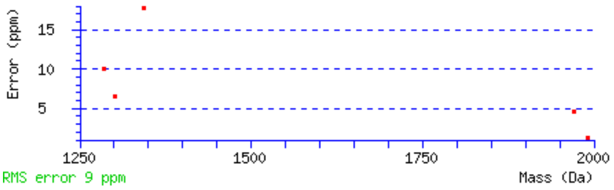

Mascot: http://www.matrixscience.com/

Mascot Search Results

User :
Email :
Search title : SampleSetID: 611, AnalysisID: 4512, MaldiWellID: 55970, SpectrumID: 109865, Path=\160212\MSMS\16-13 Jose Alvaro
Database : SwissProt sprot_160208 (550116 sequences; 196219159 residues)
Taxonomy : Mammalia (mammals) (66429 sequences)
Timestamp : 12 Feb 2016 at 11:36:54 GMT
Warning : A Peptide summary report will usually give a much clearer picture of MS/MS search results.
Top Score : 84 for GPX5_PIG, Epididymal secretory glutathione peroxidase OS=Sus scrofa GN=GPX5 PE=1 SV=1

Mascot Score Histogram

Protein score is -10*Log(P), where P is the probability that the observed match is a random event.
Protein scores greater than 61 are significant ($p<0.05$).
Protein scores are derived from ions scores as a non-probabilistic basis for ranking protein hits.

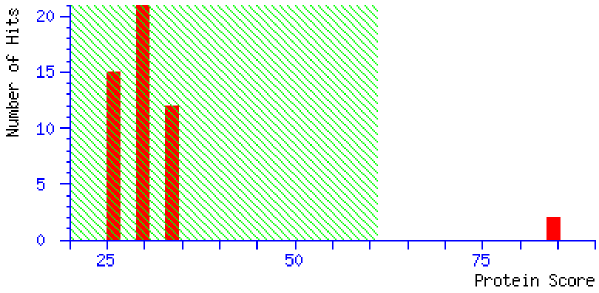

Protein Summary Report

Format As | Protein Summary (deprecated) | Help

Significance threshold p< 0.05 Max. number of hits 20

Re-Search All | Search Unmatched

Index

	Accession	Mass	Score	Description
1.	GPX5_PIG	25148	84	Epididymal secretory glutathione peroxidase OS=Sus scrofa GN=GPX5 PE=1 SV=1
2.	GPX6_MOUSE	25172	84	Glutathione peroxidase 6 OS=Mus musculus GN=Gpx6 PE=2 SV=2
3.	GCC2_RAT	195875	35	GRIP and coiled-coil domain-containing protein 2 OS=Rattus norvegicus GN=Gcc2 PE=1 SV=1
4.	OPTN_RAT	67656	35	Optineurin OS=Rattus norvegicus GN=Optn PE=1 SV=3
5.	MTPN_BOVIN	13058	35	Myotrophin OS=Bos taurus GN=MTPN PE=1 SV=3
6.	MTPN_CANLF	13058	35	Myotrophin OS=Canis lupus familiaris GN=MTPN PE=3 SV=3
7.	MTPN_HUMAN	13058	35	Myotrophin OS=Homo sapiens GN=MTPN PE=1 SV=2
8.	MTPN_MOUSE	13024	35	Myotrophin OS=Mus musculus GN=Mtpn PE=1 SV=2
9.	MTPN_RAT	13024	35	Myotrophin OS=Rattus norvegicus GN=Mtpn PE=1 SV=2
10.	SFPQ_MOUSE	75508	35	Splicing factor, proline- and glutamine-rich OS=Mus musculus GN=Sfpq PE=1 SV=1
11.	SPT17_HUMAN	43700	33	Spermatogenesis-associated protein 17 OS=Homo sapiens GN=SPATA17 PE=2 SV=1
12.	KPCT_HUMAN	83407	33	Protein kinase C theta type OS=Homo sapiens GN=PRKCQ PE=1 SV=3
13.	METL5_MOUSE	23932	33	Methyltransferase-like protein 5 OS=Mus musculus GN=Mettl5 PE=2 SV=2
14.	MTU1_HUMAN	48284	32	Mitochondrial tRNA-specific 2-thiouridylase 1 OS=Homo sapiens GN=TRMU PE=1 SV=2
15.	TFB1M_PONAB	39401	32	Dimethyladenosine transferase 1, mitochondrial OS=Pongo abelii GN=TFB1M PE=2 SV=1
16.	5NT3B_MOUSE	34688	31	7-methylguanosine phosphate-specific 5'-nucleotidase OS=Mus musculus GN=Nt5c3b PE=1 SV=3
17.	CE128_HUMAN	128565	31	Centrosomal protein of 128 kDa OS=Homo sapiens GN=CEP128 PE=1 SV=2
18.	K1KB9_MOUSE	29452	30	Kallikrein 1-related peptidase b9 OS=Mus musculus GN=Klk1b9 PE=2 SV=1
19.	RET4_HUMAN	23337	30	Retinol-binding protein 4 OS=Homo sapiens GN=RBP4 PE=1 SV=3
20.	RET4_PANTR	23337	30	Retinol-binding protein 4 OS=Pan troglodytes GN=RBP4 PE=3 SV=1

Results List

1. GPX5_PIG **Mass:** 25148 **Score:** 84 **Expect:** 0.00024 **Matches:** 6
Epididymal secretory glutathione peroxidase OS=Sus scrofa GN=GPX5 PE=1 SV=1

Observed	Mr(expt)	Mr(calc)	ppm	Start		End	Miss	Ions	Peptide
1286.7054	1285.6981	1285.6853	9.97	184	-	195	0	---	K.FLVGPDGVPVMR.W
1302.6960	1301.6887	1301.6802	6.53	184	-	195	0	---	K.FLVGPDGVPVMR.W + Oxidation (M)
1343.7094	1342.7021	1342.6782	17.8	174	-	183	1	---	R.VHDIRWNFEK.F
1971.0214	1970.0141	1970.0050	4.61	119	-	135	0	---	K.YVRPGGGYVPNFQLFEK.G
1971.0214	1970.0141	1970.0050	4.61	119	-	135	0	60	K.YVRPGGGYVPNFQLFEK.G
1991.0232	1990.0159	1990.0135	1.22	179	-	195	1	---	R.WNFEKFLVGPDGVPVMR.W

No match to: 852.2652, 856.0344, 860.0259, 861.0547, 873.0479, 877.0488, 881.2866, 888.2926, 893.0233, 897.3115, 900.2904, 905.3061, 911.3014, 928.3109, 936.2939, 942.3025, 952.3152, 961.3091, 969.3126, 1044.0745, 1107.5702, 1136.5686, 1219.6718, 1238.7053, 1265.6561, 1267.6774, 1267.6774, 1281.6871, 1283.6715, 1289.7092, 1297.6725, 1299.6655, 1300.0554, 1320.6013, 1321.6058, 1329.5953, 1331.6124, 1337.5955, 1340.6553, 1345.6250, 1356.6287, 1510.7825, 1535.8160,

1549.8221, 1577.7504, 1586.7618, 1587.7535, 1603.7034, 1604.7328, 1618.7513, 1620.7322, 1642.7328, 1985.0415, 2289.1572, 2360.1497, 2361.1541, 2361.1541, 2417.1667, 3028.4636, 3029.4534, 3153.4355, 3211.4714

2. GPX6_MOUSE **Mass:** 25172 **Score:** 84 **Expect:** 0.00025 **Matches:** 6
Glutathione peroxidase 6 OS=Mus musculus GN=Gpx6 PE=2 SV=2

Observed	Mr(expt)	Mr(calc)	ppm	Start	End	Miss	Ions	Peptide
1286.7054	1285.6981	1285.6853	9.97	186 -	197	0	---	K.FLVGPDGVPVMR.W
1302.6960	1301.6887	1301.6802	6.53	186 -	197	0	---	K.FLVGPDGVPVMR.W + Oxidation (M)
1343.7094	1342.7021	1342.6782	17.8	176 -	185	1	---	K.VHDIRWNFEK.F
1971.0214	1970.0141	1970.0050	4.61	121 -	137	0	---	K.YVRPGGGYVPNFQLFEK.G
1971.0214	1970.0141	1970.0050	4.61	121 -	137	0	60	K.YVRPGGGYVPNFQLFEK.G
1991.0232	1990.0159	1990.0135	1.22	181 -	197	1	---	R.WNFEKFLVGPDGVPVMR.W

No match to: 852.2652, 856.0344, 860.0259, 861.0547, 873.0479, 877.0488, 881.2866, 888.2926, 893.0233, 897.3115, 900.2904, 905.3061, 911.3014, 928.3109, 936.2939, 942.3025, 952.3152, 961.3091, 969.3126, 1044.0745, 1107.5702, 1136.5686, 1219.6718, 1238.7053, 1265.6561, 1267.6774, 1267.6774, 1281.6871, 1283.6715, 1289.7092, 1297.6725, 1299.6655, 1300.0554, 1320.6013, 1321.6058, 1329.5953, 1331.6124, 1337.5955, 1340.6553, 1345.6250, 1356.6287, 1510.7825, 1535.8160, 1549.8221, 1577.7504, 1586.7618, 1587.7535, 1603.7034, 1604.7328, 1618.7513, 1620.7322, 1642.7328, 1985.0415, 2289.1572, 2360.1497, 2361.1541, 2361.1541, 2417.1667, 3028.4636, 3029.4534, 3153.4355, 3211.4714

3. GCC2_RAT **Mass:** 195875 **Score:** 35 **Expect:** 21 **Matches:** 14
GRIP and coiled-coil domain-containing protein 2 OS=Rattus norvegicus GN=Gcc2 PE=1 SV=1

Observed	Mr(expt)	Mr(calc)	ppm	Start	End	Miss	Ions	Peptide
1136.5686	1135.5613	1135.6349	-64.82	212 -	220	0	---	K.QQEIIHLQK.V
1265.6561	1264.6488	1264.6411	6.10	678 -	687	1	---	K.IKSLYEENNR.L
1283.6715	1282.6642	1282.6881	-18.62	1448 -	1458	0	---	K.TVETLQHQLSK.V
1356.6287	1355.6214	1355.6980	-56.45	1384 -	1394	1	---	R.HNRMLQETVTK.E
1510.7825	1509.7752	1509.8878	-74.58	1075 -	1087	1	---	R.LLEAQILEVQRAK.G
1535.8160	1534.8087	1534.8429	-22.24	446 -	458	1	---	K.EKLALMFEIQGLK.E + Oxidation (M)
1586.7618	1585.7545	1585.7260	18.0	1302 -	1315	0	---	K.AEQAAVTSEFENYK.V
1587.7535	1586.7462	1586.8701	-78.09	1128 -	1140	1	---	K.QLQKTMQELELVK.K
1604.7328	1603.7255	1603.8206	-59.26	232 -	244	0	---	K.DINTFQEEIVQLR.A
1618.7513	1617.7440	1617.7746	-18.93	680 -	692	1	---	K.SLYEENNRLHSEK.V
1620.7322	1619.7249	1619.8267	-62.83	135 -	148	1	---	K.NELIAVHSEHSKEK.A
1991.0232	1990.0159	1989.9313	42.5	1 -	20	1	---	-.MEDSAQDAVTAAPSGTPKSK.L
2360.1497	2359.1424	2359.1577	-6.47	755 -	774	0	---	K.QMRPSILEDNEEEDVVTVLK.A + Oxidation (M)
2417.1667	2416.1594	2416.1072	21.6	1142 -	1161	1	---	K.DAQQTTLMNMEIADYERLMK.E + Oxidation (M)

No match to: 852.2652, 856.0344, 860.0259, 861.0547, 873.0479, 877.0488, 881.2866, 888.2926, 893.0233, 897.3115, 900.2904, 905.3061, 911.3014, 928.3109, 936.2939, 942.3025, 952.3152, 961.3091, 969.3126, 1044.0745, 1107.5702, 1219.6718, 1238.7053, 1267.6774, 1267.6774, 1281.6871, 1286.7054, 1289.7092, 1297.6725, 1299.6655, 1300.0554, 1302.6960, 1320.6013, 1321.6058, 1329.5953, 1331.6124, 1337.5955, 1340.6553, 1343.7094, 1345.6250, 1549.8221, 1577.7504, 1603.7034, 1642.7328, 1971.0214, 1971.0214, 1985.0415, 2289.1572, 2361.1541, 2361.1541, 3028.4636, 3029.4534, 3153.4355, 3211.4714

4. OPTN_RAT **Mass:** 67656 **Score:** 35 **Expect:** 21 **Matches:** 10
Optineurin OS=Rattus norvegicus GN=Optn PE=1 SV=3

Observed	Mr(expt)	Mr(calc)	ppm	Start	End	Miss	Ions	Peptide
1136.5686	1135.5613	1135.5985	-32.77	356 -	364	1	---	K.QELVYSNRK.L
1238.7053	1237.6980	1237.6302	54.8	146 -	155	0	---	R.QVEQEVEHLK.I
1299.6655	1298.6582	1298.6289	22.6	2 -	12	0	---	M.SHQPLSCLTEK.G
1331.6124	1330.6051	1330.6616	-42.45	134 -	144	0	---	R.TDLEQEVEQLK.R
1340.6553	1339.6480	1339.7208	-54.32	388 -	399	1	---	K.SRLATLQATHDK.L
1577.7504	1576.7431	1576.8130	-44.33	365 -	377	1	---	K.LELQVESMRSEIK.M + Oxidation (M)
1586.7618	1585.7545	1585.6712	52.5	215 -	228	1	---	R.TDSISMGKCTEDAR.T + Oxidation (M)
1603.7034	1602.6961	1602.7177	-13.47	517 -	529	1	---	R.QSLMEMQCRHGAR.T
1642.7328	1641.7255	1641.6954	18.3	543 -	556	0	---	R.GAEDMSWQHGQQPR.S + Oxidation (M)
1991.0232	1990.0159	1990.0041	5.95	453 -	469	1	---	K.QTIAKQEEDLETMAVLR.A + Oxidation (M)

No match to: 852.2652, 856.0344, 860.0259, 861.0547, 873.0479, 877.0488, 881.2866, 888.2926, 893.0233, 897.3115, 900.2904, 905.3061, 911.3014, 928.3109, 936.2939, 942.3025, 952.3152, 961.3091, 969.3126, 1044.0745, 1107.5702, 1219.6718, 1265.6561, 1267.6774, 1267.6774, 1281.6871, 1283.6715, 1286.7054, 1289.7092, 1297.6725, 1300.0554, 1302.6960, 1320.6013, 1321.6058, 1329.5953, 1337.5955, 1343.7094, 1345.6250, 1356.6287, 1510.7825, 1535.8160, 1549.8221, 1587.7535, 1604.7328, 1618.7513, 1620.7322, 1971.0214, 1971.0214, 1985.0415, 2289.1572, 2360.1497, 2361.1541, 2361.1541, 2417.1667, 3028.4636, 3029.4534, 3153.4355, 3211.4714

5. MTPN_BOVIN **Mass:** 13058 **Score:** 35 **Expect:** 21 **Matches:** 4
Myotrophin OS=Bos taurus GN=MTPN PE=1 SV=3

Observed	Mr(expt)	Mr(calc)	ppm	Start	End	Miss	Ions	Peptide
1265.6561	1264.6488	1264.6048	34.8	20 -	30	1	---	K.DYVAKGEDVNR.T
1302.6960	1301.6887	1301.6324	43.3	25 -	36	1	---	K.GEDVNRTLEGGR.K
1343.7094	1342.7021	1342.6050	72.4	2 -	11	1	---	M.CDKEFMWALK.N + Oxidation (M)
3028.4636	3027.4563	3027.5236	-22.23	58 -	85	1	---	K.GADINAPDKHHITPLLSAVYEGHVSCVK.L

No match to: 852.2652, 856.0344, 860.0259, 861.0547, 873.0479, 877.0488, 881.2866, 888.2926, 893.0233, 897.3115, 900.2904, 905.3061, 911.3014, 928.3109, 936.2939, 942.3025, 952.3152, 961.3091, 969.3126, 1044.0745, 1107.5702, 1136.5686, 1219.6718, 1238.7053, 1267.6774, 1267.6774, 1281.6871, 1283.6715, 1286.7054, 1289.7092, 1297.6725, 1299.6655, 1300.0554, 1320.6013, 1321.6058, 1329.5953, 1331.6124, 1337.5955, 1340.6553, 1345.6250, 1356.6287, 1510.7825, 1535.8160, 1549.8221, 1577.7504, 1586.7618, 1587.7535, 1603.7034, 1604.7328, 1618.7513, 1620.7322, 1642.7328, 1971.0214, 1971.0214, 1985.0415, 1991.0232, 2289.1572, 2360.1497, 2361.1541, 2361.1541, 2417.1667, 3029.4534, 3153.4355, 3211.4714

6. MTPN_CANLF **Mass:** 13058 **Score:** 35 **Expect:** 21 **Matches:** 4
Myotrophin OS=Canis lupus familiaris GN=MTPN PE=3 SV=3

Observed	Mr(expt)	Mr(calc)	ppm	Start	End	Miss	Ions	Peptide

1265.6561	1264.6488	1264.6048	34.8	20 -	30	1	---	K.DYVAKGEDVNR.T
1302.6960	1301.6887	1301.6324	43.3	25 -	36	1	---	K.GEDVNRTLEGGR.K
1343.7094	1342.7021	1342.6050	72.4	2 -	11	1	---	M.CDKEFMWALK.N + Oxidation (M)
3028.4636	3027.4563	3027.5236	-22.23	58 -	85	1	---	K.GADINAPDKHHITPLLSAVYEGHVSCVK.L

No match to: 852.2652, 856.0344, 860.0259, 861.0547, 873.0479, 877.0488, 881.2866, 888.2926, 893.0233, 897.3115, 900.2904, 905.3061, 911.3014, 928.3109, 936.2939, 942.3025, 952.3152, 961.3091, 969.3126, 1044.0745, 1107.5702, 1136.5686, 1219.6718, 1238.7053, 1267.6774, 1267.6774, 1281.6871, 1283.6715, 1286.7054, 1289.7092, 1297.6725, 1299.6655, 1300.0554, 1320.6013, 1321.6058, 1329.5953, 1331.6124, 1337.5955, 1340.6553, 1345.6250, 1356.6287, 1510.7825, 1535.8160, 1549.8221, 1577.7504, 1586.7618, 1587.7535, 1603.7034, 1604.7328, 1618.7513, 1620.7322, 1642.7328, 1971.0214, 1971.0214, 1985.0415, 1991.0232, 2289.1572, 2360.1497, 2361.1541, 2361.1541, 2417.1667, 3029.4534, 3153.4355, 3211.4714

7. MTPN_HUMAN **Mass:** 13058 **Score:** 35 **Expect:** 21 **Matches:** 4

Myotrophin OS=Homo sapiens GN=MTPN PE=1 SV=2

Observed	Mr(expt)	Mr(calc)	ppm	Start	End	Miss	Ions	Peptide
1265.6561	1264.6488	1264.6048	34.8	20 -	30	1	---	K.DYVAKGEDVNR.T
1302.6960	1301.6887	1301.6324	43.3	25 -	36	1	---	K.GEDVNRTLEGGR.K
1343.7094	1342.7021	1342.6050	72.4	2 -	11	1	---	M.CDKEFMWALK.N + Oxidation (M)
3028.4636	3027.4563	3027.5236	-22.23	58 -	85	1	---	K.GADINAPDKHHITPLLSAVYEGHVSCVK.L

No match to: 852.2652, 856.0344, 860.0259, 861.0547, 873.0479, 877.0488, 881.2866, 888.2926, 893.0233, 897.3115, 900.2904, 905.3061, 911.3014, 928.3109, 936.2939, 942.3025, 952.3152, 961.3091, 969.3126, 1044.0745, 1107.5702, 1136.5686, 1219.6718, 1238.7053, 1267.6774, 1267.6774, 1281.6871, 1283.6715, 1286.7054, 1289.7092, 1297.6725, 1299.6655, 1300.0554, 1320.6013, 1321.6058, 1329.5953, 1331.6124, 1337.5955, 1340.6553, 1345.6250, 1356.6287, 1510.7825, 1535.8160, 1549.8221, 1577.7504, 1586.7618, 1587.7535, 1603.7034, 1604.7328, 1618.7513, 1620.7322, 1642.7328, 1971.0214, 1971.0214, 1985.0415, 1991.0232, 2289.1572, 2360.1497, 2361.1541, 2361.1541, 2417.1667, 3029.4534, 3153.4355, 3211.4714

8. MTPN_MOUSE **Mass:** 13024 **Score:** 35 **Expect:** 21 **Matches:** 4

Myotrophin OS=Mus musculus GN=Mtpn PE=1 SV=2

Observed	Mr(expt)	Mr(calc)	ppm	Start	End	Miss	Ions	Peptide
1265.6561	1264.6488	1264.6048	34.8	20 -	30	1	---	K.DYVAKGEDVNR.T
1302.6960	1301.6887	1301.6324	43.3	25 -	36	1	---	K.GEDVNRTLEGGR.K
1343.7094	1342.7021	1342.6050	72.4	2 -	11	1	---	M.CDKEFMWALK.N + Oxidation (M)
3028.4636	3027.4563	3027.5236	-22.23	58 -	85	1	---	K.GADINAPDKHHITPLLSAVYEGHVSCVK.L

No match to: 852.2652, 856.0344, 860.0259, 861.0547, 873.0479, 877.0488, 881.2866, 888.2926, 893.0233, 897.3115, 900.2904, 905.3061, 911.3014, 928.3109, 936.2939, 942.3025, 952.3152, 961.3091, 969.3126, 1044.0745, 1107.5702, 1136.5686, 1219.6718, 1238.7053, 1267.6774, 1267.6774, 1281.6871, 1283.6715, 1286.7054, 1289.7092, 1297.6725, 1299.6655, 1300.0554, 1320.6013, 1321.6058, 1329.5953, 1331.6124, 1337.5955, 1340.6553, 1345.6250, 1356.6287, 1510.7825, 1535.8160, 1549.8221, 1577.7504, 1586.7618, 1587.7535, 1603.7034, 1604.7328, 1618.7513, 1620.7322, 1642.7328, 1971.0214, 1971.0214, 1985.0415, 1991.0232, 2289.1572, 2360.1497, 2361.1541, 2361.1541, 2417.1667, 3029.4534, 3153.4355, 3211.4714

9. MTPN_RAT **Mass:** 13024 **Score:** 35 **Expect:** 21 **Matches:** 4

Myotrophin OS=Rattus norvegicus GN=Mtpn PE=1 SV=2

Observed	Mr(expt)	Mr(calc)	ppm	Start	End	Miss	Ions	Peptide
1265.6561	1264.6488	1264.6048	34.8	20 -	30	1	---	K.DYVAKGEDVNR.T
1302.6960	1301.6887	1301.6324	43.3	25 -	36	1	---	K.GEDVNRTLEGGR.K
1343.7094	1342.7021	1342.6050	72.4	2 -	11	1	---	M.CDKEFMWALK.N + Oxidation (M)
3028.4636	3027.4563	3027.5236	-22.23	58 -	85	1	---	K.GADINAPDKHHITPLLSAVYEGHVSCVK.L

No match to: 852.2652, 856.0344, 860.0259, 861.0547, 873.0479, 877.0488, 881.2866, 888.2926, 893.0233, 897.3115, 900.2904, 905.3061, 911.3014, 928.3109, 936.2939, 942.3025, 952.3152, 961.3091, 969.3126, 1044.0745, 1107.5702, 1136.5686, 1219.6718, 1238.7053, 1267.6774, 1267.6774, 1281.6871, 1283.6715, 1286.7054, 1289.7092, 1297.6725, 1299.6655, 1300.0554, 1320.6013, 1321.6058, 1329.5953, 1331.6124, 1337.5955, 1340.6553, 1345.6250, 1356.6287, 1510.7825, 1535.8160, 1549.8221, 1577.7504, 1586.7618, 1587.7535, 1603.7034, 1604.7328, 1618.7513, 1620.7322, 1642.7328, 1971.0214, 1971.0214, 1985.0415, 1991.0232, 2289.1572, 2360.1497, 2361.1541, 2361.1541, 2417.1667, 3029.4534, 3153.4355, 3211.4714

10. SFPQ_MOUSE **Mass:** 75508 **Score:** 35 **Expect:** 22 **Matches:** 10

Splicing factor, proline- and glutamine-rich OS=Mus musculus GN=Sfpq PE=1 SV=1

Observed	Mr(expt)	Mr(calc)	ppm	Start	End	Miss	Ions	Peptide
952.3152	951.3079	951.3790	-74.67	576 -	582	0	---	R.EMEEQMR.R
1136.5686	1135.5613	1135.5081	46.9	674 -	685	0	---	R.GMGPGTPAGYGR.G + Oxidation (M)
1267.6774	1266.6701	1266.6139	44.4	33 -	44	0	---	R.SPPPGMGLNQNR.G
1267.6774	1266.6701	1266.6139	44.4	33 -	44	0	---	R.SPPPGMGLNQNR.G
1283.6715	1282.6642	1282.6088	43.2	33 -	44	0	---	R.SPPPGMGLNQNR.G + Oxidation (M)
1577.7504	1576.7431	1576.6973	29.0	541 -	552	1	---	R.MEELHSQEMQKR.K + 2 Oxidation (M)
1620.7322	1619.7249	1619.6708	33.4	586 -	598	1	---	R.EESYSRMGYMDPR.E
2360.1497	2359.1424	2359.1427	-0.10	204 -	228	0	---	K.MPGGPKPGGGPGMGAPGGHPKPPHR.G + 2 Oxidation (M)
2417.1667	2416.1594	2416.1434	6.61	238 -	259	0	---	R.QHHAPYHQQHHQGPPPGGPGPR.T
3211.4714	3210.4641	3210.4243	12.4	623 -	655	0	---	K.FPPLGGGGGIGYEANPGVPPATMSGSMMGSDMR.T + Oxidation (M)

No match to: 852.2652, 856.0344, 860.0259, 861.0547, 873.0479, 877.0488, 881.2866, 888.2926, 893.0233, 897.3115, 900.2904, 905.3061, 911.3014, 928.3109, 936.2939, 942.3025, 961.3091, 969.3126, 1044.0745, 1107.5702, 1219.6718, 1238.7053, 1265.6561, 1281.6871, 1286.7054, 1289.7092, 1297.6725, 1299.6655, 1300.0554, 1302.6960, 1320.6013, 1321.6058, 1329.5953, 1331.6124, 1337.5955, 1340.6553, 1343.7094, 1345.6250, 1356.6287, 1510.7825, 1535.8160, 1549.8221, 1586.7618, 1587.7535, 1603.7034, 1604.7328, 1618.7513, 1642.7328, 1971.0214, 1971.0214, 1985.0415, 1991.0232, 2289.1572, 2361.1541, 2361.1541, 3028.4636, 3029.4534, 3153.4355

11. SPT17_HUMAN **Mass:** 43700 **Score:** 33 **Expect:** 30 **Matches:** 9

Spermatogenesis-associated protein 17 OS=Homo sapiens GN=SPATA17 PE=2 SV=1

Observed	Mr(expt)	Mr(calc)	ppm	Start	End	Miss	Ions	Peptide
1107.5702	1106.5629	1106.5774	-13.06	65 -	72	1	---	K.WWRSFLGR.K
1136.5686	1135.5613	1135.6059	-39.29	164 -	172	1	---	R.KMHYLLSTK.Q + Oxidation (M)
1286.7054	1285.6981	1285.6383	46.6	110 -	118	0	---	K.YLFNYYYLK.E
1321.6058	1320.5985	1320.6099	-8.59	11 -	21	0	---	R.SSTVGNQYYFR.N

1510.7825	1509.7752	1509.7827	-4.96	184	- 195	1	---	R.KEPDPWELQLQK.A
1549.8221	1548.8148	1548.7606	35.0	301	- 313	1	---	K.NEKYIPSMHLSSK.Y + Oxidation (M)
1971.0214	1970.0141	1969.9971	8.63	304	- 320	1	---	K.YIPSMHLSSKYGPISYK.E
1971.0214	1970.0141	1969.9971	8.63	304	- 320	1	---	K.YIPSMHLSSKYGPISYK.E
2360.1497	2359.1424	2359.1559	-5.70	331	- 349	1	---	K.WICDKDFQTVLPSFELFSK.Y

No match to: 852.2652, 856.0344, 860.0259, 861.0547, 873.0479, 877.0488, 881.2866, 888.2926, 893.0233, 897.3115, 900.2904, 905.3061, 911.3014, 928.3109, 936.2939, 942.3025, 952.3152, 961.3091, 969.3126, 1044.0745, 1219.6718, 1238.7053, 1265.6561, 1267.6774, 1267.6774, 1281.6871, 1283.6715, 1289.7092, 1297.6725, 1299.6655, 1300.0554, 1302.6960, 1320.6013, 1329.5953, 1331.6124, 1337.5955, 1340.6553, 1343.7094, 1345.6250, 1356.6287, 1535.8160, 1577.7504, 1586.7618, 1587.7535, 1603.7034, 1604.7328, 1618.7513, 1620.7322, 1642.7328, 1985.0415, 1991.0232, 2289.1572, 2361.1541, 2361.1541, 2417.1667, 3028.4636, 3029.4534, 3153.4355, 3211.4714

12. KPCT_HUMAN **Mass:** 83407 **Score:** 33 **Expect:** 36 **Matches:** 12
Protein kinase C theta type OS=Homo sapiens GN=PRKCQ PE=1 SV=3

Observed	Mr(expt)	Mr(calc)	ppm	Start	End	Miss	Ions	Peptide
1238.7053	1237.6980	1237.6680	24.3	626	- 635	1	---	R.GDIRQHPLFR.E
1299.6655	1298.6582	1298.7445	-66.46	504	- 514	1	---	R.DLKLDNILLDK.D
1329.5953	1328.5880	1328.5642	18.0	693	- 703	0	---	R.NFSFMNPGMER.L
1337.5955	1336.5882	1336.6558	-50.53	303	- 312	1	---	R.CLRDTEQIFR.E
1345.6250	1344.6177	1344.6673	-36.89	636	- 645	1	---	R.EINWEELERK.E
1356.6287	1355.6214	1355.5282	68.8	262	- 272	0	---	K.CDACGMNVHHR.C
1642.7328	1641.7255	1641.7643	-23.63	693	- 706	1	---	R.NFSFMNPGMERLIS.-
1971.0214	1970.0141	1969.8629	76.8	130	- 145	0	---	K.DMNEFETEGFFALHQR.R
1971.0214	1970.0141	1969.8629	76.8	130	- 145	0	---	K.DMNEFETEGFFALHQR.R
1985.0415	1984.0342	1983.9538	40.6	339	- 355	1	---	K.REPQGISWESPLDEVDK.M
2361.1541	2360.1468	2360.1372	4.09	49	- 68	1	---	K.KPTMYPPWDSTFDAHINKGR.V
2361.1541	2360.1468	2360.1372	4.09	49	- 68	1	---	K.KPTMYPPWDSTFDAHINKGR.V

No match to: 852.2652, 856.0344, 860.0259, 861.0547, 873.0479, 877.0488, 881.2866, 888.2926, 893.0233, 897.3115, 900.2904, 905.3061, 911.3014, 928.3109, 936.2939, 942.3025, 952.3152, 961.3091, 969.3126, 1044.0745, 1107.5702, 1136.5686, 1219.6718, 1265.6561, 1267.6774, 1267.6774, 1281.6871, 1283.6715, 1286.7054, 1289.7092, 1297.6725, 1300.0554, 1302.6960, 1320.6013, 1321.6058, 1331.6124, 1340.6553, 1343.7094, 1510.7825, 1535.8160, 1549.8221, 1577.7504, 1586.7618, 1587.7535, 1603.7034, 1604.7328, 1618.7513, 1620.7322, 1991.0232, 2289.1572, 2360.1497, 2417.1667, 3028.4636, 3029.4534, 3153.4355, 3211.4714

13. METL5_MOUSE **Mass:** 23932 **Score:** 33 **Expect:** 36 **Matches:** 5
Methyltransferase-like protein 5 OS=Mus musculus GN=Mettl5 PE=2 SV=2

Observed	Mr(expt)	Mr(calc)	ppm	Start	End	Miss	Ions	Peptide
1238.7053	1237.6980	1237.6125	69.1	133	- 143	1	---	K.NNKGTDMAFLK.T
1286.7054	1285.6981	1285.7242	-20.24	196	- 206	1	---	K.KSVDIEVDLIR.F
1289.7092	1288.7019	1288.6663	27.6	13	- 23	0	---	R.LQEVDGFEKPK.L
1618.7513	1617.7440	1617.8661	-75.44	144	- 158	1	---	K.TALGMARTAVYSLHK.S
3153.4355	3152.4282	3152.4100	5.77	93	- 118	1	---	K.NVEEFELTNVDMIQCDVYSLSNRMSK.L + 2 Oxidation (M)

No match to: 852.2652, 856.0344, 860.0259, 861.0547, 873.0479, 877.0488, 881.2866, 888.2926, 893.0233, 897.3115, 900.2904, 905.3061, 911.3014, 928.3109, 936.2939, 942.3025, 952.3152, 961.3091, 969.3126, 1044.0745, 1107.5702, 1136.5686, 1219.6718, 1265.6561, 1267.6774, 1267.6774, 1281.6871, 1283.6715, 1297.6725, 1299.6655, 1300.0554, 1302.6960, 1320.6013, 1321.6058, 1329.5953, 1331.6124, 1337.5955, 1340.6553, 1343.7094, 1345.6250, 1356.6287, 1510.7825, 1535.8160, 1549.8221, 1577.7504, 1586.7618, 1587.7535, 1603.7034, 1604.7328, 1620.7322, 1642.7328, 1971.0214, 1971.0214, 1985.0415, 1991.0232, 2289.1572, 2360.1497, 2361.1541, 2361.1541, 2417.1667, 3028.4636, 3029.4534, 3211.4714

14. MTU1_HUMAN **Mass:** 48284 **Score:** 32 **Expect:** 44 **Matches:** 8
Mitochondrial tRNA-specific 2-thiouridylase 1 OS=Homo sapiens GN=TRMU PE=1 SV=2

Observed	Mr(expt)	Mr(calc)	ppm	Start	End	Miss	Ions	Peptide
928.3109	927.3036	927.3392	-38.40	53	- 59	0	---	K.DCEDAYR.V
1289.7092	1288.7019	1288.6776	18.9	283	- 294	1	---	K.DSVKGDVFVAPR.T
1329.5953	1328.5880	1328.5576	22.9	326	- 334	1	---	K.MMECHFRFR.H + Oxidation (M)
1331.6124	1330.6051	1330.5832	16.5	217	- 227	1	---	K.ESMGMCFIGKR.N + Oxidation (M)
1345.6250	1344.6177	1344.5526	48.5	326	- 334	1	---	K.MMECHFRFR.H + 2 Oxidation (M)
1535.8160	1534.8087	1534.7926	10.5	94	- 106	1	---	R.TPNPDIVCNKHIK.F
1549.8221	1548.8148	1548.8916	-49.55	188	- 201	1	---	R.TIFPLGGLTKEFVK.K
1587.7535	1586.7462	1586.8569	-69.74	310	- 323	0	---	R.VHWIAEEPPAALVR.D

No match to: 852.2652, 856.0344, 860.0259, 861.0547, 873.0479, 877.0488, 881.2866, 888.2926, 893.0233, 897.3115, 900.2904, 905.3061, 911.3014, 936.2939, 942.3025, 952.3152, 961.3091, 969.3126, 1044.0745, 1107.5702, 1136.5686, 1219.6718, 1238.7053, 1265.6561, 1267.6774, 1267.6774, 1281.6871, 1283.6715, 1286.7054, 1297.6725, 1299.6655, 1300.0554, 1302.6960, 1320.6013, 1321.6058, 1337.5955, 1340.6553, 1343.7094, 1356.6287, 1510.7825, 1577.7504, 1586.7618, 1603.7034, 1604.7328, 1618.7513, 1620.7322, 1642.7328, 1971.0214, 1971.0214, 1985.0415, 1991.0232, 2289.1572, 2360.1497, 2361.1541, 2361.1541, 2417.1667, 3028.4636, 3029.4534, 3153.4355, 3211.4714

15. TFB1M_PONAB **Mass:** 39401 **Score:** 32 **Expect:** 46 **Matches:** 6
Dimethyladenosine transferase 1, mitochondrial OS=Pongo abelii GN=TFB1M PE=2 SV=1

Observed	Mr(expt)	Mr(calc)	ppm	Start	End	Miss	Ions	Peptide
1136.5686	1135.5613	1135.6138	-46.25	248	- 256	0	---	K.VVQNVFQFR.R
1321.6058	1320.5985	1320.4953	78.2	333	- 343	1	---	K.NEEKEEDDAEN.-
1345.6250	1344.6177	1344.6997	-60.97	179	- 191	1	---	K.EVAERLAANTGSK.Q
1549.8221	1548.8148	1548.9140	-64.04	7	- 19	1	---	K.LSTWRLPPLPTIR.E
1985.0415	1984.0342	1984.0840	-25.11	72	- 89	1	---	R.SILNADVAELLVVEKDTR.F
2417.1667	2416.1594	2416.3519	-79.65	216	- 237	0	---	R.AFVPKPEVDVGVVHFTPLIQPK.I

No match to: 852.2652, 856.0344, 860.0259, 861.0547, 873.0479, 877.0488, 881.2866, 888.2926, 893.0233, 897.3115, 900.2904, 905.3061, 911.3014, 928.3109, 936.2939, 942.3025, 952.3152, 961.3091, 969.3126, 1044.0745, 1107.5702, 1219.6718, 1238.7053, 1265.6561, 1267.6774, 1267.6774, 1281.6871, 1283.6715, 1286.7054, 1289.7092, 1297.6725, 1299.6655,

1300.0554, 1302.6960, 1320.6013, 1329.5953, 1331.6124, 1337.5955, 1340.6553, 1343.7094, 1356.6287, 1510.7825, 1535.8160, 1577.7504, 1586.7618, 1587.7535, 1603.7034, 1604.7328, 1618.7513, 1620.7322, 1642.7328, 1971.0214, 1971.0214, 1991.0232, 2289.1572, 2360.1497, 2361.1541, 2361.1541, 3028.4636, 3029.4534, 3153.4355, 3211.4714

16. 5NT3B_MOUSE **Mass:** 34688 **Score:** 31 **Expect:** 49 **Matches:** 7

7-methylguanosine phosphate-specific 5'-nucleotidase OS=Mus musculus GN=Nt5c3b PE=1 SV=3

Observed	Mr(expt)	Mr(calc)	ppm	Start	End	Miss	Ions	Peptide
1343.7094	1342.7021	1342.7092	-5.30	275 -	286	0	---	K.DETLDVVNGLLR.H
1618.7513	1617.7440	1617.7457	-1.04	287 -	300	0	---	R.HILYQGDCVELQGS.-
1971.0214	1970.0141	1970.0983	-42.71	118 -	135	1	---	R.IQKVQIAQVVGESTAMLR.E
1971.0214	1970.0141	1970.0983	-42.71	118 -	135	1	---	R.IQKVQIAQVVGESTAMLR.E
2361.1541	2360.1468	2360.1583	-4.87	36 -	55	1	---	R.LQVISDFDMTLSRFAYNGQR.C
2361.1541	2360.1468	2360.1583	-4.87	36 -	55	1	---	R.LQVISDFDMTLSRFAYNGQR.C
3028.4636	3027.4563	3027.4259	10.0	169 -	193	1	---	R.QMKVFHPNIHIVSNYMDFSEDGFLK.G + 2 Oxidation (M)

No match to: 852.2652, 856.0344, 860.0259, 861.0547, 873.0479, 877.0488, 881.2866, 888.2926, 893.0233, 897.3115, 900.2904, 905.3061, 911.3014, 928.3109, 936.2939, 942.3025, 952.3152, 961.3091, 969.3126, 1044.0745, 1107.5702, 1136.5686, 1219.6718, 1238.7053, 1265.6561, 1267.6774, 1267.6774, 1281.6871, 1283.6715, 1286.7054, 1289.7092, 1297.6725, 1299.6655, 1300.0554, 1302.6960, 1320.6013, 1321.6058, 1329.5953, 1331.6124, 1337.5955, 1340.6553, 1345.6250, 1356.6287, 1510.7825, 1535.8160, 1549.8221, 1577.7504, 1586.7618, 1587.7535, 1603.7034, 1604.7328, 1620.7322, 1642.7328, 1985.0415, 1991.0232, 2289.1572, 2360.1497, 2417.1667, 3029.4534, 3153.4355, 3211.4714

17. CE128_HUMAN **Mass:** 128565 **Score:** 31 **Expect:** 50 **Matches:** 14

Centrosomal protein of 128 kDa OS=Homo sapiens GN=CEP128 PE=1 SV=2

Observed	Mr(expt)	Mr(calc)	ppm	Start	End	Miss	Ions	Peptide
1219.6718	1218.6645	1218.6026	50.8	224 -	232	1	---	R.LQELEREMR.T + Oxidation (M)
1265.6561	1264.6488	1264.6346	11.2	57 -	66	1	---	R.QVDQMLGRYR.E
1281.6871	1280.6798	1280.6738	4.72	17 -	27	1	---	R.LSPWAARSTHR.G
1286.7054	1285.6981	1285.6990	-0.66	639 -	650	1	---	K.DLSAIRADLANK.L
1289.7092	1288.7019	1288.7060	-3.18	628 -	638	1	---	K.AKLLEMQESIK.D
1297.6725	1296.6652	1296.6786	-10.30	727 -	737	1	---	K.SEAENHIRTLK.A
1321.6058	1320.5985	1320.6422	-33.08	178 -	188	1	---	R.LGDDFNRELSR.R
1340.6553	1339.6480	1339.6965	-36.19	890 -	899	1	---	K.NLRHQLMLCR.Q
1345.6250	1344.6177	1344.6932	-56.13	54 -	64	1	---	R.NLRQVDQMLGR.Y + Oxidation (M)
1356.6287	1355.6214	1355.6914	-51.63	890 -	899	1	---	K.NLRHQLMLCR.Q + Oxidation (M)
1604.7328	1603.7255	1603.8417	-72.43	674 -	687	1	---	K.SRDEETATIITQLK.L
1971.0214	1970.0141	1970.0619	-24.23	241 -	257	1	---	R.RQDQLGLMSLQLQEALK.K
1971.0214	1970.0141	1970.0619	-24.23	241 -	257	1	---	R.RQDQLGLMSLQLQEALK.K
2289.1572	2288.1499	2288.0644	37.4	151 -	169	0	---	R.FVQETDDMTQLHGFHQSLR.D

No match to: 852.2652, 856.0344, 860.0259, 861.0547, 873.0479, 877.0488, 881.2866, 888.2926, 893.0233, 897.3115, 900.2904, 905.3061, 911.3014, 928.3109, 936.2939, 942.3025, 952.3152, 961.3091, 969.3126, 1044.0745, 1107.5702, 1136.5686, 1238.7053, 1267.6774, 1267.6774, 1283.6715, 1299.6655, 1300.0554, 1302.6960, 1320.6013, 1329.5953, 1331.6124, 1337.5955, 1343.7094, 1510.7825, 1535.8160, 1549.8221, 1577.7504, 1586.7618, 1587.7535, 1603.7034, 1618.7513, 1620.7322, 1642.7328, 1985.0415, 1991.0232, 2360.1497, 2361.1541, 2361.1541, 2417.1667, 3028.4636, 3029.4534, 3153.4355, 3211.4714

18. K1KB9_MOUSE **Mass:** 29452 **Score:** 30 **Expect:** 62 **Matches:** 6

Kallikrein 1-related peptidase b9 OS=Mus musculus GN=Klk1b9 PE=2 SV=1

Observed	Mr(expt)	Mr(calc)	ppm	Start	End	Miss	Ions	Peptide
1343.7094	1342.7021	1342.6591	32.0	249 -	259	1	---	K.FTSWIKDTMAK.N + Oxidation (M)
1356.6287	1355.6214	1355.6735	-38.39	34 -	44	0	---	K.NSQPWHVAVYR.Y
1577.7504	1576.7431	1576.8031	-38.07	165 -	177	1	---	K.FQNAKDLQCVNLK.L
1586.7618	1585.7545	1585.7121	26.8	78 -	90	0	---	K.NNLYEEEPSAQHR.L
2361.1541	2360.1468	2360.3203	-73.48	126 -	147	0	---	R.LSKPADITDVVKPIALPTEEPK.L
2361.1541	2360.1468	2360.3203	-73.48	126 -	147	0	---	R.LSKPADITDVVKPIALPTEEPK.L

No match to: 852.2652, 856.0344, 860.0259, 861.0547, 873.0479, 877.0488, 881.2866, 888.2926, 893.0233, 897.3115, 900.2904, 905.3061, 911.3014, 928.3109, 936.2939, 942.3025, 952.3152, 961.3091, 969.3126, 1044.0745, 1107.5702, 1136.5686, 1219.6718, 1238.7053, 1265.6561, 1267.6774, 1267.6774, 1281.6871, 1283.6715, 1286.7054, 1289.7092, 1297.6725, 1299.6655, 1300.0554, 1302.6960, 1320.6013, 1321.6058, 1329.5953, 1331.6124, 1337.5955, 1340.6553, 1345.6250, 1510.7825, 1535.8160, 1549.8221, 1587.7535, 1603.7034, 1604.7328, 1618.7513, 1620.7322, 1642.7328, 1971.0214, 1971.0214, 1985.0415, 1991.0232, 2289.1572, 2360.1497, 2417.1667, 3028.4636, 3029.4534, 3153.4355, 3211.4714

19. RET4_HUMAN **Mass:** 23337 **Score:** 30 **Expect:** 68 **Matches:** 4

Retinol-binding protein 4 OS=Homo sapiens GN=RBP4 PE=1 SV=3

Observed	Mr(expt)	Mr(calc)	ppm	Start	End	Miss	Ions	Peptide
1289.7092	1288.7019	1288.6274	57.8	38 -	48	1	---	R.FSGTWYAMAKK.D
1302.6960	1301.6887	1301.6510	29.0	172 -	181	1	---	R.QRQEELCLAR.Q
3028.4636	3027.4563	3027.4019	18.0	49 -	76	0	---	K.DPEGLFLQDNIVAEFSVDETGQMSATAK.G + Oxidation (M)
3211.4714	3210.4641	3210.5292	-20.26	140 -	168	1	---	R.LLNLDGTCADSYSFVFSRDPNGLPPEAQK.I

No match to: 852.2652, 856.0344, 860.0259, 861.0547, 873.0479, 877.0488, 881.2866, 888.2926, 893.0233, 897.3115, 900.2904, 905.3061, 911.3014, 928.3109, 936.2939, 942.3025, 952.3152, 961.3091, 969.3126, 1044.0745, 1107.5702, 1136.5686, 1219.6718, 1238.7053, 1265.6561, 1267.6774, 1267.6774, 1281.6871, 1283.6715, 1286.7054, 1297.6725, 1299.6655, 1300.0554, 1320.6013, 1321.6058, 1329.5953, 1331.6124, 1337.5955, 1340.6553, 1343.7094, 1345.6250, 1356.6287, 1510.7825, 1535.8160, 1549.8221, 1577.7504, 1586.7618, 1587.7535, 1603.7034, 1604.7328, 1618.7513, 1620.7322, 1642.7328, 1971.0214, 1971.0214, 1985.0415, 1991.0232, 2289.1572, 2360.1497, 2361.1541, 2361.1541, 2417.1667, 3029.4534, 3153.4355

20. RET4_PANTR **Mass:** 23337 **Score:** 30 **Expect:** 68 **Matches:** 4

Retinol-binding protein 4 OS=Pan troglodytes GN=RBP4 PE=3 SV=1

Observed	Mr(expt)	Mr(calc)	ppm	Start	End	Miss	Ions	Peptide
1289.7092	1288.7019	1288.6274	57.8	38 -	48	1	---	R.FSGTWYAMAKK.D
1302.6960	1301.6887	1301.6510	29.0	172 -	181	1	---	R.QRQEELCLAR.Q

3028.4636	3027.4563	3027.4019	18.0	49 - 76	0	---	K.DPEGLFLQDNIVAEFSVDETGQMSATAK.G + Oxidation (M)
3211.4714	3210.4641	3210.5292	-20.26	140 - 168	1	---	R.LLNLDGTCADSYSFVFSRDPNGLPPEAQK.I

No match to: 852.2652, 856.0344, 860.0259, 861.0547, 873.0479, 877.0488, 881.2866, 888.2926, 893.0233, 897.3115, 900.2904, 905.3061, 911.3014, 928.3109, 936.2939, 942.3025, 952.3152, 961.3091, 969.3126, 1044.0745, 1107.5702, 1136.5686, 1219.6718, 1238.7053, 1265.6561, 1267.6774, 1267.6774, 1281.6871, 1283.6715, 1286.7054, 1297.6725, 1299.6655, 1300.0554, 1320.6013, 1321.6058, 1329.5953, 1331.6124, 1337.5955, 1340.6553, 1343.7094, 1345.6250, 1356.6287, 1510.7825, 1535.8160, 1549.8221, 1577.7504, 1586.7618, 1587.7535, 1603.7034, 1604.7328, 1618.7513, 1620.7322, 1642.7328, 1971.0214, 1971.0214, 1985.0415, 1991.0232, 2289.1572, 2360.1497, 2361.1541, 2361.1541, 2417.1667, 3029.4534, 3153.4355

Search Parameters

```
Type of search         : Sequence Query
Enzyme                 : Trypsin
Fixed modifications    : Carbamidomethyl (C)
Variable modifications : Oxidation (M)
Mass values            : Monoisotopic
Protein Mass           : Unrestricted
Peptide Mass Tolerance : ± 80 ppm
Fragment Mass Tolerance: ± 0.3 Da
Max Missed Cleavages   : 1
Instrument type        : MALDI-TOF-TOF
Query1 (852.2652,1+)   : <no title>
Query2 (856.0344,1+)   : <no title>
Query3 (860.0259,1+)   : <no title>
Query4 (861.0547,1+)   : <no title>
Query5 (873.0479,1+)   : <no title>
Query6 (877.0488,1+)   : <no title>
Query7 (881.2866,1+)   : <no title>
Query8 (888.2926,1+)   : <no title>
Query9 (893.0233,1+)   : <no title>
Query10 (897.3115,1+)  : <no title>
Query11 (900.2904,1+)  : <no title>
Query12 (905.3061,1+)  : <no title>
Query13 (911.3014,1+)  : <no title>
Query14 (928.3109,1+)  : <no title>
Query15 (936.2939,1+)  : <no title>
Query16 (942.3025,1+)  : <no title>
Query17 (952.3152,1+)  : <no title>
Query18 (961.3091,1+)  : <no title>
Query19 (969.3126,1+)  : <no title>
Query20 (1044.0745,1+) : <no title>
Query21 (1107.5702,1+) : <no title>
Query22 (1136.5686,1+) : <no title>
Query23 (1219.6718,1+) : <no title>
Query24 (1238.7053,1+) : <no title>
Query25 (1265.6561,1+) : <no title>
Query26 (1267.6774,1+) : <no title>
Query27 (1267.6774,1+) : MaldiWellID: 55970, SpectrumID: 109866,
Query28 (1281.6871,1+) : <no title>
Query29 (1283.6715,1+) : <no title>
Query30 (1286.7054,1+) : <no title>
Query31 (1289.7092,1+) : <no title>
Query32 (1297.6725,1+) : <no title>
Query33 (1299.6655,1+) : <no title>
Query34 (1300.0554,1+) : <no title>
Query35 (1302.6960,1+) : <no title>
Query36 (1320.6013,1+) : <no title>
Query37 (1321.6058,1+) : <no title>
Query38 (1329.5953,1+) : <no title>
Query39 (1331.6124,1+) : <no title>
Query40 (1337.5955,1+) : <no title>
Query41 (1340.6553,1+) : <no title>
Query42 (1343.7094,1+) : <no title>
Query43 (1345.6250,1+) : <no title>
Query44 (1356.6287,1+) : <no title>
Query45 (1510.7825,1+) : <no title>
Query46 (1535.8160,1+) : <no title>
Query47 (1549.8221,1+) : <no title>
Query48 (1577.7504,1+) : <no title>
Query49 (1586.7618,1+) : <no title>
Query50 (1587.7535,1+) : <no title>
Query51 (1603.7034,1+) : <no title>
Query52 (1604.7328,1+) : <no title>
Query53 (1618.7513,1+) : <no title>
Query54 (1620.7322,1+) : <no title>
Query55 (1642.7328,1+) : <no title>
Query56 (1971.0214,1+) : <no title>
Query57 (1971.0214,1+) : MaldiWellID: 55970, SpectrumID: 109867,
Query58 (1985.0415,1+) : <no title>
Query59 (1991.0232,1+) : <no title>
Query60 (2289.1572,1+) : <no title>
Query61 (2360.1497,1+) : <no title>
Query62 (2361.1541,1+) : <no title>
Query63 (2361.1541,1+) : MaldiWellID: 55970, SpectrumID: 109868,
Query64 (2417.1667,1+) : <no title>
Query65 (3028.4636,1+) : <no title>
Query66 (3029.4534,1+) : <no title>
```

```
Query67 (3153.4355,1+) : <no title>
Query68 (3211.4714,1+) : <no title>
```

**Mascot:** http://www.matrixscience.com/
