# Supplementary material for: Vasectomy and Photoperiodic Regimen Modify the Protein Profile, Hormonal Content and Antioxidant Enzymes Activity of Ram Seminal Plasma
Source: Int J Mol Sci. 2020 Oct 29;21(21):8063. doi: 10.3390/ijms21218063 (PMC7663742; doi:10.3390/ijms21218063)
Supplement: Supplementary file 1 [file ijms-21-08063-s001.zip › Supplementary file 2_Mascot protein identification/Epididymal secretory protein E1 identification.pdf]

Mascot Search Results

Protein View

Match to: **NPC2_BOVIN** Score: **135** Expect: **2.1e-009**
Epididymal secretory protein E1 OS=Bos taurus GN=NPC2 PE=1 SV=1

Nominal mass (M_r): **16972**; Calculated pI value: **8.20**
NCBI BLAST search of NPC2_BOVIN against nr
Unformatted sequence string for pasting into other applications

Taxonomy: Bos taurus

Fixed modifications: Carbamidomethyl (C)
Variable modifications: Oxidation (M)
Cleavage by Trypsin: cuts C-term side of KR unless next residue is P
Sequence Coverage: **40%**

Matched peptides shown in **Bold Red**

```
  1 MRFLTVAFLF LALSASALAE PVKFKDCGSW VGVIKEVNVS PCPTQPCKLH
 51 RGQSYSVNVT FTSNTQSQSS KAVVHGIVMG IPVPFPIPES DGCKSGIRCP
101 IEKDKTYNYV NKLPVKNEYP SIKVVVEWEL TDDKNQRFFC WQIPIEVEA
```

Show predicted peptides also

Sort Peptides By ◉ Residue Number ○ Increasing Mass ○ Decreasing Mass

Start - End	Observed	Mr(expt)	Mr(calc)	ppm	Miss	Sequence
24 - 35	1395.7354	1394.7281	1394.7017	19	1	K.FKDCGSWVGVIK.E (No match)
72 - 94	2419.2795	2418.2722	2418.2440	12	0	K.AVVHGIVMGIPVPFPIPESDGCK.S (No match)
72 - 94	2435.2712	2434.2639	2434.2389	10	0	K.AVVHGIVMGIPVPFPIPESDGCK.S Oxidation (M) (N
106 - 116	1338.7251	1337.7178	1337.7343	-12	1	K.TYNYVNKLPVK.N (No match)
124 - 137	1730.8853	1729.8780	1729.8635	8	1	K.VVVEWELTDDKNQR.F (No match)
124 - 137	1730.8853	1729.8780	1729.8635	8	1	K.VVVEWELTDDKNQR.F (Ions score 104)

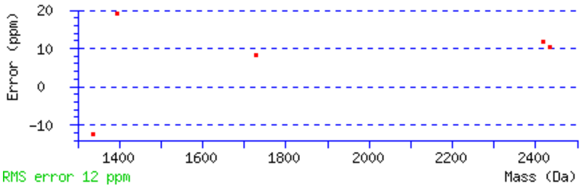

Mascot: http://www.matrixscience.com/

Mascot Search Results

User	:
Email	:
Search title	: SampleSetID: 611, AnalysisID: 4512, MaldiWellID: 55964, SpectrumID: 109844, Path=\160212\MSMS\16-13 Jose Alvaro
Database	: SwissProt sprot_160208 (550116 sequences; 196219159 residues)
Taxonomy	: Mammalia (mammals) (66429 sequences)
Timestamp	: 12 Feb 2016 at 11:36:14 GMT
Warning	: A Peptide summary report will usually give a much clearer picture of MS/MS search results.
Top Score	: 135 for NPC2_BOVIN, Epididymal secretory protein E1 OS=Bos taurus GN=NPC2 PE=1 SV=1

Mascot Score Histogram

Protein score is -10*Log(P), where P is the probability that the observed match is a random event.
Protein scores greater than 61 are significant (p<0.05).
Protein scores are derived from ions scores as a non-probabilistic basis for ranking protein hits.

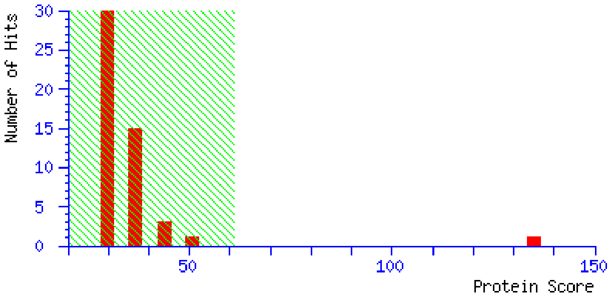

Protein Summary Report

Format As | Protein Summary (deprecated) | Help

Significance threshold p< 0.05 Max. number of hits 20

Re-Search All | Search Unmatched

Index

	Accession	Mass	Score	Description
1.	NPC2_BOVIN	16972	135	Epididymal secretory protein E1 OS=Bos taurus GN=NPC2 PE=1 SV=1
2.	TERF2_MOUSE	60397	49	Telomeric repeat-binding factor 2 OS=Mus musculus GN=Terf2 PE=1 SV=3
3.	LUZP1_HUMAN	120772	46	Leucine zipper protein 1 OS=Homo sapiens GN=LUZP1 PE=1 SV=2
4.	ATRX_MOUSE	281037	43	Transcriptional regulator ATRX OS=Mus musculus GN=Atrx PE=1 SV=3
5.	ZN594_HUMAN	96698	41	Zinc finger protein 594 OS=Homo sapiens GN=ZNF594 PE=2 SV=3
6.	TRNP1_MOUSE	23243	38	TMF-regulated nuclear protein 1 OS=Mus musculus GN=Trnp1 PE=1 SV=1
7.	K1C42_RAT	50524	38	Keratin, type I cytoskeletal 42 OS=Rattus norvegicus GN=Krt42 PE=3 SV=1
8.	IP3KB_RAT	102668	38	Inositol-trisphosphate 3-kinase B OS=Rattus norvegicus GN=Itpkb PE=1 SV=3
9.	PARG_HUMAN	112466	37	Poly(ADP-ribose) glycohydrolase OS=Homo sapiens GN=PARG PE=1 SV=1
10.	ALL1_HORSE	21910	37	Major allergen Equ c 1 OS=Equus caballus PE=1 SV=1
11.	CBR1_RABIT	30775	36	Carbonyl reductase [NADPH] 1 OS=Oryctolagus cuniculus GN=CBR1 PE=2 SV=2
12.	NRAP_MOUSE	196716	36	Nebulin-related-anchoring protein OS=Mus musculus GN=Nrap PE=1 SV=3
13.	PDLI1_MOUSE	36208	36	PDZ and LIM domain protein 1 OS=Mus musculus GN=Pdlim1 PE=1 SV=4
14.	ZNF3_HUMAN	51853	36	Zinc finger protein 3 OS=Homo sapiens GN=ZNF3 PE=1 SV=3
15.	PDLI1_RAT	36018	36	PDZ and LIM domain protein 1 OS=Rattus norvegicus GN=Pdlim1 PE=1 SV=4
16.	MPRIP_RAT	117896	35	Myosin phosphatase Rho-interacting protein OS=Rattus norvegicus GN=Mprip PE=1 SV=1
17.	AGO4_HUMAN	98175	34	Protein argonaute-4 OS=Homo sapiens GN=AGO4 PE=1 SV=2
18.	MPRIP_MOUSE	117192	34	Myosin phosphatase Rho-interacting protein OS=Mus musculus GN=Mprip PE=1 SV=2
19.	M3KL4_MOUSE	111582	34	Mitogen-activated protein kinase kinase kinase MLK4 OS=Mus musculus GN=Mlk4 PE=1 SV=2
20.	DIAC_RAT	42189	33	Di-N-acetylchitobiase OS=Rattus norvegicus GN=Ctbs PE=1 SV=1

Results List

1. NPC2_BOVIN **Mass:** 16972 **Score:** 135 **Expect:** 2.1e-009 **Matches:** 6
Epididymal secretory protein E1 OS=Bos taurus GN=NPC2 PE=1 SV=1

Observed	Mr(expt)	Mr(calc)	ppm	Start		End	Miss	Ions	Peptide
1338.7251	1337.7178	1337.7343	-12.33	106	-	116	1	---	K.TYNYVNKLPVK.N
1395.7354	1394.7281	1394.7017	19.0	24	-	35	1	---	K.FKDCGSWVGVIK.E
1730.8853	1729.8780	1729.8635	8.39	124	-	137	1	---	K.VVVEWELTDDKNQR.F
1730.8853	1729.8780	1729.8635	8.39	124	-	137	1	104	K.VVVEWELTDDKNQR.F
2419.2795	2418.2722	2418.2440	11.7	72	-	94	0	---	K.AVVHGIVMGIPVPFPIPESDGCK.S
2435.2712	2434.2639	2434.2389	10.3	72	-	94	0	---	K.AVVHGIVMGIPVPFPIPESDGCK.S + Oxidation (M)

No match to: 855.0610, 856.0469, 873.0529, 882.4213, 1044.0759, 1122.5308, 1136.5566, 1139.5569, 1139.5569, 1196.5752, 1201.4869, 1266.6499, 1290.5249, 1298.0498, 1300.0455, 1301.0454, 1320.6262, 1435.6094, 1453.5840, 1467.6008, 1510.6182, 1528.6437, 1542.6539, 1581.8621, 1585.7322, 1588.6729, 1599.6689, 1602.6768, 1638.8724, 1686.8793, 1712.9047, 1713.8983, 1719.9036, 1728.8640, 1742.8861, 1744.8925, 1776.9076, 1779.8315, 1780.8010, 1785.8284, 1787.8973, 1787.8973, 1792.8698,

1794.8204, 1801.9111, 1842.8102, 1844.9136, 1857.8766, 1893.9293, 2143.1416, 2225.1670, 2330.2119, 2371.3184, 2386.2813, 2433.3059, 2443.3013, 2449.2795, 2462.0676, 2476.3079, 2485.1541, 2499.1885, 2535.3196

2. **TERF2_MOUSE** **Mass:** 60397 **Score:** 49 **Expect:** 0.82 **Matches:** 11

Telomeric repeat-binding factor 2 OS=Mus musculus GN=Terf2 PE=1 SV=3

Observed	Mr(expt)	Mr(calc)	ppm	Start		End	Miss	Ions	Peptide
882.4213	881.4140	881.4395	-28.95	517	-	523	0	---	K.SYPFVNR.T
1338.7251	1337.7178	1337.6609	42.6	288	-	300	1	---	K.ALKSESAASSTMR.E
1395.7354	1394.7281	1394.7266	1.06	2	-	18	0	---	M.AAGAGTAGPASGPGVVR.D
1542.6539	1541.6466	1541.7620	-74.86	1	-	18	0	---	-.MAAGAGTAGPASGPGVVR.D + Oxidation (M)
1599.6689	1598.6616	1598.6703	-5.44	42	-	58	1	---	R.RSNTMAGGGGSSDSSGR.A + Oxidation (M)
1602.6768	1601.6695	1601.7243	-34.20	186	-	199	0	---	K.TEFTLTDSMVESSR.K
1730.8853	1729.8780	1729.8193	34.0	186	-	200	1	---	K.TEFTLTDSMVESSRK.L
1730.8853	1729.8780	1729.8193	34.0	186	-	200	1	---	K.TEFTLTDSMVESSRK.L
1857.8766	1856.8693	1856.9857	-62.65	314	-	330	1	---	R.EPPSRQPQNPPATIGIR.T
1893.9293	1892.9220	1892.9479	-13.69	338	-	355	1	---	K.ALSTAQDSEAAFAKLDQK.D
2419.2795	2418.2722	2418.2332	16.1	246	-	265	1	---	R.EKNLAHPVIQNFSYEVFQQK.M

No match to: 855.0610, 856.0469, 873.0529, 1044.0759, 1122.5308, 1136.5566, 1139.5569, 1139.5569, 1196.5752, 1201.4869, 1266.6499, 1290.5249, 1298.0498, 1300.0455, 1301.0454, 1320.6262, 1435.6094, 1453.5840, 1467.6008, 1510.6182, 1528.6437, 1581.8621, 1585.7322, 1588.6729, 1638.8724, 1686.8793, 1712.9047, 1713.8983, 1719.9036, 1728.8640, 1742.8861, 1744.8925, 1776.9076, 1779.8315, 1780.8010, 1785.8284, 1787.8973, 1787.8973, 1792.8698, 1794.8204, 1801.9111, 1842.8102, 1844.9136, 2143.1416, 2225.1670, 2330.2119, 2371.3184, 2386.2813, 2433.3059, 2435.2712, 2443.3013, 2449.2795, 2462.0676, 2476.3079, 2485.1541, 2499.1885, 2535.3196

3. **LUZP1_HUMAN** **Mass:** 120772 **Score:** 46 **Expect:** 1.7 **Matches:** 13

Leucine zipper protein 1 OS=Homo sapiens GN=LUZP1 PE=1 SV=2

Observed	Mr(expt)	Mr(calc)	ppm	Start		End	Miss	Ions	Peptide
1122.5308	1121.5235	1121.5353	-10.51	455	-	465	0	---	R.FVPGSSQSEGK.K
1266.6499	1265.6426	1265.6979	-43.70	188	-	198	1	---	K.SLTLSFVSERK.Y
1320.6262	1319.6189	1319.6205	-1.16	164	-	174	1	---	K.ELESSEDRLDK.T
1338.7251	1337.7178	1337.6324	63.9	227	-	237	1	---	R.DYTRNASNLER.N
1395.7354	1394.7281	1394.7154	9.15	849	-	860	0	---	K.HDITLQLAEAER.M
1542.6539	1541.6466	1541.7209	-48.20	527	-	541	1	---	R.ADKASDTSSETVFGK.R
1730.8853	1729.8780	1729.8886	-6.13	300	-	313	1	---	K.TQIKHFESLEEELK.K
1730.8853	1729.8780	1729.8886	-6.13	300	-	313	1	---	K.TQIKHFESLEEELK.K
1744.8925	1743.8852	1743.9003	-8.63	238	-	253	1	---	R.NDLRIEDGISSTLPSK.E
1779.8315	1778.8242	1778.8105	7.71	434	-	450	1	---	K.ASHMGVSTDSGTQETKK.T + Oxidation (M)
2143.1416	2142.1343	2142.0375	45.2	584	-	604	1	---	K.AANGLEADNSCPNSKAPVLSK.Y
2330.2119	2329.2046	2329.2059	-0.57	51	-	71	1	---	K.VIQAEGSNSSMLAEIEVLRQR.V
2462.0676	2461.0603	2461.2449	-74.98	792	-	815	1	---	K.VTSSITIYPSDSSSPRAAPGEALR.E

No match to: 855.0610, 856.0469, 873.0529, 882.4213, 1044.0759, 1136.5566, 1139.5569, 1139.5569, 1196.5752, 1201.4869, 1290.5249, 1298.0498, 1300.0455, 1301.0454, 1435.6094, 1453.5840, 1467.6008, 1510.6182, 1528.6437, 1581.8621, 1585.7322, 1588.6729, 1599.6689, 1602.6768, 1638.8724, 1686.8793, 1712.9047, 1713.8983, 1719.9036, 1728.8640, 1742.8861, 1776.9076, 1780.8010, 1785.8284, 1787.8973, 1787.8973, 1792.8698, 1794.8204, 1801.9111, 1842.8102, 1844.9136, 1857.8766, 1893.9293, 2225.1670, 2371.3184, 2386.2813, 2419.2795, 2433.3059, 2435.2712, 2443.3013, 2449.2795, 2476.3079, 2485.1541, 2499.1885, 2535.3196

4. **ATRX_MOUSE** **Mass:** 281037 **Score:** 43 **Expect:** 3.4 **Matches:** 13

Transcriptional regulator ATRX OS=Mus musculus GN=Atrx PE=1 SV=3

Observed	Mr(expt)	Mr(calc)	ppm	Start		End	Miss	Ions	Peptide
1122.5308	1121.5235	1121.4836	35.6	1417	-	1426	0	---	K.VQEDSSSENK.S
1139.5569	1138.5496	1138.5329	14.7	2148	-	2157	0	---	R.FLAQGTMEDK.I
1139.5569	1138.5496	1138.5329	14.7	2148	-	2157	0	---	R.FLAQGTMEDK.I
1588.6729	1587.6656	1587.7125	-29.50	1286	-	1299	1	---	K.QSEESPADDGELRR.E
1686.8793	1685.8720	1685.8083	37.8	2148	-	2161	1	---	R.FLAQGTMEDKIYDR.Q
1719.9036	1718.8963	1718.8873	5.27	2	-	17	1	---	M.TAEPMSGNKLSTLVQK.L + Oxidation (M)
1730.8853	1729.8780	1729.8118	38.3	1283	-	1298	1	---	R.IGKQSEESPADDGELR.R
1730.8853	1729.8780	1729.8118	38.3	1283	-	1298	1	29	R.IGKQSEESPADDGELR.R
1787.8973	1786.8900	1786.8229	37.5	374	-	389	1	---	K.FLKQAADNSEMTSAMK.L + Oxidation (M)
1787.8973	1786.8900	1786.8229	37.5	374	-	389	1	---	K.FLKQAADNSEMTSAMK.L + Oxidation (M)
2143.1416	2142.1343	2142.0845	23.3	123	-	141	1	---	K.GTVIVQPEPVLNEDKDDFK.G
2435.2712	2434.2639	2434.1482	47.6	1763	-	1783	1	---	R.NRFINPIQNGQCADSTMVDVR.V
2535.3196	2534.3123	2534.2886	9.35	2441	-	2464	1	---	R.GMYQSVAGGMQPPPLQRAPPPTVR.S

No match to: 855.0610, 856.0469, 873.0529, 882.4213, 1044.0759, 1136.5566, 1196.5752, 1201.4869, 1266.6499, 1290.5249, 1298.0498, 1300.0455, 1301.0454, 1320.6262, 1338.7251, 1395.7354, 1435.6094, 1453.5840, 1467.6008, 1510.6182, 1528.6437, 1542.6539, 1581.8621, 1585.7322, 1599.6689, 1602.6768, 1638.8724, 1712.9047, 1713.8983, 1728.8640, 1742.8861, 1744.8925, 1776.9076, 1779.8315, 1780.8010, 1785.8284, 1792.8698, 1794.8204, 1801.9111, 1842.8102, 1844.9136, 1857.8766, 1893.9293, 2225.1670, 2330.2119, 2371.3184, 2386.2813, 2419.2795, 2433.3059, 2443.3013, 2449.2795, 2462.0676, 2476.3079, 2485.1541, 2499.1885

5. **ZN594_HUMAN** **Mass:** 96698 **Score:** 41 **Expect:** 5.3 **Matches:** 11

Zinc finger protein 594 OS=Homo sapiens GN=ZNF594 PE=2 SV=3

Observed	Mr(expt)	Mr(calc)	ppm	Start		End	Miss	Ions	Peptide
1585.7322	1584.7249	1584.8485	-77.94	135	-	147	1	---	K.TFNRSSNLIIHQR.I
1713.8983	1712.8910	1712.7941	56.6	394	-	407	0	---	R.HQVTHTGEKPYECK.E
1730.8853	1729.8780	1729.8875	-5.48	716	-	728	1	---	K.LFMWHTAFLKHQR.L + Oxidation (M)
1730.8853	1729.8780	1729.8875	-5.48	716	-	728	1	---	K.LFMWHTAFLKHQR.L + Oxidation (M)
1776.9076	1775.9003	1775.8447	31.3	617	-	631	1	---	R.IHSGEKPYVCNKCGK.S

1780.8010	1779.7937	1779.7556	21.4	453	467	0	---	R.VHTGEKPYECSECGK.A
1794.8204	1793.8131	1793.7713	23.3	645	659	0	---	R.IHTGEKPYECSECGK.A
1842.8102	1841.8029	1841.8764	-39.88	288	302	1	---	R.IHTGEKPLKCNECEK.A
1857.8766	1856.8693	1856.8410	15.3	316	330	1	---	R.LHSGEKPYECHRCGK.T
2225.1670	2224.1597	2223.9902	76.2	642	659	1	---	K.HHRIHTGEKPYECSECGK.A
2435.2712	2434.2639	2434.1394	51.2	24	43	1	---	K.LQRQITQECELVETSNSEDR.L

No match to: 855.0610, 856.0469, 873.0529, 882.4213, 1044.0759, 1122.5308, 1136.5566, 1139.5569, 1139.5569, 1196.5752, 1201.4869, 1266.6499, 1290.5249, 1298.0498, 1300.0455, 1301.0454, 1320.6262, 1338.7251, 1395.7354, 1435.6094, 1453.5840, 1467.6008, 1510.6182, 1528.6437, 1542.6539, 1581.8621, 1588.6729, 1599.6689, 1602.6768, 1638.8724, 1686.8793, 1712.9047, 1719.9036, 1728.8640, 1742.8861, 1744.8925, 1779.8315, 1785.8284, 1787.8973, 1787.8973, 1792.8698, 1801.9111, 1844.9136, 1893.9293, 2143.1416, 2330.2119, 2371.3184, 2386.2813, 2419.2795, 2433.3059, 2443.3013, 2449.2795, 2462.0676, 2476.3079, 2485.1541, 2499.1885, 2535.3196

6. TRNP1_MOUSE **Mass:** 23243 **Score:** 38 **Expect:** 9.8 **Matches:** 6

TMF-regulated nuclear protein 1 OS=Mus musculus GN=Trnp1 PE=1 SV=1

Observed	Mr(expt)	Mr(calc)	ppm	Start	End	Miss	Ions	Peptide
1139.5569	1138.5496	1138.6207	-62.39	137	146	1	---	R.LAHRAESLSR.L
1139.5569	1138.5496	1138.6207	-62.39	137	146	1	---	R.LAHRAESLSR.L
1395.7354	1394.7281	1394.8146	-62.01	129	140	1	---	R.VFLAAELRLAHR.A
2225.1670	2224.1597	2224.0846	33.8	69	95	1	---	R.WRQGASGGSGGAGPAGIAGAAAGAGGR.A
2419.2795	2418.2722	2418.3066	-14.23	174	197	1	---	R.RGRPPALLASALGLGSCVPWGAGR.L
2443.3013	2442.2940	2442.2615	13.3	141	164	1	---	R.AESLSRLSGGVAQAELYLAAHGSR.L

No match to: 855.0610, 856.0469, 873.0529, 882.4213, 1044.0759, 1122.5308, 1136.5566, 1196.5752, 1201.4869, 1266.6499, 1290.5249, 1298.0498, 1300.0455, 1301.0454, 1320.6262, 1338.7251, 1435.6094, 1453.5840, 1467.6008, 1510.6182, 1528.6437, 1542.6539, 1581.8621, 1585.7322, 1588.6729, 1599.6689, 1602.6768, 1638.8724, 1686.8793, 1712.9047, 1713.8983, 1719.9036, 1728.8640, 1730.8853, 1730.8853, 1742.8861, 1744.8925, 1776.9076, 1779.8315, 1780.8010, 1785.8284, 1787.8973, 1787.8973, 1792.8698, 1794.8204, 1801.9111, 1842.8102, 1844.9136, 1857.8766, 1893.9293, 2143.1416, 2330.2119, 2371.3184, 2386.2813, 2433.3059, 2435.2712, 2449.2795, 2462.0676, 2476.3079, 2485.1541, 2499.1885, 2535.3196

7. K1C42_RAT **Mass:** 50524 **Score:** 38 **Expect:** 11 **Matches:** 11

Keratin, type I cytoskeletal 42 OS=Rattus norvegicus GN=Krt42 PE=3 SV=1

Observed	Mr(expt)	Mr(calc)	ppm	Start	End	Miss	Ions	Peptide
882.4213	881.4140	881.4277	-15.47	1	8	0	---	-.MASTTSIR.Q + Oxidation (M)
1122.5308	1121.5235	1121.5717	-42.91	387	395	0	---	R.LEQEIATYR.R
1136.5566	1135.5493	1135.4928	49.8	96	104	0	---	K.ETMQNLNDR.L + Oxidation (M)
1196.5752	1195.5679	1195.6422	-62.09	440	449	1	---	K.VVSSREQVHR.S
1266.6499	1265.6426	1265.6615	-14.94	181	190	1	---	R.TKYETELNLR.M
1712.9047	1711.8974	1711.7957	59.5	18	33	1	---	K.GLCAPGMGFSRMSSVR.I
1728.8640	1727.8567	1727.7906	38.3	18	33	1	---	K.GLCAPGMGFSRMSSVR.I + Oxidation (M)
1742.8861	1741.8788	1741.9111	-18.54	373	386	1	---	R.QNHEYQVLLDVKTR.L
1744.8925	1743.8852	1743.7855	57.2	18	33	1	---	K.GLCAPGMGFSRMSSVR.I + 2 Oxidation (M)
1787.8973	1786.8900	1786.8884	0.94	1	17	1	---	-.MASTTSIRQFSTSGSVK.G
1787.8973	1786.8900	1786.8884	0.94	1	17	1	---	-.MASTTSIRQFSTSGSVK.G

No match to: 855.0610, 856.0469, 873.0529, 1044.0759, 1139.5569, 1139.5569, 1201.4869, 1290.5249, 1298.0498, 1300.0455, 1301.0454, 1320.6262, 1338.7251, 1395.7354, 1435.6094, 1453.5840, 1467.6008, 1510.6182, 1528.6437, 1542.6539, 1581.8621, 1585.7322, 1588.6729, 1599.6689, 1602.6768, 1638.8724, 1686.8793, 1713.8983, 1719.9036, 1730.8853, 1730.8853, 1776.9076, 1779.8315, 1780.8010, 1785.8284, 1792.8698, 1794.8204, 1801.9111, 1842.8102, 1844.9136, 1857.8766, 1893.9293, 2143.1416, 2225.1670, 2330.2119, 2371.3184, 2386.2813, 2419.2795, 2433.3059, 2435.2712, 2443.3013, 2449.2795, 2462.0676, 2476.3079, 2485.1541, 2499.1885, 2535.3196

8. IP3KB_RAT **Mass:** 102668 **Score:** 38 **Expect:** 11 **Matches:** 11

Inositol-trisphosphate 3-kinase B OS=Rattus norvegicus GN=Itpkb PE=1 SV=3

Observed	Mr(expt)	Mr(calc)	ppm	Start	End	Miss	Ions	Peptide
1196.5752	1195.5679	1195.6125	-37.27	851	860	0	---	R.ETLEVSPFFK.C
1581.8621	1580.8548	1580.7947	38.0	402	416	1	---	R.WSRLPGDPGSVGPEK.G
1602.6768	1601.6695	1601.7130	-27.17	761	774	0	---	K.MVEVDPEAPTEEEK.A
1638.8724	1637.8651	1637.8698	-2.87	466	480	0	---	R.MLEPLPPGEVTTNLK.E
1728.8640	1727.8567	1727.8528	2.30	641	654	1	---	K.NMVQWSPFVMSFKK.K
1744.8925	1743.8852	1743.8477	21.5	641	654	1	---	K.NMVQWSPFVMSFKK.K + Oxidation (M)
1857.8766	1856.8693	1856.9317	-33.61	639	653	1	---	K.IKNMVQWSPFVMSFK.K + Oxidation (M)
1893.9293	1892.9220	1892.8071	60.7	209	224	1	---	K.SWGEQCTETPDANSRR.R
2143.1416	2142.1343	2142.1296	2.21	691	709	0	---	R.LMADVLRPFVPAYHGDVVK.D + Oxidation (M)
2386.2813	2385.2740	2385.2223	21.7	152	173	1	---	K.VGMFEAQIQAQTSAIQAPRSPR.L
2462.0676	2461.0603	2461.1914	-53.25	52	75	0	---	R.GASFLFPPAESLSLEEPGSAGGWR.S

No match to: 855.0610, 856.0469, 873.0529, 882.4213, 1044.0759, 1122.5308, 1136.5566, 1139.5569, 1139.5569, 1201.4869, 1266.6499, 1290.5249, 1298.0498, 1300.0455, 1301.0454, 1320.6262, 1338.7251, 1395.7354, 1435.6094, 1453.5840, 1467.6008, 1510.6182, 1528.6437, 1542.6539, 1585.7322, 1588.6729, 1599.6689, 1686.8793, 1712.9047, 1713.8983, 1719.9036, 1730.8853, 1730.8853, 1742.8861, 1776.9076, 1779.8315, 1780.8010, 1785.8284, 1787.8973, 1787.8973, 1792.8698, 1794.8204, 1801.9111, 1842.8102, 1844.9136, 2225.1670, 2330.2119, 2371.3184, 2419.2795, 2433.3059, 2435.2712, 2443.3013, 2449.2795, 2476.3079, 2485.1541, 2499.1885, 2535.3196

9. PARG_HUMAN **Mass:** 112466 **Score:** 37 **Expect:** 14 **Matches:** 13

Poly(ADP-ribose) glycohydrolase OS=Homo sapiens GN=PARG PE=1 SV=1

Observed	Mr(expt)	Mr(calc)	ppm	Start	End	Miss	Ions	Peptide
1139.5569	1138.5496	1138.5362	11.8	106	115	0	---	R.IESMMSSVQK.D
1139.5569	1138.5496	1138.5362	11.8	106	115	0	---	R.IESMMSSVQK.D
1338.7251	1337.7178	1337.6649	39.5	76	86	1	---	K.QKTITSWMDTK.G
1435.6094	1434.6021	1434.6415	-27.48	700	710	0	---	R.QSLEDFPEWER.C
1528.6437	1527.6364	1527.7504	-74.57	931	941	1	---	K.LLLRYYNEECR.N

Observed	Mr(expt)	Mr(calc)	ppm	Start	End	Miss	Ions	Peptide
1599.6689	1598.6616	1598.7406	-49.38	2	15	1	---	M.NAGPGCEPCTKRPR.W
1730.8853	1729.8780	1729.7811	56.1	1	15	1	---	-.MNAGPGCEPCTKRPR.W
1730.8853	1729.8780	1729.7811	56.1	1	15	1	---	-.MNAGPGCEPCTKRPR.W
1780.8010	1779.7937	1779.8032	-5.34	142	156	0	---	K.STQYLNQHQTAAMCK.W
1787.8973	1786.8900	1786.8996	-5.33	386	401	1	---	K.LPGNISSLNVECRNSK.Q
1787.8973	1786.8900	1786.9312	-23.06	126	141	1	---	K.LENVSQLSLDKSPTEK.S
1794.8204	1793.8131	1793.8027	5.80	90	105	1	---	K.TAESESLDSKENNNTR.I
2419.2795	2418.2722	2418.1631	45.1	377	398	1	---	R.TGMNDLNAKLPGNISSLNVECR.N + Oxidation (M)

No match to: 855.0610, 856.0469, 873.0529, 882.4213, 1044.0759, 1122.5308, 1136.5566, 1196.5752, 1201.4869, 1266.6499, 1290.5249, 1298.0498, 1300.0455, 1301.0454, 1320.6262, 1395.7354, 1453.5840, 1467.6008, 1510.6182, 1542.6539, 1581.8621, 1585.7322, 1588.6729, 1602.6768, 1638.8724, 1686.8793, 1712.9047, 1713.8983, 1719.9036, 1728.8640, 1742.8861, 1744.8925, 1776.9076, 1779.8315, 1785.8284, 1792.8698, 1801.9111, 1842.8102, 1844.9136, 1857.8766, 1893.9293, 2143.1416, 2225.1670, 2330.2119, 2371.3184, 2386.2813, 2433.3059, 2435.2712, 2443.3013, 2449.2795, 2462.0676, 2476.3079, 2485.1541, 2499.1885, 2535.3196

10. ALL1_HORSE **Mass:** 21910 **Score:** 37 **Expect:** 15 **Matches:** 5

Major allergen Equ c 1 OS=Equus caballus PE=1 SV=1

Observed	Mr(expt)	Mr(calc)	ppm	Start	End	Miss	Ions	Peptide
1320.6262	1319.6189	1319.6405	-16.32	176	187	1	---	R.CFQLRGNGVAQA.-
1588.6729	1587.6656	1587.7834	-74.18	130	141	0	---	K.DRPFQLFEFYAR.E
1602.6768	1601.6695	1601.7573	-54.79	65	78	0	---	R.ALDNSSLYAEYQTK.V
1779.8315	1778.8242	1778.8985	-41.76	50	64	1	---	K.IEENGSMRVFVDVIR.A + Oxidation (M)
2419.2795	2418.2722	2418.2107	25.4	27	47	1	---	R.NFDISKISGEWYSIFLASDVK.E

No match to: 855.0610, 856.0469, 873.0529, 882.4213, 1044.0759, 1122.5308, 1136.5566, 1139.5569, 1139.5569, 1196.5752, 1201.4869, 1266.6499, 1290.5249, 1298.0498, 1300.0455, 1301.0454, 1338.7251, 1395.7354, 1435.6094, 1453.5840, 1467.6008, 1510.6182, 1528.6437, 1542.6539, 1581.8621, 1585.7322, 1599.6689, 1638.8724, 1686.8793, 1712.9047, 1713.8983, 1719.9036, 1728.8640, 1730.8853, 1730.8853, 1742.8861, 1744.8925, 1776.9076, 1780.8010, 1785.8284, 1787.8973, 1787.8973, 1792.8698, 1794.8204, 1801.9111, 1842.8102, 1844.9136, 1857.8766, 1893.9293, 2143.1416, 2225.1670, 2330.2119, 2371.3184, 2386.2813, 2433.3059, 2435.2712, 2443.3013, 2449.2795, 2462.0676, 2476.3079, 2485.1541, 2499.1885, 2535.3196

11. CBR1_RABIT **Mass:** 30775 **Score:** 36 **Expect:** 15 **Matches:** 6

Carbonyl reductase [NADPH] 1 OS=Oryctolagus cuniculus GN=CBR1 PE=2 SV=2

Observed	Mr(expt)	Mr(calc)	ppm	Start	End	Miss	Ions	Peptide
1320.6262	1319.6189	1319.7132	-71.46	16	27	1	---	K.GVGFAITRALCR.L
1585.7322	1584.7249	1584.8260	-63.78	59	71	0	---	R.FHQLDITDLQSIR.A
1713.8983	1712.8910	1712.8702	12.2	120	134	0	---	R.DVCTELLPLMRPGGR.V
1801.9111	1800.9038	1800.8764	15.3	217	232	1	---	R.GGDKILVNACCPGWVR.T
1893.9293	1892.9220	1892.8648	30.2	97	112	0	---	K.MEDTTPFHIQAEVTMK.T + Oxidation (M)
2433.3059	2432.2986	2432.1399	65.3	221	242	1	---	K.ILVNACCPGWVRTDMGGPNATK.S + Oxidation (M)

No match to: 855.0610, 856.0469, 873.0529, 882.4213, 1044.0759, 1122.5308, 1136.5566, 1139.5569, 1139.5569, 1196.5752, 1201.4869, 1266.6499, 1290.5249, 1298.0498, 1300.0455, 1301.0454, 1338.7251, 1395.7354, 1435.6094, 1453.5840, 1467.6008, 1510.6182, 1528.6437, 1542.6539, 1581.8621, 1588.6729, 1599.6689, 1602.6768, 1638.8724, 1686.8793, 1712.9047, 1719.9036, 1728.8640, 1730.8853, 1730.8853, 1742.8861, 1744.8925, 1776.9076, 1779.8315, 1780.8010, 1785.8284, 1787.8973, 1787.8973, 1792.8698, 1794.8204, 1842.8102, 1844.9136, 1857.8766, 2143.1416, 2225.1670, 2330.2119, 2371.3184, 2386.2813, 2419.2795, 2435.2712, 2443.3013, 2449.2795, 2462.0676, 2476.3079, 2485.1541, 2499.1885, 2535.3196

12. NRAP_MOUSE **Mass:** 196716 **Score:** 36 **Expect:** 15 **Matches:** 17

Nebulin-related-anchoring protein OS=Mus musculus GN=Nrap PE=1 SV=3

Observed	Mr(expt)	Mr(calc)	ppm	Start	End	Miss	Ions	Peptide
882.4213	881.4140	881.4065	8.51	1168	1174	0	---	R.SDLNFMR.G
1122.5308	1121.5235	1121.4560	60.2	415	424	0	---	R.GHYDGVGMDR.R + Oxidation (M)
1320.6262	1319.6189	1319.6833	-48.81	181	192	0	---	K.AANQLASQVQYK.R
1395.7354	1394.7281	1394.6790	35.2	1125	1136	1	---	K.KAQTLASDQDYR.H
1435.6094	1434.6021	1434.6462	-30.75	262	271	1	---	R.YHQQYHREMK.G + Oxidation (M)
1585.7322	1584.7249	1584.8147	-56.67	432	446	1	---	K.VGSLASNVAYKADYK.H
1638.8724	1637.8651	1637.8083	34.7	967	980	1	---	K.FTSIKDTPEMVQAR.I + Oxidation (M)
1728.8640	1727.8567	1727.7528	60.1	269	285	1	---	R.EMKGMASPVGAEGGMTK.D + 3 Oxidation (M)
1779.8315	1778.8242	1778.9389	-64.47	235	251	1	---	R.GKGSFPAMITPAYQIAK.R
1780.8010	1779.7937	1779.8978	-58.45	304	320	1	---	R.GKGSFPAMITPAYQNAK.K
1787.8973	1786.8900	1786.9214	-17.53	1623	1638	0	---	R.QPLPQHTSDPEQLGLK.H
1787.8973	1786.8900	1786.9214	-17.53	1623	1638	0	---	R.QPLPQHTSDPEQLGLK.H
1794.8204	1793.8131	1793.9346	-67.70	483	498	1	---	R.MKFSSVTNTPQIVQAK.I + Oxidation (M)
1801.9111	1800.9038	1800.9920	-48.95	426	442	1	---	R.MLHALKVGSLASNVAYK.A
1844.9136	1843.9063	1843.9767	-38.17	1109	1124	0	---	K.AHFHLPLDMVTLVHAK.K + Oxidation (M)
2143.1416	2142.1343	2142.0568	36.2	671	688	1	---	K.AYGLQSELQYKADLAWMR.G
2462.0676	2461.0603	2461.2094	-60.56	1168	1189	1	---	R.SDLNFMRGVPCVVPGTLEIEGR.K + Oxidation (M)

No match to: 855.0610, 856.0469, 873.0529, 1044.0759, 1136.5566, 1139.5569, 1139.5569, 1196.5752, 1201.4869, 1266.6499, 1290.5249, 1298.0498, 1300.0455, 1301.0454, 1338.7251, 1453.5840, 1467.6008, 1510.6182, 1528.6437, 1542.6539, 1581.8621, 1588.6729, 1599.6689, 1602.6768, 1686.8793, 1712.9047, 1713.8983, 1719.9036, 1730.8853, 1730.8853, 1742.8861, 1744.8925, 1776.9076, 1785.8284, 1792.8698, 1842.8102, 1857.8766, 1893.9293, 2225.1670, 2330.2119, 2371.3184, 2386.2813, 2419.2795, 2433.3059, 2435.2712, 2443.3013, 2449.2795, 2476.3079, 2485.1541, 2499.1885, 2535.3196

13. PDLI1_MOUSE **Mass:** 36208 **Score:** 36 **Expect:** 17 **Matches:** 7

PDZ and LIM domain protein 1 OS=Mus musculus GN=Pdlim1 PE=1 SV=4

Observed	Mr(expt)	Mr(calc)	ppm	Start	End	Miss	Ions	Peptide
1136.5566	1135.5493	1135.6060	-49.91	261	271	0	---	K.CGTGIVGVFVK.L
1581.8621	1580.8548	1580.7651	56.8	70	83	1	---	K.IKGCADNMTLTVSR.S + Oxidation (M)
1730.8853	1729.8780	1729.8886	-6.14	84	98	1	---	R.SEQKIWSPLVTEEGK.R
1730.8853	1729.8780	1729.8886	-6.14	84	98	1	---	R.SEQKIWSPLVTEEGK.R

1744.8925	1743.8852	1743.9155	-17.39	23 -	38	1	---	K.DFEQPLAISRVTPGSK.A
1893.9293	1892.9220	1892.9731	-26.99	210 -	226	0	---	K.QSTSFLVLQEILESDGK.G
2386.2813	2385.2740	2385.2628	4.72	1 -	22	1	---	-.MTTQQIVLQGPGPWGFRLVGGK.D + Oxidation (M)

No match to: 855.0610, 856.0469, 873.0529, 882.4213, 1044.0759, 1122.5308, 1139.5569, 1139.5569, 1196.5752, 1201.4869, 1266.6499, 1290.5249, 1298.0498, 1300.0455, 1301.0454, 1320.6262, 1338.7251, 1395.7354, 1435.6094, 1453.5840, 1467.6008, 1510.6182, 1528.6437, 1542.6539, 1585.7322, 1588.6729, 1599.6689, 1602.6768, 1638.8724, 1686.8793, 1712.9047, 1713.8983, 1719.9036, 1728.8640, 1742.8861, 1776.9076, 1779.8315, 1780.8010, 1785.8284, 1787.8973, 1787.8973, 1792.8698, 1794.8204, 1801.9111, 1842.8102, 1844.9136, 1857.8766, 2143.1416, 2225.1670, 2330.2119, 2371.3184, 2419.2795, 2433.3059, 2435.2712, 2443.3013, 2449.2795, 2462.0676, 2476.3079, 2485.1541, 2499.1885, 2535.3196

14. ZNF3_HUMAN **Mass:** 51853 **Score:** 36 **Expect:** 17 **Matches:** 8

Zinc finger protein 3 OS=Homo sapiens GN=ZNF3 PE=1 SV=3

Observed	Mr(expt)	Mr(calc)	ppm	Start	End	Miss	Ions	Peptide
1395.7354	1394.7281	1394.6388	64.1	152 -	163	0	---	K.MPDFGQVTVEEK.L + Oxidation (M)
1581.8621	1580.8548	1580.8344	12.9	417 -	431	0	---	R.IHTGEKPLNGIGMSK.S
1588.6729	1587.6656	1587.7542	-55.80	348 -	360	0	---	K.AFSQSSHLYQHQR.I
1638.8724	1637.8651	1637.8849	-12.07	133 -	147	1	---	R.EVSLKRPLGNSPGER.L
1780.8010	1779.7937	1779.7556	21.4	249 -	263	0	---	R.IHTGEKPYECSDCGK.T
1787.8973	1786.8900	1786.8196	39.4	78 -	92	0	---	R.DVMLENYGNVFSLDR.E + Oxidation (M)
1787.8973	1786.8900	1786.8196	39.4	78 -	92	0	---	R.DVMLENYGNVFSLDR.E + Oxidation (M)
2225.1670	2224.1597	2224.0153	64.9	305 -	323	1	---	R.IHTGEKPYACNECGKAFSR.S

No match to: 855.0610, 856.0469, 873.0529, 882.4213, 1044.0759, 1122.5308, 1136.5566, 1139.5569, 1139.5569, 1196.5752, 1201.4869, 1266.6499, 1290.5249, 1298.0498, 1300.0455, 1301.0454, 1320.6262, 1338.7251, 1435.6094, 1453.5840, 1467.6008, 1510.6182, 1528.6437, 1542.6539, 1585.7322, 1599.6689, 1602.6768, 1686.8793, 1712.9047, 1713.8983, 1719.9036, 1728.8640, 1730.8853, 1730.8853, 1742.8861, 1744.8925, 1776.9076, 1779.8315, 1785.8284, 1792.8698, 1794.8204, 1801.9111, 1842.8102, 1844.9136, 1857.8766, 1893.9293, 2143.1416, 2330.2119, 2371.3184, 2386.2813, 2419.2795, 2433.3059, 2435.2712, 2443.3013, 2449.2795, 2462.0676, 2476.3079, 2485.1541, 2499.1885, 2535.3196

15. PDLI1_RAT **Mass:** 36018 **Score:** 36 **Expect:** 18 **Matches:** 7

PDZ and LIM domain protein 1 OS=Rattus norvegicus GN=Pdlim1 PE=1 SV=4

Observed	Mr(expt)	Mr(calc)	ppm	Start	End	Miss	Ions	Peptide
1136.5566	1135.5493	1135.6060	-49.91	261 -	271	0	---	K.CGTGIVGVFVK.L
1599.6689	1598.6616	1598.7188	-35.75	295 -	307	0	---	K.GHFFVGDQIYCEK.H
1730.8853	1729.8780	1729.8886	-6.14	84 -	98	1	---	R.SEQKIWSPLVTEEGK.R
1730.8853	1729.8780	1729.8886	-6.14	84 -	98	1	---	R.SEQKIWSPLVTEEGK.R
1744.8925	1743.8852	1743.9155	-17.39	23 -	38	1	---	K.DFEQPLAISRVTPGSK.A
1893.9293	1892.9220	1892.9731	-26.99	210 -	226	0	---	K.QSTSFLVLQEILESDGK.G
2386.2813	2385.2740	2385.2628	4.72	1 -	22	1	---	-.MTTQQIVLQGPGPWGFRLVGGK.D + Oxidation (M)

No match to: 855.0610, 856.0469, 873.0529, 882.4213, 1044.0759, 1122.5308, 1139.5569, 1139.5569, 1196.5752, 1201.4869, 1266.6499, 1290.5249, 1298.0498, 1300.0455, 1301.0454, 1320.6262, 1338.7251, 1395.7354, 1435.6094, 1453.5840, 1467.6008, 1510.6182, 1528.6437, 1542.6539, 1581.8621, 1585.7322, 1588.6729, 1602.6768, 1638.8724, 1686.8793, 1712.9047, 1713.8983, 1719.9036, 1728.8640, 1742.8861, 1776.9076, 1779.8315, 1780.8010, 1785.8284, 1787.8973, 1787.8973, 1792.8698, 1794.8204, 1801.9111, 1842.8102, 1844.9136, 1857.8766, 2143.1416, 2225.1670, 2330.2119, 2371.3184, 2419.2795, 2433.3059, 2435.2712, 2443.3013, 2449.2795, 2462.0676, 2476.3079, 2485.1541, 2499.1885, 2535.3196

16. MPRIP_RAT **Mass:** 117896 **Score:** 35 **Expect:** 21 **Matches:** 12

Myosin phosphatase Rho-interacting protein OS=Rattus norvegicus GN=Mprip PE=1 SV=1

Observed	Mr(expt)	Mr(calc)	ppm	Start	End	Miss	Ions	Peptide
1196.5752	1195.5679	1195.6349	-56.04	12 -	21	1	---	K.FQANIFNKSK.C
1266.6499	1265.6426	1265.6517	-7.15	644 -	653	0	---	R.WHQVETTPLR.E
1453.5840	1452.5767	1452.6368	-41.38	518 -	530	0	---	K.QEAEPGEPDPEQK.K
1581.8621	1580.8548	1580.7318	77.8	518 -	531	1	---	K.QEAEPGEPDPEQKK.S
1585.7322	1584.7249	1584.7341	-5.81	381 -	394	0	---	R.STESSMTPDLLNFK.K + Oxidation (M)
1638.8724	1637.8651	1637.7838	49.7	396 -	408	1	---	K.GWLTKQYEDGQWK.K
1713.8983	1712.8910	1712.8291	36.2	381 -	395	1	---	R.STESSMTPDLLNFKK.G + Oxidation (M)
1730.8853	1729.8780	1729.9210	-24.84	922 -	936	1	---	K.QEISSLKDELQTALR.D
1730.8853	1729.8780	1729.9210	-24.84	922 -	936	1	1	K.QEISSLKDELQTALR.D
1742.8861	1741.8788	1741.9210	-24.21	671 -	685	1	---	R.SERLSTHELTSLLEK.E
1794.8204	1793.8131	1793.9410	-71.30	914 -	928	1	---	K.ESEIQYLKQEISSLK.D
2371.3184	2370.3111	2370.1261	78.1	972 -	994	1	---	K.AATEALGEKSPEGTTVSGYDIMK.S + Oxidation (M)

No match to: 855.0610, 856.0469, 873.0529, 882.4213, 1044.0759, 1122.5308, 1136.5566, 1139.5569, 1139.5569, 1201.4869, 1290.5249, 1298.0498, 1300.0455, 1301.0454, 1320.6262, 1338.7251, 1395.7354, 1435.6094, 1467.6008, 1510.6182, 1528.6437, 1542.6539, 1588.6729, 1599.6689, 1602.6768, 1686.8793, 1712.9047, 1719.9036, 1728.8640, 1744.8925, 1776.9076, 1779.8315, 1780.8010, 1785.8284, 1787.8973, 1787.8973, 1792.8698, 1801.9111, 1842.8102, 1844.9136, 1857.8766, 1893.9293, 2143.1416, 2225.1670, 2330.2119, 2386.2813, 2419.2795, 2433.3059, 2435.2712, 2443.3013, 2449.2795, 2462.0676, 2476.3079, 2485.1541, 2499.1885, 2535.3196

17. AGO4_HUMAN **Mass:** 98175 **Score:** 34 **Expect:** 24 **Matches:** 12

Protein argonaute-4 OS=Homo sapiens GN=AGO4 PE=1 SV=2

Observed	Mr(expt)	Mr(calc)	ppm	Start	End	Miss	Ions	Peptide
882.4213	881.4140	881.4065	8.49	74 -	80	0	---	K.MQIFGDR.Q + Oxidation (M)
1139.5569	1138.5496	1138.5441	4.87	717 -	725	1	---	R.LFCADKTER.V
1139.5569	1138.5496	1138.5441	4.87	717 -	725	1	---	R.LFCADKTER.V
1290.5249	1289.5176	1289.6173	-77.31	103 -	114	0	---	R.VDMEVTLPGEGK.D + Oxidation (M)
1395.7354	1394.7281	1394.7075	14.8	345 -	356	1	---	K.KLTDNQTSTMIK.A + Oxidation (M)
1719.9036	1718.8963	1718.8696	15.6	527 -	542	0	---	R.VGDTLLGMATQCVQVK.N
1780.8010	1779.7937	1779.8978	-58.46	1 -	17	0	---	-.MEALGPGPPASLFQPPR.R + Oxidation (M)
1787.8973	1786.8900	1786.9611	-39.77	547 -	562	1	---	K.TSPQTLSNLCLKINAK.L
1787.8973	1786.8900	1786.9611	-39.77	547 -	562	1	---	K.TSPQTLSNLCLKINAK.L

1801.9111	1800.9038	1800.9768	-40.50	543 -	558	1	---	K.NVVKTSPQTLSNLCLK.I
1893.9293	1892.9220	1892.9190	1.60	103 -	119	1	---	R.VDMEVTLPGEGKDQTFK.V
2143.1416	2142.1343	2142.1222	5.68	42 -	58	1	---	K.IDVYHYDVDIKPEKRPR.R

No match to: 855.0610, 856.0469, 873.0529, 1044.0759, 1122.5308, 1136.5566, 1196.5752, 1201.4869, 1266.6499, 1298.0498, 1300.0455, 1301.0454, 1320.6262, 1338.7251, 1435.6094, 1453.5840, 1467.6008, 1510.6182, 1528.6437, 1542.6539, 1581.8621, 1585.7322, 1588.6729, 1599.6689, 1602.6768, 1638.8724, 1686.8793, 1712.9047, 1713.8983, 1728.8640, 1730.8853, 1730.8853, 1742.8861, 1744.8925, 1776.9076, 1779.8315, 1785.8284, 1792.8698, 1794.8204, 1842.8102, 1844.9136, 1857.8766, 2225.1670, 2330.2119, 2371.3184, 2386.2813, 2419.2795, 2433.3059, 2435.2712, 2443.3013, 2449.2795, 2462.0676, 2476.3079, 2485.1541, 2499.1885, 2535.3196

18. MPRIP_MOUSE **Mass:** 117192 **Score:** 34 **Expect:** 28 **Matches:** 12
Myosin phosphatase Rho-interacting protein OS=Mus musculus GN=Mprip PE=1 SV=2

Observed	Mr(expt)	Mr(calc)	ppm	Start	End	Miss	Ions	Peptide
1196.5752	1195.5679	1195.6349	-56.04	12 -	21	1	---	K.FQANIFNKSK.C
1266.6499	1265.6426	1265.6517	-7.15	639 -	648	0	---	R.WHQVETTPLR.E
1453.5840	1452.5767	1452.6368	-41.38	513 -	525	0	---	K.QEAEPGEPDPEQK.K
1581.8621	1580.8548	1580.7318	77.8	513 -	526	1	---	K.QEAEPGEPDPEQKK.S
1585.7322	1584.7249	1584.7341	-5.81	376 -	389	0	---	R.STESSMTPDLLNFK.K + Oxidation (M)
1638.8724	1637.8651	1637.7838	49.7	391 -	403	1	---	K.GWLTKQYEDGQWK.K
1713.8983	1712.8910	1712.8291	36.2	376 -	390	1	---	R.STESSMTPDLLNFKK.G + Oxidation (M)
1730.8853	1729.8780	1729.9210	-24.84	917 -	931	1	---	K.QEISSLKDELQTALR.D
1730.8853	1729.8780	1729.9210	-24.84	917 -	931	1	1	K.QEISSLKDELQTALR.D
1742.8861	1741.8788	1741.9210	-24.21	666 -	680	1	---	R.SERLSTHELTSLLEK.E
1794.8204	1793.8131	1793.9410	-71.30	909 -	923	1	---	K.ESEIQYLKQEISSLK.D
2371.3184	2370.3111	2370.1261	78.1	967 -	989	1	---	K.AATEALGEKSPEGTTVSGYDIMK.S + Oxidation (M)

No match to: 855.0610, 856.0469, 873.0529, 882.4213, 1044.0759, 1122.5308, 1136.5566, 1139.5569, 1139.5569, 1201.4869, 1290.5249, 1298.0498, 1300.0455, 1301.0454, 1320.6262, 1338.7251, 1395.7354, 1435.6094, 1467.6008, 1510.6182, 1528.6437, 1542.6539, 1588.6729, 1599.6689, 1602.6768, 1686.8793, 1712.9047, 1719.9036, 1728.8640, 1744.8925, 1776.9076, 1779.8315, 1780.8010, 1785.8284, 1787.8973, 1787.8973, 1792.8698, 1801.9111, 1842.8102, 1844.9136, 1857.8766, 1893.9293, 2143.1416, 2225.1670, 2330.2119, 2386.2813, 2419.2795, 2433.3059, 2435.2712, 2443.3013, 2449.2795, 2462.0676, 2476.3079, 2485.1541, 2499.1885, 2535.3196

19. M3KL4_MOUSE **Mass:** 111582 **Score:** 34 **Expect:** 28 **Matches:** 12
Mitogen-activated protein kinase kinase kinase MLK4 OS=Mus musculus GN=Mlk4 PE=1 SV=2

Observed	Mr(expt)	Mr(calc)	ppm	Start	End	Miss	Ions	Peptide
1122.5308	1121.5235	1121.6015	-69.54	157 -	165	1	---	R.EARLFAMLR.H + Oxidation (M)
1435.6094	1434.6021	1434.6926	-63.04	573 -	585	1	---	K.KGCTWGPSSVQTK.E
1585.7322	1584.7249	1584.7492	-15.30	142 -	156	1	---	R.DPEQDAAAAAESVRR.E
1638.8724	1637.8651	1637.8447	12.5	407 -	419	1	---	K.LEIQQMFSELRTK.E + Oxidation (M)
1730.8853	1729.8780	1729.8530	14.5	745 -	760	1	---	R.CQSSPSSLLRQPSAGR.A
1730.8853	1729.8780	1729.8530	14.5	745 -	760	1	---	R.CQSSPSSLLRQPSAGR.A
1785.8284	1784.8211	1784.8226	-0.80	286 -	301	0	---	R.MSAAGTYAWMAPEVIR.S + 2 Oxidation (M)
1857.8766	1856.8693	1856.8761	-3.63	520 -	537	0	---	R.SSDSGLCSPPGSPLMLPR.L
2143.1416	2142.1343	2142.0562	36.5	520 -	539	1	---	R.SSDSGLCSPPGSPLMLPRLR.A + Oxidation (M)
2419.2795	2418.2722	2418.1712	41.8	286 -	307	1	---	R.MSAAGTYAWMAPEVIRSSLFSK.G + Oxidation (M)
2435.2712	2434.2639	2434.1661	40.2	286 -	307	1	---	R.MSAAGTYAWMAPEVIRSSLFSK.G + 2 Oxidation (M)
2443.3013	2442.2940	2442.2424	21.1	761 -	785	1	---	R.APSGGSTLLLPSAPSHSSKSSLSMK.C + Oxidation (M)

No match to: 855.0610, 856.0469, 873.0529, 882.4213, 1044.0759, 1136.5566, 1139.5569, 1139.5569, 1196.5752, 1201.4869, 1266.6499, 1290.5249, 1298.0498, 1300.0455, 1301.0454, 1320.6262, 1338.7251, 1395.7354, 1453.5840, 1467.6008, 1510.6182, 1528.6437, 1542.6539, 1581.8621, 1588.6729, 1599.6689, 1602.6768, 1686.8793, 1712.9047, 1713.8983, 1719.9036, 1728.8640, 1742.8861, 1744.8925, 1776.9076, 1779.8315, 1780.8010, 1787.8973, 1787.8973, 1792.8698, 1794.8204, 1801.9111, 1842.8102, 1844.9136, 1893.9293, 2225.1670, 2330.2119, 2371.3184, 2386.2813, 2433.3059, 2449.2795, 2462.0676, 2476.3079, 2485.1541, 2499.1885, 2535.3196

20. DIAC_RAT **Mass:** 42189 **Score:** 33 **Expect:** 30 **Matches:** 8
Di-N-acetylchitobiase OS=Rattus norvegicus GN=Ctbs PE=1 SV=1

Observed	Mr(expt)	Mr(calc)	ppm	Start	End	Miss	Ions	Peptide
1585.7322	1584.7249	1584.7103	9.21	263 -	277	0	---	R.GAPCSDAAGHQVPYR.V
1602.6768	1601.6695	1601.7620	-57.73	354 -	366	0	---	R.EQTEEMWGALRPR.L
1787.8973	1786.8900	1786.7767	63.4	72 -	86	1	---	K.YDSELMCYAHSKGAR.V
1787.8973	1786.8900	1786.7767	63.4	72 -	86	1	---	K.YDSELMCYAHSKGAR.V
1792.8698	1791.8625	1791.8679	-3.02	155 -	170	0	---	R.EIEGSQVTFDVAWSPK.G
1844.9136	1843.9063	1843.9145	-4.44	42 -	56	1	---	R.DFEVFVFDVGQKTWK.S
2433.3059	2432.2986	2432.2272	29.4	231 -	250	1	---	R.KLVMGIPWYGYDYICLNLSK.D
2449.2795	2448.2722	2448.2221	20.5	231 -	250	1	---	R.KLVMGIPWYGYDYICLNLSK.D + Oxidation (M)

No match to: 855.0610, 856.0469, 873.0529, 882.4213, 1044.0759, 1122.5308, 1136.5566, 1139.5569, 1139.5569, 1196.5752, 1201.4869, 1266.6499, 1290.5249, 1298.0498, 1300.0455, 1301.0454, 1320.6262, 1338.7251, 1395.7354, 1435.6094, 1453.5840, 1467.6008, 1510.6182, 1528.6437, 1542.6539, 1581.8621, 1588.6729, 1599.6689, 1638.8724, 1686.8793, 1712.9047, 1713.8983, 1719.9036, 1728.8640, 1730.8853, 1730.8853, 1742.8861, 1744.8925, 1776.9076, 1779.8315, 1780.8010, 1785.8284, 1794.8204, 1801.9111, 1842.8102, 1857.8766, 1893.9293, 2143.1416, 2225.1670, 2330.2119, 2371.3184, 2386.2813, 2419.2795, 2435.2712, 2443.3013, 2462.0676, 2476.3079, 2485.1541, 2499.1885, 2535.3196

Search Parameters

```
Type of search         : Sequence Query
Enzyme                 : Trypsin
Fixed modifications    : Carbamidomethyl (C)
Variable modifications : Oxidation (M)
```

```
Mass values            : Monoisotopic
Protein Mass           : Unrestricted
Peptide Mass Tolerance : ± 80 ppm
Fragment Mass Tolerance: ± 0.3 Da
Max Missed Cleavages   : 1
Instrument type        : MALDI-TOF-TOF
Query1 (855.0610,1+)   : <no title>
Query2 (856.0469,1+)   : <no title>
Query3 (873.0529,1+)   : <no title>
Query4 (882.4213,1+)   : <no title>
Query5 (1044.0759,1+)  : <no title>
Query6 (1122.5308,1+)  : <no title>
Query7 (1136.5566,1+)  : <no title>
Query8 (1139.5569,1+)  : <no title>
Query9 (1139.5569,1+)  : MaldiWellID: 55964, SpectrumID: 109845,
Query10 (1196.5752,1+) : <no title>
Query11 (1201.4869,1+) : <no title>
Query12 (1266.6499,1+) : <no title>
Query13 (1290.5249,1+) : <no title>
Query14 (1298.0498,1+) : <no title>
Query15 (1300.0455,1+) : <no title>
Query16 (1301.0454,1+) : <no title>
Query17 (1320.6262,1+) : <no title>
Query18 (1338.7251,1+) : <no title>
Query19 (1395.7354,1+) : <no title>
Query20 (1435.6094,1+) : <no title>
Query21 (1453.5840,1+) : <no title>
Query22 (1467.6008,1+) : <no title>
Query23 (1510.6182,1+) : <no title>
Query24 (1528.6437,1+) : <no title>
Query25 (1542.6539,1+) : <no title>
Query26 (1581.8621,1+) : <no title>
Query27 (1585.7322,1+) : <no title>
Query28 (1588.6729,1+) : <no title>
Query29 (1599.6689,1+) : <no title>
Query30 (1602.6768,1+) : <no title>
Query31 (1638.8724,1+) : <no title>
Query32 (1686.8793,1+) : <no title>
Query33 (1712.9047,1+) : <no title>
Query34 (1713.8983,1+) : <no title>
Query35 (1719.9036,1+) : <no title>
Query36 (1728.8640,1+) : <no title>
Query37 (1730.8853,1+) : <no title>
Query38 (1730.8853,1+) : MaldiWellID: 55964, SpectrumID: 109846,
Query39 (1742.8861,1+) : <no title>
Query40 (1744.8925,1+) : <no title>
Query41 (1776.9076,1+) : <no title>
Query42 (1779.8315,1+) : <no title>
Query43 (1780.8010,1+) : <no title>
Query44 (1785.8284,1+) : <no title>
Query45 (1787.8973,1+) : <no title>
Query46 (1787.8973,1+) : MaldiWellID: 55964, SpectrumID: 109847,
Query47 (1792.8698,1+) : <no title>
Query48 (1794.8204,1+) : <no title>
Query49 (1801.9111,1+) : <no title>
Query50 (1842.8102,1+) : <no title>
Query51 (1844.9136,1+) : <no title>
Query52 (1857.8766,1+) : <no title>
Query53 (1893.9293,1+) : <no title>
Query54 (2143.1416,1+) : <no title>
Query55 (2225.1670,1+) : <no title>
Query56 (2330.2119,1+) : <no title>
Query57 (2371.3184,1+) : <no title>
Query58 (2386.2813,1+) : <no title>
Query59 (2419.2795,1+) : <no title>
Query60 (2433.3059,1+) : <no title>
Query61 (2435.2712,1+) : <no title>
Query62 (2443.3013,1+) : <no title>
Query63 (2449.2795,1+) : <no title>
Query64 (2462.0676,1+) : <no title>
Query65 (2476.3079,1+) : <no title>
Query66 (2485.1541,1+) : <no title>
Query67 (2499.1885,1+) : <no title>
Query68 (2535.3196,1+) : <no title>
```
