# Supplementary material for: Vasectomy and Photoperiodic Regimen Modify the Protein Profile, Hormonal Content and Antioxidant Enzymes Activity of Ram Seminal Plasma
Source: Int J Mol Sci. 2020 Oct 29;21(21):8063. doi: 10.3390/ijms21218063 (PMC7663742; doi:10.3390/ijms21218063)
Supplement: Supplementary file 1 [file ijms-21-08063-s001.zip › Supplementary file 2_Mascot protein identification/Inactive ribonuclease-like protein 9 identification.pdf]

Mascot Search Results

Protein View

Match to: **gi|803090389** Score: **233** Expect: **1.7e-017**
PREDICTED: inactive ribonuclease-like protein 9 [Ovis aries]

Nominal mass (M_r): **25978**; Calculated pI value: **7.53**
NCBI BLAST search of gi|803090389 against nr
Unformatted sequence string for pasting into other applications

Taxonomy: Ovis aries
Links to retrieve other entries containing this sequence from NCBI Entrez:
gi|803336779 from Ovis aries musimon

Fixed modifications: Carbamidomethyl (C)
Variable modifications: Oxidation (M)
Cleavage by Trypsin: cuts C-term side of KR unless next residue is P
Sequence Coverage: **33%**

Matched peptides shown in **Bold Red**

```
  1 MNLAKKAEWS ERENKSLVKD WISSSCCVPW TSASENMGTL INKQLLFLLF
 51 LLLKPLQFVK ITDPHLSPER RQEIEDYIND LYATGPTKPP TKETFKTRVI
101 IDSEMPLTDR EYCNLEMKMK RVHNRLYCVK EHFFLQASYD DIQKICHNMF
151 VQCKDGIRKC HRSRKIISGV HCVLTSGVMM PFCEYISSYK EGWVFITCQW
201 EDNTGEIIPV SVTDILAI
```

Show predicted peptides also

Sort Peptides By ◉ Residue Number ○ Increasing Mass ○ Decreasing Mass

Start - End	Observed	Mr(expt)	Mr(calc)	ppm	Miss	Sequence
99 - 118	2456.1501	2455.1428	2455.1433	-0	1	R.VIIDSEMPLTDREYCNLEMK.M (No match)
99 - 118	2472.1538	2471.1465	2471.1382	3	1	R.VIIDSEMPLTDREYCNLEMK.M Oxidation (M) (No m
126 - 144	2404.1614	2403.1541	2403.1569	-1	1	R.LYCVKEHFFLQASYDDIQK.I (No match)
126 - 144	2404.1614	2403.1541	2403.1569	-1	1	R.LYCVKEHFFLQASYDDIQK.I (Ions score 90)
131 - 144	1740.8196	1739.8123	1739.8155	-2	0	K.EHFFLQASYDDIQK.I (No match)
131 - 144	1740.8196	1739.8123	1739.8155	-2	0	K.EHFFLQASYDDIQK.I (Ions score 111)
145 - 154	1336.5913	1335.5840	1335.5886	-3	0	K.ICHNMFVQCK.D (No match)
166 - 190	2878.3921	2877.3848	2877.3574	10	0	K.IISGVHCVLTSGVMMPFCEYISSYK.E (No match)

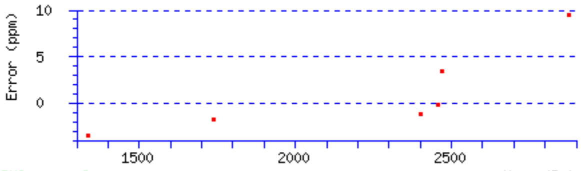

RMS error 3 ppm

Mascot: http://www.matrixscience.com/

Mascot Search Results

User	:	
Email	:	
Search title	:	SampleSetID: 611, AnalysisID: 4514, MaldiWellID: 55969, SpectrumID: 109893, Path=\160212\MSMS\16-13 Jose Alvaro NCBI
Database	:	NCBInr 160208 (79581714 sequences; 29080698065 residues)
Taxonomy	:	Mammalia (mammals) (3470019 sequences)
Timestamp	:	12 Feb 2016 at 12:49:06 GMT
Warning	:	A Peptide summary report will usually give a much clearer picture of MS/MS search results.
Top Score	:	233 for gi|803090389, PREDICTED: inactive ribonuclease-like protein 9 [Ovis aries]

Mascot Score Histogram

Protein score is -10*Log(P), where P is the probability that the observed match is a random event.
Protein scores greater than 78 are significant (p<0.05).
Protein scores are derived from ions scores as a non-probabilistic basis for ranking protein hits.

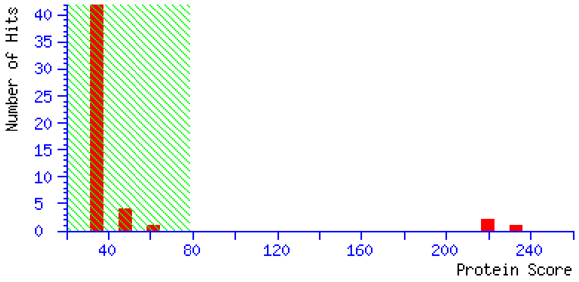

Protein Summary Report

Format As | Protein Summary (deprecated) | Help

Significance threshold p< 0.05 Max. number of hits 20

Re-Search All | Search Unmatched

Index

	Accession	Mass	Score	Description
1.	gi|803090389	25978	233	PREDICTED: inactive ribonuclease-like protein 9 [Ovis aries]
2.	gi|926702343	21755	219	PREDICTED: inactive ribonuclease-like protein 9 isoform X2 [Capra hircus]
3.	gi|926702341	25865	217	PREDICTED: inactive ribonuclease-like protein 9 isoform X1 [Capra hircus]
4.	gi|532090119	68419	55	PREDICTED: protein FAM73A [Ictidomys tridecemlineatus]
5.	gi|21779933	5241	49	myoneurin, partial [Mus musculus]
6.	gi|13938641	7157	43	Mynn protein [Mus musculus]
7.	gi|556729555	76071	42	PREDICTED: pyrin [Pantholops hodgsonii]
8.	gi|635079676	33919	41	PREDICTED: 39S ribosomal protein L19, mitochondrial [Chlorocebus sabaeus]
9.	gi|558154526	10601	41	PREDICTED: protein S100-A8 [Myotis lucifugus]
10.	gi|829979512	61098	39	PREDICTED: zinc finger and BTB domain-containing protein 18 isoform X1 [Microcebus murinus]
11.	gi|426390324	56778	39	PREDICTED: zinc finger and SCAN domain-containing protein 5C-like [Gorilla gorilla gorilla]
12.	gi|635717345	12871	39	anti-Friend virus immunoglobulin heavy chain varaible region, partial [Mus musculus]
13.	gi|674074688	39601	38	PREDICTED: proline-rich protein 19 [Nannospalax galili]
14.	gi|505838136	26619	38	PREDICTED: microtubule-associated protein RP/EB family member 1 isoform X2 [Sorex araneus]
15.	gi|157778864	13086	38	immunoglobulin heavy chain variable region [Homo sapiens]
16.	gi|670985269	51098	38	PREDICTED: fibrinogen gamma chain [Ursus maritimus]
17.	gi|4838361	13740	38	immunoglobulin heavy chain variable region [Mus musculus]
18.	gi|821101831	59974	38	PREDICTED: zinc finger and BTB domain-containing protein 18 isoform X2 [Dasypus novemcinctus]
19.	gi|831217526	13851	38	PREDICTED: peptidyl-prolyl cis-trans isomerase-like 3 [Otolemur garnettii]
20.	gi|149052108	12683	38	similar to implantation serine proteinase 2 (predicted), partial [Rattus norvegicus]

Results List

1. gi|803090389 **Mass:** 25978 **Score:** 233 **Expect:** 1.7e-017 **Matches:** 8
PREDICTED: inactive ribonuclease-like protein 9 [Ovis aries]

Observed	Mr(expt)	Mr(calc)	ppm	Start	End	Miss	Ions	Peptide
1336.5913	1335.5840	1335.5886	-3.44	145 -	154	0	---	K.ICHNMFVQCK.D
1740.8196	1739.8123	1739.8155	-1.82	131 -	144	0	---	K.EHFFLQASYDDIQK.I
1740.8196	1739.8123	1739.8155	-1.82	131 -	144	0	111	K.EHFFLQASYDDIQK.I
2404.1614	2403.1541	2403.1569	-1.16	126 -	144	1	---	R.LYCVKEHFFLQASYDDIQK.I
2404.1614	2403.1541	2403.1569	-1.16	126 -	144	1	90	R.LYCVKEHFFLQASYDDIQK.I
2456.1501	2455.1428	2455.1433	-0.19	99 -	118	1	---	R.VIIDSEMPLTDREYCNLEMK.M
2472.1538	2471.1465	2471.1382	3.37	99 -	118	1	---	R.VIIDSEMPLTDREYCNLEMK.M + Oxidation (M)
2878.3921	2877.3848	2877.3574	9.54	166 -	190	0	---	K.IISGVHCVLTSGVMMPFCEYISSYK.E

No match to: 854.0066, 855.0240, 856.0018, 858.0048, 859.2367, 859.9990, 860.2466, 871.0083, 873.0118, 886.9786, 888.2522, 892.2707, 902.2651, 918.2707, 948.2864, 951.2624, 978.2960, 981.2753, 1042.0453, 1044.0452, 1062.0494, 1066.0582, 1097.9999, 1233.0858, 1284.0266, 1298.0360, 1300.0240, 1301.0287, 1320.5753, 1547.8593, 1547.8593, 1642.7354, 1722.8113, 1739.7985, 1789.7299, 1794.7837, 2256.2166, 2289.1689, 2365.1731, 2381.1460, 2403.0527, 2408.1543, 2418.1707, 2422.1621, 2452.2048, 2454.1101, 2457.1443, 2461.1396, 2468.1174, 2470.1589, 2473.1538, 2484.1687, 2486.1538, 2487.1653, 2501.1709, 2505.1096, 2892.3655, 2909.3730, 3153.4763, 3211.4216

2. gi|926702343 **Mass:** 21755 **Score:** 219 **Expect:** 4.4e-016 **Matches:** 5

PREDICTED: inactive ribonuclease-like protein 9 isoform X2 [Capra hircus]

Observed	Mr(expt)	Mr(calc)	ppm	Start	End	Miss	Ions	Peptide
1336.5913	1335.5840	1335.5886	-3.44	109 -	118	0	---	K.ICHNMFVQCK.D
1740.8196	1739.8123	1739.8155	-1.82	95 -	108	0	---	K.EHFFLQASYDDIQK.I
1740.8196	1739.8123	1739.8155	-1.82	95 -	108	0	111	K.EHFFLQASYDDIQK.I
2404.1614	2403.1541	2403.1569	-1.16	90 -	108	1	---	R.LYCVKEHFFLQASYDDIQK.I
2404.1614	2403.1541	2403.1569	-1.16	90 -	108	1	90	R.LYCVKEHFFLQASYDDIQK.I

No match to: 854.0066, 855.0240, 856.0018, 858.0048, 859.2367, 859.9990, 860.2466, 871.0083, 873.0118, 886.9786, 888.2522, 892.2707, 902.2651, 918.2707, 948.2864, 951.2624, 978.2960, 981.2753, 1042.0453, 1044.0452, 1062.0494, 1066.0582, 1097.9999, 1233.0858, 1284.0266, 1298.0360, 1300.0240, 1301.0287, 1320.5753, 1547.8593, 1547.8593, 1642.7354, 1722.8113, 1739.7985, 1789.7299, 1794.7837, 2256.2166, 2289.1689, 2365.1731, 2381.1460, 2403.0527, 2408.1543, 2418.1707, 2422.1621, 2452.2048, 2454.1101, 2456.1501, 2457.1443, 2461.1396, 2468.1174, 2470.1589, 2472.1538, 2473.1538, 2484.1687, 2486.1538, 2487.1653, 2501.1709, 2505.1096, 2878.3921, 2892.3655, 2909.3730, 3153.4763, 3211.4216

3. gi|926702341 **Mass:** 25865 **Score:** 217 **Expect:** 6.9e-016 **Matches:** 5

PREDICTED: inactive ribonuclease-like protein 9 isoform X1 [Capra hircus]

Observed	Mr(expt)	Mr(calc)	ppm	Start	End	Miss	Ions	Peptide
1336.5913	1335.5840	1335.5886	-3.44	145 -	154	0	---	K.ICHNMFVQCK.D
1740.8196	1739.8123	1739.8155	-1.82	131 -	144	0	---	K.EHFFLQASYDDIQK.I
1740.8196	1739.8123	1739.8155	-1.82	131 -	144	0	111	K.EHFFLQASYDDIQK.I
2404.1614	2403.1541	2403.1569	-1.16	126 -	144	1	---	R.LYCVKEHFFLQASYDDIQK.I
2404.1614	2403.1541	2403.1569	-1.16	126 -	144	1	90	R.LYCVKEHFFLQASYDDIQK.I

No match to: 854.0066, 855.0240, 856.0018, 858.0048, 859.2367, 859.9990, 860.2466, 871.0083, 873.0118, 886.9786, 888.2522, 892.2707, 902.2651, 918.2707, 948.2864, 951.2624, 978.2960, 981.2753, 1042.0453, 1044.0452, 1062.0494, 1066.0582, 1097.9999, 1233.0858, 1284.0266, 1298.0360, 1300.0240, 1301.0287, 1320.5753, 1547.8593, 1547.8593, 1642.7354, 1722.8113, 1739.7985, 1789.7299, 1794.7837, 2256.2166, 2289.1689, 2365.1731, 2381.1460, 2403.0527, 2408.1543, 2418.1707, 2422.1621, 2452.2048, 2454.1101, 2456.1501, 2457.1443, 2461.1396, 2468.1174, 2470.1589, 2472.1538, 2473.1538, 2484.1687, 2486.1538, 2487.1653, 2501.1709, 2505.1096, 2878.3921, 2892.3655, 2909.3730, 3153.4763, 3211.4216

4. gi|532090119 **Mass:** 68419 **Score:** 55 **Expect:** 11 **Matches:** 10

PREDICTED: protein FAM73A [Ictidomys tridecemlineatus]

Observed	Mr(expt)	Mr(calc)	ppm	Start	End	Miss	Ions	Peptide
1320.5753	1319.5680	1319.6734	-79.89	192 -	201	1	---	R.WEQALTFRNR.Q
1722.8113	1721.8040	1721.8956	-53.20	563 -	577	1	---	R.RMELLMAYLGADALR.H
2404.1614	2403.1541	2403.1965	-17.63	578 -	600	1	---	R.HTSSCLSGHGHVISSGLLEAKVQ.-
2404.1614	2403.1541	2403.1965	-17.63	578 -	600	1	---	R.HTSSCLSGHGHVISSGLLEAKVQ.-
2408.1543	2407.1470	2407.1334	5.66	341 -	360	1	---	R.TEMLECLGDSDFLAKLHCIR.Q
2422.1621	2421.1548	2421.3056	-62.27	15 -	35	1	---	K.TAALRVFDLPLSWYNSLSQIK.F
2456.1501	2455.1428	2455.2068	-26.08	518 -	536	1	---	R.NSLYDLCCFFKNQVIFFLK.D
2892.3655	2891.3582	2891.4812	-42.55	166 -	190	0	---	K.LVNIPVTTPENLYLMGMELFEEALR.R
3153.4763	3152.4690	3152.4647	1.38	276 -	305	1	---	K.GNVDDFGLRDTSSVASTDSFASVAELAEHR.E
3211.4216	3210.4143	3210.4688	-16.97	246 -	275	1	---	R.LQEEFEATLGGSDPNSLANDTDKDTDTTVK.G

No match to: 854.0066, 855.0240, 856.0018, 858.0048, 859.2367, 859.9990, 860.2466, 871.0083, 873.0118, 886.9786, 888.2522, 892.2707, 902.2651, 918.2707, 948.2864, 951.2624, 978.2960, 981.2753, 1042.0453, 1044.0452, 1062.0494, 1066.0582, 1097.9999, 1233.0858, 1284.0266, 1298.0360, 1300.0240, 1301.0287, 1336.5913, 1547.8593, 1547.8593, 1642.7354, 1739.7985, 1740.8196, 1740.8196, 1789.7299, 1794.7837, 2256.2166, 2289.1689, 2365.1731, 2381.1460, 2403.0527, 2418.1707, 2452.2048, 2454.1101, 2457.1443, 2461.1396, 2468.1174, 2470.1589, 2472.1538, 2473.1538, 2484.1687, 2486.1538, 2487.1653, 2501.1709, 2505.1096, 2878.3921, 2909.3730

5. gi|21779933 **Mass:** 5241 **Score:** 49 **Expect:** 46 **Matches:** 4

myoneurin, partial [Mus musculus]

Observed	Mr(expt)	Mr(calc)	ppm	Start	End	Miss	Ions	Peptide
1739.7985	1738.7912	1738.7668	14.1	1 -	13	0	---	-.MQYSHHCEHLLER.L
2404.1614	2403.1541	2403.1352	7.89	17 -	36	1	---	K.QREAGFLCDCTVVIGEFQFK.A
2404.1614	2403.1541	2403.1352	7.89	17 -	36	1	12	K.QREAGFLCDCTVVIGEFQFK.A
2484.1687	2483.1614	2483.1726	-4.50	19 -	39	1	---	R.EAGFLCDCTVVIGEFQFKAHR.N

No match to: 854.0066, 855.0240, 856.0018, 858.0048, 859.2367, 859.9990, 860.2466, 871.0083, 873.0118, 886.9786, 888.2522, 892.2707, 902.2651, 918.2707, 948.2864, 951.2624, 978.2960, 981.2753, 1042.0453, 1044.0452, 1062.0494, 1066.0582, 1097.9999, 1233.0858, 1284.0266, 1298.0360, 1300.0240, 1301.0287, 1320.5753, 1336.5913, 1547.8593, 1547.8593, 1642.7354, 1722.8113, 1740.8196, 1740.8196, 1789.7299, 1794.7837, 2256.2166, 2289.1689, 2365.1731, 2381.1460, 2403.0527, 2408.1543, 2418.1707, 2422.1621, 2452.2048, 2454.1101, 2456.1501, 2457.1443, 2461.1396, 2468.1174, 2470.1589, 2472.1538, 2473.1538, 2486.1538, 2487.1653, 2501.1709, 2505.1096, 2878.3921, 2892.3655, 2909.3730, 3153.4763, 3211.4216

6. gi|13938641 **Mass:** 7157 **Score:** 43 **Expect:** 1.7e+002 **Matches:** 4

Mynn protein [Mus musculus]

Observed	Mr(expt)	Mr(calc)	ppm	Start	End	Miss	Ions	Peptide
1739.7985	1738.7912	1738.7668	14.1	1 -	13	0	---	-.MQYSHHCEHLLER.L
2404.1614	2403.1541	2403.1352	7.89	17 -	36	1	---	K.QREAGFLCDCTVVIGEFQFK.A
2404.1614	2403.1541	2403.1352	7.89	17 -	36	1	12	K.QREAGFLCDCTVVIGEFQFK.A
2484.1687	2483.1614	2483.1726	-4.50	19 -	39	1	---	R.EAGFLCDCTVVIGEFQFKAHR.N

No match to: 854.0066, 855.0240, 856.0018, 858.0048, 859.2367, 859.9990, 860.2466, 871.0083, 873.0118, 886.9786, 888.2522, 892.2707, 902.2651, 918.2707, 948.2864, 951.2624, 978.2960, 981.2753, 1042.0453, 1044.0452, 1062.0494, 1066.0582, 1097.9999, 1233.0858, 1284.0266, 1298.0360, 1300.0240, 1301.0287, 1320.5753, 1336.5913, 1547.8593, 1547.8593, 1642.7354, 1722.8113, 1740.8196, 1740.8196, 1789.7299, 1794.7837, 2256.2166, 2289.1689, 2365.1731, 2381.1460, 2403.0527, 2408.1543, 2418.1707, 2422.1621, 2452.2048, 2454.1101, 2456.1501, 2457.1443, 2461.1396, 2468.1174, 2470.1589, 2472.1538, 2473.1538, 2486.1538, 2487.1653, 2501.1709, 2505.1096, 2878.3921, 2892.3655, 2909.3730, 3153.4763, 3211.4216

7. gi|556729555 **Mass:** 76071 **Score:** 42 **Expect:** 2.3e+002 **Matches:** 8

PREDICTED: pyrin [Pantholops hodgsonii]

Observed	Mr(expt)	Mr(calc)	ppm	Start	End	Miss	Ions	Peptide
2256.2166	2255.2093	2255.0827	56.1	593 -	610	1	---	K.QCQPEWELMKDIGVTLHR.A + Oxidation (M)

2403.0527	2402.0454	2402.1657	-50.07	454	472	1	---	K.QARLLFCEDHGELICLICR.L
2422.1621	2421.1548	2421.1772	-9.24	350	372	1	---	K.ASSVPHEPSDPEVSLSSGRLQDK.A
2456.1501	2455.1428	2455.1384	1.80	96	119	1	---	R.IQEGDTDSSATSGSSGEMKPKSLK.T + Oxidation (M)
2468.1174	2467.1101	2467.2271	-47.40	4	23	1	---	R.TRSDHLLYSLEELLPYDFEK.F
2486.1538	2485.1465	2485.2417	-38.28	6	25	1	---	R.SDHLLYSLEELLPYDFEKFK.F
2501.1709	2500.1636	2500.1838	-8.09	430	450	1	---	R.EWQEGLQMASLNPKSLPQCER.H
2878.3921	2877.3848	2877.4277	-14.91	272	299	1	---	K.MNTENPSSATTASEVATLKTGPTVTLEK.G

No match to: 854.0066, 855.0240, 856.0018, 858.0048, 859.2367, 859.9990, 860.2466, 871.0083, 873.0118, 886.9786, 888.2522, 892.2707, 902.2651, 918.2707, 948.2864, 951.2624, 978.2960, 981.2753, 1042.0453, 1044.0452, 1062.0494, 1066.0582, 1097.9999, 1233.0858, 1284.0266, 1298.0360, 1300.0240, 1301.0287, 1320.5753, 1336.5913, 1547.8593, 1547.8593, 1642.7354, 1722.8113, 1739.7985, 1740.8196, 1740.8196, 1789.7299, 1794.7837, 2289.1689, 2365.1731, 2381.1460, 2404.1614, 2404.1614, 2408.1543, 2418.1707, 2452.2048, 2454.1101, 2457.1443, 2461.1396, 2470.1589, 2472.1538, 2473.1538, 2484.1687, 2487.1653, 2505.1096, 2892.3655, 2909.3730, 3153.4763, 3211.4216

8. gi|635079676 **Mass:** 33919 **Score:** 41 **Expect:** 2.7e+002 **Matches:** 7

PREDICTED: 39S ribosomal protein L19, mitochondrial [Chlorocebus sabaeus]

Observed	Mr(expt)	Mr(calc)	ppm	Start	End	Miss	Ions	Peptide
1739.7985	1738.7912	1738.8382	-27.02	242	255	1	---	K.GIRFDLCLTEEQMK.E
2289.1689	2288.1616	2288.1055	24.5	2	23	1	---	M.ASCIAAGHWAAMGLGRSFQAAR.T
2365.1731	2364.1658	2364.0191	62.1	260	277	1	---	K.WSQPWLEFDMMREYDTSK.I + Oxidation (M)
2381.1460	2380.1387	2380.0140	52.4	260	277	1	---	K.WSQPWLEFDMMREYDTSK.I + 2 Oxidation (M)
2452.2048	2451.1975	2451.1358	25.2	1	23	1	---	-.MASCIAAGHWAAMGLGRSFQAAR.T + 2 Oxidation (M)
2461.1396	2460.1323	2460.2108	-31.88	43	65	0	---	R.QHSTGPSEPGAFQPPPKPVIMDK.R + Oxidation (M)
2501.1709	2500.1636	2500.2533	-35.85	153	173	1	---	R.NVIEGQGVEICFELYNPRVHK.I

No match to: 854.0066, 855.0240, 856.0018, 858.0048, 859.2367, 859.9990, 860.2466, 871.0083, 873.0118, 886.9786, 888.2522, 892.2707, 902.2651, 918.2707, 948.2864, 951.2624, 978.2960, 981.2753, 1042.0453, 1044.0452, 1062.0494, 1066.0582, 1097.9999, 1233.0858, 1284.0266, 1298.0360, 1300.0240, 1301.0287, 1320.5753, 1336.5913, 1547.8593, 1547.8593, 1642.7354, 1722.8113, 1740.8196, 1740.8196, 1789.7299, 1794.7837, 2256.2166, 2403.0527, 2404.1614, 2404.1614, 2408.1543, 2418.1707, 2422.1621, 2454.1101, 2456.1501, 2457.1443, 2468.1174, 2470.1589, 2472.1538, 2473.1538, 2484.1687, 2486.1538, 2487.1653, 2505.1096, 2878.3921, 2892.3655, 2909.3730, 3153.4763, 3211.4216

9. gi|558154526 **Mass:** 10601 **Score:** 41 **Expect:** 3e+002 **Matches:** 4

PREDICTED: protein S100-A8 [Myotis lucifugus]

Observed	Mr(expt)	Mr(calc)	ppm	Start	End	Miss	Ions	Peptide
1642.7354	1641.7281	1641.7821	-32.86	37	49	0	---	K.LLEDECPHFLQNK.N
2256.2166	2255.2093	2255.0602	66.1	1	18	0	---	-.MLTEMENTLNNFIEIYHK.Y + Oxidation (M)
2461.1396	2460.1323	2460.1896	-23.28	37	56	1	---	K.LLEDECPHFLQNKNAATWFK.E
2487.1653	2486.1580	2486.2151	-22.96	2	21	1	---	M.LTEMENTLNNFIEIYHKYSK.L

No match to: 854.0066, 855.0240, 856.0018, 858.0048, 859.2367, 859.9990, 860.2466, 871.0083, 873.0118, 886.9786, 888.2522, 892.2707, 902.2651, 918.2707, 948.2864, 951.2624, 978.2960, 981.2753, 1042.0453, 1044.0452, 1062.0494, 1066.0582, 1097.9999, 1233.0858, 1284.0266, 1298.0360, 1300.0240, 1301.0287, 1320.5753, 1336.5913, 1547.8593, 1547.8593, 1722.8113, 1739.7985, 1740.8196, 1740.8196, 1789.7299, 1794.7837, 2289.1689, 2365.1731, 2381.1460, 2403.0527, 2404.1614, 2404.1614, 2408.1543, 2418.1707, 2422.1621, 2452.2048, 2454.1101, 2456.1501, 2457.1443, 2468.1174, 2470.1589, 2472.1538, 2473.1538, 2484.1687, 2486.1538, 2501.1709, 2505.1096, 2878.3921, 2892.3655, 2909.3730, 3153.4763, 3211.4216

10. gi|829979512 **Mass:** 61098 **Score:** 39 **Expect:** 4.1e+002 **Matches:** 7

PREDICTED: zinc finger and BTB domain-containing protein 18 isoform X1 [Microcebus murinus]

Observed	Mr(expt)	Mr(calc)	ppm	Start	End	Miss	Ions	Peptide
2289.1689	2288.1616	2288.0921	30.4	261	281	0	---	K.SSLSGVENLNSSYFSSQDVLR.S
2408.1543	2407.1470	2407.1002	19.5	3	23	0	---	K.LSLTPTPLGYEDSMEFPDHSR.H + Oxidation (M)
2452.2048	2451.1975	2451.1603	15.2	55	74	1	---	R.AVLASCSMYFHLFYKDQLDK.R + Oxidation (M)
2486.1538	2485.1465	2485.2999	-61.71	503	524	1	---	K.FHCELVNSLSVKSEALSLPTVR.D
2487.1653	2486.1580	2486.1696	-4.66	34	54	1	---	R.HQGFLCDCTVLVGDAQFRAHR.A
2505.1096	2504.1023	2503.9592	57.2	292	313	0	---	K.EASCDESDVGTNDYDMEHSTVK.E + Oxidation (M)
2892.3655	2891.3582	2891.5143	-53.97	102	126	1	---	K.LQFKDLPIEDVLAAASYLHMYDIVK.V

No match to: 854.0066, 855.0240, 856.0018, 858.0048, 859.2367, 859.9990, 860.2466, 871.0083, 873.0118, 886.9786, 888.2522, 892.2707, 902.2651, 918.2707, 948.2864, 951.2624, 978.2960, 981.2753, 1042.0453, 1044.0452, 1062.0494, 1066.0582, 1097.9999, 1233.0858, 1284.0266, 1298.0360, 1300.0240, 1301.0287, 1320.5753, 1336.5913, 1547.8593, 1547.8593, 1642.7354, 1722.8113, 1739.7985, 1740.8196, 1740.8196, 1789.7299, 1794.7837, 2256.2166, 2365.1731, 2381.1460, 2403.0527, 2404.1614, 2404.1614, 2418.1707, 2422.1621, 2454.1101, 2456.1501, 2457.1443, 2461.1396, 2468.1174, 2470.1589, 2472.1538, 2473.1538, 2484.1687, 2501.1709, 2878.3921, 2909.3730, 3153.4763, 3211.4216

11. gi|426390324 **Mass:** 56778 **Score:** 39 **Expect:** 4.3e+002 **Matches:** 7

PREDICTED: zinc finger and SCAN domain-containing protein 5C-like [Gorilla gorilla gorilla]

Observed	Mr(expt)	Mr(calc)	ppm	Start	End	Miss	Ions	Peptide
1722.8113	1721.8040	1721.7501	31.3	432	445	1	---	R.SHTGEKPFECKDCK.K
1794.7837	1793.7764	1793.8440	-37.68	49	63	1	---	R.MFSCPKESDPIQALR.K + Oxidation (M)
2381.1460	2380.1387	2380.0496	37.5	160	181	1	---	R.DVSSQRTSSVNQMCPGEGQASR.E
2456.1501	2455.1428	2455.1261	6.83	404	423	1	---	R.THTGEKPYTCDVCQKQFTQK.S
2468.1174	2467.1101	2467.2051	-38.51	298	321	1	---	K.RSKPDASSISQEGPQGEATPVGNR.E
2487.1653	2486.1580	2486.2765	-47.64	230	252	1	---	R.EENPGLTSPEPQLPNSPNLVRAK.E
3211.4216	3210.4143	3210.5577	-44.65	128	155	1	---	K.WSVVNLLGKEYLMQESDVEMAEAPVSVR.D + 2 Oxidation (M)

No match to: 854.0066, 855.0240, 856.0018, 858.0048, 859.2367, 859.9990, 860.2466, 871.0083, 873.0118, 886.9786, 888.2522, 892.2707, 902.2651, 918.2707, 948.2864, 951.2624, 978.2960, 981.2753, 1042.0453, 1044.0452, 1062.0494, 1066.0582, 1097.9999, 1233.0858, 1284.0266, 1298.0360, 1300.0240, 1301.0287, 1320.5753, 1336.5913, 1547.8593, 1547.8593, 1642.7354, 1739.7985, 1740.8196, 1740.8196, 1789.7299, 2256.2166, 2289.1689, 2365.1731, 2403.0527, 2404.1614, 2404.1614, 2408.1543, 2418.1707, 2422.1621, 2452.2048, 2454.1101, 2457.1443, 2461.1396, 2470.1589, 2472.1538, 2473.1538, 2484.1687, 2486.1538, 2501.1709, 2505.1096, 2878.3921, 2892.3655, 2909.3730, 3153.4763

12. gi|635717345 **Mass:** 12871 **Score:** 39 **Expect:** 4.6e+002 **Matches:** 4

anti-Friend virus immunoglobulin heavy chain varaible region, partial [Mus musculus]

Observed	Mr(expt)	Mr(calc)	ppm	Start	End	Miss	Ions	Peptide
1789.7299	1788.7226	1788.7818	-33.06	16	30	0	---	K.ASGYSFTGYYMNWVK.Q + Oxidation (M)

2468.1174	2467.1101	2467.0750	14.2	77 -	97	1	---	K.SLTSEDSAVYYCARGYYYGSR.D
2470.1589	2469.1516	2469.2176	-26.72	36 -	57	1	---	K.SLEWIGEINPSTGGTTYNQKFK.A
2892.3655	2891.3582	2891.2021	54.0	91 -	115	1	---	R.GYYYGSRDYYAMDYWGQGTSVTVSS.- + Oxidation (M)

No match to: 854.0066, 855.0240, 856.0018, 858.0048, 859.2367, 859.9990, 860.2466, 871.0083, 873.0118, 886.9786, 888.2522, 892.2707, 902.2651, 918.2707, 948.2864, 951.2624, 978.2960, 981.2753, 1042.0453, 1044.0452, 1062.0494, 1066.0582, 1097.9999, 1233.0858, 1284.0266, 1298.0360, 1300.0240, 1301.0287, 1320.5753, 1336.5913, 1547.8593, 1547.8593, 1642.7354, 1722.8113, 1739.7985, 1740.8196, 1740.8196, 1794.7837, 2256.2166, 2289.1689, 2365.1731, 2381.1460, 2403.0527, 2404.1614, 2404.1614, 2408.1543, 2418.1707, 2422.1621, 2452.2048, 2454.1101, 2456.1501, 2457.1443, 2461.1396, 2472.1538, 2473.1538, 2484.1687, 2486.1538, 2487.1653, 2501.1709, 2505.1096, 2878.3921, 2909.3730, 3153.4763, 3211.4216

13. gi|674074688 **Mass:** 39601 **Score:** 38 **Expect:** 5.1e+002 **Matches:** 7
PREDICTED: proline-rich protein 19 [Nannospalax galili]

Observed	Mr(expt)	Mr(calc)	ppm	Start	End	Miss	Ions	Peptide
1320.5753	1319.5680	1319.5968	-21.84	344 -	354	0	---	R.SPEAWSFPPMR.L + Oxidation (M)
1722.8113	1721.8040	1721.9107	-61.96	23 -	37	1	---	R.ERNMALVGSHRPLAR.Q + Oxidation (M)
2422.1621	2421.1548	2421.3115	-64.70	84 -	106	1	---	K.SLDVARLLNSGSLEPDTLLPPSK.S
2456.1501	2455.1428	2455.2022	-24.19	173 -	194	0	---	R.DTIMGTLQGCHGCVPDLTLVLR.G
2457.1443	2456.1370	2456.1867	-20.22	230 -	252	1	---	R.TQQGTKGLTFAMPHTSSTPSAHR.V + Oxidation (M)
2472.1538	2471.1465	2471.1971	-20.48	173 -	194	0	---	R.DTIMGTLQGCHGCVPDLTLVLR.G + Oxidation (M)
2484.1687	2483.1614	2483.2577	-38.78	90 -	113	1	---	R.LLNSGSLEPDTLLPPSKSSCSPGK.I

No match to: 854.0066, 855.0240, 856.0018, 858.0048, 859.2367, 859.9990, 860.2466, 871.0083, 873.0118, 886.9786, 888.2522, 892.2707, 902.2651, 918.2707, 948.2864, 951.2624, 978.2960, 981.2753, 1042.0453, 1044.0452, 1062.0494, 1066.0582, 1097.9999, 1233.0858, 1284.0266, 1298.0360, 1300.0240, 1301.0287, 1336.5913, 1547.8593, 1547.8593, 1642.7354, 1739.7985, 1740.8196, 1740.8196, 1789.7299, 1794.7837, 2256.2166, 2289.1689, 2365.1731, 2381.1460, 2403.0527, 2404.1614, 2404.1614, 2408.1543, 2418.1707, 2452.2048, 2454.1101, 2461.1396, 2468.1174, 2470.1589, 2473.1538, 2486.1538, 2487.1653, 2501.1709, 2505.1096, 2878.3921, 2892.3655, 2909.3730, 3153.4763, 3211.4216

14. gi|505838136 **Mass:** 26619 **Score:** 38 **Expect:** 5.1e+002 **Matches:** 5
PREDICTED: microtubule-associated protein RP/EB family member 1 isoform X2 [Sorex araneus]

Observed	Mr(expt)	Mr(calc)	ppm	Start	End	Miss	Ions	Peptide
1320.5753	1319.5680	1319.6510	-62.85	37 -	46	0	---	K.LEHEYIQNFK.I
1794.7837	1793.7764	1793.9100	-74.48	33 -	46	1	---	K.FQAKLEHEYIQNFK.I
2456.1501	2455.1428	2455.2013	-23.81	152 -	174	1	---	R.KNPGVGNGDDEAAELMQQINVLK.L + Oxidation (M)
2892.3655	2891.3582	2891.5141	-53.91	93 -	120	1	---	K.DYDPVAARQGQETAVAPSLVAPAVNKPK.K
3153.4763	3152.4690	3152.5634	-29.95	1 -	29	1	---	-.MAVNVYSTSVTSDNLSRHDMLFPGSIALK.K

No match to: 854.0066, 855.0240, 856.0018, 858.0048, 859.2367, 859.9990, 860.2466, 871.0083, 873.0118, 886.9786, 888.2522, 892.2707, 902.2651, 918.2707, 948.2864, 951.2624, 978.2960, 981.2753, 1042.0453, 1044.0452, 1062.0494, 1066.0582, 1097.9999, 1233.0858, 1284.0266, 1298.0360, 1300.0240, 1301.0287, 1336.5913, 1547.8593, 1547.8593, 1642.7354, 1722.8113, 1739.7985, 1740.8196, 1740.8196, 1789.7299, 2256.2166, 2289.1689, 2365.1731, 2381.1460, 2403.0527, 2404.1614, 2404.1614, 2408.1543, 2418.1707, 2422.1621, 2452.2048, 2454.1101, 2457.1443, 2461.1396, 2468.1174, 2470.1589, 2472.1538, 2473.1538, 2484.1687, 2486.1538, 2487.1653, 2501.1709, 2505.1096, 2878.3921, 2909.3730, 3211.4216

15. gi|157778864 **Mass:** 13086 **Score:** 38 **Expect:** 5.1e+002 **Matches:** 4
immunoglobulin heavy chain variable region [Homo sapiens]

Observed	Mr(expt)	Mr(calc)	ppm	Start	End	Miss	Ions	Peptide
1320.5753	1319.5680	1319.5452	17.3	82 -	92	0	---	R.SDDTAVYYCAR.D
2408.1543	2407.1470	2407.1359	4.60	58 -	78	1	---	K.LQGRVTMTTDTSTTTAYMELR.S + 2 Oxidation (M)
2457.1443	2456.1370	2455.9791	64.3	82 -	103	1	---	R.SDDTAVYYCARDGAVCSGSSCR.G
2892.3655	2891.3582	2891.1916	57.6	93 -	118	1	---	R.DGAVCSGSSCRGNYYYYGLDVWGQGT.-

No match to: 854.0066, 855.0240, 856.0018, 858.0048, 859.2367, 859.9990, 860.2466, 871.0083, 873.0118, 886.9786, 888.2522, 892.2707, 902.2651, 918.2707, 948.2864, 951.2624, 978.2960, 981.2753, 1042.0453, 1044.0452, 1062.0494, 1066.0582, 1097.9999, 1233.0858, 1284.0266, 1298.0360, 1300.0240, 1301.0287, 1336.5913, 1547.8593, 1547.8593, 1642.7354, 1722.8113, 1739.7985, 1740.8196, 1740.8196, 1789.7299, 1794.7837, 2256.2166, 2289.1689, 2365.1731, 2381.1460, 2403.0527, 2404.1614, 2404.1614, 2418.1707, 2422.1621, 2452.2048, 2454.1101, 2456.1501, 2461.1396, 2468.1174, 2470.1589, 2472.1538, 2473.1538, 2484.1687, 2486.1538, 2487.1653, 2501.1709, 2505.1096, 2878.3921, 2909.3730, 3153.4763, 3211.4216

16. gi|670985269 **Mass:** 51098 **Score:** 38 **Expect:** 5.4e+002 **Matches:** 7
PREDICTED: fibrinogen gamma chain [Ursus maritimus]

Observed	Mr(expt)	Mr(calc)	ppm	Start	End	Miss	Ions	Peptide
2256.2166	2255.2093	2255.2287	-8.59	412 -	432	1	---	K.IIPFNRLAIGEGQQHHLGGAK.Q
2365.1731	2364.1658	2364.1453	8.68	115 -	134	1	---	K.MMEEIIKYEALVGSHESNIR.F + Oxidation (M)
2381.1460	2380.1387	2380.1402	-0.63	115 -	134	1	---	K.MMEEIIKYEALVGSHESNIR.F + 2 Oxidation (M)
2456.1501	2455.1428	2455.1842	-16.86	202 -	222	0	---	K.QQFLVYCEIDGTGNGWTVLQK.R
2878.3921	2877.3848	2877.3181	23.2	383 -	406	1	---	K.TSTPNGYDNGIIWATWQSRWYSMK.K + Oxidation (M)
2892.3655	2891.3582	2891.2086	51.7	302 -	328	0	---	R.LTYAYFIGGDAGDAFDGYDFGDDPSDK.F
3211.4216	3210.4143	3210.3731	12.8	300 -	328	1	---	K.YRLTYAYFIGGDAGDAFDGYDFGDDPSDK.F

No match to: 854.0066, 855.0240, 856.0018, 858.0048, 859.2367, 859.9990, 860.2466, 871.0083, 873.0118, 886.9786, 888.2522, 892.2707, 902.2651, 918.2707, 948.2864, 951.2624, 978.2960, 981.2753, 1042.0453, 1044.0452, 1062.0494, 1066.0582, 1097.9999, 1233.0858, 1284.0266, 1298.0360, 1300.0240, 1301.0287, 1320.5753, 1336.5913, 1547.8593, 1547.8593, 1642.7354, 1722.8113, 1739.7985, 1740.8196, 1740.8196, 1789.7299, 1794.7837, 2289.1689, 2403.0527, 2404.1614, 2404.1614, 2408.1543, 2418.1707, 2422.1621, 2452.2048, 2454.1101, 2457.1443, 2461.1396, 2468.1174, 2470.1589, 2472.1538, 2473.1538, 2484.1687, 2486.1538, 2487.1653, 2501.1709, 2505.1096, 2909.3730, 3153.4763

17. gi|4838361 **Mass:** 13740 **Score:** 38 **Expect:** 5.8e+002 **Matches:** 6
immunoglobulin heavy chain variable region [Mus musculus]

Observed	Mr(expt)	Mr(calc)	ppm	Start	End	Miss	Ions	Peptide
1789.7299	1788.7226	1788.8042	-45.62	24 -	38	0	---	K.ASGYSFTGHYMQWVR.Q
2365.1731	2364.1658	2364.0944	30.2	102 -	123	1	---	K.SVVAKDYAMDYWGQGTTVTVSS.-
2381.1460	2380.1387	2380.0893	20.8	102 -	123	1	---	K.SVVAKDYAMDYWGQGTTVTVSS.- + Oxidation (M)
2404.1614	2403.1541	2403.0954	24.5	44 -	63	0	---	K.SLEWIGYINCYNGATSYHQK.F
2404.1614	2403.1541	2403.0954	24.5	44 -	63	0	---	K.SLEWIGYINCYNGATSYHQK.F
2505.1096	2504.1023	2504.2615	-63.55	1 -	23	1	---	-.QVQLQESGPELVKTGASVMISCK.A + Oxidation (M)

No match to: 854.0066, 855.0240, 856.0018, 858.0048, 859.2367, 859.9990, 860.2466, 871.0083, 873.0118, 886.9786, 888.2522,

892.2707, 902.2651, 918.2707, 948.2864, 951.2624, 978.2960, 981.2753, 1042.0453, 1044.0452, 1062.0494, 1066.0582, 1097.9999, 1233.0858, 1284.0266, 1298.0360, 1300.0240, 1301.0287, 1320.5753, 1336.5913, 1547.8593, 1547.8593, 1642.7354, 1722.8113, 1739.7985, 1740.8196, 1740.8196, 1794.7837, 2256.2166, 2289.1689, 2403.0527, 2408.1543, 2418.1707, 2422.1621, 2452.2048, 2454.1101, 2456.1501, 2457.1443, 2461.1396, 2468.1174, 2470.1589, 2472.1538, 2473.1538, 2484.1687, 2486.1538, 2487.1653, 2501.1709, 2878.3921, 2892.3655, 2909.3730, 3153.4763, 3211.4216

18. gi|821101831 **Mass:** 59974 **Score:** 38 **Expect:** 5.8e+002 **Matches:** 7

PREDICTED: zinc finger and BTB domain-containing protein 18 isoform X2 [Dasypus novemcinctus]

Observed	Mr(expt)	Mr(calc)	ppm	Start	End	Miss	Ions	Peptide
1789.7299	1788.7226	1788.6720	28.3	2 -	16	0	---	M.MGGYEDSMEFPDHSR.H + 2 Oxidation (M)
2289.1689	2288.1616	2288.0921	30.4	255 -	275	0	---	K.SSLSGVENLNSSYFSSQDVLR.S
2452.2048	2451.1975	2451.1603	15.2	48 -	67	1	---	R.AVLASCSMYFHLFYKDQLDK.R + Oxidation (M)
2456.1501	2455.1428	2455.2893	-59.66	497 -	518	1	---	K.FHCELVNSLSVKSEALSLPAVR.D
2487.1653	2486.1580	2486.1696	-4.66	27 -	47	1	---	R.HQGFLCDCTVLVGDAQFRAHR.A
2892.3655	2891.3582	2891.5143	-53.97	95 -	119	1	---	K.LQFKDLPIEDVLAAASYLHMYDIVK.V
3153.4763	3152.4690	3152.3572	35.5	1 -	26	1	---	-.MMGGYEDSMEFPDHSRHLLQCLSEQR.H

No match to: 854.0066, 855.0240, 856.0018, 858.0048, 859.2367, 859.9990, 860.2466, 871.0083, 873.0118, 886.9786, 888.2522, 892.2707, 902.2651, 918.2707, 948.2864, 951.2624, 978.2960, 981.2753, 1042.0453, 1044.0452, 1062.0494, 1066.0582, 1097.9999, 1233.0858, 1284.0266, 1298.0360, 1300.0240, 1301.0287, 1320.5753, 1336.5913, 1547.8593, 1547.8593, 1642.7354, 1722.8113, 1739.7985, 1740.8196, 1740.8196, 1794.7837, 2256.2166, 2365.1731, 2381.1460, 2403.0527, 2404.1614, 2404.1614, 2408.1543, 2418.1707, 2422.1621, 2454.1101, 2457.1443, 2461.1396, 2468.1174, 2470.1589, 2472.1538, 2473.1538, 2484.1687, 2486.1538, 2501.1709, 2505.1096, 2878.3921, 2909.3730, 3211.4216

19. gi|831217526 **Mass:** 13851 **Score:** 38 **Expect:** 6e+002 **Matches:** 5

PREDICTED: peptidyl-prolyl cis-trans isomerase-like 3 [Otolemur garnettii]

Observed	Mr(expt)	Mr(calc)	ppm	Start	End	Miss	Ions	Peptide
1794.7837	1793.7764	1793.8731	-53.87	46 -	62	1	---	R.NIKGFMVQTGDPTGTGR.G + Oxidation (M)
2403.0527	2402.0454	2402.1325	-36.25	86 -	108	0	---	R.GVVSMANNGPNTNGSQFFITYGK.Q
2404.1614	2403.1541	2403.1893	-14.64	2 -	21	1	---	M.WSVTLHTDVGDIKIEVFCER.T
2404.1614	2403.1541	2403.1893	-14.64	2 -	21	1	---	M.WSVTLHTDVGDIKIEVFCER.T
2909.3730	2908.3657	2908.4039	-13.12	82 -	108	1	---	K.HNVRGVVSMANNGPNTNGSQFFITYGK.Q

No match to: 854.0066, 855.0240, 856.0018, 858.0048, 859.2367, 859.9990, 860.2466, 871.0083, 873.0118, 886.9786, 888.2522, 892.2707, 902.2651, 918.2707, 948.2864, 951.2624, 978.2960, 981.2753, 1042.0453, 1044.0452, 1062.0494, 1066.0582, 1097.9999, 1233.0858, 1284.0266, 1298.0360, 1300.0240, 1301.0287, 1320.5753, 1336.5913, 1547.8593, 1547.8593, 1642.7354, 1722.8113, 1739.7985, 1740.8196, 1740.8196, 1789.7299, 2256.2166, 2289.1689, 2365.1731, 2381.1460, 2408.1543, 2418.1707, 2422.1621, 2452.2048, 2454.1101, 2456.1501, 2457.1443, 2461.1396, 2468.1174, 2470.1589, 2472.1538, 2473.1538, 2484.1687, 2486.1538, 2487.1653, 2501.1709, 2505.1096, 2878.3921, 2892.3655, 3153.4763, 3211.4216

20. gi|149052108 **Mass:** 12683 **Score:** 38 **Expect:** 6e+002 **Matches:** 5

similar to implantation serine proteinase 2 (predicted), partial [Rattus norvegicus]

Observed	Mr(expt)	Mr(calc)	ppm	Start	End	Miss	Ions	Peptide
1547.8593	1546.8520	1546.7425	70.8	1 -	13	0	---	-.MVHMYGSLPPPYR.L
1547.8593	1546.8520	1546.7425	70.8	1 -	13	0	---	-.MVHMYGSLPPPYR.L
2256.2166	2255.2093	2255.1885	9.24	2 -	20	1	---	M.VHMYGSLPPPYRLQQVQVK.I + Oxidation (M)
2403.0527	2402.0454	2402.2239	-74.29	1 -	20	1	---	-.MVHMYGSLPPPYRLQQVQVK.I + 2 Oxidation (M)
2422.1621	2421.1548	2421.3130	-65.34	92 -	112	1	---	R.GIPGVYTLVQSFLPWITKQMK.- + Oxidation (M)

No match to: 854.0066, 855.0240, 856.0018, 858.0048, 859.2367, 859.9990, 860.2466, 871.0083, 873.0118, 886.9786, 888.2522, 892.2707, 902.2651, 918.2707, 948.2864, 951.2624, 978.2960, 981.2753, 1042.0453, 1044.0452, 1062.0494, 1066.0582, 1097.9999, 1233.0858, 1284.0266, 1298.0360, 1300.0240, 1301.0287, 1320.5753, 1336.5913, 1642.7354, 1722.8113, 1739.7985, 1740.8196, 1740.8196, 1789.7299, 1794.7837, 2289.1689, 2365.1731, 2381.1460, 2404.1614, 2404.1614, 2408.1543, 2418.1707, 2452.2048, 2454.1101, 2456.1501, 2457.1443, 2461.1396, 2468.1174, 2470.1589, 2472.1538, 2473.1538, 2484.1687, 2486.1538, 2487.1653, 2501.1709, 2505.1096, 2878.3921, 2892.3655, 2909.3730, 3153.4763, 3211.4216

Search Parameters

Type of search : Sequence Query
Enzyme : Trypsin
Fixed modifications : Carbamidomethyl (C)
Variable modifications : Oxidation (M)
Mass values : Monoisotopic
Protein Mass : Unrestricted
Peptide Mass Tolerance : ± 80 ppm
Fragment Mass Tolerance: ± 0.3 Da
Max Missed Cleavages : 1
Instrument type : MALDI-TOF-TOF
Query1 (854.0066,1+) : <no title>
Query2 (855.0240,1+) : <no title>
Query3 (856.0018,1+) : <no title>
Query4 (858.0048,1+) : <no title>
Query5 (859.2367,1+) : <no title>
Query6 (859.9990,1+) : <no title>
Query7 (860.2466,1+) : <no title>
Query8 (871.0083,1+) : <no title>
Query9 (873.0118,1+) : <no title>
Query10 (886.9786,1+) : <no title>
Query11 (888.2522,1+) : <no title>
Query12 (892.2707,1+) : <no title>
Query13 (902.2651,1+) : <no title>
Query14 (918.2707,1+) : <no title>
Query15 (948.2864,1+) : <no title>
Query16 (951.2624,1+) : <no title>
Query17 (978.2960,1+) : <no title>
Query18 (981.2753,1+) : <no title>
Query19 (1042.0453,1+) : <no title>
Query20 (1044.0452,1+) : <no title>

```
Query21 (1062.0494,1+) : <no title>
Query22 (1066.0582,1+) : <no title>
Query23 (1097.9999,1+) : <no title>
Query24 (1233.0858,1+) : <no title>
Query25 (1284.0266,1+) : <no title>
Query26 (1298.0360,1+) : <no title>
Query27 (1300.0240,1+) : <no title>
Query28 (1301.0287,1+) : <no title>
Query29 (1320.5753,1+) : <no title>
Query30 (1336.5913,1+) : <no title>
Query31 (1547.8593,1+) : <no title>
Query32 (1547.8593,1+) : MaldiWellID: 55969, SpectrumID: 109895,
Query33 (1642.7354,1+) : <no title>
Query34 (1722.8113,1+) : <no title>
Query35 (1739.7985,1+) : <no title>
Query36 (1740.8196,1+) : <no title>
Query37 (1740.8196,1+) : MaldiWellID: 55969, SpectrumID: 109894,
Query38 (1789.7299,1+) : <no title>
Query39 (1794.7837,1+) : <no title>
Query40 (2256.2166,1+) : <no title>
Query41 (2289.1689,1+) : <no title>
Query42 (2365.1731,1+) : <no title>
Query43 (2381.1460,1+) : <no title>
Query44 (2403.0527,1+) : <no title>
Query45 (2404.1614,1+) : <no title>
Query46 (2404.1614,1+) : MaldiWellID: 55969, SpectrumID: 109896,
Query47 (2408.1543,1+) : <no title>
Query48 (2418.1707,1+) : <no title>
Query49 (2422.1621,1+) : <no title>
Query50 (2452.2048,1+) : <no title>
Query51 (2454.1101,1+) : <no title>
Query52 (2456.1501,1+) : <no title>
Query53 (2457.1443,1+) : <no title>
Query54 (2461.1396,1+) : <no title>
Query55 (2468.1174,1+) : <no title>
Query56 (2470.1589,1+) : <no title>
Query57 (2472.1538,1+) : <no title>
Query58 (2473.1538,1+) : <no title>
Query59 (2484.1687,1+) : <no title>
Query60 (2486.1538,1+) : <no title>
Query61 (2487.1653,1+) : <no title>
Query62 (2501.1709,1+) : <no title>
Query63 (2505.1096,1+) : <no title>
Query64 (2878.3921,1+) : <no title>
Query65 (2892.3655,1+) : <no title>
Query66 (2909.3730,1+) : <no title>
Query67 (3153.4763,1+) : <no title>
Query68 (3211.4216,1+) : <no title>
```
