# Supplementary material for: Vasectomy and Photoperiodic Regimen Modify the Protein Profile, Hormonal Content and Antioxidant Enzymes Activity of Ram Seminal Plasma
Source: Int J Mol Sci. 2020 Oct 29;21(21):8063. doi: 10.3390/ijms21218063 (PMC7663742; doi:10.3390/ijms21218063)
Supplement: Supplementary file 1 [file ijms-21-08063-s001.zip › Supplementary file 2_Mascot protein identification/Lactotransferrin identification.pdf]

Mascot Search Results

Protein View

Match to: **TRFL_CAPHI** Score: **258** Expect: **1.1e-021**
Lactotransferrin OS=Capra hircus GN=LTF PE=1 SV=1

Nominal mass (M_r): **79361**; Calculated pI value: **8.34**
NCBI BLAST search of TRFL_CAPHI against nr
Unformatted sequence string for pasting into other applications

Taxonomy: Capra hircus

Fixed modifications: Carbamidomethyl (C)
Variable modifications: Oxidation (M)
Cleavage by Trypsin: cuts C-term side of KR unless next residue is P
Number of mass values searched: **65**
Number of mass values matched: **28**
Sequence Coverage: **41%**

Matched peptides shown in **Bold Red**

1 MKLFVPALLS LGALGLCLAA PRKNVRWCAI SLPEWSK**CYQ** **WQRR**MRKLGA
51 PSITCIRRTS ALECIRAIAG KNADAVTLDS GMVFEAGLDP YK**LRPVAAEI**
101 **YGTEKSPQTH YYAVAVVKKG SNFQLDQLQG QK**SCHMGLGR SAGWNIPVGI
151 LRPFLSWTES AEPLQGAVAR FFSASCVPCV DGKAYPNLCQ LCKGVGENK**C**
201 **ACSSQEPYFG YSGAFK**CLQD GAGDVAFVKE TTVFENLPEK ADRDQYELLC
251 LNNTR**APVDA FKECHLAQVP SHAVVARSVD GKENLIWELL R**KAQEKFGKN
301 KSQSFQLFGS PEGRR**DLLFK DSALGFVRIP SKVDSALYLG SR**YLTALKNL
351 RETAEELKAR CTRVVWCAVG PEEQSKCQQW SEQSGQNVTC ATASTTDDCI
401 ALVLKGEADA LSLDGGYIYT AGK**CGLVPVM AENR**KSSKY**S SLDCVLRPTE**
451 **GYLAVAVVKK** ANEGLTWNSL KGKKSCHTAV DRTAGWNIPM GLIANQTGSC
501 AFDEFFSQSC APGADPKSSL CALCAGDDQG LDKCVPNSK**E KYYGYTGAFR**
551 CLAEDVGDVA FVKNDTVWEN TNGESSADWA K**NLNREDFRL LCLDGTTKPV**
601 **TEAQSCYLAV APNHAVVSRS DRAAHVEQVL LHQQALFGK**N GK**NCPDQFCL**
651 **FK**SETK**NLLF NDNTECLAKL GGRPTYEKYL GTEYVTAIAN LKKCSTSPLL**
701 **EACAFLTR**

Show predicted peptides also

Sort Peptides By ◉ Residue Number ○ Increasing Mass ○ Decreasing Mass

Start - End	Observed	Mr(expt)	Mr(calc)	ppm	Miss	Sequence
38 - 43	940.3949	939.3876	939.4021	-15	0	K.CYQWQR.R
38 - 44	1096.4980	1095.4907	1095.5032	-11	1	K.CYQWQRR.M
93 - 105	1446.8004	1445.7931	1445.7878	4	0	K.LRPVAAEIYGTEK.S
93 - 118	2890.5337	2889.5264	2889.5388	-4	1	K.LRPVAAEIYGTEKSPQTHYYAVAVVK.K
106 - 118	1462.7750	1461.7677	1461.7616	4	0	K.SPQTHYYAVAVVK.K
119 - 132	1590.8412	1589.8339	1589.8162	11	1	K.KGSNFQLDQLQGQK.S
200 - 216	1958.7950	1957.7877	1957.7975	-5	0	K.CACSSQEPYFGYSGAFK.C
256 - 277	2402.2341	2401.2268	2401.2325	-2	1	R.APVDAFKECHLAQVPSHAVVAR.S
278 - 291	1671.9061	1670.8988	1670.8991	-0	1	R.SVDGKENLIWELLR.K
283 - 291	1185.6573	1184.6500	1184.6553	-4	0	K.ENLIWELLR.K
316 - 328	1480.8190	1479.8117	1479.8086	2	1	R.DLLFKDSALGFVR.I
329 - 342	1505.8336	1504.8263	1504.8249	1	1	R.IPSKVDSALYLGSR.Y
333 - 342	1080.5172	1079.5099	1079.5611	-47	0	K.VDSALYLGSR.Y
424 - 434	1245.5985	1244.5912	1244.6006	-7	0	K.CGLVPVMAENR.K
439 - 459	2340.2273	2339.2200	2339.2195	0	0	K.YSSLDCVLRPTEGYLAVAVVK.K
439 - 460	2468.3242	2467.3169	2467.3145	1	1	K.YSSLDCVLRPTEGYLAVAVVKK.A
540 - 550	1354.6420	1353.6347	1353.6353	-0	1	K.EKYYGYTGAFR.C
542 - 550	1097.4967	1096.4894	1096.4978	-8	0	K.YYGYTGAFR.C
582 - 589	1063.5172	1062.5099	1062.5206	-10	1	K.NLNREDFR.L
590 - 619	3270.6653	3269.6580	3269.6537	1	0	R.LLCLDGTTKPVTEAQSCYLAVAPNHAVVSR.S
620 - 639	2247.1912	2246.1839	2246.1920	-4	1	R.SDRAAHVEQVLLHQQALFGK.N
623 - 639	1889.0319	1888.0246	1888.0319	-4	0	R.AAHVEQVLLHQQALFGK.N
643 - 652	1328.5688	1327.5615	1327.5689	-6	0	K.NCPDQFCLFK.S
657 - 669	1551.7411	1550.7338	1550.7399	-4	0	K.NLLFNDNTECLAK.L
670 - 678	1020.5010	1019.4937	1019.5400	-45	0	K.LGGRPTYEK.Y
679 - 692	1555.8271	1554.8198	1554.8293	-6	0	K.YLGTEYVTAIANLK.K
679 - 693	1683.9292	1682.9219	1682.9243	-1	1	K.YLGTEYVTAIANLKK.C
693 - 708	1853.9751	1852.9678	1852.9175	27	1	K.KCSTSPLLEACAFLTR.-

No match to: 850.2828, 854.0068, 855.0179, 855.9982, 857.0005, 859.9986, 861.2448, 923.3679, 954.4086, 1044.039

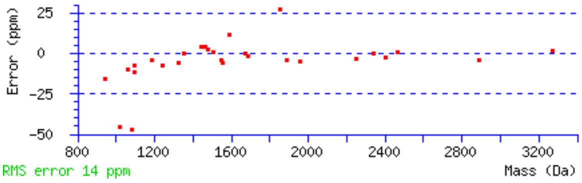

Mascot Search Results

```
User          :
Email         :
Search title  : SampleSetID: 610, AnalysisID: 4511, MaldiWellID: 55571, SpectrumID: 109834, Path=\160212\MS\16-13 Jose Alvaro
Database      : SwissProt sprot_160208 (550116 sequences; 196219159 residues)
Taxonomy      : Mammalia (mammals) (66429 sequences)
Timestamp     : 12 Feb 2016 at 10:22:56 GMT
Top Score     : 258 for TRFL_CAPHI, Lactotransferrin OS=Capra hircus GN=LTF PE=1 SV=1
```

Mascot Score Histogram

Protein score is -10*Log(P), where P is the probability that the observed match is a random event.
Protein scores greater than 61 are significant (p<0.05).

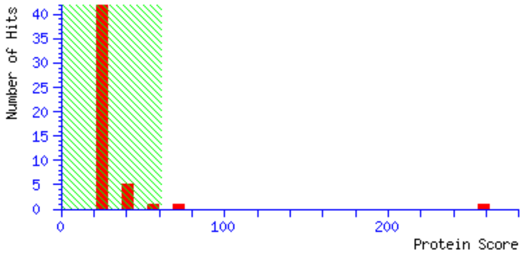

Protein Summary Report

Format As | Protein Summary | Help

Significance threshold p< 0.05 | Max. number of hits 20

Re-Search All | Search Unmatched

Index

	Accession	Mass	Score	Description
1.	TRFL_CAPHI	79361	258	Lactotransferrin OS=Capra hircus GN=LTF PE=1 SV=1
2.	TRFL_BOVIN	80002	67	Lactotransferrin OS=Bos taurus GN=LTF PE=1 SV=2
3.	TRFL_BUBBU	79733	58	Lactotransferrin OS=Bubalus bubalis GN=LTF PE=1 SV=1
4.	PR3C1_RAT	24901	38	Prolactin-3C1 OS=Rattus norvegicus GN=Prl3c1 PE=2 SV=1
5.	DMD_RAT	427559	37	Dystrophin OS=Rattus norvegicus GN=Dmd PE=1 SV=2
6.	CCD57_MOUSE	116693	37	Coiled-coil domain-containing protein 57 OS=Mus musculus GN=Ccdc57 PE=1 SV=1
7.	UCP1_OCHDA	33596	34	Mitochondrial brown fat uncoupling protein 1 OS=Ochotona dauurica GN=UCP1 PE=2 SV=1
8.	DPOLN_MOUSE	97640	33	DNA polymerase nu OS=Mus musculus GN=Poln PE=2 SV=2
9.	NDUS1_RAT	80331	32	NADH-ubiquinone oxidoreductase 75 kDa subunit, mitochondrial OS=Rattus norvegicus GN=Ndufs1 PE=1 SV=1
10.	GIT2_HUMAN	85117	31	ARF GTPase-activating protein GIT2 OS=Homo sapiens GN=GIT2 PE=1 SV=2
11.	RABP2_HUMAN	15854	31	Cellular retinoic acid-binding protein 2 OS=Homo sapiens GN=CRABP2 PE=1 SV=2
12.	IOD2_BOVIN	30628	29	Type II iodothyronine deiodinase OS=Bos taurus GN=DIO2 PE=2 SV=2
13.	NDUS1_MESAU	20780	29	NADH-ubiquinone oxidoreductase 75 kDa subunit, mitochondrial (Fragments) OS=Mesocricetus auratus GN=NDUFS1 PE=1 SV=1
14.	PTK6_HUMAN	52371	29	Protein-tyrosine kinase 6 OS=Homo sapiens GN=PTK6 PE=1 SV=1
15.	KAP2_BOVIN	45408	29	cAMP-dependent protein kinase type II-alpha regulatory subunit OS=Bos taurus GN=PRKAR2A PE=1 SV=2
16.	PCID2_BOVIN	47410	29	PCI domain-containing protein 2 OS=Bos taurus GN=PCID2 PE=2 SV=1
17.	HMCS1_RAT	58025	29	Hydroxymethylglutaryl-CoA synthase, cytoplasmic OS=Rattus norvegicus GN=Hmgcs1 PE=1 SV=1
18.	TM242_MOUSE	14964	29	Transmembrane protein 242 OS=Mus musculus GN=Tmem242 PE=2 SV=1
19.	ST2A2_MOUSE	33422	28	Bile salt sulfotransferase 2 OS=Mus musculus GN=Sult2a2 PE=2 SV=1
20.	SUH2_RAT	33432	28	Probable alcohol sulfotransferase OS=Rattus norvegicus PE=2 SV=2

Results List

1. TRFL_CAPHI **Mass:** 79361 **Score:** 258 **Expect:** 1.1e-021 **Matches:** 28
Lactotransferrin OS=Capra hircus GN=LTF PE=1 SV=1

Observed	Mr(expt)	Mr(calc)	ppm	Start		End	Miss	Peptide
940.3949	939.3876	939.4021	-15.43	38	-	43	0	K.CYQWQR.R
1020.5010	1019.4937	1019.5400	-45.37	670	-	678	0	K.LGGRPTYEK.Y
1063.5172	1062.5099	1062.5206	-10.08	582	-	589	1	K.NLNREDFR.L
1080.5172	1079.5099	1079.5611	-47.41	333	-	342	0	K.VDSALYLGSR.Y
1096.4980	1095.4907	1095.5032	-11.41	38	-	44	1	K.CYQWQRR.M
1097.4967	1096.4894	1096.4978	-7.63	542	-	550	0	K.YYGYTGAFR.C
1185.6573	1184.6500	1184.6553	-4.47	283	-	291	0	K.ENLIWELLR.K
1245.5985	1244.5912	1244.6006	-7.50	424	-	434	0	K.CGLVPVMAENR.K
1328.5688	1327.5615	1327.5689	-5.59	643	-	652	0	K.NCPDQFCLFK.S
1354.6420	1353.6347	1353.6353	-0.45	540	-	550	1	K.EKYYGYTGAFR.C
1446.8004	1445.7931	1445.7878	3.69	93	-	105	0	K.LRPVAAEIYGTEK.S
1462.7750	1461.7677	1461.7616	4.18	106	-	118	0	K.SPQTHYYAVAVVK.K
1480.8190	1479.8117	1479.8086	2.14	316	-	328	1	R.DLLFKDSALGFVR.I
1505.8336	1504.8263	1504.8249	0.93	329	-	342	1	R.IPSKVDSALYLGSR.Y
1551.7411	1550.7338	1550.7399	-3.90	657	-	669	0	K.NLLFNDNTECLAK.L
1555.8271	1554.8198	1554.8293	-6.11	679	-	692	0	K.YLGTEYVTAIANLK.K
1590.8412	1589.8339	1589.8162	11.2	119	-	132	1	K.KGSNFQLDQLQGQK.S
1671.9061	1670.8988	1670.8991	-0.19	278	-	291	1	R.SVDGKENLIWELLR.K
1683.9292	1682.9219	1682.9243	-1.40	679	-	693	1	K.YLGTEYVTAIANLKK.C
1853.9751	1852.9678	1852.9175	27.1	693	-	708	1	K.KCSTSPLLEACAFLTR.-
1889.0319	1888.0246	1888.0319	-3.86	623	-	639	0	R.AAHVEQVLLHQQALFGK.N
1958.7950	1957.7877	1957.7975	-4.99	200	-	216	0	K.CACSSQEPYFGYSGAFK.C
2247.1912	2246.1839	2246.1920	-3.59	620	-	639	1	R.SDRAAHVEQVLLHQQALFGK.N
2340.2273	2339.2200	2339.2195	0.22	439	-	459	0	K.YSSLDCVLRPTEGYLAVAVVK.K

2402.2341	2401.2268	2401.2325	-2.36	256	277	1	R.APVDAFKECHLAQVPSHAVVAR.S
2468.3242	2467.3169	2467.3145	1.00	439	460	1	K.YSSLDCVLRPTEGYLAVAVVKK.A
2890.5337	2889.5264	2889.5388	-4.30	93	118	1	K.LRPVAAEIYGTEKSPQTHYYAVAVVK.K
3270.6653	3269.6580	3269.6537	1.33	590	619	0	R.LLCLDGTTKPVTEAQSCYLAVAPNHAVVSR.S

No match to: 850.2828, 854.0068, 855.0179, 855.9982, 857.0005, 859.9986, 861.2448, 923.3679, 954.4086, 1044.0396, 1045.0438, 1167.6447, 1236.6335, 1300.0328, 1497.7250, 1511.7294, 1608.8254, 1655.8672, 1685.9320, 1738.8187, 1755.8503, 1769.8682, 1800.0060, 1883.9473, 1972.8079, 2189.1829, 2227.1101, 2351.2632, 2354.2493, 2416.2493, 2452.1997, 2482.3257, 2771.4365, 2899.5171, 3211.4919, 3254.7114, 3284.6660

2. TRFL_BOVIN **Mass:** 80002 **Score:** 67 **Expect:** 0.015 **Matches:** 12

Lactotransferrin OS=Bos taurus GN=LTF PE=1 SV=2

Observed	Mr(expt)	Mr(calc)	ppm	Start	End	Miss	Peptide
1063.5172	1062.5099	1062.5206	-10.08	582	589	1	K.NLNREDFR.L
1080.5172	1079.5099	1079.5611	-47.41	333	342	0	K.VDSALYLGSR.Y
1097.4967	1096.4894	1096.4978	-7.63	542	550	0	K.YYGYTGAFR.C
1328.5688	1327.5615	1327.6053	-32.99	643	652	1	K.NCPDKFCLFK.S
1354.6420	1353.6347	1353.6353	-0.45	540	550	1	K.EKYYGYTGAFR.C
1505.8336	1504.8263	1504.8249	0.93	329	342	1	R.IPSKVDSALYLGSR.Y
1551.7411	1550.7338	1550.7399	-3.90	657	669	0	K.NLLFNDNTECLAK.L
1608.8254	1607.8181	1607.7614	35.3	73	88	0	K.ADAVTLDGGMVFEAGR.D
1800.0060	1798.9987	1798.9829	8.81	333	348	1	K.VDSALYLGSRYLTTLK.N
1853.9751	1852.9678	1852.9175	27.1	693	708	1	K.KCSTSPLLEACAFLTR.-
2402.2341	2401.2268	2401.2325	-2.36	256	277	1	R.APVDAFKECHLAQVPSHAVVAR.S
2890.5337	2889.5264	2889.5388	-4.30	93	118	1	K.LRPVAAEIYGTKESPQTHYYAVAVVK.K

No match to: 850.2828, 854.0068, 855.0179, 855.9982, 857.0005, 859.9986, 861.2448, 923.3679, 940.3949, 954.4086, 1020.5010, 1044.0396, 1045.0438, 1096.4980, 1167.6447, 1185.6573, 1236.6335, 1245.5985, 1300.0328, 1446.8004, 1462.7750, 1480.8190, 1497.7250, 1511.7294, 1555.8271, 1590.8412, 1655.8672, 1671.9061, 1683.9292, 1685.9320, 1738.8187, 1755.8503, 1769.8682, 1883.9473, 1889.0319, 1958.7950, 1972.8079, 2189.1829, 2227.1101, 2247.1912, 2340.2273, 2351.2632, 2354.2493, 2416.2493, 2452.1997, 2468.3242, 2482.3257, 2771.4365, 2899.5171, 3211.4919, 3254.7114, 3270.6653, 3284.6660

3. TRFL_BUBBU **Mass:** 79733 **Score:** 58 **Expect:** 0.098 **Matches:** 11

Lactotransferrin OS=Bubalus bubalis GN=LTF PE=1 SV=1

Observed	Mr(expt)	Mr(calc)	ppm	Start	End	Miss	Peptide
1063.5172	1062.5099	1062.5206	-10.08	582	589	1	K.NLNREDFR.L
1080.5172	1079.5099	1079.5611	-47.41	333	342	0	K.VDSALYLGSR.Y
1097.4967	1096.4894	1096.4978	-7.63	542	550	0	K.YYGYTGAFR.C
1328.5688	1327.5615	1327.6053	-32.99	643	652	1	K.NCPDKFCLFK.S
1354.6420	1353.6347	1353.6353	-0.45	540	550	1	K.EKYYGYTGAFR.C
1505.8336	1504.8263	1504.8249	0.93	329	342	1	R.IPSKVDSALYLGSR.Y
1551.7411	1550.7338	1550.7399	-3.90	657	669	0	K.NLLFNDNTECLAK.L
1853.9751	1852.9678	1852.9175	27.1	693	708	1	K.KCSTSPLLEACAFLTR.-
2189.1829	2188.1756	2188.1389	16.8	623	642	0	R.AAHVEQVLLHQQALFGENGK.N
2402.2341	2401.2268	2401.2325	-2.36	256	277	1	R.APVDAFKECHLAQVPSHAVVAR.S
2890.5337	2889.5264	2889.5388	-4.30	93	118	1	K.LRPVAAEIYGTKESPQTHYYAVAVVK.K

No match to: 850.2828, 854.0068, 855.0179, 855.9982, 857.0005, 859.9986, 861.2448, 923.3679, 940.3949, 954.4086, 1020.5010, 1044.0396, 1045.0438, 1096.4980, 1167.6447, 1185.6573, 1236.6335, 1245.5985, 1300.0328, 1446.8004, 1462.7750, 1480.8190, 1497.7250, 1511.7294, 1555.8271, 1590.8412, 1608.8254, 1655.8672, 1671.9061, 1683.9292, 1685.9320, 1738.8187, 1755.8503, 1769.8682, 1800.0060, 1883.9473, 1889.0319, 1958.7950, 1972.8079, 2227.1101, 2247.1912, 2340.2273, 2351.2632, 2354.2493, 2416.2493, 2452.1997, 2468.3242, 2482.3257, 2771.4365, 2899.5171, 3211.4919, 3254.7114, 3270.6653, 3284.6660

4. PR3C1_RAT **Mass:** 24901 **Score:** 38 **Expect:** 10 **Matches:** 5

Prolactin-3C1 OS=Rattus norvegicus GN=Prl3c1 PE=2 SV=1

Observed	Mr(expt)	Mr(calc)	ppm	Start	End	Miss	Peptide
1063.5172	1062.5099	1062.5492	-36.93	1	9	0	-.MQLSLTQAR.T + Oxidation (M)
1462.7750	1461.7677	1461.7762	-5.80	1	12	1	-.MQLSLTQARTWK.G
1480.8190	1479.8117	1479.7755	24.5	58	69	1	K.MYKILDLNVAER.R + Oxidation (M)
2416.2493	2415.2420	2415.3413	-41.11	90	110	1	K.TNEDLLKVIISVSNAWIYPLK.M
3254.7114	3253.7041	3253.5933	34.1	111	139	1	K.MLIPAVLTHLGSYDGMMARAIELNYGNQK.I + 3 Oxidation (M)

No match to: 850.2828, 854.0068, 855.0179, 855.9982, 857.0005, 859.9986, 861.2448, 923.3679, 940.3949, 954.4086, 1020.5010, 1044.0396, 1045.0438, 1080.5172, 1096.4980, 1097.4967, 1167.6447, 1185.6573, 1236.6335, 1245.5985, 1300.0328, 1328.5688, 1354.6420, 1446.8004, 1497.7250, 1505.8336, 1511.7294, 1551.7411, 1555.8271, 1590.8412, 1608.8254, 1655.8672, 1671.9061, 1683.9292, 1685.9320, 1738.8187, 1755.8503, 1769.8682, 1800.0060, 1853.9751, 1883.9473, 1889.0319, 1958.7950, 1972.8079, 2189.1829, 2227.1101, 2247.1912, 2340.2273, 2351.2632, 2354.2493, 2402.2341, 2452.1997, 2468.3242, 2482.3257, 2771.4365, 2890.5337, 2899.5171, 3211.4919, 3270.6653, 3284.6660

5. DMD_RAT **Mass:** 427559 **Score:** 37 **Expect:** 14 **Matches:** 17

Dystrophin OS=Rattus norvegicus GN=Dmd PE=1 SV=2

Observed	Mr(expt)	Mr(calc)	ppm	Start	End	Miss	Peptide
1185.6573	1184.6500	1184.6223	23.4	250	259	0	R.EVEMLPRPSK.V
1236.6335	1235.6262	1235.5856	32.9	575	584	1	K.WLSEKEDAMK.N
1354.6420	1353.6347	1353.6711	-26.85	2600	2611	1	K.EGPHTMDAIQKK.I
1511.7294	1510.7221	1510.6947	18.2	3213	3225	1	K.QVASSTGFCDQRR.L
1590.8412	1589.8339	1589.8777	-27.52	2307	2320	1	R.ALPEKQGELEVHIK.D
1655.8672	1654.8599	1654.8314	17.2	2876	2888	1	K.LYQEPRELPPEER.A
1671.9061	1670.8988	1670.8879	6.53	1379	1392	0	K.SLHLIQESLEFIDK.Q
1853.9751	1852.9678	1852.9795	-6.32	724	738	1	K.RLDVDITELHSWITR.S
1883.9473	1882.9400	1882.9425	-1.29	1695	1709	1	K.WIIHTDELLDESEKR.K
2247.1912	2246.1839	2246.1001	37.3	1493	1511	0	K.SVEQEVVQSQLSHCVNLYK.S
2340.2273	2339.2200	2339.1580	26.5	1240	1258	1	K.ELETLTTNYQWLCTRLNGK.C
2351.2632	2350.2559	2350.2128	18.3	2268	2289	0	K.QLNETGGAVLVSAPIRPEEQDK.L
2402.2341	2401.2268	2401.1795	19.7	1933	1955	0	R.QAESLSENGAAMAVEPTQIQLSK.R
2416.2493	2415.2420	2415.2493	-3.03	2420	2444	0	R.APGLSTTGASASQTVTVVTQPVDTK.E
2890.5337	2889.5264	2889.5560	-10.22	2263	2289	1	R.QGILKQLNETGGAVLVSAPIRPEEQDK.L
2899.5171	2898.5098	2898.3759	46.2	3056	3079	1	R.AISPNKVPYYINHETQTTCWDHPK.M
3270.6653	3269.6580	3269.5346	37.7	378	406	0	K.EQFHAHEGFMMDLTSHQGLVGNVLQLGSR.L + 2 Oxidation (M)

No match to: 850.2828, 854.0068, 855.0179, 855.9982, 857.0005, 859.9986, 861.2448, 923.3679, 940.3949, 954.4086, 1020.5010, 1044.0396, 1045.0438, 1063.5172, 1080.5172, 1096.4980, 1097.4967, 1167.6447, 1245.5985, 1300.0328, 1328.5688, 1446.8004, 1462.7750, 1480.8190, 1497.7250, 1505.8336, 1551.7411, 1555.8271, 1608.8254, 1683.9292, 1685.9320, 1738.8187, 1755.8503, 1769.8682, 1800.0060, 1889.0319, 1958.7950, 1972.8079, 2189.1829, 2227.1101, 2354.2493, 2452.1997, 2468.3242, 2482.3257, 2771.4365, 3211.4919, 3254.7114, 3284.6660

6. CCD57_MOUSE **Mass:** 116693 **Score:** 37 **Expect:** 14 **Matches:** 9

Coiled-coil domain-containing protein 57 OS=Mus musculus GN=Ccdc57 PE=1 SV=1

Observed	Mr(expt)	Mr(calc)	ppm	Start	End	Miss	Peptide
1020.5010	1019.4937	1019.5400	-45.35	352 -	360	1	K.FREDAAALK.A
1446.8004	1445.7931	1445.7375	38.5	393 -	404	1	K.AQVARFQQDIDR.Y
1511.7294	1510.7221	1510.7852	-41.72	29 -	42	0	R.AQLQEAALQAAQNR.L
1655.8672	1654.8599	1654.7951	39.2	70 -	82	1	R.ELERYDVEFTQAR.Q
1738.8187	1737.8114	1737.8944	-47.74	861 -	876	1	R.GHKAEMASRPAQLSQK.Q
1853.9751	1852.9678	1852.9326	19.0	864 -	879	1	K.AEMASRPAQLSQKQHR.I + Oxidation (M)
1889.0319	1888.0246	1887.9843	21.4	880 -	894	1	R.IPTETWKPVYQKENR.T
3254.7114	3253.7041	3253.6652	12.0	305 -	332	1	R.AHAEELQTLDAKVLELQFLCETLEGQLR.R
3284.6660	3283.6587	3283.6181	12.4	607 -	640	0	K.TSVATADPHHGVHSSAAAADAALADQTSTALALR.K

No match to: 850.2828, 854.0068, 855.0179, 855.9982, 857.0005, 859.9986, 861.2448, 923.3679, 940.3949, 954.4086, 1044.0396, 1045.0438, 1063.5172, 1080.5172, 1096.4980, 1097.4967, 1167.6447, 1185.6573, 1236.6335, 1245.5985, 1300.0328, 1328.5688, 1354.6420, 1462.7750, 1480.8190, 1497.7250, 1505.8336, 1551.7411, 1555.8271, 1590.8412, 1608.8254, 1671.9061, 1683.9292, 1685.9320, 1755.8503, 1769.8682, 1800.0060, 1883.9473, 1958.7950, 1972.8079, 2189.1829, 2227.1101, 2247.1912, 2340.2273, 2351.2632, 2354.2493, 2402.2341, 2416.2493, 2452.1997, 2468.3242, 2482.3257, 2771.4365, 2890.5337, 2899.5171, 3211.4919, 3270.6653

7. UCP1_OCHDA **Mass:** 33596 **Score:** 34 **Expect:** 26 **Matches:** 5

Mitochondrial brown fat uncoupling protein 1 OS=Ochotona dauurica GN=UCP1 PE=2 SV=1

Observed	Mr(expt)	Mr(calc)	ppm	Start	End	Miss	Peptide
1462.7750	1461.7677	1461.8079	-27.47	162 -	174	0	R.IIATTESLSTLWK.G
1590.8412	1589.8339	1589.7832	31.9	41 -	54	0	R.LQIQGECQTTSGIR.Y
1800.0060	1798.9987	1798.9941	2.56	68 -	84	1	K.TEGPLKLYSGLPAGLQR.Q
2452.1997	2451.1924	2451.2865	-38.39	183 -	203	1	R.NIIINCTELVTYDLMKGALVR.N + Oxidation (M)
3270.6653	3269.6580	3269.6357	6.83	85 -	113	1	R.QISFASLRIGLYDTVQEFWGGEEATPSLR.S

No match to: 850.2828, 854.0068, 855.0179, 855.9982, 857.0005, 859.9986, 861.2448, 923.3679, 940.3949, 954.4086, 1020.5010, 1044.0396, 1045.0438, 1063.5172, 1080.5172, 1096.4980, 1097.4967, 1167.6447, 1185.6573, 1236.6335, 1245.5985, 1300.0328, 1328.5688, 1354.6420, 1446.8004, 1480.8190, 1497.7250, 1505.8336, 1511.7294, 1551.7411, 1555.8271, 1608.8254, 1655.8672, 1671.9061, 1683.9292, 1685.9320, 1738.8187, 1755.8503, 1769.8682, 1853.9751, 1883.9473, 1889.0319, 1958.7950, 1972.8079, 2189.1829, 2227.1101, 2247.1912, 2340.2273, 2351.2632, 2354.2493, 2402.2341, 2416.2493, 2468.3242, 2482.3257, 2771.4365, 2890.5337, 2899.5171, 3211.4919, 3254.7114, 3284.6660

8. DPOLN_MOUSE **Mass:** 97640 **Score:** 33 **Expect:** 33 **Matches:** 8

DNA polymerase nu OS=Mus musculus GN=Poln PE=2 SV=2

Observed	Mr(expt)	Mr(calc)	ppm	Start	End	Miss	Peptide
1245.5985	1244.5912	1244.6071	-12.72	442 -	451	1	K.IPVDKEEMER.T
1462.7750	1461.7677	1461.7245	29.5	447 -	459	1	K.EEMERTSALLGAR.L
1497.7250	1496.7177	1496.7406	-15.25	662 -	673	1	K.DIPIERVTHMDR.E + Oxidation (M)
1608.8254	1607.8181	1607.8308	-7.85	850 -	864	0	R.SWGHLTPLQEILGSA.-
1889.0319	1888.0246	1887.9803	23.5	253 -	271	0	K.SHVGNSTLTLPAHGGALEK.D
2354.2493	2353.2420	2353.2927	-21.52	823 -	842	0	R.IMESLQQVQTLELQLQVPLK.V + Oxidation (M)
2468.3242	2467.3169	2467.2383	31.9	608 -	629	0	R.TLFVSSEGHTFLAADFSQIELR.I
3254.7114	3253.7041	3253.6877	5.04	501 -	529	1	K.HLPRTGLQNQLSTSEAMLNSLQDLHPLPK.L + Oxidation (M)

No match to: 850.2828, 854.0068, 855.0179, 855.9982, 857.0005, 859.9986, 861.2448, 923.3679, 940.3949, 954.4086, 1020.5010, 1044.0396, 1045.0438, 1063.5172, 1080.5172, 1096.4980, 1097.4967, 1167.6447, 1185.6573, 1236.6335, 1300.0328, 1328.5688, 1354.6420, 1446.8004, 1480.8190, 1505.8336, 1511.7294, 1551.7411, 1555.8271, 1590.8412, 1655.8672, 1671.9061, 1683.9292, 1685.9320, 1738.8187, 1755.8503, 1769.8682, 1800.0060, 1853.9751, 1883.9473, 1958.7950, 1972.8079, 2189.1829, 2227.1101, 2247.1912, 2340.2273, 2351.2632, 2402.2341, 2416.2493, 2452.1997, 2482.3257, 2771.4365, 2890.5337, 2899.5171, 3211.4919, 3270.6653, 3284.6660

9. NDUS1_RAT **Mass:** 80331 **Score:** 32 **Expect:** 44 **Matches:** 7

NADH-ubiquinone oxidoreductase 75 kDa subunit, mitochondrial OS=Rattus norvegicus GN=Ndufs1 PE=1 SV=1

Observed	Mr(expt)	Mr(calc)	ppm	Start	End	Miss	Peptide
1097.4967	1096.4894	1096.5149	-23.23	593 -	602	0	K.SATYVNTEGR.A
1354.6420	1353.6347	1353.6235	8.32	201 -	212	0	R.GNDMQVGTYIEK.M
1505.8336	1504.8263	1504.8072	12.7	544 -	557	0	K.LLFLLGADGGCITR.Q
1608.8254	1607.8181	1607.7791	24.3	185 -	200	0	R.FASEIAGVDDLGTTGR.G
2351.2632	2350.2559	2350.1483	45.8	88 -	108	1	K.VVAACAMPVMKGWNILTNSEK.S + 2 Oxidation (M)
2416.2493	2415.2420	2415.2434	-0.58	429 -	450	1	K.VALIGSPVDLTYRYDHLGDSPK.I
2899.5171	2898.5098	2898.5888	-27.26	326 -	355	1	R.VAGMLQSFEGKAVAAIAGGLVDAEALVALK.D

No match to: 850.2828, 854.0068, 855.0179, 855.9982, 857.0005, 859.9986, 861.2448, 923.3679, 940.3949, 954.4086, 1020.5010, 1044.0396, 1045.0438, 1063.5172, 1080.5172, 1096.4980, 1167.6447, 1185.6573, 1236.6335, 1245.5985, 1300.0328, 1328.5688, 1446.8004, 1462.7750, 1480.8190, 1497.7250, 1511.7294, 1551.7411, 1555.8271, 1590.8412, 1655.8672, 1671.9061, 1683.9292, 1685.9320, 1738.8187, 1755.8503, 1769.8682, 1800.0060, 1853.9751, 1883.9473, 1889.0319, 1958.7950, 1972.8079, 2189.1829, 2227.1101, 2247.1912, 2340.2273, 2354.2493, 2402.2341, 2452.1997, 2468.3242, 2482.3257, 2771.4365, 2890.5337, 3211.4919, 3254.7114, 3270.6653, 3284.6660

10. GIT2_HUMAN **Mass:** 85117 **Score:** 31 **Expect:** 52 **Matches:** 7

ARF GTPase-activating protein GIT2 OS=Homo sapiens GN=GIT2 PE=1 SV=2

Observed	Mr(expt)	Mr(calc)	ppm	Start	End	Miss	Peptide
1063.5172	1062.5099	1062.5128	-2.69	143 -	151	0	R.TGNLETCLR.L
1080.5172	1079.5099	1079.5393	-27.25	693 -	701	1	K.SDMVRTSLR.L + Oxidation (M)
1655.8672	1654.8599	1654.8348	15.2	702 -	715	1	R.LLTSSAYRLQSECK.K
1671.9061	1670.8988	1670.8780	12.5	152 -	166	0	R.LLSLGAQANFFHPEK.G
2452.1997	2451.1924	2451.1839	3.47	413 -	434	1	R.QKSLDSDLSDGPVTVQEFMEVK.N
2771.4365	2770.4292	2770.3571	26.0	666 -	689	1	K.HDSYIPCSERIHVAVTEMAALFPK.K
3284.6660	3283.6587	3283.6759	-5.22	717 -	747	1	K.TLPGDPGSPTDVQLVTQQVIQCAYDIAKAAK.Q

No match to: 850.2828, 854.0068, 855.0179, 855.9982, 857.0005, 859.9986, 861.2448, 923.3679, 940.3949, 954.4086, 1020.5010, 1044.0396, 1045.0438, 1096.4980, 1097.4967, 1167.6447, 1185.6573, 1236.6335, 1245.5985, 1300.0328, 1328.5688, 1354.6420, 1446.8004, 1462.7750, 1480.8190, 1497.7250, 1505.8336, 1511.7294, 1551.7411, 1555.8271, 1590.8412, 1608.8254, 1683.9292, 1685.9320, 1738.8187, 1755.8503, 1769.8682, 1800.0060, 1853.9751, 1883.9473, 1889.0319, 1958.7950, 1972.8079, 2189.1829, 2227.1101, 2247.1912, 2340.2273, 2351.2632, 2354.2493, 2402.2341, 2416.2493, 2468.3242, 2482.3257, 2890.5337, 2899.5171, 3211.4919, 3254.7114, 3270.6653

11. RABP2_HUMAN **Mass:** 15854 **Score:** 31 **Expect:** 53 **Matches:** 5

Cellular retinoic acid-binding protein 2 OS=Homo sapiens GN=CRABP2 PE=1 SV=2

Observed	Mr(expt)	Mr(calc)	ppm	Start	End	Miss	Peptide
1080.5172	1079.5099	1079.4858	22.3	1 -	9	0	-.MPNFSGNWK.I
1096.4980	1095.4907	1095.4808	9.10	1 -	9	0	-.MPNFSGNWK.I + Oxidation (M)
1462.7750	1461.7677	1461.7551	8.66	1 -	12	1	-.MPNFSGNWKIIR.S
1497.7250	1496.7177	1496.7835	-43.93	55 -	67	1	K.TSTTVRTTEINFK.V
2899.5171	2898.5098	2898.4103	34.3	113 -	137	1	R.ELTNDGELILTMTADDVVCTRVYVR.E + Oxidation (M)

No match to: 850.2828, 854.0068, 855.0179, 855.9982, 857.0005, 859.9986, 861.2448, 923.3679, 940.3949, 954.4086, 1020.5010, 1044.0396, 1045.0438, 1063.5172, 1097.4967, 1167.6447, 1185.6573, 1236.6335, 1245.5985, 1300.0328, 1328.5688, 1354.6420, 1446.8004, 1480.8190, 1505.8336, 1511.7294, 1551.7411, 1555.8271, 1590.8412, 1608.8254, 1655.8672, 1671.9061, 1683.9292, 1685.9320, 1738.8187, 1755.8503,

1769.8682, 1800.0060, 1853.9751, 1883.9473, 1889.0319, 1958.7950, 1972.8079, 2189.1829, 2227.1101, 2247.1912, 2340.2273, 2351.2632, 2354.2493, 2402.2341, 2416.2493, 2452.1997, 2468.3242, 2482.3257, 2771.4365, 2890.5337, 3211.4919, 3254.7114, 3270.6653, 3284.6660

12. IOD2_BOVIN **Mass:** 30628 **Score:** 29 **Expect:** 78 **Matches:** 6

Type II iodothyronine deiodinase OS=Bos taurus GN=DIO2 PE=2 SV=2

Observed	Mr(expt)	Mr(calc)	ppm	Start	End	Miss	Peptide
940.3949	939.3876	939.4154	-29.54	54 -	61	0	R.MLTSEGMR.C + Oxidation (M)
954.4086	953.4013	953.4427	-43.42	185 -	191	1	K.HRNQEDR.C
1080.5172	1079.5099	1079.5216	-10.78	53 -	61	1	R.RMLTSEGMR.C
1096.4980	1095.4907	1095.5165	-23.51	53 -	61	1	R.RMLTSEGMR.C + Oxidation (M)
1511.7294	1510.7221	1510.7094	8.39	54 -	65	1	R.MLTSEGMRCIWK.S
2227.1101	2226.1028	2226.0488	24.3	210 -	229	1	R.VVADRMDNNANVAYGVAFER.V + Oxidation (M)

No match to: 850.2828, 854.0068, 855.0179, 855.9982, 857.0005, 859.9986, 861.2448, 923.3679, 1020.5010, 1044.0396, 1045.0438, 1063.5172, 1097.4967, 1167.6447, 1185.6573, 1236.6335, 1245.5985, 1300.0328, 1328.5688, 1354.6420, 1446.8004, 1462.7750, 1480.8190, 1497.7250, 1505.8336, 1551.7411, 1555.8271, 1590.8412, 1608.8254, 1655.8672, 1671.9061, 1683.9292, 1685.9320, 1738.8187, 1755.8503, 1769.8682, 1800.0060, 1853.9751, 1883.9473, 1889.0319, 1958.7950, 1972.8079, 2189.1829, 2247.1912, 2340.2273, 2351.2632, 2354.2493, 2402.2341, 2416.2493, 2452.1997, 2468.3242, 2482.3257, 2771.4365, 2890.5337, 2899.5171, 3211.4919, 3254.7114, 3270.6653, 3284.6660

13. NDUS1_MESAU **Mass:** 20780 **Score:** 29 **Expect:** 82 **Matches:** 4

NADH-ubiquinone oxidoreductase 75 kDa subunit, mitochondrial (Fragments) OS=Mesocricetus auratus GN=NDUFS1 PE=1 SV=1

Observed	Mr(expt)	Mr(calc)	ppm	Start	End	Miss	Peptide
1097.4967	1096.4894	1096.5149	-23.23	138 -	147	0	R.SATYVNTEGR.V
1505.8336	1504.8263	1504.8072	12.7	124 -	137	0	K.LLFLLGADGGCITR.S
1608.8254	1607.8181	1607.7791	24.3	1 -	16	0	-.FASEIAGVDDLGTTGR.K
2416.2493	2415.2420	2415.2434	-0.58	77 -	98	1	R.VALIGSPVDLTYRYDHLGDSPK.I

No match to: 850.2828, 854.0068, 855.0179, 855.9982, 857.0005, 859.9986, 861.2448, 923.3679, 940.3949, 954.4086, 1020.5010, 1044.0396, 1045.0438, 1063.5172, 1080.5172, 1096.4980, 1167.6447, 1185.6573, 1236.6335, 1245.5985, 1300.0328, 1328.5688, 1354.6420, 1446.8004, 1462.7750, 1480.8190, 1497.7250, 1511.7294, 1551.7411, 1555.8271, 1590.8412, 1655.8672, 1671.9061, 1683.9292, 1685.9320, 1738.8187, 1755.8503, 1769.8682, 1800.0060, 1853.9751, 1883.9473, 1889.0319, 1958.7950, 1972.8079, 2189.1829, 2227.1101, 2247.1912, 2340.2273, 2351.2632, 2354.2493, 2402.2341, 2452.1997, 2468.3242, 2482.3257, 2771.4365, 2890.5337, 2899.5171, 3211.4919, 3254.7114, 3270.6653, 3284.6660

14. PTK6_HUMAN **Mass:** 52371 **Score:** 29 **Expect:** 88 **Matches:** 6

Protein-tyrosine kinase 6 OS=Homo sapiens GN=PTK6 PE=1 SV=1

Observed	Mr(expt)	Mr(calc)	ppm	Start	End	Miss	Peptide
954.4086	953.4013	953.4277	-27.62	189 -	195	0	R.EEFTLCR.K
1245.5985	1244.5912	1244.5561	28.2	441 -	451	0	R.LSSFTSYENPT.-
1354.6420	1353.6347	1353.6823	-35.15	1 -	12	1	-.MVSRDQAHLGPK.Y + Oxidation (M)
1551.7411	1550.7338	1550.7044	19.0	406 -	418	0	R.MPCPLECPPSVHK.L
2351.2632	2350.2559	2350.2433	5.36	137 -	156	0	R.LHLNEAVSFLSLPELVNYHR.A
2482.3257	2481.3184	2481.2832	14.2	220 -	240	1	K.VISRDNLLHQQMLQSEIQAMK.K

No match to: 850.2828, 854.0068, 855.0179, 855.9982, 857.0005, 859.9986, 861.2448, 923.3679, 940.3949, 1020.5010, 1044.0396, 1045.0438, 1063.5172, 1080.5172, 1096.4980, 1097.4967, 1167.6447, 1185.6573, 1236.6335, 1300.0328, 1328.5688, 1446.8004, 1462.7750, 1480.8190, 1497.7250, 1505.8336, 1511.7294, 1555.8271, 1590.8412, 1608.8254, 1655.8672, 1671.9061, 1683.9292, 1685.9320, 1738.8187, 1755.8503, 1769.8682, 1800.0060, 1853.9751, 1883.9473, 1889.0319, 1958.7950, 1972.8079, 2189.1829, 2227.1101, 2247.1912, 2340.2273, 2354.2493, 2402.2341, 2416.2493, 2452.1997, 2468.3242, 2771.4365, 2890.5337, 2899.5171, 3211.4919, 3254.7114, 3270.6653, 3284.6660

15. KAP2_BOVIN **Mass:** 45408 **Score:** 29 **Expect:** 88 **Matches:** 6

cAMP-dependent protein kinase type II-alpha regulatory subunit OS=Bos taurus GN=PRKAR2A PE=1 SV=2

Observed	Mr(expt)	Mr(calc)	ppm	Start	End	Miss	Peptide
1245.5985	1244.5912	1244.6361	-36.03	281 -	291	1	K.DGERIITQGEK.A
1555.8271	1554.8198	1554.8004	12.5	248 -	260	0	K.MFESFIESVPLLK.S + Oxidation (M)
1683.9292	1682.9219	1682.8953	15.8	247 -	260	1	R.KMFESFIESVPLLK.S + Oxidation (M)
1883.9473	1882.9400	1882.9928	-28.01	292 -	308	0	K.ADSFYIIESGEVSILIK.S
2340.2273	2339.2200	2339.2083	5.03	248 -	267	1	K.MFESFIESVPLLKSLEVSER.M
2416.2493	2415.2420	2415.1563	35.5	345 -	366	1	R.AASAYAVGDVKCLVMDVQAFER.L + Oxidation (M)

No match to: 850.2828, 854.0068, 855.0179, 855.9982, 857.0005, 859.9986, 861.2448, 923.3679, 940.3949, 954.4086, 1020.5010, 1044.0396, 1045.0438, 1063.5172, 1080.5172, 1096.4980, 1097.4967, 1167.6447, 1185.6573, 1236.6335, 1300.0328, 1328.5688, 1354.6420, 1446.8004, 1462.7750, 1480.8190, 1497.7250, 1505.8336, 1511.7294, 1551.7411, 1590.8412, 1608.8254, 1655.8672, 1671.9061, 1685.9320, 1738.8187, 1755.8503, 1769.8682, 1800.0060, 1853.9751, 1889.0319, 1958.7950, 1972.8079, 2189.1829, 2227.1101, 2247.1912, 2351.2632, 2354.2493, 2402.2341, 2452.1997, 2468.3242, 2482.3257, 2771.4365, 2890.5337, 2899.5171, 3211.4919, 3254.7114, 3270.6653, 3284.6660

16. PCID2_BOVIN **Mass:** 47410 **Score:** 29 **Expect:** 88 **Matches:** 6

PCI domain-containing protein 2 OS=Bos taurus GN=PCID2 PE=2 SV=1

Observed	Mr(expt)	Mr(calc)	ppm	Start	End	Miss	Peptide
1097.4967	1096.4894	1096.5414	-47.38	384 -	392	0	K.GYISHQHQK.L
1167.6447	1166.6374	1166.6812	-37.48	319 -	327	1	K.IITYRNLFK.K
1236.6335	1235.6262	1235.6398	-10.95	21 -	32	0	R.DGASLAELVSFK.H
1555.8271	1554.8198	1554.8262	-4.09	260 -	272	0	K.MLLGHMPTIELLR.K + 2 Oxidation (M)
1683.9292	1682.9219	1682.9211	0.46	260 -	273	1	K.MLLGHMPTIELLRK.Y + 2 Oxidation (M)
1972.8079	1971.8006	1971.8965	-48.61	141 -	157	1	K.AAELLMGCFRVCASDTR.A + Oxidation (M)

No match to: 850.2828, 854.0068, 855.0179, 855.9982, 857.0005, 859.9986, 861.2448, 923.3679, 940.3949, 954.4086, 1020.5010, 1044.0396, 1045.0438, 1063.5172, 1080.5172, 1096.4980, 1185.6573, 1245.5985, 1300.0328, 1328.5688, 1354.6420, 1446.8004, 1462.7750, 1480.8190, 1497.7250, 1505.8336, 1511.7294, 1551.7411, 1590.8412, 1608.8254, 1655.8672, 1671.9061, 1685.9320, 1738.8187, 1755.8503, 1769.8682, 1800.0060, 1853.9751, 1883.9473, 1889.0319, 1958.7950, 2189.1829, 2227.1101, 2247.1912, 2340.2273, 2351.2632, 2354.2493, 2402.2341, 2416.2493, 2452.1997, 2468.3242, 2482.3257, 2771.4365, 2890.5337, 2899.5171, 3211.4919, 3254.7114, 3270.6653, 3284.6660

17. HMCS1_RAT **Mass:** 58025 **Score:** 29 **Expect:** 88 **Matches:** 6

Hydroxymethylglutaryl-CoA synthase, cytoplasmic OS=Rattus norvegicus GN=Hmgcs1 PE=1 SV=1

Observed	Mr(expt)	Mr(calc)	ppm	Start	End	Miss	Peptide
1020.5010	1019.4937	1019.5321	-37.66	401 -	409	0	K.ITASLCDLK.S
1236.6335	1235.6262	1235.6510	-20.03	322 -	332	1	K.ASAELFNQKTK.A
1497.7250	1496.7177	1496.6640	35.9	416 -	428	0	R.TCVAPDVFAENMK.L + Oxidation (M)
1769.8682	1768.8609	1768.8203	23.0	278 -	291	1	R.MFLNDFLNDQNRDK.N
2402.2341	2401.2268	2401.1076	49.6	56 -	75	1	R.MGFCTDREDINSLCLTVVQK.L + Oxidation (M)
3270.6653	3269.6580	3269.5274	40.0	192 -	219	1	R.GLRGTHMQHAYDFYKPDMLSEYPVVDGK.L + Oxidation (M)

No match to: 850.2828, 854.0068, 855.0179, 855.9982, 857.0005, 859.9986, 861.2448, 923.3679, 940.3949, 954.4086, 1044.0396, 1045.0438, 1063.5172, 1080.5172, 1096.4980, 1097.4967, 1167.6447, 1185.6573, 1245.5985, 1300.0328, 1328.5688, 1354.6420, 1446.8004, 1462.7750, 1480.8190, 1505.8336, 1511.7294, 1551.7411, 1555.8271, 1590.8412, 1608.8254, 1655.8672, 1671.9061, 1683.9292, 1685.9320, 1738.8187, 1755.8503, 1800.0060, 1853.9751, 1883.9473, 1889.0319, 1958.7950, 1972.8079, 2189.1829, 2227.1101, 2247.1912, 2340.2273, 2351.2632, 2354.2493, 2416.2493, 2452.1997, 2468.3242, 2482.3257, 2771.4365, 2890.5337, 2899.5171, 3211.4919, 3254.7114, 3284.6660

18. TM242_MOUSE **Mass:** 14964 **Score:** 29 **Expect:** 90 **Matches:** 3
Transmembrane protein 242 OS=Mus musculus GN=Tmem242 PE=2 SV=1

Observed	Mr(expt)	Mr(calc)	ppm	Start	End	Miss	Peptide
2340.2273	2339.2200	2339.2559	-15.34	29 -	53	0	K.GGIFLGSAAAAGMLAGFVTTLSLAK.K + Oxidation (M)
2468.3242	2467.3169	2467.3509	-13.75	29 -	54	1	K.GGIFLGSAAAAGMLAGFVTTLSLAKK.K + Oxidation (M)
2771.4365	2770.4292	2770.3297	35.9	2 -	28	1	M.ETSGPGPGESSELEAPGSPDDRLFLVK.G

No match to: 850.2828, 854.0068, 855.0179, 855.9982, 857.0005, 859.9986, 861.2448, 923.3679, 940.3949, 954.4086, 1020.5010, 1044.0396, 1045.0438, 1063.5172, 1080.5172, 1096.4980, 1097.4967, 1167.6447, 1185.6573, 1236.6335, 1245.5985, 1300.0328, 1328.5688, 1354.6420, 1446.8004, 1462.7750, 1480.8190, 1497.7250, 1505.8336, 1511.7294, 1551.7411, 1555.8271, 1590.8412, 1608.8254, 1655.8672, 1671.9061, 1683.9292, 1685.9320, 1738.8187, 1755.8503, 1769.8682, 1800.0060, 1853.9751, 1883.9473, 1889.0319, 1958.7950, 1972.8079, 2189.1829, 2227.1101, 2247.1912, 2351.2632, 2354.2493, 2402.2341, 2416.2493, 2452.1997, 2482.3257, 2890.5337, 2899.5171, 3211.4919, 3254.7114, 3270.6653, 3284.6660

19. ST2A2_MOUSE **Mass:** 33422 **Score:** 28 **Expect:** 98 **Matches:** 5
Bile salt sulfotransferase 2 OS=Mus musculus GN=Sult2a2 PE=2 SV=1

Observed	Mr(expt)	Mr(calc)	ppm	Start	End	Miss	Peptide
1328.5688	1327.5615	1327.6078	-34.84	225 -	235	0	K.ENNMSNYSLIK.E + Oxidation (M)
1505.8336	1504.8263	1504.8766	-33.39	95 -	107	0	R.LITSHLPIHLFSK.S
2189.1829	2188.1756	2188.2045	-13.17	95 -	113	1	R.LITSHLPIHLFSKSFFSSK.A
2340.2273	2339.2200	2339.2063	5.89	122 -	141	1	R.NPRDILVSGYFFWGNTNLVK.N
2452.1997	2451.1924	2451.1028	36.6	1 -	20	0	-.MMSDYNWFEGIPFPAISYQR.E

No match to: 850.2828, 854.0068, 855.0179, 855.9982, 857.0005, 859.9986, 861.2448, 923.3679, 940.3949, 954.4086, 1020.5010, 1044.0396, 1045.0438, 1063.5172, 1080.5172, 1096.4980, 1097.4967, 1167.6447, 1185.6573, 1236.6335, 1245.5985, 1300.0328, 1354.6420, 1446.8004, 1462.7750, 1480.8190, 1497.7250, 1511.7294, 1551.7411, 1555.8271, 1590.8412, 1608.8254, 1655.8672, 1671.9061, 1683.9292, 1685.9320, 1738.8187, 1755.8503, 1769.8682, 1800.0060, 1853.9751, 1883.9473, 1889.0319, 1958.7950, 1972.8079, 2227.1101, 2247.1912, 2351.2632, 2354.2493, 2402.2341, 2416.2493, 2468.3242, 2482.3257, 2771.4365, 2890.5337, 2899.5171, 3211.4919, 3254.7114, 3270.6653, 3284.6660

20. SUH2_RAT **Mass:** 33432 **Score:** 28 **Expect:** 98 **Matches:** 5
Probable alcohol sulfotransferase OS=Rattus norvegicus PE=2 SV=2

Observed	Mr(expt)	Mr(calc)	ppm	Start	End	Miss	Peptide
1328.5688	1327.5615	1327.6078	-34.84	225 -	235	0	K.ENNMSNYSLIK.E + Oxidation (M)
1505.8336	1504.8263	1504.8766	-33.39	95 -	107	0	R.LITSHLPIHLFSK.S
2189.1829	2188.1756	2188.2045	-13.17	95 -	113	1	R.LITSHLPIHLFSKSFFSSK.A
2340.2273	2339.2200	2339.2063	5.89	122 -	141	1	R.NPRDILVSGYFFWGNTNLVK.N
2452.1997	2451.1924	2451.1028	36.6	1 -	20	0	-.MMSDYNWFEGIPFPAISYQR.E

No match to: 850.2828, 854.0068, 855.0179, 855.9982, 857.0005, 859.9986, 861.2448, 923.3679, 940.3949, 954.4086, 1020.5010, 1044.0396, 1045.0438, 1063.5172, 1080.5172, 1096.4980, 1097.4967, 1167.6447, 1185.6573, 1236.6335, 1245.5985, 1300.0328, 1354.6420, 1446.8004, 1462.7750, 1480.8190, 1497.7250, 1511.7294, 1551.7411, 1555.8271, 1590.8412, 1608.8254, 1655.8672, 1671.9061, 1683.9292, 1685.9320, 1738.8187, 1755.8503, 1769.8682, 1800.0060, 1853.9751, 1883.9473, 1889.0319, 1958.7950, 1972.8079, 2227.1101, 2247.1912, 2351.2632, 2354.2493, 2402.2341, 2416.2493, 2468.3242, 2482.3257, 2771.4365, 2890.5337, 2899.5171, 3211.4919, 3254.7114, 3270.6653, 3284.6660

Search Parameters

Type of search : Peptide Mass Fingerprint
Enzyme : Trypsin
Fixed modifications : Carbamidomethyl (C)
Variable modifications : Oxidation (M)
Mass values : Monoisotopic
Protein Mass : Unrestricted
Peptide Mass Tolerance : ± 50 ppm
Peptide Charge State : 1+
Max Missed Cleavages : 1
Number of queries : 65

Mascot: http://www.matrixscience.com/
