# Supplementary material for: Vasectomy and Photoperiodic Regimen Modify the Protein Profile, Hormonal Content and Antioxidant Enzymes Activity of Ram Seminal Plasma
Source: Int J Mol Sci. 2020 Oct 29;21(21):8063. doi: 10.3390/ijms21218063 (PMC7663742; doi:10.3390/ijms21218063)
Supplement: Supplementary file 1 [file ijms-21-08063-s001.zip › Supplementary file 2_Mascot protein identification/Phosphoglycerate kinase identification.pdf]

Mascot Search Results

Protein View

Match to: **PGK2_HORSE** Score: **237** Expect: **1.3e-019**
Phosphoglycerate kinase 2 OS=Equus caballus GN=PGK2 PE=3 SV=3

Nominal mass (M_r): **45249**; Calculated pI value: **8.62**
NCBI BLAST search of PGK2_HORSE against nr
Unformatted sequence string for pasting into other applications

Taxonomy: Equus caballus

Fixed modifications: Carbamidomethyl (C)
Variable modifications: Oxidation (M)
Cleavage by Trypsin: cuts C-term side of KR unless next residue is P
Sequence Coverage: **34%**

Matched peptides shown in **Bold Red**

```
  1 MSLSKKLTLD KLDVKGKRII MRVDFNVPMK KNQITNNQRI KASIPSIKYC
 51 LDNGARSVVL MSHLGRPDGV PMPDKYSLEP VAAELKSLLG KDVLFLKDCV
101 GSEVEKACAN PATGSVILLE NLRFHVEEEG KGQDPSGNKL KAEAGKIEAF
151 RASLSKLGDV YVNDAFGTAH RAHSSMVGIN LPQKASGFLM KKELEYFAKA
201 LENPERPFLA ILGGAKVADK IQLIKNMLDK VNEMIIGGGM AYTFLKVLNN
251 MEIGASLFDE EGAKIVKDIM AKANKNGVRI TFPVDFVTAD KFEENAKVGQ
301 ATVASGIPAG WMGLDCGPET NKKYAQVMAQ AKLIVWNGPV GVFEWDAFAK
351 GTKALMDEIV KATSRGCITI IGGGDTATCC AKWNTEDKVS HVSTGGGASL
401 ELLEGKILPG VDALSNL
```

Show predicted peptides also

Sort Peptides By ◉ Residue Number ○ Increasing Mass ○ Decreasing Mass

Start - End	Observed	Mr(expt)	Mr(calc)	ppm	Miss	Sequence
32 - 39	987.4899	986.4826	986.4893	-7	0	K.NQITNNQR.I (No match)
87 - 97	1232.7129	1231.7056	1231.7540	-39	1	K.SLLGKDVLFLK.D (No match)
124 - 139	1757.8077	1756.8004	1756.8016	-1	1	R.FHVEEEGKGQDPSGNK.L (No match)
157 - 171	1634.7924	1633.7851	1633.7849	0	0	K.LGDVYVNDAFGTAHR.A (No match)
157 - 171	1634.7924	1633.7851	1633.7849	0	0	K.LGDVYVNDAFGTAHR.A (Ions score 116)
200 - 216	1796.0061	1794.9988	1794.9992	-0	0	K.ALENPERPFLAILGGAK.V (No match)
200 - 216	1796.0061	1794.9988	1794.9992	-0	0	K.ALENPERPFLAILGGAK.V (Ions score 73)
231 - 246	1759.8289	1758.8216	1758.8685	-27	0	K.VNEMIIGGGMAYTFLK.V Oxidation (M) (No match
247 - 264	1936.9341	1935.9268	1935.9248	1	0	K.VLNNMEIGASLFDEEGAK.I (No match)
247 - 264	1952.9316	1951.9243	1951.9197	2	0	K.VLNNMEIGASLFDEEGAK.I Oxidation (M) (No mat
333 - 350	2048.0571	2047.0498	2047.0567	-3	0	K.LIVWNGPVGVFEWDAFAK.G (No match)
383 - 406	2514.2427	2513.2354	2513.2398	-2	1	K.WNTEDKVSHVSTGGGASLELLEGK.I (No match)

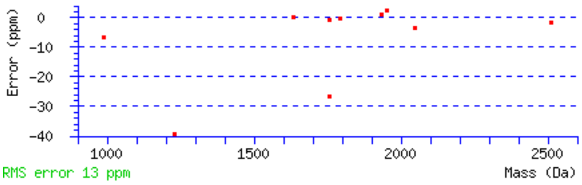

Mascot Search Results

User	:	
Email	:	
Search title	:	SampleSetID: 611, AnalysisID: 4512, MaldiWellID: 55967, SpectrumID: 109855, Path=\160212\MSMS\16-13 Jose Alvaro
Database	:	SwissProt sprot_160208 (550116 sequences; 196219159 residues)
Taxonomy	:	Mammalia (mammals) (66429 sequences)
Timestamp	:	12 Feb 2016 at 11:36:38 GMT
Warning	:	A Peptide summary report will usually give a much clearer picture of MS/MS search results.
Top Score	:	237 for PGK2_HORSE, Phosphoglycerate kinase 2 OS=Equus caballus GN=PGK2 PE=3 SV=3

Mascot Score Histogram

Protein score is -10*Log(P), where P is the probability that the observed match is a random event.
Protein scores greater than 61 are significant (p<0.05).
Protein scores are derived from ions scores as a non-probabilistic basis for ranking protein hits.

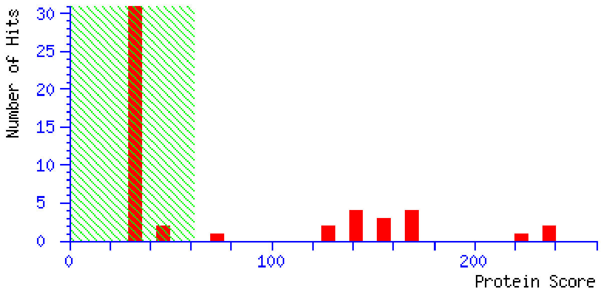

Protein Summary Report

Format As | Protein Summary (deprecated) | Help

Significance threshold p< 0.05 Max. number of hits 20

Re-Search All | Search Unmatched

Index

	Accession	Mass	Score	Description
1.	PGK2_HORSE	45249	237	Phosphoglycerate kinase 2 OS=Equus caballus GN=PGK2 PE=3 SV=3
2.	PGK2_HUMAN	45166	234	Phosphoglycerate kinase 2 OS=Homo sapiens GN=PGK2 PE=1 SV=3
3.	PGK2_PIG	45266	229	Phosphoglycerate kinase 2 OS=Sus scrofa GN=PGK2 PE=2 SV=3
4.	PGK1_HUMAN	44985	163	Phosphoglycerate kinase 1 OS=Homo sapiens GN=PGK1 PE=1 SV=3
5.	PGK1_PANTR	44985	163	Phosphoglycerate kinase 1 OS=Pan troglodytes GN=PGK1 PE=2 SV=1
6.	PGK1_MACFA	44957	163	Phosphoglycerate kinase 1 OS=Macaca fascicularis GN=PGK1 PE=2 SV=3
7.	PGK1_PONAB	44957	163	Phosphoglycerate kinase 1 OS=Pongo abelii GN=PGK1 PE=2 SV=4
8.	PGK1_HORSE	44973	155	Phosphoglycerate kinase 1 OS=Equus caballus GN=PGK1 PE=1 SV=2
9.	PGK1_BOVIN	44908	154	Phosphoglycerate kinase 1 OS=Bos taurus GN=PGK1 PE=2 SV=3
10.	PGK2_MACFA	45235	152	Phosphoglycerate kinase 2 OS=Macaca fascicularis GN=PGK2 PE=2 SV=1
11.	PGK1_CRIGR	44933	148	Phosphoglycerate kinase 1 OS=Cricetulus griseus GN=PGK1 PE=2 SV=2
12.	PGK1_MOUSE	44921	148	Phosphoglycerate kinase 1 OS=Mus musculus GN=Pgk1 PE=1 SV=4
13.	PGK1_RAT	44909	148	Phosphoglycerate kinase 1 OS=Rattus norvegicus GN=Pgk1 PE=1 SV=2
14.	PGK1_PIG	44929	148	Phosphoglycerate kinase 1 OS=Sus scrofa GN=PGK1 PE=1 SV=3
15.	PGK1_MACEU	45405	133	Phosphoglycerate kinase 1 OS=Macropus eugenii GN=PGK1 PE=2 SV=2
16.	PGK2_MOUSE	45223	127	Phosphoglycerate kinase 2 OS=Mus musculus GN=Pgk2 PE=1 SV=4
17.	NEBU_HUMAN	775393	79	Nebulin OS=Homo sapiens GN=NEB PE=1 SV=5
18.	CC171_HUMAN	154083	50	Coiled-coil domain-containing protein 171 OS=Homo sapiens GN=CCDC171 PE=2 SV=1
19.	CE034_BOVIN	73735	40	Uncharacterized protein C5orf34 homolog OS=Bos taurus PE=2 SV=1
20.	LDHA_PANTR	36924	39	L-lactate dehydrogenase A chain OS=Pan troglodytes GN=LDHA PE=2 SV=3

Results List

1. PGK2_HORSE **Mass:** 45249 **Score:** 237 **Expect:** 1.3e-019 **Matches:** 12
Phosphoglycerate kinase 2 OS=Equus caballus GN=PGK2 PE=3 SV=3

Observed	Mr(expt)	Mr(calc)	ppm	Start	End	Miss	Ions	Peptide
987.4899	986.4826	986.4893	-6.80	32 -	39	0	---	K.NQITNNQR.I
1232.7129	1231.7056	1231.7540	-39.27	87 -	97	1	---	K.SLLGKDVLFLK.D
1634.7924	1633.7851	1633.7849	0.15	157 -	171	0	---	K.LGDVYVNDAFGTAHR.A
1634.7924	1633.7851	1633.7849	0.15	157 -	171	0	116	K.LGDVYVNDAFGTAHR.A
1757.8077	1756.8004	1756.8016	-0.69	124 -	139	1	---	R.FHVEEEGKGQDPSGNK.L
1759.8289	1758.8216	1758.8685	-26.63	231 -	246	0	---	K.VNEMIIGGGMAYTFLK.V + Oxidation (M)
1796.0061	1794.9988	1794.9992	-0.20	200 -	216	0	---	K.ALENPERPFLAILGGAK.V
1796.0061	1794.9988	1794.9992	-0.20	200 -	216	0	73	K.ALENPERPFLAILGGAK.V
1936.9341	1935.9268	1935.9248	1.07	247 -	264	0	---	K.VLNNMEIGASLFDEEGAK.I
1952.9316	1951.9243	1951.9197	2.38	247 -	264	0	---	K.VLNNMEIGASLFDEEGAK.I + Oxidation (M)
2048.0571	2047.0498	2047.0567	-3.38	333 -	350	0	---	K.LIVWNGPVGVFEWDAFAK.G

2514.2427	2513.2354	2513.2398	-1.74	383	406	1	---	K.WNTEDKVSHVSTGGGASLELLEGK.I

No match to: 855.0386, 856.0210, 858.2581, 860.0115, 863.5002, 873.0222, 875.2742, 881.2588, 885.4301, 917.2859, 951.2886, 988.4872, 1014.4448, 1037.5166, 1044.0621, 1118.5048, 1134.5503, 1136.5503, 1175.6055, 1175.6055, 1189.6184, 1248.5896, 1300.0398, 1320.6007, 1367.7023, 1375.6530, 1376.6458, 1399.7317, 1416.7859, 1421.6720, 1431.6903, 1434.7605, 1458.7081, 1473.8019, 1475.7791, 1482.8335, 1616.8318, 1691.8187, 1720.8691, 1739.7987, 1747.8970, 1756.7877, 1768.8522, 1810.0177, 1812.9647, 1812.9647, 1826.9792, 1835.8917, 1853.0256, 1868.0201, 1888.9293, 2198.1147, 2225.1714, 2246.1958, 2289.1677, 2485.2241, 3211.4470

2. PGK2_HUMAN **Mass:** 45166 **Score:** 234 **Expect:** 2.6e-019 **Matches:** 13

Phosphoglycerate kinase 2 OS=Homo sapiens GN=PGK2 PE=1 SV=3

Observed	Mr(expt)	Mr(calc)	ppm	Start	End	Miss	Ions	Peptide
885.4301	884.4228	884.4280	-5.81	193	199	0	---	K.ELDYFAK.A
987.4899	986.4826	986.4893	-6.80	32	39	0	---	K.NQITNNQR.I
1175.6055	1174.5982	1174.5982	0.02	142	151	1	---	K.AEPDKIEAFR.A
1175.6055	1174.5982	1174.5982	0.02	142	151	1	69	K.AEPDKIEAFR.A
1189.6184	1188.6111	1188.6754	-54.07	76	86	0	---	K.YSLAPVAVELK.S
1232.7129	1231.7056	1231.7540	-39.27	87	97	1	---	K.SLLGKDVLFLK.D
1376.6458	1375.6385	1375.7030	-46.90	172	184	0	---	R.AHSSMVGVNLPHK.A
1634.7924	1633.7851	1633.7849	0.15	157	171	0	---	K.LGDVYVNDAFGTAHR.A
1634.7924	1633.7851	1633.7849	0.15	157	171	0	116	K.LGDVYVNDAFGTAHR.A
1759.8289	1758.8216	1758.8685	-26.63	231	246	0	---	K.VNEMIIGGGMAYTFLK.V + Oxidation (M)
1936.9341	1935.9268	1935.9248	1.07	247	264	0	---	K.VLNNMEIGASLFDEEGAK.I
1952.9316	1951.9243	1951.9197	2.38	247	264	0	---	K.VLNNMEIGASLFDEEGAK.I + Oxidation (M)
2514.2427	2513.2354	2513.2398	-1.74	383	406	1	---	K.WNTEDKVSHVSTGGGASLELLEGK.I

No match to: 855.0386, 856.0210, 858.2581, 860.0115, 863.5002, 873.0222, 875.2742, 881.2588, 917.2859, 951.2886, 988.4872, 1014.4448, 1037.5166, 1044.0621, 1118.5048, 1134.5503, 1136.5503, 1248.5896, 1300.0398, 1320.6007, 1367.7023, 1375.6530, 1399.7317, 1416.7859, 1421.6720, 1431.6903, 1434.7605, 1458.7081, 1473.8019, 1475.7791, 1482.8335, 1616.8318, 1691.8187, 1720.8691, 1739.7987, 1747.8970, 1756.7877, 1757.8077, 1768.8522, 1796.0061, 1796.0061, 1810.0177, 1812.9647, 1812.9647, 1826.9792, 1835.8917, 1853.0256, 1868.0201, 1888.9293, 2048.0571, 2198.1147, 2225.1714, 2246.1958, 2289.1677, 2485.2241, 3211.4470

3. PGK2_PIG **Mass:** 45266 **Score:** 229 **Expect:** 8.4e-019 **Matches:** 10

Phosphoglycerate kinase 2 OS=Sus scrofa GN=PGK2 PE=2 SV=3

Observed	Mr(expt)	Mr(calc)	ppm	Start	End	Miss	Ions	Peptide
885.4301	884.4228	884.4280	-5.81	193	199	0	---	K.ELDYFAK.A
1232.7129	1231.7056	1231.7540	-39.27	87	97	1	---	K.SLLGKDVLFLK.D
1367.7023	1366.6950	1366.7027	-5.63	172	184	0	---	R.AHSSMVGVNLPQK.A
1634.7924	1633.7851	1633.7849	0.15	157	171	0	---	K.LGDVYVNDAFGTAHR.A
1634.7924	1633.7851	1633.7849	0.15	157	171	0	116	K.LGDVYVNDAFGTAHR.A
1757.8077	1756.8004	1756.8016	-0.69	124	139	1	---	R.FHVEEEGKGQDPSGNK.L
1759.8289	1758.8216	1758.8685	-26.63	231	246	0	---	K.VNEMIIGGGMAFTFLK.V + 2 Oxidation (M)
1796.0061	1794.9988	1794.9992	-0.20	200	216	0	---	K.ALENPERPFLAILGGAK.V
1796.0061	1794.9988	1794.9992	-0.20	200	216	0	73	K.ALENPERPFLAILGGAK.V
2514.2427	2513.2354	2513.2398	-1.74	383	406	1	---	K.WNTEDKVSHVSTGGGASLELLEGK.V

No match to: 855.0386, 856.0210, 858.2581, 860.0115, 863.5002, 873.0222, 875.2742, 881.2588, 917.2859, 951.2886, 987.4899, 988.4872, 1014.4448, 1037.5166, 1044.0621, 1118.5048, 1134.5503, 1136.5503, 1175.6055, 1175.6055, 1189.6184, 1248.5896, 1300.0398, 1320.6007, 1375.6530, 1376.6458, 1399.7317, 1416.7859, 1421.6720, 1431.6903, 1434.7605, 1458.7081, 1473.8019, 1475.7791, 1482.8335, 1616.8318, 1691.8187, 1720.8691, 1739.7987, 1747.8970, 1756.7877, 1768.8522, 1810.0177, 1812.9647, 1812.9647, 1826.9792, 1835.8917, 1853.0256, 1868.0201, 1888.9293, 1936.9341, 1952.9316, 2048.0571, 2198.1147, 2225.1714, 2246.1958, 2289.1677, 2485.2241, 3211.4470

4. PGK1_HUMAN **Mass:** 44985 **Score:** 163 **Expect:** 3.3e-012 **Matches:** 10

Phosphoglycerate kinase 1 OS=Homo sapiens GN=PGK1 PE=1 SV=3

Observed	Mr(expt)	Mr(calc)	ppm	Start	End	Miss	Ions	Peptide
1232.7129	1231.7056	1231.7540	-39.27	87	97	1	---	K.SLLGKDVLFLK.D
1367.7023	1366.6950	1366.7027	-5.63	172	184	0	---	R.AHSSMVGVNLPQK.A
1434.7605	1433.7532	1433.7523	0.63	19	30	1	---	R.VVMRVDFNVPMK.N
1634.7924	1633.7851	1633.7849	0.15	157	171	0	---	K.LGDVYVNDAFGTAHR.A
1634.7924	1633.7851	1633.7849	0.15	157	171	0	116	K.LGDVYVNDAFGTAHR.A
1747.8970	1746.8897	1746.8862	2.00	92	106	1	---	K.DVLFLKDCVGPEVEK.A
1759.8289	1758.8216	1758.8685	-26.63	231	246	0	---	K.VNEMIIGGGMAFTFLK.V + 2 Oxidation (M)
1768.8522	1767.8449	1767.9301	-48.19	107	123	0	---	K.ACANPAAGSVILLENLR.F
2048.0571	2047.0498	2046.9905	29.0	23	39	1	---	R.VDFNVPMKNNQITNNQR.I + Oxidation (M)
2514.2427	2513.2354	2513.2398	-1.74	383	406	1	---	K.WNTEDKVSHVSTGGGASLELLEGK.V

No match to: 855.0386, 856.0210, 858.2581, 860.0115, 863.5002, 873.0222, 875.2742, 881.2588, 885.4301, 917.2859, 951.2886, 987.4899, 988.4872, 1014.4448, 1037.5166, 1044.0621, 1118.5048, 1134.5503, 1136.5503, 1175.6055, 1175.6055, 1189.6184, 1248.5896, 1300.0398, 1320.6007, 1375.6530, 1376.6458, 1399.7317, 1416.7859, 1421.6720, 1431.6903, 1458.7081, 1473.8019, 1475.7791, 1482.8335, 1616.8318, 1691.8187, 1720.8691, 1739.7987, 1756.7877, 1757.8077, 1796.0061, 1796.0061, 1810.0177, 1812.9647, 1812.9647, 1826.9792, 1835.8917, 1853.0256, 1868.0201, 1888.9293, 1936.9341, 1952.9316, 2198.1147, 2225.1714, 2246.1958, 2289.1677, 2485.2241, 3211.4470

5. PGK1_PANTR **Mass:** 44985 **Score:** 163 **Expect:** 3.3e-012 **Matches:** 10

Phosphoglycerate kinase 1 OS=Pan troglodytes GN=PGK1 PE=2 SV=1

Observed	Mr(expt)	Mr(calc)	ppm	Start	End	Miss	Ions	Peptide
1232.7129	1231.7056	1231.7540	-39.27	87	97	1	---	K.SLLGKDVLFLK.D
1367.7023	1366.6950	1366.7027	-5.63	172	184	0	---	R.AHSSMVGVNLPQK.A
1434.7605	1433.7532	1433.7523	0.63	19	30	1	---	R.VVMRVDFNVPMK.N
1634.7924	1633.7851	1633.7849	0.15	157	171	0	---	K.LGDVYVNDAFGTAHR.A

1634.7924	1633.7851	1633.7849	0.15	157	171	0	116	K.LGDVYVNDAFGTAHR.A
1747.8970	1746.8897	1746.8862	2.00	92	106	1	---	K.DVLFLKDCVGPEVEK.A
1759.8289	1758.8216	1758.8685	-26.63	231	246	0	---	K.VNEMIIGGGMAFTFLK.V + 2 Oxidation (M)
1768.8522	1767.8449	1767.9301	-48.19	107	123	0	---	K.ACANPAAGSVILLENLR.F
2048.0571	2047.0498	2046.9905	29.0	23	39	1	---	R.VDFNVPMKNNQITNNQR.I + Oxidation (M)
2514.2427	2513.2354	2513.2398	-1.74	383	406	1	---	K.WNTEDKVSHVSTGGGASLELLEGK.V

No match to: 855.0386, 856.0210, 858.2581, 860.0115, 863.5002, 873.0222, 875.2742, 881.2588, 885.4301, 917.2859, 951.2886, 987.4899, 988.4872, 1014.4448, 1037.5166, 1044.0621, 1118.5048, 1134.5503, 1136.5503, 1175.6055, 1175.6055, 1189.6184, 1248.5896, 1300.0398, 1320.6007, 1375.6530, 1376.6458, 1399.7317, 1416.7859, 1421.6720, 1431.6903, 1458.7081, 1473.8019, 1475.7791, 1482.8335, 1616.8318, 1691.8187, 1720.8691, 1739.7987, 1756.7877, 1757.8077, 1796.0061, 1796.0061, 1810.0177, 1812.9647, 1812.9647, 1826.9792, 1835.8917, 1853.0256, 1868.0201, 1888.9293, 1936.9341, 1952.9316, 2198.1147, 2225.1714, 2246.1958, 2289.1677, 2485.2241, 3211.4470

6. PGK1_MACFA **Mass:** 44957 **Score:** 163 **Expect:** 3.3e-012 **Matches:** 10

Phosphoglycerate kinase 1 OS=Macaca fascicularis GN=PGK1 PE=2 SV=3

Observed	Mr(expt)	Mr(calc)	ppm	Start	End	Miss	Ions	Peptide
1232.7129	1231.7056	1231.7540	-39.27	87	97	1	---	K.SLLGKDVLFLK.D
1367.7023	1366.6950	1366.7027	-5.63	172	184	0	---	R.AHSSMVGVNLPQK.A
1434.7605	1433.7532	1433.7523	0.63	19	30	1	---	R.VVMRVDFNVPMK.N
1634.7924	1633.7851	1633.7849	0.15	157	171	0	---	K.LGDVYVNDAFGTAHR.A
1634.7924	1633.7851	1633.7849	0.15	157	171	0	116	K.LGDVYVNDAFGTAHR.A
1747.8970	1746.8897	1746.8862	2.00	92	106	1	---	K.DVLFLKDCVGPEVEK.A
1759.8289	1758.8216	1758.8685	-26.63	231	246	0	---	K.VNEMIIGGGMAFTFLK.V + 2 Oxidation (M)
1768.8522	1767.8449	1767.9301	-48.19	107	123	0	---	K.ACANPAAGSVILLENLR.F
2048.0571	2047.0498	2046.9905	29.0	23	39	1	---	R.VDFNVPMKNNQITNNQR.I + Oxidation (M)
2514.2427	2513.2354	2513.2398	-1.74	383	406	1	---	K.WNTEDKVSHVSTGGGASLELLEGK.V

No match to: 855.0386, 856.0210, 858.2581, 860.0115, 863.5002, 873.0222, 875.2742, 881.2588, 885.4301, 917.2859, 951.2886, 987.4899, 988.4872, 1014.4448, 1037.5166, 1044.0621, 1118.5048, 1134.5503, 1136.5503, 1175.6055, 1175.6055, 1189.6184, 1248.5896, 1300.0398, 1320.6007, 1375.6530, 1376.6458, 1399.7317, 1416.7859, 1421.6720, 1431.6903, 1458.7081, 1473.8019, 1475.7791, 1482.8335, 1616.8318, 1691.8187, 1720.8691, 1739.7987, 1756.7877, 1757.8077, 1796.0061, 1796.0061, 1810.0177, 1812.9647, 1812.9647, 1826.9792, 1835.8917, 1853.0256, 1868.0201, 1888.9293, 1936.9341, 1952.9316, 2198.1147, 2225.1714, 2246.1958, 2289.1677, 2485.2241, 3211.4470

7. PGK1_PONAB **Mass:** 44957 **Score:** 163 **Expect:** 3.3e-012 **Matches:** 10

Phosphoglycerate kinase 1 OS=Pongo abelii GN=PGK1 PE=2 SV=4

Observed	Mr(expt)	Mr(calc)	ppm	Start	End	Miss	Ions	Peptide
1232.7129	1231.7056	1231.7540	-39.27	87	97	1	---	K.SLLGKDVLFLK.D
1367.7023	1366.6950	1366.7027	-5.63	172	184	0	---	R.AHSSMVGVNLPQK.A
1434.7605	1433.7532	1433.7523	0.63	19	30	1	---	R.VVMRVDFNVPMK.N
1634.7924	1633.7851	1633.7849	0.15	157	171	0	---	K.LGDVYVNDAFGTAHR.A
1634.7924	1633.7851	1633.7849	0.15	157	171	0	116	K.LGDVYVNDAFGTAHR.A
1747.8970	1746.8897	1746.8862	2.00	92	106	1	---	K.DVLFLKDCVGPEVEK.A
1759.8289	1758.8216	1758.8685	-26.63	231	246	0	---	K.VNEMIIGGGMAFTFLK.V + 2 Oxidation (M)
1768.8522	1767.8449	1767.9301	-48.19	107	123	0	---	K.ACANPAAGSVILLENLR.F
2048.0571	2047.0498	2046.9905	29.0	23	39	1	---	R.VDFNVPMKNNQITNNQR.I + Oxidation (M)
2514.2427	2513.2354	2513.2398	-1.74	383	406	1	---	K.WNTEDKVSHVSTGGGASLELLEGK.V

No match to: 855.0386, 856.0210, 858.2581, 860.0115, 863.5002, 873.0222, 875.2742, 881.2588, 885.4301, 917.2859, 951.2886, 987.4899, 988.4872, 1014.4448, 1037.5166, 1044.0621, 1118.5048, 1134.5503, 1136.5503, 1175.6055, 1175.6055, 1189.6184, 1248.5896, 1300.0398, 1320.6007, 1375.6530, 1376.6458, 1399.7317, 1416.7859, 1421.6720, 1431.6903, 1458.7081, 1473.8019, 1475.7791, 1482.8335, 1616.8318, 1691.8187, 1720.8691, 1739.7987, 1756.7877, 1757.8077, 1796.0061, 1796.0061, 1810.0177, 1812.9647, 1812.9647, 1826.9792, 1835.8917, 1853.0256, 1868.0201, 1888.9293, 1936.9341, 1952.9316, 2198.1147, 2225.1714, 2246.1958, 2289.1677, 2485.2241, 3211.4470

8. PGK1_HORSE **Mass:** 44973 **Score:** 155 **Expect:** 2.1e-011 **Matches:** 9

Phosphoglycerate kinase 1 OS=Equus caballus GN=PGK1 PE=1 SV=2

Observed	Mr(expt)	Mr(calc)	ppm	Start	End	Miss	Ions	Peptide
1232.7129	1231.7056	1231.7540	-39.27	87	97	1	---	K.SLLGKDVLFLK.D
1367.7023	1366.6950	1366.7027	-5.63	172	184	0	---	R.AHSSMVGVNLPQK.A
1434.7605	1433.7532	1433.7523	0.63	19	30	1	---	R.VVMRVDFNVPMK.N
1634.7924	1633.7851	1633.7849	0.15	157	171	0	---	K.LGDVYVNDAFGTAHR.A
1634.7924	1633.7851	1633.7849	0.15	157	171	0	116	K.LGDVYVNDAFGTAHR.A
1747.8970	1746.8897	1746.8862	2.00	92	106	1	---	K.DVLFLKDCVGPEVEK.A
1759.8289	1758.8216	1758.8685	-26.63	231	246	0	---	K.VNEMIIGGGMAFTFLK.V + 2 Oxidation (M)
2048.0571	2047.0498	2046.9905	29.0	23	39	1	---	R.VDFNVPMKNNQITNNQR.I + Oxidation (M)
2514.2427	2513.2354	2513.2398	-1.74	383	406	1	---	K.WNTEDKVSHVSTGGGASLELLEGK.V

No match to: 855.0386, 856.0210, 858.2581, 860.0115, 863.5002, 873.0222, 875.2742, 881.2588, 885.4301, 917.2859, 951.2886, 987.4899, 988.4872, 1014.4448, 1037.5166, 1044.0621, 1118.5048, 1134.5503, 1136.5503, 1175.6055, 1175.6055, 1189.6184, 1248.5896, 1300.0398, 1320.6007, 1375.6530, 1376.6458, 1399.7317, 1416.7859, 1421.6720, 1431.6903, 1458.7081, 1473.8019, 1475.7791, 1482.8335, 1616.8318, 1691.8187, 1720.8691, 1739.7987, 1756.7877, 1757.8077, 1768.8522, 1796.0061, 1796.0061, 1810.0177, 1812.9647, 1812.9647, 1826.9792, 1835.8917, 1853.0256, 1868.0201, 1888.9293, 1936.9341, 1952.9316, 2198.1147, 2225.1714, 2246.1958, 2289.1677, 2485.2241, 3211.4470

9. PGK1_BOVIN **Mass:** 44908 **Score:** 154 **Expect:** 2.6e-011 **Matches:** 9

Phosphoglycerate kinase 1 OS=Bos taurus GN=PGK1 PE=2 SV=3

Observed	Mr(expt)	Mr(calc)	ppm	Start	End	Miss	Ions	Peptide
1232.7129	1231.7056	1231.7540	-39.27	87	97	1	---	K.SLLGKDVLFLK.D
1367.7023	1366.6950	1366.7391	-32.24	172	184	1	---	R.AHSSMVGVNLPKK.A
1434.7605	1433.7532	1433.7523	0.63	19	30	1	---	R.VVMRVDFNVPMK.N
1634.7924	1633.7851	1633.7849	0.15	157	171	0	---	K.LGDVYVNDAFGTAHR.A

1634.7924	1633.7851	1633.7849	0.15	157	171	0	116	K.LGDVYVNDAFGTAHR.A
1747.8970	1746.8897	1746.8862	2.00	92	106	1	---	K.DVLFLKDCVGPEVEK.A
1759.8289	1758.8216	1758.8685	-26.63	231	246	0	---	K.VNEMIIGGGMAFTFLK.V + 2 Oxidation (M)
2048.0571	2047.0498	2046.9905	29.0	23	39	1	---	R.VDFNVPMKNNQITNNQR.I + Oxidation (M)
2514.2427	2513.2354	2513.2398	-1.74	383	406	1	---	K.WNTEDKVSHVSTGGGASLELLEGK.V

No match to: 855.0386, 856.0210, 858.2581, 860.0115, 863.5002, 873.0222, 875.2742, 881.2588, 885.4301, 917.2859, 951.2886, 987.4899, 988.4872, 1014.4448, 1037.5166, 1044.0621, 1118.5048, 1134.5503, 1136.5503, 1175.6055, 1175.6055, 1189.6184, 1248.5896, 1300.0398, 1320.6007, 1375.6530, 1376.6458, 1399.7317, 1416.7859, 1421.6720, 1431.6903, 1458.7081, 1473.8019, 1475.7791, 1482.8335, 1616.8318, 1691.8187, 1720.8691, 1739.7987, 1756.7877, 1757.8077, 1768.8522, 1796.0061, 1796.0061, 1810.0177, 1812.9647, 1812.9647, 1826.9792, 1835.8917, 1853.0256, 1868.0201, 1888.9293, 1936.9341, 1952.9316, 2198.1147, 2225.1714, 2246.1958, 2289.1677, 2485.2241, 3211.4470

10. PGK2_MACFA **Mass:** 45235 **Score:** 152 **Expect:** 4.2e-011 **Matches:** 10

Phosphoglycerate kinase 2 OS=Macaca fascicularis GN=PGK2 PE=2 SV=1

Observed	Mr(expt)	Mr(calc)	ppm	Start	End	Miss	Ions	Peptide
885.4301	884.4228	884.4280	-5.81	193	199	0	---	K.ELDYFAK.A
987.4899	986.4826	986.4893	-6.80	32	39	0	---	K.NQITNNQR.I
1232.7129	1231.7056	1231.7540	-39.27	87	97	1	---	K.SLLGKDVLFLK.D
1376.6458	1375.6385	1375.7030	-46.90	172	184	0	---	R.AHSSMVGVNLPHK.A
1634.7924	1633.7851	1633.7849	0.15	157	171	0	---	K.LGDVYVNDAFGTAHR.A
1634.7924	1633.7851	1633.7849	0.15	157	171	0	116	K.LGDVYVNDAFGTAHR.A
1759.8289	1758.8216	1758.8685	-26.63	231	246	0	---	K.VNEMIIGGGMAYTFLK.V + Oxidation (M)
1936.9341	1935.9268	1935.9248	1.07	247	264	0	---	K.VLNNMEIGASLFDEEGAK.I
1952.9316	1951.9243	1951.9197	2.38	247	264	0	---	K.VLNNMEIGASLFDEEGAK.I + Oxidation (M)
2514.2427	2513.2354	2513.2398	-1.74	383	406	1	---	K.WNTEDKVSHVSTGGGASLELLEGK.I

No match to: 855.0386, 856.0210, 858.2581, 860.0115, 863.5002, 873.0222, 875.2742, 881.2588, 917.2859, 951.2886, 988.4872, 1014.4448, 1037.5166, 1044.0621, 1118.5048, 1134.5503, 1136.5503, 1175.6055, 1175.6055, 1189.6184, 1248.5896, 1300.0398, 1320.6007, 1367.7023, 1375.6530, 1399.7317, 1416.7859, 1421.6720, 1431.6903, 1434.7605, 1458.7081, 1473.8019, 1475.7791, 1482.8335, 1616.8318, 1691.8187, 1720.8691, 1739.7987, 1747.8970, 1756.7877, 1757.8077, 1768.8522, 1796.0061, 1796.0061, 1810.0177, 1812.9647, 1812.9647, 1826.9792, 1835.8917, 1853.0256, 1868.0201, 1888.9293, 2048.0571, 2198.1147, 2225.1714, 2246.1958, 2289.1677, 2485.2241, 3211.4470

11. PGK1_CRIGR **Mass:** 44933 **Score:** 148 **Expect:** 1.1e-010 **Matches:** 8

Phosphoglycerate kinase 1 OS=Cricetulus griseus GN=PGK1 PE=2 SV=2

Observed	Mr(expt)	Mr(calc)	ppm	Start	End	Miss	Ions	Peptide
1232.7129	1231.7056	1231.7540	-39.27	87	97	1	---	K.SLLGKDVLFLK.D
1367.7023	1366.6950	1366.7027	-5.63	172	184	0	---	R.AHSSMVGVNLPQK.A
1434.7605	1433.7532	1433.7523	0.63	19	30	1	---	R.VVMRVDFNVPMK.N
1634.7924	1633.7851	1633.7849	0.15	157	171	0	---	K.LGDVYVNDAFGTAHR.A
1634.7924	1633.7851	1633.7849	0.15	157	171	0	116	K.LGDVYVNDAFGTAHR.A
1759.8289	1758.8216	1758.8685	-26.63	231	246	0	---	K.VNEMIIGGGMAFTFLK.V + 2 Oxidation (M)
2048.0571	2047.0498	2046.9905	29.0	23	39	1	---	R.VDFNVPMKNNQITNNQR.I + Oxidation (M)
2514.2427	2513.2354	2513.2398	-1.74	383	406	1	---	K.WNTEDKVSHVSTGGGASLELLEGK.V

No match to: 855.0386, 856.0210, 858.2581, 860.0115, 863.5002, 873.0222, 875.2742, 881.2588, 885.4301, 917.2859, 951.2886, 987.4899, 988.4872, 1014.4448, 1037.5166, 1044.0621, 1118.5048, 1134.5503, 1136.5503, 1175.6055, 1175.6055, 1189.6184, 1248.5896, 1300.0398, 1320.6007, 1375.6530, 1376.6458, 1399.7317, 1416.7859, 1421.6720, 1431.6903, 1458.7081, 1473.8019, 1475.7791, 1482.8335, 1616.8318, 1691.8187, 1720.8691, 1739.7987, 1747.8970, 1756.7877, 1757.8077, 1768.8522, 1796.0061, 1796.0061, 1810.0177, 1812.9647, 1812.9647, 1826.9792, 1835.8917, 1853.0256, 1868.0201, 1888.9293, 1936.9341, 1952.9316, 2198.1147, 2225.1714, 2246.1958, 2289.1677, 2485.2241, 3211.4470

12. PGK1_MOUSE **Mass:** 44921 **Score:** 148 **Expect:** 1.1e-010 **Matches:** 8

Phosphoglycerate kinase 1 OS=Mus musculus GN=Pgk1 PE=1 SV=4

Observed	Mr(expt)	Mr(calc)	ppm	Start	End	Miss	Ions	Peptide
1232.7129	1231.7056	1231.7540	-39.27	87	97	1	---	K.SLLGKDVLFLK.D
1367.7023	1366.6950	1366.7027	-5.63	172	184	0	---	R.AHSSMVGVNLPQK.A
1434.7605	1433.7532	1433.7523	0.63	19	30	1	---	R.VVMRVDFNVPMK.N
1634.7924	1633.7851	1633.7849	0.15	157	171	0	---	K.LGDVYVNDAFGTAHR.A
1634.7924	1633.7851	1633.7849	0.15	157	171	0	116	K.LGDVYVNDAFGTAHR.A
1759.8289	1758.8216	1758.8685	-26.63	231	246	0	---	K.VNEMIIGGGMAFTFLK.V + 2 Oxidation (M)
2048.0571	2047.0498	2046.9905	29.0	23	39	1	---	R.VDFNVPMKNNQITNNQR.I + Oxidation (M)
2514.2427	2513.2354	2513.2398	-1.74	383	406	1	---	K.WNTEDKVSHVSTGGGASLELLEGK.V

No match to: 855.0386, 856.0210, 858.2581, 860.0115, 863.5002, 873.0222, 875.2742, 881.2588, 885.4301, 917.2859, 951.2886, 987.4899, 988.4872, 1014.4448, 1037.5166, 1044.0621, 1118.5048, 1134.5503, 1136.5503, 1175.6055, 1175.6055, 1189.6184, 1248.5896, 1300.0398, 1320.6007, 1375.6530, 1376.6458, 1399.7317, 1416.7859, 1421.6720, 1431.6903, 1458.7081, 1473.8019, 1475.7791, 1482.8335, 1616.8318, 1691.8187, 1720.8691, 1739.7987, 1747.8970, 1756.7877, 1757.8077, 1768.8522, 1796.0061, 1796.0061, 1810.0177, 1812.9647, 1812.9647, 1826.9792, 1835.8917, 1853.0256, 1868.0201, 1888.9293, 1936.9341, 1952.9316, 2198.1147, 2225.1714, 2246.1958, 2289.1677, 2485.2241, 3211.4470

13. PGK1_RAT **Mass:** 44909 **Score:** 148 **Expect:** 1.1e-010 **Matches:** 8

Phosphoglycerate kinase 1 OS=Rattus norvegicus GN=Pgk1 PE=1 SV=2

Observed	Mr(expt)	Mr(calc)	ppm	Start	End	Miss	Ions	Peptide
1232.7129	1231.7056	1231.7540	-39.27	87	97	1	---	K.SLLGKDVLFLK.D
1367.7023	1366.6950	1366.7027	-5.63	172	184	0	---	R.AHSSMVGVNLPQK.A
1434.7605	1433.7532	1433.7523	0.63	19	30	1	---	R.VVMRVDFNVPMK.N
1634.7924	1633.7851	1633.7849	0.15	157	171	0	---	K.LGDVYVNDAFGTAHR.A
1634.7924	1633.7851	1633.7849	0.15	157	171	0	116	K.LGDVYVNDAFGTAHR.A
1759.8289	1758.8216	1758.8685	-26.63	231	246	0	---	K.VNEMIIGGGMAFTFLK.V + 2 Oxidation (M)
2048.0571	2047.0498	2046.9905	29.0	23	39	1	---	R.VDFNVPMKNNQITNNQR.I + Oxidation (M)
2514.2427	2513.2354	2513.2398	-1.74	383	406	1	---	K.WNTEDKVSHVSTGGGASLELLEGK.V

No match to: 855.0386, 856.0210, 858.2581, 860.0115, 863.5002, 873.0222, 875.2742, 881.2588, 885.4301, 917.2859, 951.2886, 987.4899, 988.4872, 1014.4448, 1037.5166, 1044.0621, 1118.5048, 1134.5503, 1136.5503, 1175.6055, 1175.6055, 1189.6184, 1248.5896, 1300.0398, 1320.6007, 1375.6530, 1376.6458, 1399.7317, 1416.7859, 1421.6720, 1431.6903, 1458.7081, 1473.8019, 1475.7791, 1482.8335, 1616.8318, 1691.8187, 1720.8691, 1739.7987, 1747.8970, 1756.7877, 1757.8077, 1768.8522, 1796.0061, 1796.0061, 1810.0177, 1812.9647, 1812.9647, 1826.9792, 1835.8917, 1853.0256, 1868.0201, 1888.9293, 1936.9341, 1952.9316, 2198.1147, 2225.1714, 2246.1958, 2289.1677, 2485.2241, 3211.4470

14. PGK1_PIG **Mass:** 44929 **Score:** 148 **Expect:** 1.1e-010 **Matches:** 8

Phosphoglycerate kinase 1 OS=Sus scrofa GN=PGK1 PE=1 SV=3

Observed	Mr(expt)	Mr(calc)	ppm	Start	End	Miss	Ions	Peptide
1367.7023	1366.6950	1366.7391	-32.24	172	- 184	1	---	R.AHSSMVGVNLPKK.A
1434.7605	1433.7532	1433.7523	0.63	19	- 30	1	---	R.VVMRVDFNVPMK.N
1634.7924	1633.7851	1633.7849	0.15	157	- 171	0	---	K.LGDVYVNDAFGTAHR.A
1634.7924	1633.7851	1633.7849	0.15	157	- 171	0	116	K.LGDVYVNDAFGTAHR.A
1747.8970	1746.8897	1746.8862	2.00	92	- 106	1	---	K.DVLFLKDCVGPEVEK.A
1759.8289	1758.8216	1758.8685	-26.63	231	- 246	0	---	K.VNEMIIGGGMAFTFLK.V + 2 Oxidation (M)
2048.0571	2047.0498	2046.9905	29.0	23	- 39	1	---	R.VDFNVPMKNNQITNNQR.I + Oxidation (M)
2514.2427	2513.2354	2513.2398	-1.74	383	- 406	1	---	K.WNTEDKVSHVSTGGGASLELLEGK.V

No match to: 855.0386, 856.0210, 858.2581, 860.0115, 863.5002, 873.0222, 875.2742, 881.2588, 885.4301, 917.2859, 951.2886, 987.4899, 988.4872, 1014.4448, 1037.5166, 1044.0621, 1118.5048, 1134.5503, 1136.5503, 1175.6055, 1175.6055, 1189.6184, 1232.7129, 1248.5896, 1300.0398, 1320.6007, 1375.6530, 1376.6458, 1399.7317, 1416.7859, 1421.6720, 1431.6903, 1458.7081, 1473.8019, 1475.7791, 1482.8335, 1616.8318, 1691.8187, 1720.8691, 1739.7987, 1756.7877, 1757.8077, 1768.8522, 1796.0061, 1796.0061, 1810.0177, 1812.9647, 1812.9647, 1826.9792, 1835.8917, 1853.0256, 1868.0201, 1888.9293, 1936.9341, 1952.9316, 2198.1147, 2225.1714, 2246.1958, 2289.1677, 2485.2241, 3211.4470

15. PGK1_MACEU **Mass:** 45405 **Score:** 133 **Expect:** 3.3e-009 **Matches:** 5

Phosphoglycerate kinase 1 OS=Macropus eugenii GN=PGK1 PE=2 SV=2

Observed	Mr(expt)	Mr(calc)	ppm	Start	End	Miss	Ions	Peptide
1232.7129	1231.7056	1231.7540	-39.27	87	- 97	1	---	K.SLLGKDVLFLK.D
1367.7023	1366.6950	1366.7027	-5.63	172	- 184	0	---	R.AHSSMVGVNLPQK.A
1634.7924	1633.7851	1633.7849	0.15	157	- 171	0	---	K.LGDVYVNDAFGTAHR.A
1634.7924	1633.7851	1633.7849	0.15	157	- 171	0	116	K.LGDVYVNDAFGTAHR.A
2514.2427	2513.2354	2513.2398	-1.74	383	- 406	1	---	K.WNTEDKVSHVSTGGGASLELLEGK.V

No match to: 855.0386, 856.0210, 858.2581, 860.0115, 863.5002, 873.0222, 875.2742, 881.2588, 885.4301, 917.2859, 951.2886, 987.4899, 988.4872, 1014.4448, 1037.5166, 1044.0621, 1118.5048, 1134.5503, 1136.5503, 1175.6055, 1175.6055, 1189.6184, 1248.5896, 1300.0398, 1320.6007, 1375.6530, 1376.6458, 1399.7317, 1416.7859, 1421.6720, 1431.6903, 1434.7605, 1458.7081, 1473.8019, 1475.7791, 1482.8335, 1616.8318, 1691.8187, 1720.8691, 1739.7987, 1747.8970, 1756.7877, 1757.8077, 1759.8289, 1768.8522, 1796.0061, 1796.0061, 1810.0177, 1812.9647, 1812.9647, 1826.9792, 1835.8917, 1853.0256, 1868.0201, 1888.9293, 1936.9341, 1952.9316, 2048.0571, 2198.1147, 2225.1714, 2246.1958, 2289.1677, 2485.2241, 3211.4470

16. PGK2_MOUSE **Mass:** 45223 **Score:** 127 **Expect:** 1.3e-008 **Matches:** 4

Phosphoglycerate kinase 2 OS=Mus musculus GN=Pgk2 PE=1 SV=4

Observed	Mr(expt)	Mr(calc)	ppm	Start	End	Miss	Ions	Peptide
1634.7924	1633.7851	1633.7849	0.15	157	- 171	0	---	K.LGDVYVNDAFGTAHR.A
1634.7924	1633.7851	1633.7849	0.15	157	- 171	0	116	K.LGDVYVNDAFGTAHR.A
1810.0177	1809.0104	1809.0512	-22.54	200	- 216	0	---	K.ALEKPERPFLAILGGAK.V
2048.0571	2047.0498	2046.9905	29.0	23	- 39	1	---	R.VDFNVPMKNNQITNNQR.I + Oxidation (M)

No match to: 855.0386, 856.0210, 858.2581, 860.0115, 863.5002, 873.0222, 875.2742, 881.2588, 885.4301, 917.2859, 951.2886, 987.4899, 988.4872, 1014.4448, 1037.5166, 1044.0621, 1118.5048, 1134.5503, 1136.5503, 1175.6055, 1175.6055, 1189.6184, 1232.7129, 1248.5896, 1300.0398, 1320.6007, 1367.7023, 1375.6530, 1376.6458, 1399.7317, 1416.7859, 1421.6720, 1431.6903, 1434.7605, 1458.7081, 1473.8019, 1475.7791, 1482.8335, 1616.8318, 1691.8187, 1720.8691, 1739.7987, 1747.8970, 1756.7877, 1757.8077, 1759.8289, 1768.8522, 1796.0061, 1796.0061, 1812.9647, 1812.9647, 1826.9792, 1835.8917, 1853.0256, 1868.0201, 1888.9293, 1936.9341, 1952.9316, 2198.1147, 2225.1714, 2246.1958, 2289.1677, 2485.2241, 2514.2427, 3211.4470

17. NEBU_HUMAN **Mass:** 775393 **Score:** 79 **Expect:** 0.00082 **Matches:** 43

Nebulin OS=Homo sapiens GN=NEB PE=1 SV=5

Observed	Mr(expt)	Mr(calc)	ppm	Start	End	Miss	Ions	Peptide
885.4301	884.4228	884.3949	31.5	2517	- 2523	0	---	R.MGYEELK.R + Oxidation (M)
988.4872	987.4799	987.5349	-55.65	3653	- 3661	1	---	R.AGEILSDRK.Y
1014.4448	1013.4375	1013.4276	9.75	950	- 957	0	---	K.ADYNSWMK.G
1037.5166	1036.5093	1036.4607	46.9	302	- 310	0	---	R.MNADNISTR.K + Oxidation (M)
1118.5048	1117.4975	1117.5412	-39.12	3546	- 3554	0	---	K.MMWSIHVAK.I + Oxidation (M)
1134.5503	1133.5430	1133.5362	6.05	3546	- 3554	0	---	K.MMWSIHVAK.I + 2 Oxidation (M)
1136.5503	1135.5430	1135.4977	39.9	3060	- 3068	0	---	K.MMWSMHVAK.I + Oxidation (M)
1175.6055	1174.5982	1174.6571	-50.09	4730	- 4739	1	---	K.NQKHLASHIK.Y
1175.6055	1174.5982	1174.6571	-50.09	4730	- 4739	1	---	K.NQKHLASHIK.Y
1189.6184	1188.6111	1188.5927	15.5	2235	- 2243	0	---	K.HLYTIDWNK.D
1367.7023	1366.6950	1366.6914	2.62	2018	- 2029	1	---	K.ANAINMSDKLYK.L
1376.6458	1375.6385	1375.6442	-4.12	6347	- 6358	0	---	K.ATPTPFTPEMER.V
1399.7317	1398.7244	1398.6779	33.3	798	- 809	0	---	R.VNAYNLSDNVYK.Q
1416.7859	1415.7786	1415.7408	26.7	4485	- 4496	1	---	K.HADLVNSELKYK.E
1421.6720	1420.6647	1420.6946	-21.06	1426	- 1437	0	---	R.NVNQIQSDNVYK.D
1431.6903	1430.6830	1430.6534	20.7	4380	- 4390	1	---	K.NQEMMSQIKYK.K + 2 Oxidation (M)
1434.7605	1433.7532	1433.7184	24.3	5819	- 5831	1	---	K.VMKDANNLASEVK.Y + Oxidation (M)
1458.7081	1457.7008	1457.7548	-37.02	853	- 866	1	---	K.GKMIGALSINDDPK.M
1473.8019	1472.7946	1472.7698	16.9	3150	- 3163	0	---	R.GIGWVPIGSMDVVK.C + Oxidation (M)
1616.8318	1615.8245	1615.7631	38.0	4435	- 4448	0	---	K.YTFSPDTPHISHSK.D
1634.7924	1633.7851	1633.8788	-57.31	5032	- 5045	1	---	K.IHTTPDTPEIRQVK.K
1634.7924	1633.7851	1633.8788	-57.31	5032	- 5045	1	---	K.IHTTPDTPEIRQVK.K

1691.8187	1690.8114	1690.8275	-9.48	110	124	1	---	K.GQPYASTTDTPELRR.I
1720.8691	1719.8618	1719.8978	-20.92	5626	5641	0	---	R.GTCHAVPDTPQILLAK.T
1747.8970	1746.8897	1746.8975	-4.43	2907	2922	1	---	R.GIGWVSIGSLDVEKCK.R
1756.7877	1755.7804	1755.8580	-44.18	216	229	1	---	K.SLFYPYNDSPELRR.V
1757.8077	1756.8004	1756.9393	-79.04	2324	2339	1	---	R.SLQDDPKLVLSMNVAK.M
1759.8289	1758.8216	1758.8649	-24.60	3773	3788	1	---	K.QKGHHIGAQSIEDDPK.I
1812.9647	1811.9574	1811.9669	-5.24	2940	2955	1	---	R.FKFTSVTDSLEQVLAK.N
1812.9647	1811.9574	1811.9087	26.9	2080	2095	1	5	R.SLEDDPKLVHSMQVAK.M + Oxidation (M)
1826.9792	1825.9719	1825.9422	16.3	6218	6234	0	---	K.ENLGTGIPTTVTPEIER.V
1835.8917	1834.8844	1834.8818	1.43	4542	4557	0	---	K.ANVHIPNDMMNHVLAK.R + 2 Oxidation (M)
1868.0201	1867.0128	1866.9332	42.7	3218	3233	0	---	K.TQVHIMPDTPEIMLAR.Q + Oxidation (M)
1888.9293	1887.9220	1887.9036	9.75	1042	1057	1	---	K.VNAYNISENMYKADLK.D + Oxidation (M)
1936.9341	1935.9268	1935.8608	34.1	5100	5115	1	---	K.GFGCFLYDTPDMVRSR.H + Oxidation (M)
1952.9316	1951.9243	1952.0255	-51.83	4888	4904	1	---	R.NDYKLVTDTPVYVQAVK.S
2048.0571	2047.0498	2046.9469	50.3	4435	4452	1	---	K.YTFSPDTPHISHSKDMGK.L
2198.1147	2197.1074	2196.9707	62.2	321	337	1	---	K.DQIYFMQTETPEYKMNK.K + 2 Oxidation (M)
2246.1958	2245.1885	2245.1702	8.14	993	1012	1	---	K.FTSIEDAPITVQSKINQAQR.S
2289.1677	2288.1604	2288.1180	18.5	5700	5719	1	---	K.SNYSIMLEPPEVKHAMEVAK.K + Oxidation (M)
2485.2241	2484.2168	2484.2002	6.70	3775	3797	1	---	K.GHHIGAQSIEDDPKIMCAIHAGK.I
2514.2427	2513.2354	2513.2802	-17.81	245	267	1	---	K.GLAEQQAQFTPLADPPDIEFAKK.V
3211.4470	3210.4397	3210.6117	-53.56	6583	6613	1	---	K.TTELPQQRSSSVATQQTTVSSIPSHPSTAGK.I

No match to: 855.0386, 856.0210, 858.2581, 860.0115, 863.5002, 873.0222, 875.2742, 881.2588, 917.2859, 951.2886, 987.4899, 1044.0621, 1232.7129, 1248.5896, 1300.0398, 1320.6007, 1375.6530, 1475.7791, 1482.8335, 1739.7987, 1768.8522, 1796.0061, 1796.0061, 1810.0177, 1853.0256, 2225.1714

18. CC171_HUMAN **Mass:** 154083 **Score:** 50 **Expect:** 0.73 **Matches:** 17
Coiled-coil domain-containing protein 171 OS=Homo sapiens GN=CCDC171 PE=2 SV=1

Observed	Mr(expt)	Mr(calc)	ppm	Start	End	Miss	Ions	Peptide
863.5002	862.4929	862.4331	69.4	1002	1008	1	---	K.RSVNEMK.K
987.4899	986.4826	986.5396	-57.78	495	502	1	---	K.NIKELQDK.L
1037.5166	1036.5093	1036.4495	57.7	478	486	0	---	K.ACNELDSTK.Q
1320.6007	1319.5934	1319.6470	-40.57	1159	1170	0	---	R.DQISLSWSAASR.N
1375.6530	1374.6457	1374.6636	-12.97	1192	1204	0	---	K.GGPEVVACQAMIK.S + Oxidation (M)
1376.6458	1375.6385	1375.7129	-54.08	517	528	1	---	K.CADREALISTLK.V
1473.8019	1472.7946	1472.8212	-18.03	937	949	1	---	K.DSLVQRLAHGLHK.V
1475.7791	1474.7718	1474.7272	30.3	1217	1228	1	---	R.IMTLEKEMTSHR.S
1616.8318	1615.8245	1615.8570	-20.08	444	457	1	---	K.DKDKPPSFSVVLER.L
1634.7924	1633.7851	1633.7076	47.5	241	253	0	---	K.LETEHMDCSDLLR.R + Oxidation (M)
1634.7924	1633.7851	1633.7076	47.5	241	253	0	---	K.LETEHMDCSDLLR.R + Oxidation (M)
1739.7987	1738.7914	1738.8122	-11.94	255	268	1	---	R.QTSELEFSTQREER.L
1757.8077	1756.8004	1756.8672	-38.01	753	766	0	---	R.DFLQEQVNTFELFK.L
1759.8289	1758.8216	1758.8934	-40.80	112	126	1	---	R.IQEKLCAQNSELQAK.T
1868.0201	1867.0128	1867.0064	3.44	975	990	1	---	K.QILGFTQRLHAAEVER.R
2198.1147	2197.1074	2197.0862	9.65	1070	1089	1	---	K.LELHSSEEADKNQTLGEAVK.S
2246.1958	2245.1885	2245.1590	13.2	50	69	1	---	K.LEITTKHNAELASYESQIAK.L

No match to: 855.0386, 856.0210, 858.2581, 860.0115, 873.0222, 875.2742, 881.2588, 885.4301, 917.2859, 951.2886, 988.4872, 1014.4448, 1044.0621, 1118.5048, 1134.5503, 1136.5503, 1175.6055, 1175.6055, 1189.6184, 1232.7129, 1248.5896, 1300.0398, 1367.7023, 1399.7317, 1416.7859, 1421.6720, 1431.6903, 1434.7605, 1458.7081, 1482.8335, 1691.8187, 1720.8691, 1747.8970, 1756.7877, 1768.8522, 1796.0061, 1796.0061, 1810.0177, 1812.9647, 1812.9647, 1826.9792, 1835.8917, 1853.0256, 1888.9293, 1936.9341, 1952.9316, 2048.0571, 2225.1714, 2289.1677, 2485.2241, 2514.2427, 3211.4470

19. CE034_BOVIN **Mass:** 73735 **Score:** 40 **Expect:** 6.5 **Matches:** 10
Uncharacterized protein C5orf34 homolog OS=Bos taurus PE=2 SV=1

Observed	Mr(expt)	Mr(calc)	ppm	Start	End	Miss	Ions	Peptide
1320.6007	1319.5934	1319.6180	-18.59	195	204	0	---	K.ENELYHQIMK.S + Oxidation (M)
1367.7023	1366.6950	1366.6398	40.4	279	290	1	---	R.MLTSDVSEERGK.E + Oxidation (M)
1376.6458	1375.6385	1375.7194	-58.82	602	613	1	---	K.SSETLLKEVNEK.S
1431.6903	1430.6830	1430.7453	-43.50	496	507	1	---	R.QVNRGLTLGWCK.L
1747.8970	1746.8897	1746.9165	-15.35	57	70	1	---	R.THFVISAYREQLQR.A
1757.8077	1756.8004	1756.7944	3.43	321	334	0	---	K.QFGEEEYSYHELVK.V
1796.0061	1794.9988	1794.9853	7.55	39	54	1	---	K.APPVSAHPLQQPERIR.Q
1796.0061	1794.9988	1794.9853	7.55	39	54	1	---	K.APPVSAHPLQQPERIR.Q
1888.9293	1887.9220	1887.9538	-16.80	151	167	1	---	K.VSQKPDSSIEVSEKNNK.G
2289.1677	2288.1604	2288.1259	15.1	345	364	1	---	R.LTHKHMNSIEIYPGDGSVFK.S + Oxidation (M)

No match to: 855.0386, 856.0210, 858.2581, 860.0115, 863.5002, 873.0222, 875.2742, 881.2588, 885.4301, 917.2859, 951.2886, 987.4899, 988.4872, 1014.4448, 1037.5166, 1044.0621, 1118.5048, 1134.5503, 1136.5503, 1175.6055, 1175.6055, 1189.6184, 1232.7129, 1248.5896, 1300.0398, 1375.6530, 1399.7317, 1416.7859, 1421.6720, 1434.7605, 1458.7081, 1473.8019, 1475.7791, 1482.8335, 1616.8318, 1634.7924, 1634.7924, 1691.8187, 1720.8691, 1739.7987, 1756.7877, 1759.8289, 1768.8522, 1810.0177, 1812.9647, 1812.9647, 1826.9792, 1835.8917, 1853.0256, 1868.0201, 1936.9341, 1952.9316, 2048.0571, 2198.1147, 2225.1714, 2246.1958, 2485.2241, 2514.2427, 3211.4470

20. LDHA_PANTR **Mass:** 36924 **Score:** 39 **Expect:** 9 **Matches:** 7
L-lactate dehydrogenase A chain OS=Pan troglodytes GN=LDHA PE=2 SV=3

Observed	Mr(expt)	Mr(calc)	ppm	Start	End	Miss	Ions	Peptide
1118.5048	1117.4975	1117.5768	-70.91	319	328	0	---	K.SADTLWGIQK.E
1134.5503	1133.5430	1133.5564	-11.81	306	315	0	---	K.VTLTSEEEAR.L
1248.5896	1247.5823	1247.5928	-8.42	158	169	0	---	R.VIGSGCNLDSAR.F

1375.6530	1374.6457	1374.7354	-65.25	306 - 317	1	---	K.VTLTSEEEARLK.K
1757.8077	1756.8004	1756.9359	-77.13	229 - 243	1	---	K.EVHKQVVESAYEVIK.L
2048.0571	2047.0498	2047.0698	-9.75	6 - 22	1	---	K.DQLIHNLLKEEQTPQNK.I
2289.1677	2288.1604	2288.1723	-5.17	285 - 305	0	---	K.DDVFLSVPCILGQNGISDLVK.V

No match to: 855.0386, 856.0210, 858.2581, 860.0115, 863.5002, 873.0222, 875.2742, 881.2588, 885.4301, 917.2859, 951.2886, 987.4899, 988.4872, 1014.4448, 1037.5166, 1044.0621, 1136.5503, 1175.6055, 1175.6055, 1189.6184, 1232.7129, 1300.0398, 1320.6007, 1367.7023, 1376.6458, 1399.7317, 1416.7859, 1421.6720, 1431.6903, 1434.7605, 1458.7081, 1473.8019, 1475.7791, 1482.8335, 1616.8318, 1634.7924, 1634.7924, 1691.8187, 1720.8691, 1739.7987, 1747.8970, 1756.7877, 1759.8289, 1768.8522, 1796.0061, 1796.0061, 1810.0177, 1812.9647, 1812.9647, 1826.9792, 1835.8917, 1853.0256, 1868.0201, 1888.9293, 1936.9341, 1952.9316, 2198.1147, 2225.1714, 2246.1958, 2485.2241, 2514.2427, 3211.4470

Search Parameters

```
Type of search         : Sequence Query
Enzyme                 : Trypsin
Fixed modifications    : Carbamidomethyl (C)
Variable modifications : Oxidation (M)
Mass values            : Monoisotopic
Protein Mass           : Unrestricted
Peptide Mass Tolerance : ± 80 ppm
Fragment Mass Tolerance: ± 0.3 Da
Max Missed Cleavages   : 1
Instrument type        : MALDI-TOF-TOF
Query1 (855.0386,1+)   : <no title>
Query2 (856.0210,1+)   : <no title>
Query3 (858.2581,1+)   : <no title>
Query4 (860.0115,1+)   : <no title>
Query5 (863.5002,1+)   : <no title>
Query6 (873.0222,1+)   : <no title>
Query7 (875.2742,1+)   : <no title>
Query8 (881.2588,1+)   : <no title>
Query9 (885.4301,1+)   : <no title>
Query10 (917.2859,1+)  : <no title>
Query11 (951.2886,1+)  : <no title>
Query12 (987.4899,1+)  : <no title>
Query13 (988.4872,1+)  : <no title>
Query14 (1014.4448,1+) : <no title>
Query15 (1037.5166,1+) : <no title>
Query16 (1044.0621,1+) : <no title>
Query17 (1118.5048,1+) : <no title>
Query18 (1134.5503,1+) : <no title>
Query19 (1136.5503,1+) : <no title>
Query20 (1175.6055,1+) : <no title>
Query21 (1175.6055,1+) : MaldiWellID: 55967, SpectrumID: 109859,
Query22 (1189.6184,1+) : <no title>
Query23 (1232.7129,1+) : <no title>
Query24 (1248.5896,1+) : <no title>
Query25 (1300.0398,1+) : <no title>
Query26 (1320.6007,1+) : <no title>
Query27 (1367.7023,1+) : <no title>
Query28 (1375.6530,1+) : <no title>
Query29 (1376.6458,1+) : <no title>
Query30 (1399.7317,1+) : <no title>
Query31 (1416.7859,1+) : <no title>
Query32 (1421.6720,1+) : <no title>
Query33 (1431.6903,1+) : <no title>
Query34 (1434.7605,1+) : <no title>
Query35 (1458.7081,1+) : <no title>
Query36 (1473.8019,1+) : <no title>
Query37 (1475.7791,1+) : <no title>
Query38 (1482.8335,1+) : <no title>
Query39 (1616.8318,1+) : <no title>
Query40 (1634.7924,1+) : <no title>
Query41 (1634.7924,1+) : MaldiWellID: 55967, SpectrumID: 109856,
Query42 (1691.8187,1+) : <no title>
Query43 (1720.8691,1+) : <no title>
Query44 (1739.7987,1+) : <no title>
Query45 (1747.8970,1+) : <no title>
Query46 (1756.7877,1+) : <no title>
Query47 (1757.8077,1+) : <no title>
Query48 (1759.8289,1+) : <no title>
Query49 (1768.8522,1+) : <no title>
Query50 (1796.0061,1+) : <no title>
Query51 (1796.0061,1+) : MaldiWellID: 55967, SpectrumID: 109857,
Query52 (1810.0177,1+) : <no title>
Query53 (1812.9647,1+) : <no title>
Query54 (1812.9647,1+) : MaldiWellID: 55967, SpectrumID: 109858,
Query55 (1826.9792,1+) : <no title>
Query56 (1835.8917,1+) : <no title>
Query57 (1853.0256,1+) : <no title>
Query58 (1868.0201,1+) : <no title>
Query59 (1888.9293,1+) : <no title>
Query60 (1936.9341,1+) : <no title>
Query61 (1952.9316,1+) : <no title>
Query62 (2048.0571,1+) : <no title>
Query63 (2198.1147,1+) : <no title>
Query64 (2225.1714,1+) : <no title>
```

```
Query65 (2246.1958,1+) : <no title>
Query66 (2289.1677,1+) : <no title>
Query67 (2485.2241,1+) : <no title>
Query68 (2514.2427,1+) : <no title>
Query69 (3211.4470,1+) : <no title>
```
