# Supplementary material for: Vasectomy and Photoperiodic Regimen Modify the Protein Profile, Hormonal Content and Antioxidant Enzymes Activity of Ram Seminal Plasma
Source: Int J Mol Sci. 2020 Oct 29;21(21):8063. doi: 10.3390/ijms21218063 (PMC7663742; doi:10.3390/ijms21218063)
Supplement: Supplementary file 1 [file ijms-21-08063-s001.zip › Supplementary file 2_Mascot protein identification/Sorbitol dehydrogenase identification.pdf]

# Mascot Search Results

## Protein View

Match to: **DHSO_BOVIN** Score: **133** Expect: **3.3e-009**
**Sorbitol dehydrogenase OS=Bos taurus GN=SORD PE=2 SV=3**

Nominal mass ($M_r$): **38645**; Calculated pI value: **7.14**
NCBI BLAST search of DHSO_BOVIN against nr
Unformatted sequence string for pasting into other applications

Taxonomy: Bos taurus

Fixed modifications: Carbamidomethyl (C)
Variable modifications: Oxidation (M)
Cleavage by Trypsin: cuts C-term side of KR unless next residue is P
Number of mass values searched: **65**
Number of mass values matched: **16**
Sequence Coverage: **54%**

Matched peptides shown in **Bold Red**

**1** MAAAKPENLS LVVHGPGDLR **LENYPIPEPG PNEVLLKMHS VGICGSDVHY**
**51** **WQHGRIGDFV VKKPMVLGHE ASGTVVK**VGS LVR**HLQPGDR VAIEPGAPR**E
**101** TDEFCKIGR**Y NLSPTIFFCA TPPDDGNLCR** FYK**HNANFCY KLPDNVTFEE**
**151** **GALIEPLSVG IHACR**RAGVT LGNKVLVCGA GPIGLVSLLA AK**AMGAAQVV**
**201** **VTDLSASR**LS K**AKEVGADFI LQISNESPQE IAK**KVEGLLG SKPEVTIECT
**251** GVETSIQAGI YATHSGGTLV LVGLGSEMTS VPLVHAATRE VDIKGVFR**YC**
**301** **NTWPMAISML ASKSVNVKPL VTHRFPLEK**A LEAFETSKKG LGLKVMIKCD
**351** PNDQNP

Show predicted peptides also

Sort Peptides By ◉ Residue Number ○ Increasing Mass ○ Decreasing Mass

| Start - End | Observed | Mr(expt) | Mr(calc) | ppm | Miss | Sequence |
|---|---|---|---|---|---|---|
| 21 - 37 | 1922.0380 | 1921.0307 | 1921.0196 | 6 | 0 | R.LENYPIPEPGPNEVLLK.M |
| 38 - 55 | 2125.9543 | 2124.9470 | 2124.9371 | 5 | 0 | K.MHSVGICGSDVHYWQHGR.I |
| 38 - 55 | 2141.9475 | 2140.9402 | 2140.9320 | 4 | 0 | K.MHSVGICGSDVHYWQHGR.I Oxidation (M) |
| 56 - 77 | 2311.3108 | 2310.3035 | 2310.2770 | 11 | 1 | R.IGDFVVKKPMVLGHEASGTVVK.V |
| 63 - 77 | 1552.8657 | 1551.8584 | 1551.8443 | 9 | 0 | K.KPMVLGHEASGTVVK.V |
| 63 - 77 | 1568.8557 | 1567.8484 | 1567.8392 | 6 | 0 | K.KPMVLGHEASGTVVK.V Oxidation (M) |
| 84 - 99 | 1712.9301 | 1711.9228 | 1711.9118 | 6 | 1 | R.HLQPGDRVAIEPGAPR.E |
| 110 - 130 | 2458.1199 | 2457.1126 | 2457.1093 | 1 | 0 | R.YNLSPTIFFCATPPDDGNLCR.F |
| 134 - 141 | 1053.4615 | 1052.4542 | 1052.4498 | 4 | 0 | K.HNANFCYK.L |
| 142 - 165 | 2637.3391 | 2636.3318 | 2636.3268 | 2 | 0 | K.LPDNVTFEEGALIEPLSVGIHACR.R |
| 193 - 208 | 1575.8231 | 1574.8158 | 1574.8086 | 5 | 0 | K.AMGAAQVVVTDLSASR.L |
| 212 - 233 | 2387.2449 | 2386.2376 | 2386.2380 | -0 | 1 | K.AKEVGADFILQISNESPQEIAK.K |
| 214 - 233 | 2188.1106 | 2187.1033 | 2187.1059 | -1 | 0 | K.EVGADFILQISNESPQEIAK.K |
| 299 - 313 | 1772.8123 | 1771.8050 | 1771.8095 | -3 | 0 | R.YCNTWPMAISMLASK.S |
| 314 - 324 | 1249.7527 | 1248.7454 | 1248.7303 | 12 | 0 | K.SVNVKPLVTHR.F |
| 314 - 329 | 1864.0842 | 1863.0769 | 1863.0730 | 2 | 1 | K.SVNVKPLVTHRFPLEK.A |

**No match to:** 853.2671, 855.0451, 856.0256, 861.0641, 870.0215, 871.0246, 873.0248, 877.0393, 877.2717, 879.0370

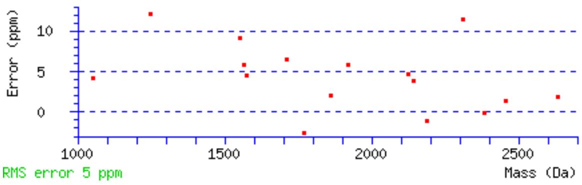


**Mascot:** http://www.matrixscience.com/

# Mascot Search Results

```
User          :
Email         :
Search title  : SampleSetID: 610, AnalysisID: 4511, MaldiWellID: 55565, SpectrumID: 109828, Path=\160212\MS\16-13 Jose Alvaro
Database      : SwissProt sprot_160208 (550116 sequences; 196219159 residues)
Taxonomy      : Mammalia (mammals) (66429 sequences)
Timestamp     : 12 Feb 2016 at 10:22:08 GMT
Top Score     : 133 for DHSO_BOVIN, Sorbitol dehydrogenase OS=Bos taurus GN=SORD PE=2 SV=3
```

## Mascot Score Histogram

Protein score is -10*Log(P), where P is the probability that the observed match is a random event.
Protein scores greater than 61 are significant (p<0.05).

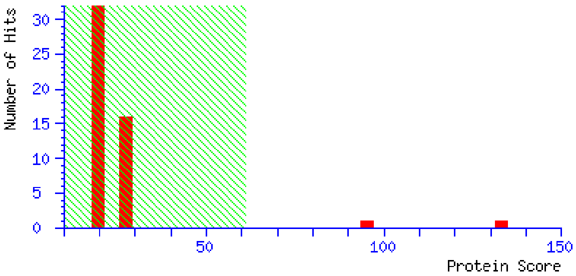


## Protein Summary Report

Format As | Protein Summary | Help

Significance threshold p< 0.05 | Max. number of hits 20

Re-Search All | Search Unmatched

## Index

| | Accession | Mass | Score | Description |
|---|---|---|---|---|
| 1. | DHSO_BOVIN | 38645 | 133 | Sorbitol dehydrogenase OS=Bos taurus GN=SORD PE=2 SV=3 |
| 2. | DHSO_SHEEP | 38377 | 93 | Sorbitol dehydrogenase OS=Ovis aries GN=SORD PE=1 SV=1 |
| 3. | TSNAX_MACFA | 33163 | 28 | Translin-associated protein X OS=Macaca fascicularis GN=TSNAX PE=2 SV=1 |
| 4. | F8I2_HUMAN | 39478 | 27 | Factor VIII intron 22 protein OS=Homo sapiens GN=F8A1 PE=1 SV=2 |
| 5. | PNMT_HUMAN | 31178 | 27 | Phenylethanolamine N-methyltransferase OS=Homo sapiens GN=PNMT PE=1 SV=1 |
| 6. | OVGP1_BOVIN | 59922 | 26 | Oviduct-specific glycoprotein (Fragment) OS=Bos taurus GN=OVGP1 PE=1 SV=1 |
| 7. | PKHA7_HUMAN | 127626 | 26 | Pleckstrin homology domain-containing family A member 7 OS=Homo sapiens GN=PLEKHA7 PE=1 SV=2 |
| 8. | F107B_HUMAN | 15548 | 25 | Protein FAM107B OS=Homo sapiens GN=FAM107B PE=1 SV=1 |
| 9. | F107B_MOUSE | 15562 | 25 | Protein FAM107B OS=Mus musculus GN=Fam107b PE=1 SV=2 |
| 10. | F107B_RAT | 15576 | 25 | Protein FAM107B OS=Rattus norvegicus GN=Fam107b PE=2 SV=1 |
| 11. | KR204_HUMAN | 4791 | 25 | Putative keratin-associated protein 20-4 OS=Homo sapiens GN=KRTAP20-4 PE=3 SV=1 |
| 12. | PATE2_HUMAN | 13633 | 25 | Prostate and testis expressed protein 2 OS=Homo sapiens GN=PATE2 PE=2 SV=1 |
| 13. | MAGF1_HUMAN | 35200 | 24 | Melanoma-associated antigen F1 OS=Homo sapiens GN=MAGEF1 PE=1 SV=2 |
| 14. | TLR2_GORGO | 90906 | 24 | Toll-like receptor 2 OS=Gorilla gorilla gorilla GN=TLR2 PE=2 SV=1 |
| 15. | TLR2_HUMAN | 90920 | 24 | Toll-like receptor 2 OS=Homo sapiens GN=TLR2 PE=1 SV=1 |
| 16. | TLR2_MACMU | 91154 | 24 | Toll-like receptor 2 OS=Macaca mulatta GN=TLR2 PE=2 SV=1 |
| 17. | GT2D2_BOVIN | 108145 | 24 | General transcription factor II-I repeat domain-containing protein 2 OS=Bos taurus GN=GTF2IRD2 PE=2 SV=1 |
| 18. | KPYM_PONAB | 58494 | 24 | Pyruvate kinase PKM OS=Pongo abelii GN=PKM PE=2 SV=3 |
| 19. | WDR76_PAPAN | 70432 | 23 | WD repeat-containing protein 76 OS=Papio anubis GN=WDR76 PE=3 SV=1 |
| 20. | CRKL_MOUSE | 33923 | 23 | Crk-like protein OS=Mus musculus GN=Crkl PE=1 SV=2 |

## Results List

**1.** DHSO_BOVIN **Mass:** 38645 **Score:** 133 **Expect:** 3.3e-009 **Matches:** 16

Sorbitol dehydrogenase OS=Bos taurus GN=SORD PE=2 SV=3

| Observed | Mr(expt) | Mr(calc) | ppm | Start | | End | Miss | Peptide |
|---|---|---|---|---|---|---|---|---|
| 1053.4615 | 1052.4542 | 1052.4498 | 4.22 | 134 | - | 141 | 0 | K.HNANFCYK.L |
| 1249.7527 | 1248.7454 | 1248.7303 | 12.1 | 314 | - | 324 | 0 | K.SVNVKPLVTHR.F |
| 1552.8657 | 1551.8584 | 1551.8443 | 9.10 | 63 | - | 77 | 0 | K.KPMVLGHEASGTVVK.V |
| 1568.8557 | 1567.8484 | 1567.8392 | 5.87 | 63 | - | 77 | 0 | K.KPMVLGHEASGTVVK.V + Oxidation (M) |
| 1575.8231 | 1574.8158 | 1574.8086 | 4.56 | 193 | - | 208 | 0 | K.AMGAAQVVVTDLSASR.L |
| 1712.9301 | 1711.9228 | 1711.9118 | 6.45 | 84 | - | 99 | 1 | R.HLQPGDRVAIEPGAPR.E |
| 1772.8123 | 1771.8050 | 1771.8095 | -2.55 | 299 | - | 313 | 0 | R.YCNTWPMAISMLASK.S |
| 1864.0842 | 1863.0769 | 1863.0730 | 2.08 | 314 | - | 329 | 1 | K.SVNVKPLVTHRFPLEK.A |
| 1922.0380 | 1921.0307 | 1921.0196 | 5.77 | 21 | - | 37 | 0 | R.LENYPIPEPGPNEVLLK.M |
| 2125.9543 | 2124.9470 | 2124.9371 | 4.68 | 38 | - | 55 | 0 | K.MHSVGICGSDVHYWQHGR.I |
| 2141.9475 | 2140.9402 | 2140.9320 | 3.85 | 38 | - | 55 | 0 | K.MHSVGICGSDVHYWQHGR.I + Oxidation (M) |
| 2188.1106 | 2187.1033 | 2187.1059 | -1.18 | 214 | - | 233 | 0 | K.EVGADFILQISNESPQEIAK.K |
| 2311.3108 | 2310.3035 | 2310.2770 | 11.5 | 56 | - | 77 | 1 | R.IGDFVVKKPMVLGHEASGTVVK.V |
| 2387.2449 | 2386.2376 | 2386.2380 | -0.14 | 212 | - | 233 | 1 | K.AKEVGADFILQISNESPQEIAK.K |
| 2458.1199 | 2457.1126 | 2457.1093 | 1.34 | 110 | - | 130 | 0 | R.YNLSPTIFFCATPPDDGNLCR.F |
| 2637.3391 | 2636.3318 | 2636.3268 | 1.90 | 142 | - | 165 | 0 | K.LPDNVTFEEGALIEPLSVGIHACR.R |

**No match to:** 853.2671, 855.0451, 856.0256, 861.0641, 870.0215, 871.0246, 873.0248, 877.0393, 877.2717, 879.0370, 884.3177,

885.9973, 893.0132, 901.3589, 908.9853, 924.9604, 1034.1259, 1044.0710, 1050.0869, 1066.0652, 1067.4818, 1082.0472, 1098.0142, 1110.5029, 1136.5674, 1300.0475, 1306.7865, 1320.5912, 1642.7385, 1765.0378, 1779.0547, 1787.8590, 1921.0986, 1936.0398, 1983.9967, 1986.0781, 2043.1068, 2048.9885, 2139.9622, 2142.9429, 2146.0459, 2156.9597, 2170.0632, 2226.1350, 2256.1982, 2289.1533, 2330.1792, 2335.0740, 2472.1384

**2.** DHSO_SHEEP **Mass:** 38377 **Score:** 93 **Expect:** 3.7e-005 **Matches:** 12

Sorbitol dehydrogenase OS=Ovis aries GN=SORD PE=1 SV=1

| Observed | Mr(expt) | Mr(calc) | ppm | Start | | End | Miss | Peptide |
|---|---|---|---|---|---|---|---|---|
| 1053.4615 | 1052.4542 | 1052.4498 | 4.22 | 132 | - | 139 | 0 | K.HNANFCYK.L |
| 1249.7527 | 1248.7454 | 1248.7303 | 12.1 | 312 | - | 322 | 0 | K.SVNVKPLVTHR.F |
| 1552.8657 | 1551.8584 | 1551.8443 | 9.10 | 61 | - | 75 | 0 | K.KPMVLGHEASGTVVK.V |
| 1568.8557 | 1567.8484 | 1567.8392 | 5.87 | 61 | - | 75 | 0 | K.KPMVLGHEASGTVVK.V + Oxidation (M) |
| 1575.8231 | 1574.8158 | 1574.8086 | 4.56 | 191 | - | 206 | 0 | K.AMGAAQVVVTDLSASR.L |
| 1765.0378 | 1764.0305 | 1764.0332 | -1.49 | 173 | - | 190 | 0 | K.VLVCGAGPIGLVNLLAAK.A |
| 1772.8123 | 1771.8050 | 1771.8095 | -2.55 | 297 | - | 311 | 0 | R.YCNTWPMAISMLASK.S |
| 1864.0842 | 1863.0769 | 1863.0730 | 2.08 | 312 | - | 327 | 1 | K.SVNVKPLVTHRFPLEK.A |
| 1922.0380 | 1921.0307 | 1921.0196 | 5.77 | 20 | - | 36 | 0 | R.LENYPIPEPGPNEVLLK.M |
| 2311.3108 | 2310.3035 | 2310.2770 | 11.5 | 54 | - | 75 | 1 | R.IGDFVVKKPMVLGHEASGTVVK.V |
| 2458.1199 | 2457.1126 | 2457.1093 | 1.34 | 108 | - | 128 | 0 | R.YNLSPTIFFCATPPDDGNLCR.F |
| 2637.3391 | 2636.3318 | 2636.3268 | 1.90 | 140 | - | 163 | 0 | K.LPDNVTFEEGALIEPLSVGIHACR.R |

**No match to:** 853.2671, 855.0451, 856.0256, 861.0641, 870.0215, 871.0246, 873.0248, 877.0393, 877.2717, 879.0370, 884.3177, 885.9973, 893.0132, 901.3589, 908.9853, 924.9604, 1034.1259, 1044.0710, 1050.0869, 1066.0652, 1067.4818, 1082.0472, 1098.0142, 1110.5029, 1136.5674, 1300.0475, 1306.7865, 1320.5912, 1642.7385, 1712.9301, 1779.0547, 1787.8590, 1921.0986, 1936.0398, 1983.9967, 1986.0781, 2043.1068, 2048.9885, 2125.9543, 2139.9622, 2141.9475, 2142.9429, 2146.0459, 2156.9597, 2170.0632, 2188.1106, 2226.1350, 2256.1982, 2289.1533, 2330.1792, 2335.0740, 2387.2449, 2472.1384

**3.** TSNAX_MACFA **Mass:** 33163 **Score:** 28 **Expect:** 1e+002 **Matches:** 5

Translin-associated protein X OS=Macaca fascicularis GN=TSNAX PE=2 SV=1

| Observed | Mr(expt) | Mr(calc) | ppm | Start | | End | Miss | Peptide |
|---|---|---|---|---|---|---|---|---|
| 1067.4818 | 1066.4745 | 1066.5117 | -34.86 | 253 | - | 261 | 0 | K.VENACYALK.V |
| 1320.5912 | 1319.5839 | 1319.6232 | -29.73 | 15 | - | 24 | 1 | K.HDNFPHNQRR.E |
| 2125.9543 | 2124.9470 | 2124.9348 | 5.73 | 162 | - | 179 | 1 | K.ENKTPSSDTQDEQFGTWR.L |
| 2188.1106 | 2187.1033 | 2187.0167 | 39.6 | 101 | - | 118 | 0 | K.IFQVAQELSGEDMHQFHR.A + Oxidation (M) |
| 2335.0740 | 2334.0667 | 2334.0719 | -2.22 | 270 | - | 290 | 1 | K.HMLADVSSVKTEMIDQEEGIS.- + Oxidation (M) |

**No match to:** 853.2671, 855.0451, 856.0256, 861.0641, 870.0215, 871.0246, 873.0248, 877.0393, 877.2717, 879.0370, 884.3177, 885.9973, 893.0132, 901.3589, 908.9853, 924.9604, 1034.1259, 1044.0710, 1050.0869, 1053.4615, 1066.0652, 1082.0472, 1098.0142, 1110.5029, 1136.5674, 1249.7527, 1300.0475, 1306.7865, 1552.8657, 1568.8557, 1575.8231, 1642.7385, 1712.9301, 1765.0378, 1772.8123, 1779.0547, 1787.8590, 1864.0842, 1921.0986, 1922.0380, 1936.0398, 1983.9967, 1986.0781, 2043.1068, 2048.9885, 2139.9622, 2141.9475, 2142.9429, 2146.0459, 2156.9597, 2170.0632, 2226.1350, 2256.1982, 2289.1533, 2311.3108, 2330.1792, 2387.2449, 2458.1199, 2472.1384, 2637.3391

**4.** F8I2_HUMAN **Mass:** 39478 **Score:** 27 **Expect:** 1.4e+002 **Matches:** 4

Factor VIII intron 22 protein OS=Homo sapiens GN=F8A1 PE=1 SV=2

| Observed | Mr(expt) | Mr(calc) | ppm | Start | | End | Miss | Peptide |
|---|---|---|---|---|---|---|---|---|
| 2139.9622 | 2138.9549 | 2139.0531 | -45.90 | 76 | - | 95 | 0 | R.CQQALFHGPGEALALTEAAR.L |
| 2141.9475 | 2140.9402 | 2141.0324 | -43.04 | 1 | - | 25 | 0 | -.MAAAAAGLGGGGAGPGPEAGDFLAR.Y |
| 2256.1982 | 2255.1909 | 2255.1732 | 7.86 | 108 | - | 130 | 0 | R.LVCPAAYGEPLQAAASALGAAVR.L |
| 2330.1792 | 2329.1719 | 2329.1563 | 6.70 | 2 | - | 27 | 1 | M.AAAAAGLGGGGAGPGPEAGDFLARYR.L |

**No match to:** 853.2671, 855.0451, 856.0256, 861.0641, 870.0215, 871.0246, 873.0248, 877.0393, 877.2717, 879.0370, 884.3177, 885.9973, 893.0132, 901.3589, 908.9853, 924.9604, 1034.1259, 1044.0710, 1050.0869, 1053.4615, 1066.0652, 1067.4818, 1082.0472, 1098.0142, 1110.5029, 1136.5674, 1249.7527, 1300.0475, 1306.7865, 1320.5912, 1552.8657, 1568.8557, 1575.8231, 1642.7385, 1712.9301, 1765.0378, 1772.8123, 1779.0547, 1787.8590, 1864.0842, 1921.0986, 1922.0380, 1936.0398, 1983.9967, 1986.0781, 2043.1068, 2048.9885, 2125.9543, 2142.9429, 2146.0459, 2156.9597, 2170.0632, 2188.1106, 2226.1350, 2289.1533, 2311.3108, 2335.0740, 2387.2449, 2458.1199, 2472.1384, 2637.3391

**5.** PNMT_HUMAN **Mass:** 31178 **Score:** 27 **Expect:** 1.4e+002 **Matches:** 4

Phenylethanolamine N-methyltransferase OS=Homo sapiens GN=PNMT PE=1 SV=1

| Observed | Mr(expt) | Mr(calc) | ppm | Start | | End | Miss | Peptide |
|---|---|---|---|---|---|---|---|---|
| 1787.8590 | 1786.8517 | 1786.8924 | -22.75 | 255 | - | 270 | 0 | R.TYIMPAHLQTGVDDVK.G |
| 2156.9597 | 2155.9524 | 2156.0246 | -33.49 | 7 | - | 29 | 0 | R.SPNAGAAPDSAPGQAAVASAYQR.F |
| 2188.1106 | 2187.1033 | 2187.0994 | 1.79 | 252 | - | 270 | 1 | R.DLRTYIMPAHLQTGVDDVK.G + Oxidation (M) |
| 2226.1350 | 2225.1277 | 2225.0436 | 37.8 | 38 | - | 57 | 1 | R.NNYAPPRGDLCNPNGVGPWK.L |

**No match to:** 853.2671, 855.0451, 856.0256, 861.0641, 870.0215, 871.0246, 873.0248, 877.0393, 877.2717, 879.0370, 884.3177, 885.9973, 893.0132, 901.3589, 908.9853, 924.9604, 1034.1259, 1044.0710, 1050.0869, 1053.4615, 1066.0652, 1067.4818, 1082.0472, 1098.0142, 1110.5029, 1136.5674, 1249.7527, 1300.0475, 1306.7865, 1320.5912, 1552.8657, 1568.8557, 1575.8231, 1642.7385, 1712.9301, 1765.0378, 1772.8123, 1779.0547, 1864.0842, 1921.0986, 1922.0380, 1936.0398, 1983.9967, 1986.0781, 2043.1068, 2048.9885, 2125.9543, 2139.9622, 2141.9475, 2142.9429, 2146.0459, 2170.0632, 2256.1982, 2289.1533, 2311.3108, 2330.1792, 2335.0740, 2387.2449, 2458.1199, 2472.1384, 2637.3391

**6.** OVGP1_BOVIN **Mass:** 59922 **Score:** 26 **Expect:** 1.5e+002 **Matches:** 6

Oviduct-specific glycoprotein (Fragment) OS=Bos taurus GN=OVGP1 PE=1 SV=1

| Observed | Mr(expt) | Mr(calc) | ppm | Start | | End | Miss | Peptide |
|---|---|---|---|---|---|---|---|---|
| 1136.5674 | 1135.5601 | 1135.6060 | -40.36 | 253 | - | 262 | 0 | K.LLMGLPTYGR.T + Oxidation (M) |
| 1320.5912 | 1319.5839 | 1319.6180 | -25.82 | 104 | - | 114 | 0 | R.FTTMLSTFSNR.E + Oxidation (M) |
| 1568.8557 | 1567.8484 | 1567.8107 | 24.1 | 453 | - | 467 | 1 | R.TPLSFGRHTAAPEGK.T |
| 1575.8231 | 1574.8158 | 1574.7722 | 27.7 | 522 | - | 535 | 1 | K.MTVTPDGRAETLER.R |
| 2256.1982 | 2255.1909 | 2255.0939 | 43.0 | 324 | - | 342 | 1 | K.EWVGYDDAISFGYKAFFIK.R |
| 2289.1533 | 2288.1460 | 2288.0758 | 30.7 | 287 | - | 304 | 1 | K.YTKQAGFLAYYEICCFVR.R |

**No match to:** 853.2671, 855.0451, 856.0256, 861.0641, 870.0215, 871.0246, 873.0248, 877.0393, 877.2717, 879.0370, 884.3177, 885.9973, 893.0132, 901.3589, 908.9853, 924.9604, 1034.1259, 1044.0710, 1050.0869, 1053.4615, 1066.0652, 1067.4818, 1082.0472, 1098.0142, 1110.5029, 1249.7527, 1300.0475, 1306.7865, 1552.8657, 1642.7385, 1712.9301, 1765.0378, 1772.8123, 1779.0547, 1787.8590, 1864.0842, 1921.0986, 1922.0380, 1936.0398, 1983.9967, 1986.0781, 2043.1068, 2048.9885, 2125.9543, 2139.9622, 2141.9475, 2142.9429, 2146.0459, 2156.9597, 2170.0632, 2188.1106, 2226.1350, 2311.3108, 2330.1792, 2335.0740, 2387.2449, 2458.1199, 2472.1384, 2637.3391

7. PKHA7_HUMAN **Mass:** 127626 **Score:** 26 **Expect:** 1.8e+002 **Matches:** 8

Pleckstrin homology domain-containing family A member 7 OS=Homo sapiens GN=PLEKHA7 PE=1 SV=2

| Observed | Mr(expt) | Mr(calc) | ppm | Start | | End | Miss | Peptide |
|---|---|---|---|---|---|---|---|---|
| 1136.5674 | 1135.5601 | 1135.6125 | -46.08 | 661 | - | 669 | 1 | K.KDLEYLDLK.M |
| 1787.8590 | 1786.8517 | 1786.8429 | 4.94 | 186 | - | 198 | 0 | R.WFVLADYCLFYYK.D |
| 1983.9967 | 1982.9894 | 1983.0762 | -43.77 | 586 | - | 602 | 1 | R.RPHTPAERVTVKPPDQR.R |
| 2125.9543 | 2124.9470 | 2124.9997 | -24.81 | 686 | - | 703 | 1 | K.IAESDTDVKLSIFCEQDR.V |
| 2146.0459 | 2145.0386 | 2145.0030 | 16.6 | 186 | - | 201 | 1 | R.WFVLADYCLFYYKDSR.E |
| 2170.0632 | 2169.0559 | 2168.9545 | 46.7 | 394 | - | 414 | 0 | K.NGMLPASYGPGEQNGTGGYQR.A + Oxidation (M) |
| 2289.1533 | 2288.1460 | 2288.1219 | 10.5 | 720 | - | 737 | 1 | K.DQLESVLEVLHRQMEQYR.D + Oxidation (M) |
| 2335.0740 | 2334.0667 | 2334.1604 | -40.14 | 458 | - | 478 | 1 | R.QGPGQSLSFPENYQTLPKSTR.H |

**No match to:** 853.2671, 855.0451, 856.0256, 861.0641, 870.0215, 871.0246, 873.0248, 877.0393, 877.2717, 879.0370, 884.3177, 885.9973, 893.0132, 901.3589, 908.9853, 924.9604, 1034.1259, 1044.0710, 1050.0869, 1053.4615, 1066.0652, 1067.4818, 1082.0472, 1098.0142, 1110.5029, 1249.7527, 1300.0475, 1306.7865, 1320.5912, 1552.8657, 1568.8557, 1575.8231, 1642.7385, 1712.9301, 1765.0378, 1772.8123, 1779.0547, 1864.0842, 1921.0986, 1922.0380, 1936.0398, 1986.0781, 2043.1068, 2048.9885, 2139.9622, 2141.9475, 2142.9429, 2156.9597, 2188.1106, 2226.1350, 2256.1982, 2311.3108, 2330.1792, 2387.2449, 2458.1199, 2472.1384, 2637.3391

8. F107B_HUMAN **Mass:** 15548 **Score:** 25 **Expect:** 2.1e+002 **Matches:** 3

Protein FAM107B OS=Homo sapiens GN=FAM107B PE=1 SV=1

| Observed | Mr(expt) | Mr(calc) | ppm | Start | | End | Miss | Peptide |
|---|---|---|---|---|---|---|---|---|
| 1787.8590 | 1786.8517 | 1786.9101 | -32.67 | 101 | - | 115 | 1 | K.LQEEQENAPEFVKVK.G |
| 2141.9475 | 2140.9402 | 2141.0276 | -40.83 | 2 | - | 19 | 0 | M.AEPDYIEDDNPELIRPQK.L |
| 2289.1533 | 2288.1460 | 2288.0630 | 36.3 | 1 | - | 19 | 0 | -.MAEPDYIEDDNPELIRPQK.L + Oxidation (M) |

**No match to:** 853.2671, 855.0451, 856.0256, 861.0641, 870.0215, 871.0246, 873.0248, 877.0393, 877.2717, 879.0370, 884.3177, 885.9973, 893.0132, 901.3589, 908.9853, 924.9604, 1034.1259, 1044.0710, 1050.0869, 1053.4615, 1066.0652, 1067.4818, 1082.0472, 1098.0142, 1110.5029, 1136.5674, 1249.7527, 1300.0475, 1306.7865, 1320.5912, 1552.8657, 1568.8557, 1575.8231, 1642.7385, 1712.9301, 1765.0378, 1772.8123, 1779.0547, 1864.0842, 1921.0986, 1922.0380, 1936.0398, 1983.9967, 1986.0781, 2043.1068, 2048.9885, 2125.9543, 2139.9622, 2142.9429, 2146.0459, 2156.9597, 2170.0632, 2188.1106, 2226.1350, 2256.1982, 2311.3108, 2330.1792, 2335.0740, 2387.2449, 2458.1199, 2472.1384, 2637.3391

9. F107B_MOUSE **Mass:** 15562 **Score:** 25 **Expect:** 2.1e+002 **Matches:** 3

Protein FAM107B OS=Mus musculus GN=Fam107b PE=1 SV=2

| Observed | Mr(expt) | Mr(calc) | ppm | Start | | End | Miss | Peptide |
|---|---|---|---|---|---|---|---|---|
| 1787.8590 | 1786.8517 | 1786.9101 | -32.67 | 101 | - | 115 | 1 | K.LQEEQENAPEFVKVK.G |
| 2141.9475 | 2140.9402 | 2141.0276 | -40.83 | 2 | - | 19 | 0 | M.AEPDYIEDDNPELIRPQK.L |
| 2289.1533 | 2288.1460 | 2288.0630 | 36.3 | 1 | - | 19 | 0 | -.MAEPDYIEDDNPELIRPQK.L + Oxidation (M) |

**No match to:** 853.2671, 855.0451, 856.0256, 861.0641, 870.0215, 871.0246, 873.0248, 877.0393, 877.2717, 879.0370, 884.3177, 885.9973, 893.0132, 901.3589, 908.9853, 924.9604, 1034.1259, 1044.0710, 1050.0869, 1053.4615, 1066.0652, 1067.4818, 1082.0472, 1098.0142, 1110.5029, 1136.5674, 1249.7527, 1300.0475, 1306.7865, 1320.5912, 1552.8657, 1568.8557, 1575.8231, 1642.7385, 1712.9301, 1765.0378, 1772.8123, 1779.0547, 1864.0842, 1921.0986, 1922.0380, 1936.0398, 1983.9967, 1986.0781, 2043.1068, 2048.9885, 2125.9543, 2139.9622, 2142.9429, 2146.0459, 2156.9597, 2170.0632, 2188.1106, 2226.1350, 2256.1982, 2311.3108, 2330.1792, 2335.0740, 2387.2449, 2458.1199, 2472.1384, 2637.3391

10. F107B_RAT **Mass:** 15576 **Score:** 25 **Expect:** 2.1e+002 **Matches:** 3

Protein FAM107B OS=Rattus norvegicus GN=Fam107b PE=2 SV=1

| Observed | Mr(expt) | Mr(calc) | ppm | Start | | End | Miss | Peptide |
|---|---|---|---|---|---|---|---|---|
| 1787.8590 | 1786.8517 | 1786.9101 | -32.67 | 101 | - | 115 | 1 | K.LQEEQENAPEFVKVK.G |
| 2141.9475 | 2140.9402 | 2141.0276 | -40.83 | 2 | - | 19 | 0 | M.AEPDYIEDDNPELIRPQK.L |
| 2289.1533 | 2288.1460 | 2288.0630 | 36.3 | 1 | - | 19 | 0 | -.MAEPDYIEDDNPELIRPQK.L + Oxidation (M) |

**No match to:** 853.2671, 855.0451, 856.0256, 861.0641, 870.0215, 871.0246, 873.0248, 877.0393, 877.2717, 879.0370, 884.3177, 885.9973, 893.0132, 901.3589, 908.9853, 924.9604, 1034.1259, 1044.0710, 1050.0869, 1053.4615, 1066.0652, 1067.4818, 1082.0472, 1098.0142, 1110.5029, 1136.5674, 1249.7527, 1300.0475, 1306.7865, 1320.5912, 1552.8657, 1568.8557, 1575.8231, 1642.7385, 1712.9301, 1765.0378, 1772.8123, 1779.0547, 1864.0842, 1921.0986, 1922.0380, 1936.0398, 1983.9967, 1986.0781, 2043.1068, 2048.9885, 2125.9543, 2139.9622, 2142.9429, 2146.0459, 2156.9597, 2170.0632, 2188.1106, 2226.1350, 2256.1982, 2311.3108, 2330.1792, 2335.0740, 2387.2449, 2458.1199, 2472.1384, 2637.3391

11. KR204_HUMAN **Mass:** 4791 **Score:** 25 **Expect:** 2.1e+002 **Matches:** 2

Putative keratin-associated protein 20-4 OS=Homo sapiens GN=KRTAP20-4 PE=3 SV=1

| Observed | Mr(expt) | Mr(calc) | ppm | Start | | End | Miss | Peptide |
|---|---|---|---|---|---|---|---|---|
| 2256.1982 | 2255.1909 | 2255.0827 | 48.0 | 2 | - | 23 | 0 | M.SYYSHLSGGLGCGLAVAVTMGR.T |
| 2387.2449 | 2386.2376 | 2386.1232 | 48.0 | 1 | - | 23 | 0 | -.MSYYSHLSGGLGCGLAVAVTMGR.T |

**No match to:** 853.2671, 855.0451, 856.0256, 861.0641, 870.0215, 871.0246, 873.0248, 877.0393, 877.2717, 879.0370, 884.3177, 885.9973, 893.0132, 901.3589, 908.9853, 924.9604, 1034.1259, 1044.0710, 1050.0869, 1053.4615, 1066.0652, 1067.4818, 1082.0472, 1098.0142, 1110.5029, 1136.5674, 1249.7527, 1300.0475, 1306.7865, 1320.5912, 1552.8657, 1568.8557, 1575.8231, 1642.7385, 1712.9301, 1765.0378, 1772.8123, 1779.0547, 1787.8590, 1864.0842, 1921.0986, 1922.0380, 1936.0398, 1983.9967, 1986.0781, 2043.1068, 2048.9885, 2125.9543, 2139.9622, 2141.9475, 2142.9429, 2146.0459, 2156.9597, 2170.0632, 2188.1106, 2226.1350, 2289.1533, 2311.3108, 2330.1792, 2335.0740, 2458.1199, 2472.1384, 2637.3391

12. PATE2_HUMAN **Mass:** 13633 **Score:** 25 **Expect:** 2.2e+002 **Matches:** 3

Prostate and testis expressed protein 2 OS=Homo sapiens GN=PATE2 PE=2 SV=1

| Observed | Mr(expt) | Mr(calc) | ppm | Start | | End | Miss | Peptide |
|---|---|---|---|---|---|---|---|---|
| 1320.5912 | 1319.5839 | 1319.5196 | 48.8 | 26 | - | 35 | 0 | K.ATEIMCYECK.K + Oxidation (M) |
| 2170.0632 | 2169.0559 | 2169.0169 | 18.0 | 37 | - | 54 | 1 | K.YHLGLCYGVMTSCSLKHK.Q + Oxidation (M) |
| 2335.0740 | 2334.0667 | 2334.0191 | 20.4 | 95 | - | 113 | 1 | K.RVELICCDHSNYCNLPEGV.- |

**No match to:** 853.2671, 855.0451, 856.0256, 861.0641, 870.0215, 871.0246, 873.0248, 877.0393, 877.2717, 879.0370, 884.3177, 885.9973, 893.0132, 901.3589, 908.9853, 924.9604, 1034.1259, 1044.0710, 1050.0869, 1053.4615, 1066.0652, 1067.4818, 1082.0472, 1098.0142, 1110.5029, 1136.5674, 1249.7527, 1300.0475, 1306.7865, 1552.8657, 1568.8557, 1575.8231, 1642.7385, 1712.9301, 1765.0378, 1772.8123, 1779.0547, 1787.8590, 1864.0842, 1921.0986, 1922.0380, 1936.0398, 1983.9967, 1986.0781, 2043.1068, 2048.9885, 2125.9543, 2139.9622, 2141.9475, 2142.9429, 2146.0459, 2156.9597, 2188.1106, 2226.1350, 2256.1982, 2289.1533, 2311.3108, 2330.1792, 2387.2449, 2458.1199, 2472.1384, 2637.3391

13. MAGF1_HUMAN **Mass:** 35200 **Score:** 24 **Expect:** 2.5e+002 **Matches:** 4

Melanoma-associated antigen F1 OS=Homo sapiens GN=MAGEF1 PE=1 SV=2

| Observed | Mr(expt) | Mr(calc) | ppm | Start | | End | Miss | Peptide |
|---|---|---|---|---|---|---|---|---|

| | | | | | | | |
|---|---|---|---|---|---|---|---|
| 1765.0378 | 1764.0305 | 1763.9491 | 46.1 | 245 - | 260 | 1 | R.SNLEISKMEVLGFVAK.L |
| 1936.0398 | 1935.0325 | 1935.0061 | 13.6 | 2 - | 19 | 1 | M.LQTPESRGLPVPQAEGEK.D |
| 2048.9885 | 2047.9812 | 2047.9559 | 12.4 | 9 - | 28 | 1 | R.GLPVPQAEGEKDGGHDGETR.A |
| 2170.0632 | 2169.0559 | 2169.1472 | -42.07 | 167 - | 185 | 1 | R.LGLLMMILGLIYMRGNSAR.E + 3 Oxidation (M) |

**No match to:** 853.2671, 855.0451, 856.0256, 861.0641, 870.0215, 871.0246, 873.0248, 877.0393, 877.2717, 879.0370, 884.3177, 885.9973, 893.0132, 901.3589, 908.9853, 924.9604, 1034.1259, 1044.0710, 1050.0869, 1053.4615, 1066.0652, 1067.4818, 1082.0472, 1098.0142, 1110.5029, 1136.5674, 1249.7527, 1300.0475, 1306.7865, 1320.5912, 1552.8657, 1568.8557, 1575.8231, 1642.7385, 1712.9301, 1772.8123, 1779.0547, 1787.8590, 1864.0842, 1921.0986, 1922.0380, 1983.9967, 1986.0781, 2043.1068, 2125.9543, 2139.9622, 2141.9475, 2142.9429, 2146.0459, 2156.9597, 2188.1106, 2226.1350, 2256.1982, 2289.1533, 2311.3108, 2330.1792, 2335.0740, 2387.2449, 2458.1199, 2472.1384, 2637.3391

**14.** TLR2_GORGO **Mass:** 90906 **Score:** 24 **Expect:** 2.5e+002 **Matches:** 7

Toll-like receptor 2 OS=Gorilla gorilla gorilla GN=TLR2 PE=2 SV=1

| Observed | Mr(expt) | Mr(calc) | ppm | Start | End | Miss | Peptide |
|---|---|---|---|---|---|---|---|
| 1864.0842 | 1863.0769 | 1863.0400 | 19.8 | 193 - | 208 | 1 | K.SLKSIQNVSHLILHMK.Q + Oxidation (M) |
| 2125.9543 | 2124.9470 | 2124.9972 | -23.62 | 755 - | 771 | 1 | K.IMNTKTYLEWPMDEAQR.E |
| 2141.9475 | 2140.9402 | 2140.9921 | -24.24 | 755 - | 771 | 1 | K.IMNTKTYLEWPMDEAQR.E + Oxidation (M) |
| 2170.0632 | 2169.0559 | 2169.1568 | -46.53 | 724 - | 742 | 0 | R.LFDENNDAAILILLEPIEK.K |
| 2188.1106 | 2187.1033 | 2187.0656 | 17.2 | 361 - | 378 | 0 | K.SLEYLDLSENLMVEEYLK.N |
| 2226.1350 | 2225.1277 | 2225.2105 | -37.19 | 1 - | 19 | 0 | -.MPHTLWMVWVLGVIISLSK.E + Oxidation (M) |
| 2256.1982 | 2255.1909 | 2255.2997 | -48.21 | 489 - | 508 | 1 | K.LMTLPDASLLPMLLVLKISR.N + 2 Oxidation (M) |

**No match to:** 853.2671, 855.0451, 856.0256, 861.0641, 870.0215, 871.0246, 873.0248, 877.0393, 877.2717, 879.0370, 884.3177, 885.9973, 893.0132, 901.3589, 908.9853, 924.9604, 1034.1259, 1044.0710, 1050.0869, 1053.4615, 1066.0652, 1067.4818, 1082.0472, 1098.0142, 1110.5029, 1136.5674, 1249.7527, 1300.0475, 1306.7865, 1320.5912, 1552.8657, 1568.8557, 1575.8231, 1642.7385, 1712.9301, 1765.0378, 1772.8123, 1779.0547, 1787.8590, 1921.0986, 1922.0380, 1936.0398, 1983.9967, 1986.0781, 2043.1068, 2048.9885, 2139.9622, 2142.9429, 2146.0459, 2156.9597, 2289.1533, 2311.3108, 2330.1792, 2335.0740, 2387.2449, 2458.1199, 2472.1384, 2637.3391

**15.** TLR2_HUMAN **Mass:** 90920 **Score:** 24 **Expect:** 2.5e+002 **Matches:** 7

Toll-like receptor 2 OS=Homo sapiens GN=TLR2 PE=1 SV=1

| Observed | Mr(expt) | Mr(calc) | ppm | Start | End | Miss | Peptide |
|---|---|---|---|---|---|---|---|
| 1864.0842 | 1863.0769 | 1863.0400 | 19.8 | 193 - | 208 | 1 | K.SLKSIQNVSHLILHMK.Q + Oxidation (M) |
| 2125.9543 | 2124.9470 | 2124.9972 | -23.62 | 755 - | 771 | 1 | K.IMNTKTYLEWPMDEAQR.E |
| 2141.9475 | 2140.9402 | 2140.9921 | -24.24 | 755 - | 771 | 1 | K.IMNTKTYLEWPMDEAQR.E + Oxidation (M) |
| 2170.0632 | 2169.0559 | 2169.1568 | -46.53 | 724 - | 742 | 0 | R.LFDENNDAAILILLEPIEK.K |
| 2188.1106 | 2187.1033 | 2187.0656 | 17.2 | 361 - | 378 | 0 | K.SLEYLDLSENLMVEEYLK.N |
| 2226.1350 | 2225.1277 | 2225.2105 | -37.19 | 1 - | 19 | 0 | -.MPHTLWMVWVLGVIISLSK.E + Oxidation (M) |
| 2256.1982 | 2255.1909 | 2255.2997 | -48.21 | 489 - | 508 | 1 | K.LMTLPDASLLPMLLVLKISR.N + 2 Oxidation (M) |

**No match to:** 853.2671, 855.0451, 856.0256, 861.0641, 870.0215, 871.0246, 873.0248, 877.0393, 877.2717, 879.0370, 884.3177, 885.9973, 893.0132, 901.3589, 908.9853, 924.9604, 1034.1259, 1044.0710, 1050.0869, 1053.4615, 1066.0652, 1067.4818, 1082.0472, 1098.0142, 1110.5029, 1136.5674, 1249.7527, 1300.0475, 1306.7865, 1320.5912, 1552.8657, 1568.8557, 1575.8231, 1642.7385, 1712.9301, 1765.0378, 1772.8123, 1779.0547, 1787.8590, 1921.0986, 1922.0380, 1936.0398, 1983.9967, 1986.0781, 2043.1068, 2048.9885, 2139.9622, 2142.9429, 2146.0459, 2156.9597, 2289.1533, 2311.3108, 2330.1792, 2335.0740, 2387.2449, 2458.1199, 2472.1384, 2637.3391

**16.** TLR2_MACMU **Mass:** 91154 **Score:** 24 **Expect:** 2.8e+002 **Matches:** 6

Toll-like receptor 2 OS=Macaca mulatta GN=TLR2 PE=2 SV=1

| Observed | Mr(expt) | Mr(calc) | ppm | Start | End | Miss | Peptide |
|---|---|---|---|---|---|---|---|
| 1864.0842 | 1863.0769 | 1863.0400 | 19.8 | 193 - | 208 | 1 | K.SLKSIQNVSHLILHMK.Q + Oxidation (M) |
| 1936.0398 | 1935.0325 | 1935.1153 | -42.77 | 396 - | 413 | 1 | R.QNHLASLGKIGETLLTLK.N |
| 1986.0781 | 1985.0708 | 1985.1408 | -35.26 | 405 - | 422 | 1 | K.IGETLLTLKNLTNLDISK.N |
| 2188.1106 | 2187.1033 | 2187.0656 | 17.2 | 361 - | 378 | 0 | K.SLEYLDLSENLMVEEYLK.N |
| 2226.1350 | 2225.1277 | 2225.2105 | -37.19 | 1 - | 19 | 0 | -.MPHTLWMVWVLGVIISLSK.E + Oxidation (M) |
| 2256.1982 | 2255.1909 | 2255.2997 | -48.21 | 489 - | 508 | 1 | K.LMTLPDASLLPMLLVLKISR.N + 2 Oxidation (M) |

**No match to:** 853.2671, 855.0451, 856.0256, 861.0641, 870.0215, 871.0246, 873.0248, 877.0393, 877.2717, 879.0370, 884.3177, 885.9973, 893.0132, 901.3589, 908.9853, 924.9604, 1034.1259, 1044.0710, 1050.0869, 1053.4615, 1066.0652, 1067.4818, 1082.0472, 1098.0142, 1110.5029, 1136.5674, 1249.7527, 1300.0475, 1306.7865, 1320.5912, 1552.8657, 1568.8557, 1575.8231, 1642.7385, 1712.9301, 1765.0378, 1772.8123, 1779.0547, 1787.8590, 1921.0986, 1922.0380, 1983.9967, 2043.1068, 2048.9885, 2125.9543, 2139.9622, 2141.9475, 2142.9429, 2146.0459, 2156.9597, 2170.0632, 2289.1533, 2311.3108, 2330.1792, 2335.0740, 2387.2449, 2458.1199, 2472.1384, 2637.3391

**17.** GT2D2_BOVIN **Mass:** 108145 **Score:** 24 **Expect:** 2.9e+002 **Matches:** 7

General transcription factor II-I repeat domain-containing protein 2 OS=Bos taurus GN=GTF2IRD2 PE=2 SV=1

| Observed | Mr(expt) | Mr(calc) | ppm | Start | End | Miss | Peptide |
|---|---|---|---|---|---|---|---|
| 2043.1068 | 2042.0995 | 2042.0111 | 43.3 | 651 - | 667 | 1 | K.SVCCIIHPESLCAQKLK.M |
| 2125.9543 | 2124.9470 | 2125.0110 | -30.09 | 1 - | 20 | 0 | -.MAQVAVSTPPIAHEESSESR.M |
| 2141.9475 | 2140.9402 | 2141.0059 | -30.66 | 1 - | 20 | 0 | -.MAQVAVSTPPIAHEESSESR.M + Oxidation (M) |
| 2146.0459 | 2145.0386 | 2145.0988 | -28.03 | 614 - | 635 | 0 | K.LVSVASTGTPAMVDANDGLVTK.L |
| 2170.0632 | 2169.0559 | 2169.0776 | -10.00 | 497 - | 515 | 1 | K.YLLGSSDTVCPEQKQVFAK.V |
| 2188.1106 | 2187.1033 | 2187.0989 | 2.01 | 21 - | 39 | 1 | R.MVVTFLVSALESMCKELAK.S + 2 Oxidation (M) |
| 2387.2449 | 2386.2376 | 2386.2778 | -16.82 | 614 - | 637 | 1 | K.LVSVASTGTPAMVDANDGLVTKLK.S |

**No match to:** 853.2671, 855.0451, 856.0256, 861.0641, 870.0215, 871.0246, 873.0248, 877.0393, 877.2717, 879.0370, 884.3177, 885.9973, 893.0132, 901.3589, 908.9853, 924.9604, 1034.1259, 1044.0710, 1050.0869, 1053.4615, 1066.0652, 1067.4818, 1082.0472, 1098.0142, 1110.5029, 1136.5674, 1249.7527, 1300.0475, 1306.7865, 1320.5912, 1552.8657, 1568.8557, 1575.8231, 1642.7385, 1712.9301, 1765.0378, 1772.8123, 1779.0547, 1787.8590, 1864.0842, 1921.0986, 1922.0380, 1936.0398, 1983.9967, 1986.0781, 2048.9885, 2139.9622, 2142.9429, 2156.9597, 2226.1350, 2256.1982, 2289.1533, 2311.3108, 2330.1792, 2335.0740, 2458.1199, 2472.1384, 2637.3391

**18.** KPYM_PONAB **Mass:** 58494 **Score:** 24 **Expect:** 2.9e+002 **Matches:** 6

Pyruvate kinase PKM OS=Pongo abelii GN=PKM PE=2 SV=3

| Observed | Mr(expt) | Mr(calc) | ppm | Start | End | Miss | Peptide |
|---|---|---|---|---|---|---|---|
| 1053.4615 | 1052.4542 | 1052.4678 | -12.86 | 312 - | 319 | 1 | K.MMIGRCNR.A + Oxidation (M) |
| 1642.7385 | 1641.7312 | 1641.7635 | -19.63 | 476 - | 489 | 0 | K.DPVQEAWAEDVDLR.V |
| 1765.0378 | 1764.0305 | 1763.9781 | 29.7 | 207 - | 224 | 1 | K.KGVNLPGAAVDLPAVSEK.D |

| | | | | | | | |
|---|---|---|---|---|---|---|---|
| 1922.0380 | 1921.0307 | 1920.9793 | 26.8 | 189 - 207 | 1 | K.GADFLLTEVENGGSLGSKK.G |
| 1983.9967 | 1982.9894 | 1982.9230 | 33.5 | 152 - 166 | 1 | K.CDENILWLDYKNICK.V |
| 2335.0740 | 2334.0667 | 2333.9814 | 36.6 | 401 - 422 | 0 | R.ASSHSTDLMEAMAMGSVEASYK.C + 2 Oxidation (M) |

**No match to:** 853.2671, 855.0451, 856.0256, 861.0641, 870.0215, 871.0246, 873.0248, 877.0393, 877.2717, 879.0370, 884.3177, 885.9973, 893.0132, 901.3589, 908.9853, 924.9604, 1034.1259, 1044.0710, 1050.0869, 1066.0652, 1067.4818, 1082.0472, 1098.0142, 1110.5029, 1136.5674, 1249.7527, 1300.0475, 1306.7865, 1320.5912, 1552.8657, 1568.8557, 1575.8231, 1712.9301, 1772.8123, 1779.0547, 1787.8590, 1864.0842, 1921.0986, 1936.0398, 1986.0781, 2043.1068, 2048.9885, 2125.9543, 2139.9622, 2141.9475, 2142.9429, 2146.0459, 2156.9597, 2170.0632, 2188.1106, 2226.1350, 2256.1982, 2289.1533, 2311.3108, 2330.1792, 2387.2449, 2458.1199, 2472.1384, 2637.3391

**19.** WDR76_PAPAN **Mass:** 70432 **Score:** 23 **Expect:** 3e+002 **Matches:** 5

WD repeat-containing protein 76 OS=Papio anubis GN=WDR76 PE=3 SV=1

| Observed | Mr(expt) | Mr(calc) | ppm | Start - End | Miss | Peptide |
|---|---|---|---|---|---|---|
| 1936.0398 | 1935.0325 | 1934.9825 | 25.8 | 266 - 282 | 1 | R.FKGFLHTWAGMNKPSSK.N |
| 2170.0632 | 2169.0559 | 2169.0338 | 10.2 | 170 - 189 | 0 | K.NISENADFFASLQLSESAAR.L |
| 2330.1792 | 2329.1719 | 2329.2464 | -31.99 | 328 - 349 | 1 | R.TLVAVGAKFGQVGLCDLTQQPK.E |
| 2387.2449 | 2386.2376 | 2386.1701 | 28.3 | 49 - 68 | 0 | K.VYLAPFSLSNYQLDQLMCPK.S |
| 2458.1199 | 2457.1126 | 2457.1352 | -9.18 | 584 - 605 | 0 | R.VHSFGGECLVSVCSINAMHPTR.Y |

**No match to:** 853.2671, 855.0451, 856.0256, 861.0641, 870.0215, 871.0246, 873.0248, 877.0393, 877.2717, 879.0370, 884.3177, 885.9973, 893.0132, 901.3589, 908.9853, 924.9604, 1034.1259, 1044.0710, 1050.0869, 1053.4615, 1066.0652, 1067.4818, 1082.0472, 1098.0142, 1110.5029, 1136.5674, 1249.7527, 1300.0475, 1306.7865, 1320.5912, 1552.8657, 1568.8557, 1575.8231, 1642.7385, 1712.9301, 1765.0378, 1772.8123, 1779.0547, 1787.8590, 1864.0842, 1921.0986, 1922.0380, 1983.9967, 1986.0781, 2043.1068, 2048.9885, 2125.9543, 2139.9622, 2141.9475, 2142.9429, 2146.0459, 2156.9597, 2188.1106, 2226.1350, 2256.1982, 2289.1533, 2311.3108, 2335.0740, 2472.1384, 2637.3391

**20.** CRKL_MOUSE **Mass:** 33923 **Score:** 23 **Expect:** 3.3e+002 **Matches:** 4

Crk-like protein OS=Mus musculus GN=Crkl PE=1 SV=2

| Observed | Mr(expt) | Mr(calc) | ppm | Start - End | Miss | Peptide |
|---|---|---|---|---|---|---|
| 1568.8557 | 1567.8484 | 1567.8583 | -6.28 | 58 - 70 | 1 | R.VSHYIINSLPNRR.F |
| 1575.8231 | 1574.8158 | 1574.8127 | 2.00 | 167 - 180 | 1 | K.DGRVGMIPVPYVEK.L + Oxidation (M) |
| 2311.3108 | 2310.3035 | 2310.2372 | 28.7 | 146 - 164 | 1 | K.KGELLVIIEKPEEQWWSAR.N |
| 2330.1792 | 2329.1719 | 2329.1015 | 30.2 | 284 - 303 | 1 | K.GLFPFTHVKIFDPQNPDDNE.- |

**No match to:** 853.2671, 855.0451, 856.0256, 861.0641, 870.0215, 871.0246, 873.0248, 877.0393, 877.2717, 879.0370, 884.3177, 885.9973, 893.0132, 901.3589, 908.9853, 924.9604, 1034.1259, 1044.0710, 1050.0869, 1053.4615, 1066.0652, 1067.4818, 1082.0472, 1098.0142, 1110.5029, 1136.5674, 1249.7527, 1300.0475, 1306.7865, 1320.5912, 1552.8657, 1642.7385, 1712.9301, 1765.0378, 1772.8123, 1779.0547, 1787.8590, 1864.0842, 1921.0986, 1922.0380, 1936.0398, 1983.9967, 1986.0781, 2043.1068, 2048.9885, 2125.9543, 2139.9622, 2141.9475, 2142.9429, 2146.0459, 2156.9597, 2170.0632, 2188.1106, 2226.1350, 2256.1982, 2289.1533, 2335.0740, 2387.2449, 2458.1199, 2472.1384, 2637.3391

## Search Parameters

**Type of search : Peptide Mass Fingerprint**
**Enzyme : Trypsin**
**Fixed modifications : Carbamidomethyl (C)**
**Variable modifications : Oxidation (M)**
**Mass values : Monoisotopic**
**Protein Mass : Unrestricted**
**Peptide Mass Tolerance : ± 50 ppm**
**Peptide Charge State : 1+**
**Max Missed Cleavages : 1**
**Number of queries : 65**
